# When more is less: Emergent suppressive interactions in three-drug combinations

**DOI:** 10.1186/s12866-017-1017-3

**Published:** 2017-05-06

**Authors:** Casey Beppler, Elif Tekin, Cynthia White, Zhiyuan Mao, Jeffrey H. Miller, Robert Damoiseaux, Van M. Savage, Pamela J. Yeh

**Affiliations:** 1 Department of Ecology and Evolutionary Biology, University of California, Los Angeles, CA USA; 2 Department of Biomathematics, University of California, David Geffen School of Medicine, Los Angeles, CA USA; 3 Department of Microbiology, Immunology, and Molecular Genetics, and the Molecular Biology Institute, University of California, David Geffen School of Medicine, Los Angeles, CA USA; 4 Department of Medical and Molecular Pharmacology, University of California, Los Angeles, CA USA; 5 Santa Fe Institute, Santa Fe, NM USA

**Keywords:** Higher-order interactions, Antagonism, Multiple stressors, Antibiotics, Drug resistance

## Abstract

**Background:**

In drug-drug interactions, there are surprising cases in which the growth inhibition of bacteria by a single antibiotic decreases when a second antibiotic is added. These interactions are termed suppressive and have been argued to have the potential to limit the evolution of resistance. Nevertheless, little attention has been given to suppressive interactions because clinical studies typically search for increases in killing efficiency and because suppressive interactions are believed to be rare based on pairwise studies.

**Results:**

Here, we quantify the effects of single-, double-, and triple-drug combinations from a set of 14 antibiotics and 3 bacteria strains, totaling 364 unique three-drug combinations per bacteria strain. We find that increasing the number of drugs can increase the prevalence of suppressive interactions: 17% of three-drug combinations are suppressive compared to 5% of two-drug combinations in this study. Most cases of suppression we find (97%) are “hidden” cases for which the triple-drug bacterial growth is less than the single-drug treatments but exceeds that of a pairwise combination.

**Conclusions:**

We find a surprising number of suppressive interactions in higher-order drug combinations. Without examining lower-order (pairwise) bacterial growth, emergent suppressive effects would be missed, potentially affecting our understanding of evolution of resistance and treatment strategies for resistant pathogens. These findings suggest that careful examination of the full factorial of drug combinations is needed to uncover suppressive interactions in higher-order combinations.

**Electronic supplementary material:**

The online version of this article (doi:10.1186/s12866-017-1017-3) contains supplementary material, which is available to authorized users.

## Background

Antibiotic resistance is a critical public health issue [1]. With few new antibiotics in development [2] combination therapies [3, 4] have been used to overcome the problem of drug-resistant bacteria. However, it can be difficult to determine which combinations are the most rational choices because several factors must be carefully balanced in order to identify exceptional therapeutic combinations. Efficiency of killing bacteria and toxicity to the patient are critical, but just as important, yet harder to measure and seldom considered, is the likelihood to facilitate the evolution of resistance to the drugs. While there are several factors that affect the likelihood and rate of resistance evolving, including mutation rate and rate of horizontal gene transfer, the strength of selection often plays an important role. With regard to this selection pressure, there is typically a critical tradeoff: highly efficient antibiotic combinations can be good for the individual patient, but might also create stronger selection pressures for the evolution of resistance [5]. Because these effects are often at odds with each other, identifying the best drug combinations is not trivial.

Drug-drug interactions further complicate finding the best drug combinations. Depending on whether the combination effect is greater than, equal to, or less than the effect expected based on individual drugs, the interaction is termed as synergistic, additive, or antagonistic, respectively [6–8]. Oftentimes, clinicians prescribe synergistic combinations because they have maximum killing efficiency [9]. In contrast, hyper-antagonistic interactions are not used in the clinic as they yield higher bacterial growth when two drugs are combined than at least one of the drugs does on its own. This special type of interaction has been termed a “suppressive” interaction [10, 11]. In a study of pairwise antibiotic interactions, suppression accounted for a nontrivial 16 of 180 interactions (9%) [11].

These suppressive combinations have traditionally been considered a poor treatment strategy because of their decreased killing efficiency—much higher concentration of drugs would be needed in order to achieve similar bacterial growth inhibition [12]. Thus, little attention has been given to suppression because of its obvious limitations in clinical practice. However, recent studies demonstrate that suppressive or antagonistic drug combinations can be used to combat evolution of drug resistance: suppressive combinations have been shown to select *against* resistance [13], decrease spontaneous evolution of resistance [14], and reduce the rate of adaptation [5]. Because of the tradeoffs between killing efficiency and selection against resistance, antagonistic combinations may provide the most favorable treatment option under some conditions [15]. In sum, there is intriguing and compelling evidence (although all in vitro and in silico) that suppressive combinations might be useful to combat resistance [8], but their rare occurrence makes their identification and use more daunting.

Although suppressive interactions have been examined in pairwise combinations of both antibiotic [11] and anti-fungal [16] compounds, there have been no systematic examinations of suppressive interactions in higher-order drug combinations. Indeed, three-drug interactions themselves have not been extensively studied (but see [17]), and the classification and understanding of emergent interactions—interactions that only arise when all three drugs are present—is still developing. Indeed, we note that it is possible that a higher-order, three-drug interaction can occur even when none of the three pairs of antibiotics exhibit interactions in isolation. This kind of interaction is truly “emergent” because all three drugs must be present to observe any sort of interaction.

Here, we take advantage of new data and mathematical methods [18, 19] to classify emergent interactions and show that novel suppressive interactions occur in three-drug combinations. Using wild-type non-pathogenic *Escherichia coli*, a pathogenic clinical isolate of *E. coli*, and a non-pathogenic strain of *Staphylococcus epidermidis,* we measured growth of bacteria in all possible single, pairwise, and three-drug combinations in a set of 14 antibiotics to find emergent interactions. It has been suggested that synergy and antagonism occur about equally in pairwise antibiotic combinations, with suppression being more infrequent [11]. While suppression for two-antibiotic combinations ranges from 5% (this study) to 9% in a previous study on two-drug combinations, our results suggest that adding a third drug may increase the relative number of suppressive interactions. Furthermore, we find “emergent” suppressive interactions in three-drug combinations that may reduce growth as compared to the single-drug effects, but not as compared to the effect of at least one pairwise combination. Because the suppressive effect of these combinations is only revealed by comparison to constituent pairwise combinations, we term these “hidden suppressors” and discuss possible implications for their use in better understanding and potentially mitigating tradeoffs between bacterial killing efficacy and evolution of resistance.

## Methods

### Bacteria and antibiotics

The primary *E. coli* strain used in these experiments is BW25113, the wild-type strain (*lacI* ^q^ *rrnB*_*T14*_ Δ*lacZ*_*WJ16*_ *hsdR514* Δ*araBAD*_*AH33*_ Δ*rhaBAD*_*LD78*_) [20] derived from the strain W1485 background [21]. BW25113 is the wild-type strain that was used to make the Keio Collection of single-gene knockouts and is a common lab strain used in a range of studies [22–24]). Additionally, drug combinations were also tested in (1) the pathogenic *E. coli* strain CFT073, a highly virulent pyelonephritis strain isolated from human clinical specimen (from ATCC) and (2) *Staphylococcus epidermidis* 14990 (from ATCC).

The 14 antibiotics in the study include: clindamycin, ciprofloxacin, tobramycin, streptomycin, cefoxitin, nitrofurantoin, ampicillin, erythromycin, gentamicin, chloramphenicol, vancomycin, fusidic acid, doxycycline, and trimethoprim (Table 1). We chose this set of antibiotics because they cover a range of mechanisms of action [25]. In addition, while some antibiotics such as vancomycin are ineffective by themselves in gram-negative bacteria, recent work has shown increased efficacy when used in combination with other drugs, making their effects in combination interesting for this study [26].

**Table 1:** Summary of antibiotics used in three-drug experiments

Drug	Drug abbreviation	Concentration range^a^ (xMIC)	MIC^a^(μg/ml)	Main mechanism(s) of Action	Origin	Cidal-static
Clindamycin	CLI	0.146–0.333	120	Protein synthesis, 50S	Semi-synthetic	Bacteriostatic
Erythromycin	ERY	0.080–0.400	150	Protein synthesis, 50S	Natural	Bacteriostatic
Chloramphenicol	CHL	0.203–0.374	187	Protein synthesis, 50S	Natural	Bacteriostatic
Fusidic acid	FUS	0.217–0.298	369	Protein synthesis, 50S	Natural	Bacteriostatic
Gentamicin	GEN	0.010–0.138	4	Protein synthesis, 30S, aminoglycoside	Natural	Bactericidal
Tobramycin	TOB	0.063–0.163	8	Protein synthesis, 30S, aminoglycoside	Natural	Bactericidal
Streptomycin	STR	0.056–0.278	18	Protein synthesis, 30S, aminoglycoside	Natural	Bactericidal
Doxycycline	DOX	0.182–0.318	2.2	Protein synthesis, 30S	Semi-synthetic	Bacteriostatic
Cefoxitin	FOX	0.150–0.193	6	Cell wall	Semi-synthetic	Bactericidal
Ampicillin	AMP	0.300–0.433	3	Cell wall	Semi-synthetic	Bactericidal
Vancomycin	VAN	<0.350–0.750	>100	Cell wall	Natural	Bactericidal
Nitrofurantoin	NTR	0.250–0.750	4	Multiple mechanisms, DNA	Synthetic	Bactericidal
Ciprofloxacin	CPR	0.200–0.375	0.04	DNA gyrase	Synthetic	Bactericidal
Trimethoprim	TMP	0.150–0.250	0.4	Folic acid biosynthesis	Synthetic	Bacteriostatic

^a^in wildtype, non-pathogenic *E. coli* BW25113

### Three-drug suppression experiments

Single-drug concentrations were chosen to reduce growth by ~15–35% as compared to the no-drug controls. In a few cases, growth was reduced by less than 15%. For example, the ciprofloxacin concentration reduced growth by 7% in *E. coli* BW25113. This was because it was difficult to obtain a consistent growth reduction percentage at higher drug concentrations. Another special case was vancomycin, which increased growth to 109% compared to no-drug growth in *E. coli* BW25113. (Some antibiotics in some bacteria populations result in hormesis, a phenomenon where populations grow slightly better at very low levels of a stressor agent such as an antibiotic [27]. It is not thoroughly understood, although it could be an effect of antibiotics being used as communication tools rather than warfare [28–30].) We used lower concentrations of vancomycin because a higher concentration would have resulted in population death of many two-drug combinations with vancomycin. If a population had been killed with two drugs, adding a third drug would not have yielded meaningful data regarding how three-drug combinations affect bacterial growth.

Growth in three-drug combinations was compared to growth in no-drug, the three single-drug, and the three two-drug conditions. Experiments were performed as described by Tekin et al. [19]. Briefly, we started with a single colony which was used to inoculate cultures for glycerol stocks stored at −80 °C. A single colony from this glycerol culture was used to inoculate cultures in LB media (10 g/l tryptone, 5 g/l yeast extract, and 10 g/l NaCl). These cultures were resuspended in MC buffer and stored at 4 °C. We then grew bacteria for experiments in an incubator shaker by inoculating 20 μl of the MC stock into 2 ml LB for 5 h at 37 °C. 25 μl of a 10^−4^ dilution of this culture in LB was used to inoculate into 975 μl of media with antibiotics. From this 1 ml mix of antibiotics and bacteria in growth media, we aliquoted 100 μl per well into 4–6 wells of a 96-well plate. We grew these cultures for 18 h at 37 °C and 215 rpm. From a single experiment, we took the means of the 4–6 wells. For the primary *E. coli* BW25113 strain, we repeated this entire experiment for each three-drug combination at least three times, and a minimum of two times at identical concentrations between experiments. Then, we took the median of these replicates at identical concentrations and used this value as our growth measurement. Thus, for each drug condition at identical concentrations, we had at least 8 samples total. Two independent experiments, each with at least 4 replicates (or 8 total samples), were used for verification in *E. coli* CFT073 and *S. epidermidis* 14990.

Following previous studies by the authors [26] and others (e.g. [31]), we took an OD_600_ measurement at 18 h after bacteria encountered antibiotics and compared this to bacteria grown in no-drug environments to obtain relative growth. This measurement can be described as the relative difference in OD_600_ measurements at 18 h between populations with drugs and no-drug controls. Below, this is the measurement we refer to when we compare bacteria growth among populations. This growth measurement served as a reasonable proxy for other comparable measurements, including both growth rate (Additional file 1: Figure S1) and number of colony forming units (Additional file 1: Figure S2).

## Measurement of suppression

### Two-drug interactions

Pairwise suppressive interactions in terms of relative fitnesses were defined as described in Yeh et al. and Segre et al. [11, 32] as

$$ {\left[{\mathrm{DA}}_{\mathrm{X},\mathrm{Y}}\right]}_s=\frac{w_{\mathrm{X}\mathrm{Y}}-{w}_{\mathrm{X}}{w}_{\mathrm{Y}}}{\left| \min \left({w}_{\mathrm{X}},{w}_{\mathrm{Y}}\right)-{w}_{\mathrm{X}}{w}_{\mathrm{Y}}\right|} $$

where *w*_X_ is the fitness (measured as growth) of the bacteria population in the presence of drug X, *w*_Y_ is the fitness of the bacteria population in the presence of drug Y, and *w*_XY_ is the fitness of the population in the presence of both drugs X and Y. Here, DA_X , Y_ = *w*_XY_ − *w*_X_ *w*_Y_ quantifies the deviation from expectation that two drugs do not interact, and the sign of this expression yields whether an interaction is synergistic (DA_X , Y_ < 0) or antagonistic (DA_X , Y_ > 0). The scale factor |min(*w*_X_, *w*_Y_) − *w*_X_ *w*_Y_| applied to the positive DA_X , Y_ further quantifies the degree of antagonism [11, 32–34], hence leading to a concrete criteria to determine suppressive interaction. According to this definition, suppressive interactions are those for which [DA_X , Y_]_*s*_ > 1.15. When the maxima for both single growth and pairwise growth were greater than 90%, cases were excluded from calculations of pairwise suppression as the effect of the second drug on the growth is not obvious due to variation in growth measurements.

### Three-drug interactions

Three-drug suppression was defined according to the emergent three-way interaction method [17, 18, 35] that distinguishes the pairwise interaction effects from the overall three-way interaction. According to this method, the emergent three-way interaction measure in terms of relative fitnesses is E3_X , Y , Z_ = *w*_XYZ_ − *w*_X_ *w*_YZ_ − *w*_Y_ *w*_XZ_ − *w*_Z_ *w*_XY_ + 2*w*_X_ *w*_Y_ *w*_Z_, where X, Y and Z represents the combined drugs. Note that the unscaled interaction measures defined for the two- and three-drug interactions, i.e. DA_X , Y_ and E3_X , Y , Z_, are analogous to second- and third-order cumulants, which are widely used in the areas of theoretical physics [36] and statistics [37].

The interaction strength is further quantified by implementing a rescaling method introduced by Tekin et al. [19]—rescaling introduced for two-drug interactions [32] is extended and further developed for higher-order interactions, and shown to enhance the characterization of emergent properties. Accordingly, when E3 is greater than zero, corresponding to an antagonistic interaction, it is rescaled as

$$ {\left[\mathrm{E}3\right]}_s=\frac{w_{\mathrm{X}\mathrm{Y}\mathrm{Z}}-{w}_{\mathrm{X}}{w}_{\mathrm{Y}\mathrm{Z}}-{w}_{\mathrm{Y}}{w}_{\mathrm{X}\mathrm{Z}}-{w}_{\mathrm{Z}}{w}_{\mathrm{X}\mathrm{Y}}+2{w}_{\mathrm{X}}{w}_{\mathrm{Y}}{w}_{\mathrm{Z}}}{\left| \min \left({w}_{\mathrm{X}},{w}_{\mathrm{Y}},{w}_{\mathrm{Z}},{w}_{\mathrm{X}\mathrm{Y}},{w}_{\mathrm{X}\mathrm{Z}},{w}_{\mathrm{Y}\mathrm{Z}}\right)-{w}_{\mathrm{X}}{w}_{\mathrm{Y}\mathrm{Z}}-{w}_{\mathrm{Y}}{w}_{\mathrm{X}\mathrm{Z}}-{w}_{\mathrm{Z}}{w}_{\mathrm{X}\mathrm{Y}}+2{w}_{\mathrm{X}}{w}_{\mathrm{Y}}{w}_{\mathrm{Z}}\right|} $$

As shown in Tekin et al. [19], a histogram of all rescaled emergent three-way interaction ([E3]_*s*_) measures yields a multimodal distribution. Based on the location of the peaks of the distribution, suppressive interactions are those combinations for which [E3]_*s*_>1.30 [19]. The cutoff values for two- and three-drug interactions (i.e. 1.15 and 1.30, respectively) are chosen consistently based on natural breakpoints in the resulting distribution of interaction metrics. We note that the error or variability of data might be amplified because rescaling methods involve division and subtraction. The best way to avoid this is through carefully collected data with high replication.

For triple-antibiotic interaction assays in which a pairwise combination results in lethality of the two- and three-drug conditions, the [E3]_*s*_ measure is non-applicable because it is impossible to determine the effect of the addition of the third drug, although we note that suppression could be found if two drugs kill a bacteria population and the addition of a third drug allowed the population to grow, “synthetic rescue.” Based on error in OD_600_ measurements as determined by Tekin et al. [19], growth measurements below 4.7% are defined as lethal. For *E. coli* BW25113, we found 37 such cases (see [19] and additional data in Additional file 1: Table S1). Thus, we examined the number of suppressive interactions out of a total 327 (rather than 364) [E3]_*s*_ measures. For *E. coli* CFT073 and *S. epidermidis* 14990, the [E3]_*s*_ measure was applicable in 356 and 313 cases, respectively.

Due to the full factorial design of our experiments, many more two-drug combinations were measured than three-drug combinations. In measuring the number of pairwise suppressive interactions in three-drug combinations for each bacteria strain, we first measured the interaction for each two-drug experiment. For example, in *E. coli* BW25113 for each two-drug combination, an average of 10.9 experiments were conducted, ranging from 6 to 12. The median interaction metric calculations (i.e. [DA_X , Y_]_*s*_) across these experiments was used to determine the interaction type of the two-drug combination.

## Results and discussion

**Fig. 1:**
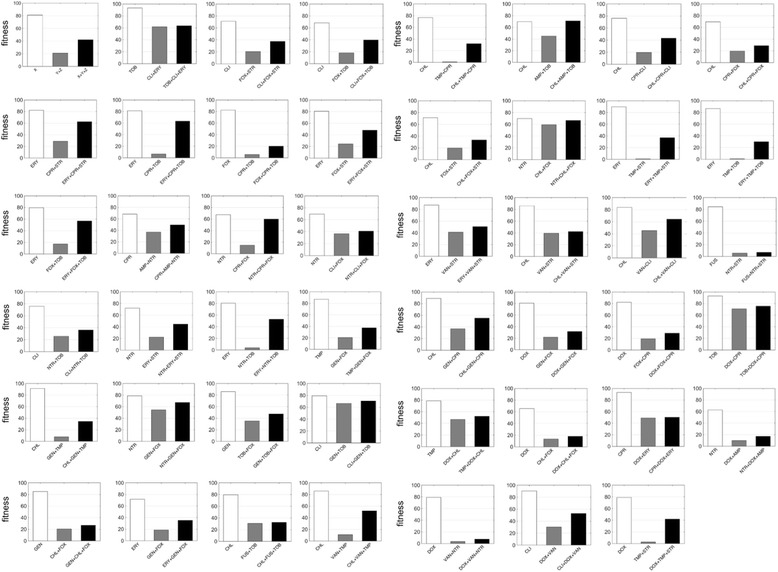
Suppressive three-drug interactions in *E. coli* BW25113. Growth measurements are shown for bacteria in single-drug, two-drug, and three-drug conditions relative to the no-drug control (100% growth, not shown). Emergent suppression was determined following Tekin et al. [19] (see Methods). The upper left figure is a schematic with X, Y, and Z representing three different drugs. Only the experimental data for the three-drug combination (X + Y + Z), the suppressed pairwise drug combination with the lowest growth (Y + Z), and the suppressor single drug (X) is shown. 46 triple combinations of a total 327 interaction measures were determined to be suppressive. Antibiotic abbreviations are as listed in Table 1

To identify suppressive emergent three-way interactions (E3_X , Y , Z_), we tested growth under the single-, double-, and triple-drug conditions in wild-type non-pathogenic *E. coli* (Additional file 1: Figure S3). In nearly all cases – 45 out of 46 suppressive three-drug combinations (97.8%) – the growth of the triple combination exceeded the growth of at least one of the pairwise combinations, but did not exceed growth in any of the single-drug conditions. Thus, in Fig. 1, which is a simplification of Additional file 1: Figure S3 to save space, we only show experimental data for the three-drug combination, the suppressed pairwise combination with the lowest growth, and the remaining single drug that was not part of the suppressed pair. See Additional file 1: Table S2 for all emergent interaction measures.

**Fig. 2:**
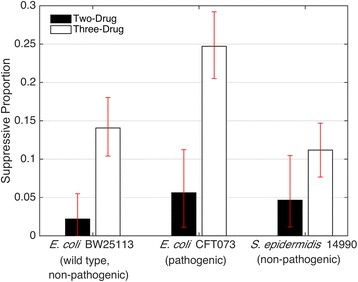
Comparison of suppressive interactions in pairwise versus emergent three-drug interactions. Bars represent the proportion of interactions that are classified as antagonistic suppressive in pairwise interactions (black) and in three-drug interactions (white) for three bacteria strains: wild-type, non-pathogenic *E. coli* BW25113; pathogenic *E. coli* CFT073; and non-pathogenic *S. epidermidis* 14990. For the data presented in this paper, 95% confidence intervals resulting from bootstrapping experiments are shown

In three-drug combinations in the wild-type non-pathogenic *E. coli* strain, 14% (46 of 327 cases) of interactions were suppressive. In contrast, 2.2% (2 of 91) of two-drug combinations were suppressive (Fig. 2). There was a significant difference in the percentage of suppressive interactions in two- and three-drug combinations (z: 3.141, *p*-value = 0.0017). Intriguingly, we found that this pattern—antagonistic suppression is significantly more common among three-drug combinations relative to two-drug combinations—is consistent across different bacterial strains included in our study (see below for more discussion).

**Fig. 3:**
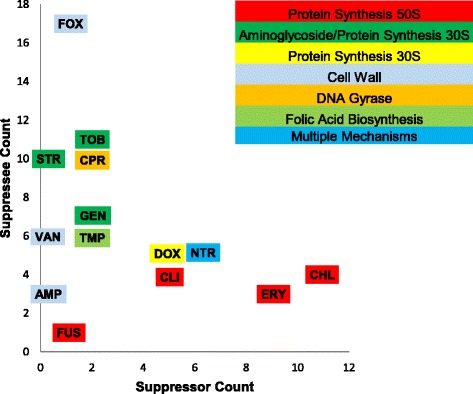
Suppressor and suppressee antibiotics. For each antibiotic, the number of suppressive interactions in *E. coli* BW25113 in which it acts as the suppressor (x axis) and the suppressee (y axis) are plotted. Antibiotic abbreviations are as listed in Table 1

For each drug, we also examined the number of times they were the suppressor (e.g. drug X in Fig. 1) compared with the number of times they were the suppressee (e.g. drugs Y or Z in Fig. 1). We did not find a significant effect, though there was a trend towards a negative correlation (Fig. 3, *R* = −0.375, *N* = 14, one-tailed *p*-value = 0.094).

Our finding that there was an increase in the percentage of suppressive interactions when the number of drugs used in a combination was increased is an intriguing result for several reasons. First, this suggests that clinicians should be cautious in assigning drugs for combination therapies, as one drug may have a negative impact on killing efficacy. On the other hand, there may be potential benefits for using suppressive combinations, such as slowing the evolution of resistance as previously discussed [5, 8, 13, 14].

Second, nearly all (97.8%) of the suppressive emergent interactions found in the BW25113 strain were “hidden suppressors” because they reduced growth as compared to single-drug controls, but exceeded the growth of a pairwise combination. These were “hidden” because if one did not experimentally examine two-drug growth, this suppressive effect would not be found. Suppression has been shown to be more effective at slowing the evolution of resistance both theoretically, and empirically in vitro [5, 8, 13] at least under some environments. Therefore, determining how suppression in three-drug combinations affects fitness landscapes and the evolution of resistance could be an extremely useful step (albeit one of many) towards clinical application. More generally, we need to better understand the tradeoff between slowing the evolution of resistance and increasing the bacterial killing efficiency of these combinations.

Because the effects of an antibiotic combination can vary between different bacterial strains, the results from experiments in the wild-type non-pathogenic *E. coli* were compared to three-drug combination results in two other bacteria strains: (1) pathogenic *E. coli* strain CFT073, and (2) *Staphylococcus epidermidis* 14990 using the same 14 antibiotics (Additional file 1: Figures. S4 and S5, Additional file 1: Tables S1 and S2). In *E. coli* strain CFT073, 8% of pairwise combinations were identified as suppressive, compared to 25% of triple combinations (z: 3.543, *p* = 0.0004). In *S. epidermidis* 14990, 5% of pairwise combinations were suppressive, compared to 11% of triple combinations (z: 1.806, *p* = 0.0709). Over three strains, 5% of pairwise were suppressive compared with 17% of triple (z: 5.016, *p* < 0.0001). With this data, we concluded that the general pattern that three-drugs have greater suppression cases than two-drugs still holds (Fig. 2).

Critically, as this research was conducted at sub-inhibitory concentrations following many other interaction studies [11, 13, 18, 26, 38–40], further research would be needed to determine if bacteria load would actually be reduced with suppressive drug combinations at higher drug concentrations. In addition, as shown by Chait and colleagues [13] only certain concentrations yield suppressive interactions, so interactions must also be tested across a broad range of concentrations. Therefore, before translation of these ideas to the clinic, several other substantial steps would be needed given the variance in antibiotic concentration that is expected in vivo, such as the careful consideration of concentrations, pharmacodynamics, and pharmacokinetics. Given that work on suppressive antibiotic combinations has been entirely based on in vitro and in silico studies, one critical test would be to confirm these findings using an animal infection model.

In all suppressive cases in this study, the triple-drug combination growth exceeded the growth of at least one pairwise combination, as consistent by definition. Intriguingly, we found only 5 non-hidden suppressor cases across 169 suppressive interactions in 3 bacteria strains (3%) in which the triple-drug combination growth exceeded a single-drug growth condition. It is possible that such cases would be more frequently identified by using higher antibiotic dosages, corresponding to lower growth of bacteria for which the suppressive effect would be most clearly observed. That is, because our single-drug concentrations were chosen to reduce growth only by 15–35%, it is not surprising that most triple-drug combination growth did not exceed the growth of a single-drug condition.

Our analysis shows that, within this study, there is a higher proportion of suppressive interactions in three-drug combinations across three bacteria strains (17%) than in two-drug combinations (5%). We also compared our results to Yeh and colleagues [11], which followed similar suppression criteria as in our study. These data revealed that suppressive pairwise interactions are also relatively infrequent, with only 9% showing suppressive effects.

Determining the mechanism of suppression can be very challenging. In a seminal paper on suppressive mechanisms, Bollenbach and colleagues showed that there is an optimal ratio between protein synthesis and DNA replication, and when only one of these processes is hampered, growth rates of the bacteria would be lower than when both processes are hampered [38]. Thus, many protein synthesis inhibitors and DNA synthesis inhibitors in combination could produce higher growth than just a single inhibitor by itself, leading to the observed suppressive interaction. While there are likely different mechanisms in other suppressive combinations [16, 41], there have been no studies to date that have presented alternative hypotheses and supporting data on mechanisms of suppression.

One intriguing study showed that bacteriostatic and bactericidal combinations often result in antagonism [31]. As suppression is an extreme form of antagonism, we examined whether our suppressive interactions had significantly higher rates of cidal-static combinations. Out of all three-drug combinations from 14 drugs, where eight were bactericidal and six were bacteriostatic, there were 288 (or 79%) combinations that involved at least one of each type of drug in the wildtype, non-pathogenic *E. coli* strain. Our data showed that 40 of 46 (or 87%) suppressive combinations involved at least one bactericidal and one bacteriostatic. This was not significantly different than expected (chi-square = 1.755, *p*-value = 0.1852), although statistical power was low.

It is possible that there are hubs of suppressive activity, that is, some drugs are much more likely to be involved in suppressive interactions as either suppressors or suppressees [16]. We found that, in wild-type *E. coli*, some drugs were much more likely to be suppressors (e.g., erythromycin and chloramphenicol) whereas other drugs were more likely to be suppressees (e.g., cefoxitin, tobramycin, and ciprofloxacin) (Fig. 3). This finding supports previous research that indicates that some drugs are much more likely to be involved in suppressive interactions [16, 38]. Interestingly, we did not find a significant correlation between these two groups, i.e. suppressor and suppressee, but patterns suggested a trend towards negative correlation, meaning that a drug was typically only a suppressor or a suppressee. This was consistent with what previous work has found, where protein synthesis inhibitors are the suppressors and DNA synthesis inhibitors the suppressees in combinations of these two classes of drugs [38]. However, this pattern is not consistent in the two other bacteria strains studied (Additional file 1: Figure S5).

In addition, in the wild-type BW25113 *E. coli*, the number of times a drug was a suppressee appeared to be roughly constant based on mechanism of action, and regardless of how many times it was a suppressor (and vice versa). While our sample size was small, this was suggestive that mechanism of action could be a significant predictor of suppressive interactions in three-drug combinations. This would be consistent with two-drug suppressive interactions, where certain combinations of mechanisms of action (e.g., DNA synthesis inhibitor and protein synthesis inhibitors) are more likely to result in suppression [11, 13, 38].

Exploration of mechanisms involving higher-order suppressive interactions could facilitate our understanding of how suppression translates from two-drug to higher-order combinations within drug combinations. Furthermore, other analogous forms of suppression occur in higher-order interactions in microbial communities, such as in species-species interactions or gene-gene interactions. For example, the presence of a third species can result in stable communities by affecting the interaction between the other two species (e.g. [42]), and combinations of alleles affect phenotypes such that some genes effectively suppress others in yeast populations exposed to different temperatures (e.g. [43]). Future work examining the prevalence, patterns, mechanisms, and types of suppression will increase our understanding of interactions within microbial communities.

Given the paucity of novel antibiotics in development [2] alternative strategies to overcome the problem of drug-resistant bacteria, such as combination treatments, must be considered. Another proposed strategy employs bacteriophages to either directly kill bacteria [44, 45], or to introduce genes that reverse antibiotic resistance [46]. The problem of resistance in bacteriophage therapies is further complicated by the ability of the virus to act as a vector for gene transfer, potentially arming the bacteria with antibiotic resistance or virulence genes [47]. Other strategies take advantage of virally-derived proteins that target bacteria [48] or non-viral predators of pathogenic bacteria [49]. In all cases, best practices must be considered to avoid the evolution of resistance to these new strategies. Our data highlights the need for further research in order to determine if suppressive combinations are of use in the fight against antibiotic resistance.

## Additional file

Additional file 1:
** Figure S1.** Comparison of relative optical density measurement to growth rate measurement. **Figure S2.** Comparison of relative optical density measurement to colony forming units. **Figure S3.** Emergent three-way interaction measures in *E. coli* BW25113. **Figure S4.** Suppressive three-drug interactions in *S. epidermidis* 14990 and *E. coli* CFT073. **Figure S5.** Suppressor and suppressee antibiotics for *S. epidermidis* 14990 and *E. coli* CFT07. **Table S1.** Full data set for 14 drugs in *E. coli* BW25113, *S. epidermidis* 14990, and *E. coli* CFT073. **Table S2.** Emergent suppressive three-drug combinations from 14 antibiotics (see Methods, Fig. 1) for *E. coli* CFT073, *E. coli* BW25113, *S. epidermidis* 14990. (PDF 7668 kb)
