# Supplementary material for: When more is less: Emergent suppressive interactions in three-drug combinations
Source: BMC Microbiol. 2017 May 6;17:107. doi: 10.1186/s12866-017-1017-3 (PMC5420147; doi:10.1186/s12866-017-1017-3)
Supplement: Additional file 1: — Figure S1. Comparison of relative optical density measurement to growth rate measurement. Figure S2. Comparison of relative optical density measurement to colony forming units. Figure S3. Emergent three-way interaction measures in E. coli BW25113. Figure S4. Suppressive three-drug interactions in S. epidermidis 14990 and E. coli CFT073. Figure S5. Suppressor and suppressee antibiotics for S. epidermidis 14990 and E. coli CFT07. Table S1. Full data set for 14 drugs in E. coli BW25113, S. epidermidis 14990, and E. coli CFT073. Table S2. Emergent suppressive three-drug combinations from 14 antibiotics (see Methods, Fig. 1) for E. coli CFT073, E. coli BW25113, S. epidermidis 14990. (PDF 7668 kb) [file 12866_2017_1017_MOESM1_ESM.pdf]

Supplementary Materials

When more is less: emergent suppressive interactions in three-drug combinations

Casey Beppler, Elif Tekin, Cynthia White, Zhiyuan Mao, Jeffrey H. Miller, Robert Damoiseaux, Van M. Savage, and Pamela J. Yeh

Supplementary Figure 1. Comparison of relative optical density measurement to growth rate measurement.

We performed experiments using 0.5, 0.75, 1, 1.25, 1.5 and 1.75 µg/ml streptomycin in *E. coli* BW25113 and measured growth relative to the no-drug control every hour to determine the growth rate, i.e. the slope. We compared this with the result of OD_{600} measurement (relative to no-drug controls) at a single time point (18 hours). We found a correlation of r=0.74 and P < 0.00001.

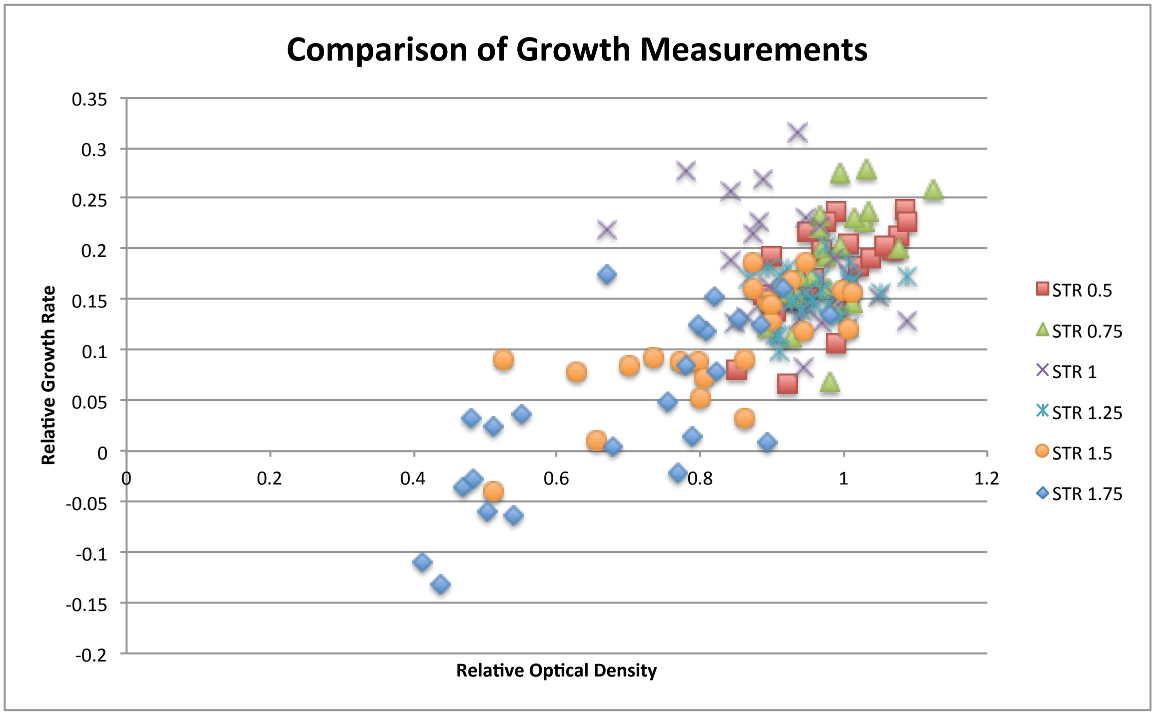

Supplementary Figure 2. Comparison of relative optical density measurement to colony forming units.

We performed experiments in *E. coli* BW25113 using streptomycin, erythromycin, cefoxitin, tobramycin, and clindamycin with 2-3 different concentrations per drug to compare counts based on colony forming units vs. OD_{600} measurement at 18 hours (relative to no-drug controls). The concentrations (in µg/ml) were as follows: streptomycin 4.1, 5.7, and 11; erythromycin 75 and 121; cefoxitin 1.5, 2.9, and 9; clindamycin 60 and 75; and tobramycin 6, 4.8, and 3.8. This comparison yielded a correlation rate of 93% ($n=14$, $P < 0.0001$).

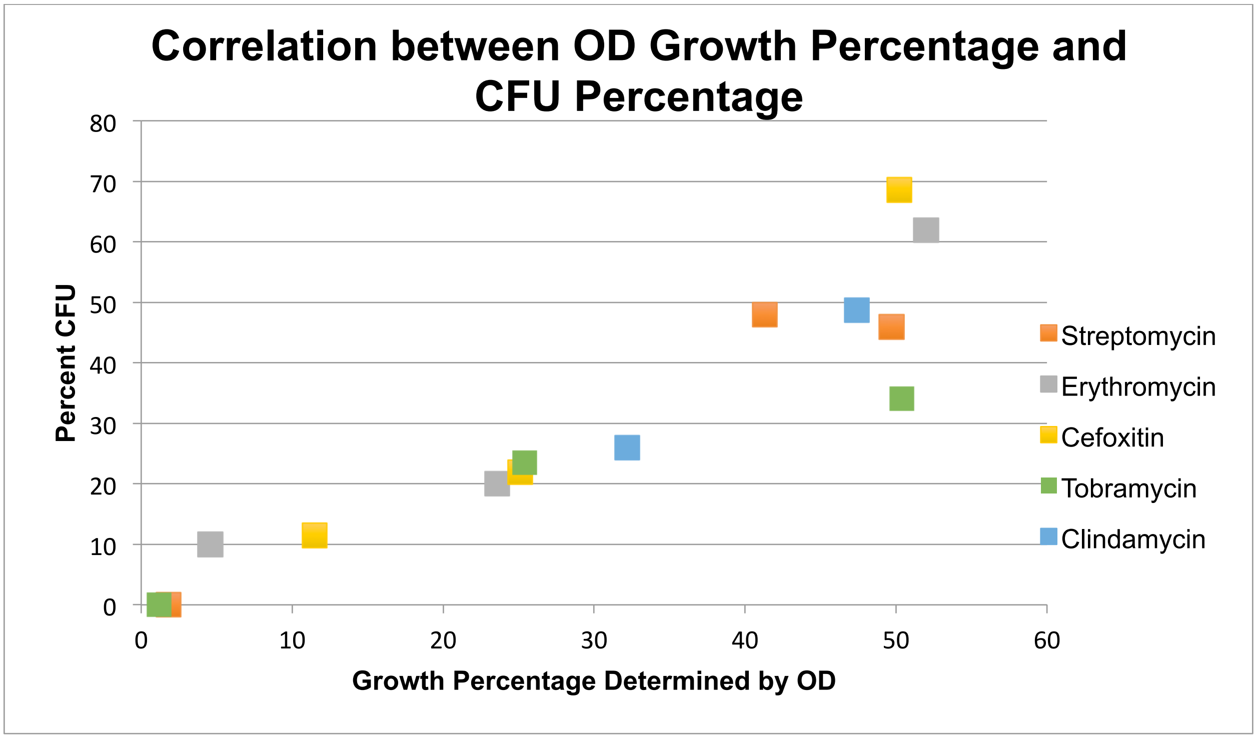

Supplementary Figure 3. Emergent three-way interaction measures in *E. coli* BW25113.

All 46 three-drug suppressive combinations showing growth percentage in different drug environments: all three single drugs, all two-drug combinations, and the single three-drug combination. X-axis indicates the drug environment, and Y-axis indicates the growth rate relative to bacteria grown in no-drug environment. In a few of the cases, we find multiple levels of suppression in a single three-drug combination: two drugs are suppressing one or both drugs when examining pairwise and single drug effects, and three drugs suppress one of the two-drug pairs when examining higher-order effects. For example, in the experiments involving DOX, NTR, and AMP, DOX-NTR suppresses both DOX by itself and NTR by itself, and DOX-NTR-AMP suppresses DOX-AMP. We found no cases where the two-drug suppressive combination was the one being suppressed in the three-drug suppressive combination.

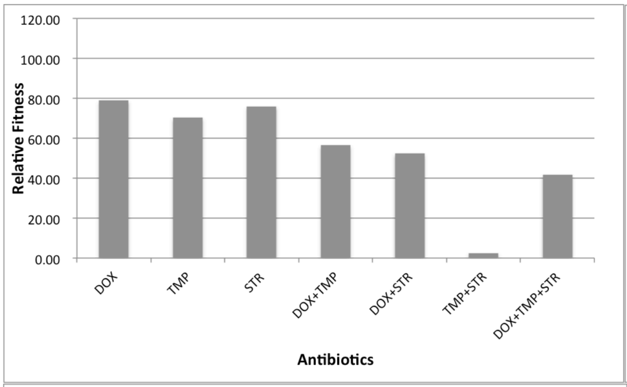
120.00
100.00
80.00
60.00
40.00
20.00
0.00
Relative Fitness
DOX
TMP
STR
DOX+TMP
DOX+STR
TMP+STR
DOX+TMP+STR
Antibiotics

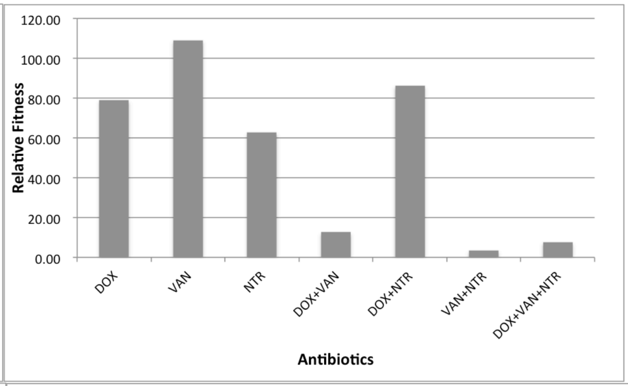
120.00
100.00
80.00
60.00
40.00
20.00
0.00
Relative Fitness
DOX
VAN
NTR
DOX+VAN
DOX+NTR
VAN+NTR
DOX+VAN+NTR
Antibiotics

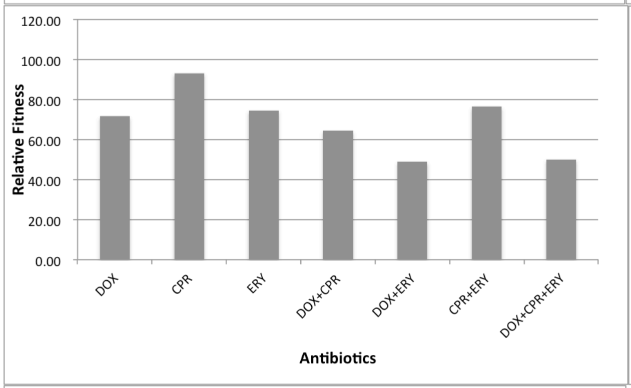
120.00
100.00
80.00
60.00
40.00
20.00
0.00
Relative Fitness
DOX
CPR
ERY
DOX+CPR
DOX+ERY
CPR+ERY
DOX+CPR+ERY
Antibiotics

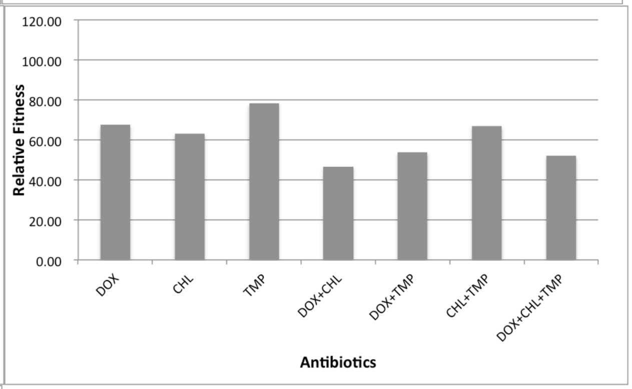
120.00
100.00
80.00
60.00
40.00
20.00
0.00
Relative Fitness
DOX
CHL
TMP
DOX+CHL
DOX+TMP
CHL+TMP
DOX+CHL+TMP
Antibiotics

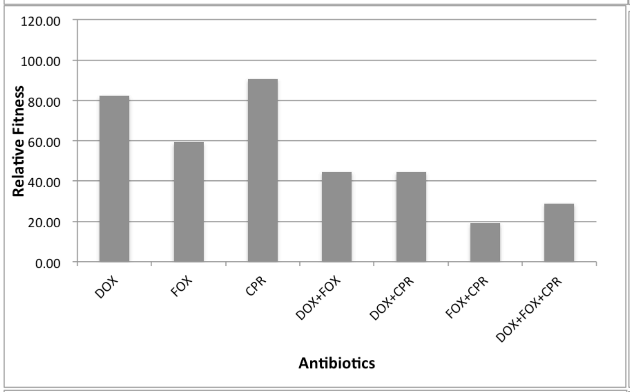
120.00
100.00
80.00
60.00
40.00
20.00
0.00
Relative Fitness
DOX
FOX
CPR
DOX+FOX
DOX+CPR
FOX+CPR
DOX+FOX+CPR
Antibiotics

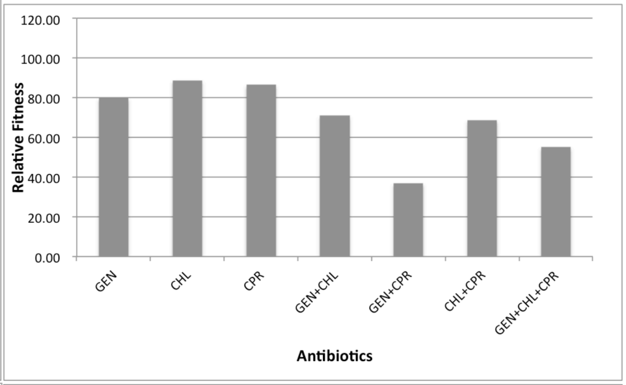
120.00
100.00
80.00
60.00
40.00
20.00
0.00
Relative Fitness
GEN
CHL
CPR
GEN+CHL
GEN+CPR
CHL+CPR
GEN+CHL+CPR
Antibiotics

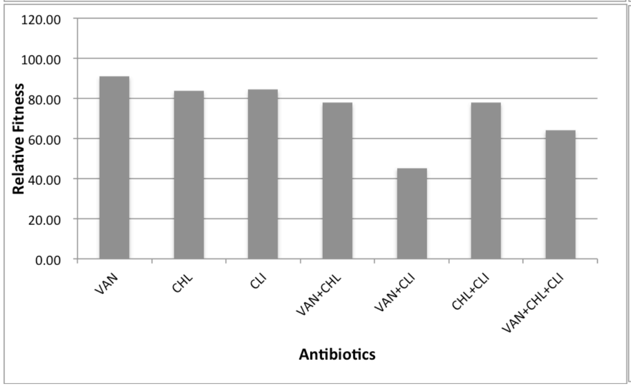
120.00
100.00
80.00
60.00
40.00
20.00
0.00
Relative Fitness
VAN
CHL
CLI
VAN+CHL
VAN+CLI
CHL+CLI
VAN+CHL+CLI
Antibiotics

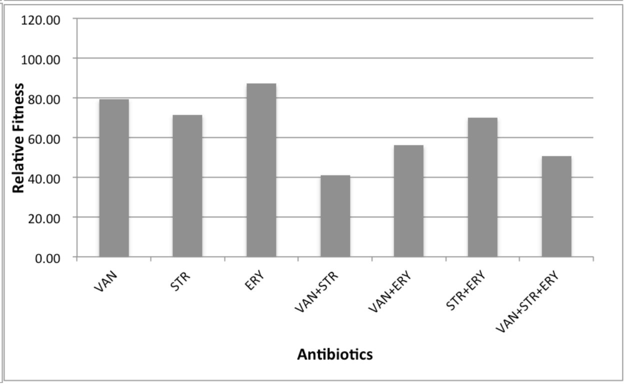
120.00
100.00
80.00
60.00
40.00
20.00
0.00
Relative Fitness
VAN
STR
ERY
VAN+STR
VAN+ERY
STR+ERY
VAN+STR+ERY
Antibiotics

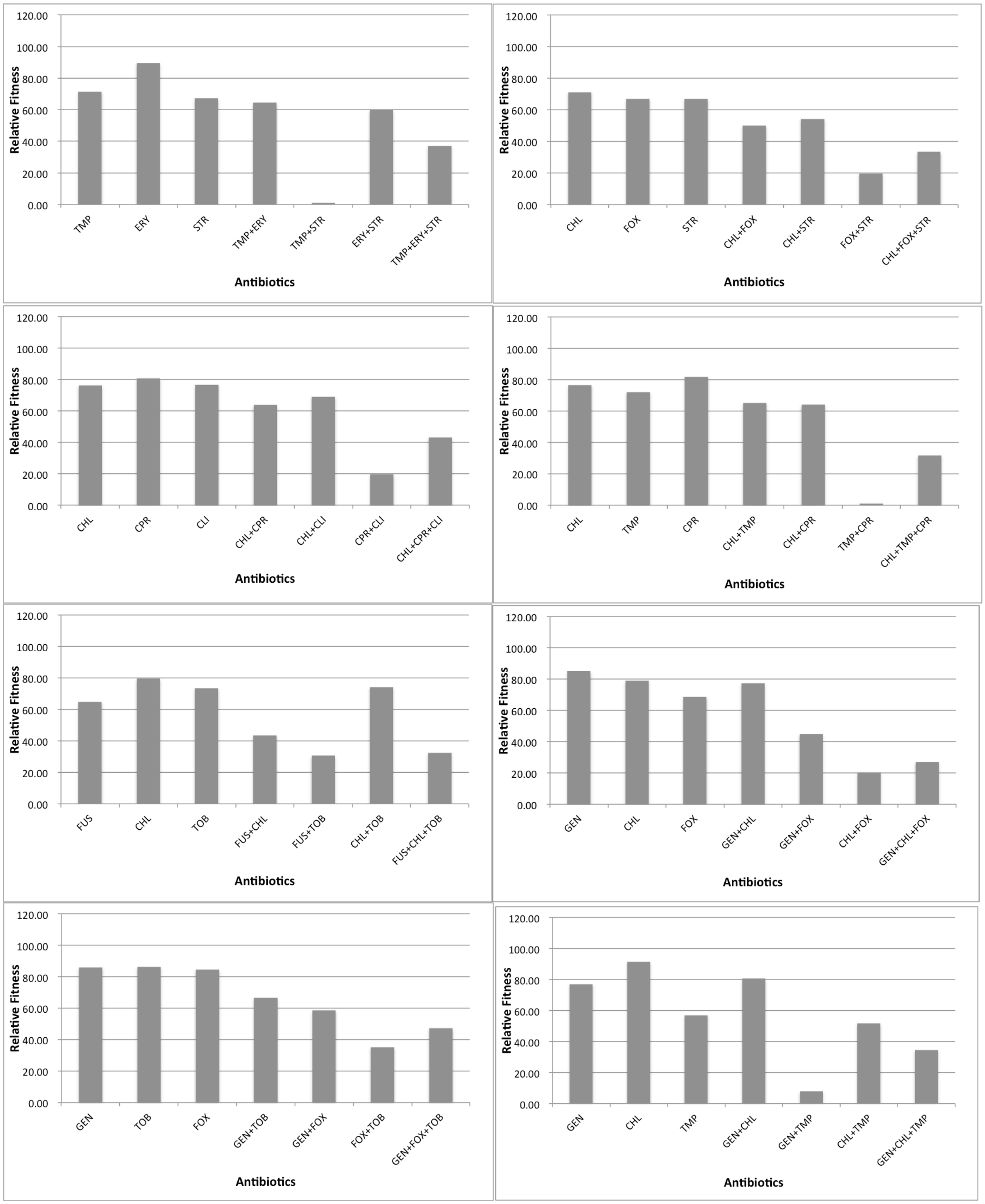
Relative Fitness
Antibiotics
TMP
ERY
STR
TMP+ERY
TMP+STR
ERY+STR
TMP+ERY+STR
CHL
FOX
STR
CHL+FOX
CHL+STR
FOX+STR
CHL+FOX+STR
CHL
CPR
CLI
CHL+CPR
CHL+CLI
CPR+CLI
CHL+CPR+CLI
CHL
TMP
CPR
CHL+TMP
CHL+CPR
TMP+CPR
CHL+TMP+CPR
FUS
CHL
TOB
FUS+CHL
FUS+TOB
CHL+TOB
FUS+CHL+TOB
GEN
CHL
FOX
GEN+CHL
GEN+FOX
CHL+FOX
GEN+CHL+FOX
GEN
TOB
FOX
GEN+TOB
GEN+FOX
FOX+TOB
GEN+FOX+TOB
GEN
CHL
TMP
GEN+CHL
GEN+TMP
CHL+TMP
GEN+CHL+TMP
0.00
20.00
40.00
60.00
80.00
100.00
120.00

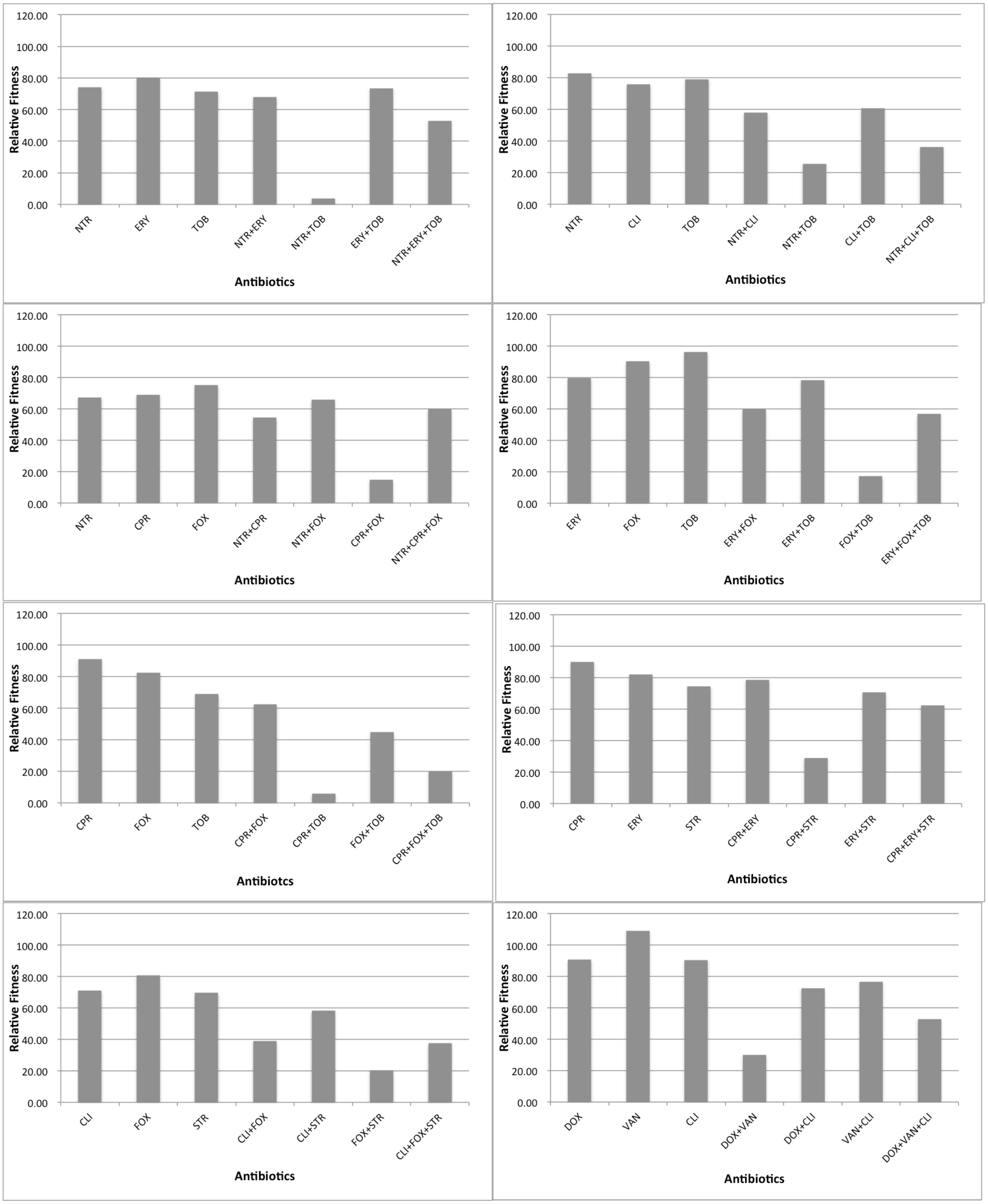
120.00
100.00
80.00
60.00
40.00
20.00
0.00
Relative Fitness
NTR
ERY
TOB
NTR+ERY
NTR+TOB
ERY+TOB
NTR+ERY+TOB
Antibiotics
Relative Fitness
NTR
CLI
TOB
NTR+CLI
NTR+TOB
CLI+TOB
NTR+CLI+TOB
Antibiotics
Relative Fitness
NTR
CPR
FOX
NTR+CPR
NTR+FOX
CPR+FOX
NTR+CPR+FOX
Antibiotics
Relative Fitness
ERY
FOX
TOB
ERY+FOX
ERY+TOB
FOX+TOB
ERY+FOX+TOB
Antibiotics
Relative Fitness
CPR
FOX
TOB
CPR+FOX
CPR+TOB
FOX+TOB
CPR+FOX+TOB
Antibiotcs
Relative Fitness
CPR
ERY
STR
CPR+ERY
CPR+STR
ERY+STR
CPR+ERY+STR
Antibiotics
Relative Fitness
CLI
FOX
STR
CLI+FOX
CLI+STR
FOX+STR
CLI+FOX+STR
Antibiotics
Relative Fitness
DOX
VAN
CLI
DOX+VAN
DOX+CLI
VAN+CLI
DOX+VAN+CLI
Antibiotics

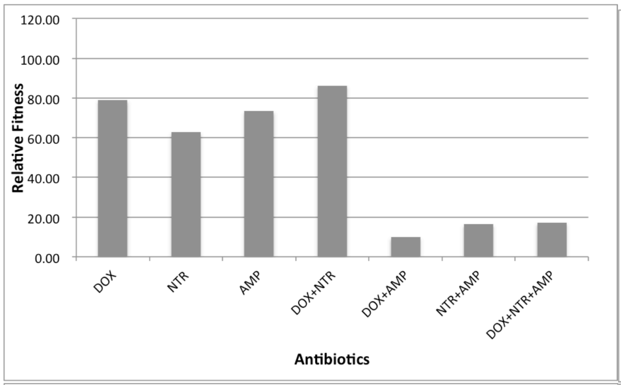
120.00
100.00
80.00
60.00
40.00
20.00
0.00
Relative Fitness
DOX
NTR
AMP
DOX+NTR
DOX+AMP
NTR+AMP
DOX+NTR+AMP
Antibiotics

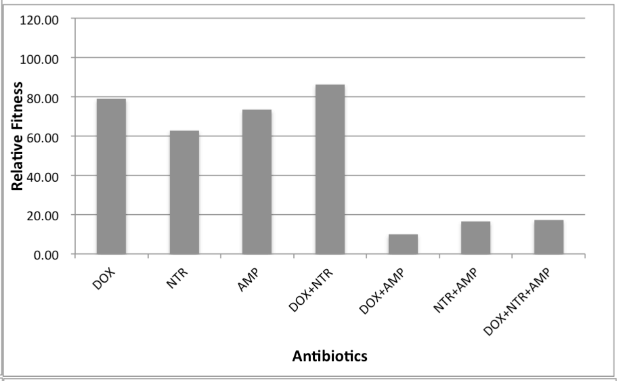
120.00
100.00
80.00
60.00
40.00
20.00
0.00
Relative Fitness
DOX
NTR
AMP
DOX+NTR
DOX+AMP
NTR+AMP
DOX+NTR+AMP
Antibiotics

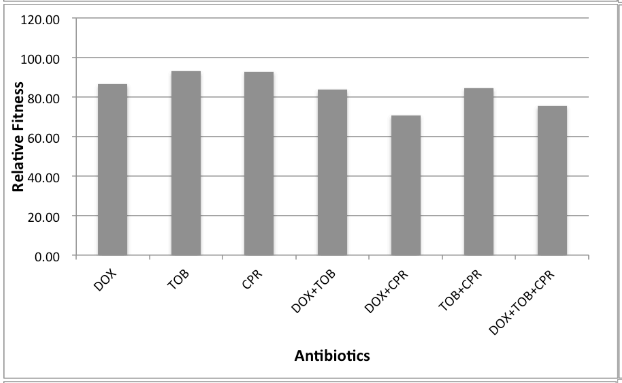
120.00
100.00
80.00
60.00
40.00
20.00
0.00
Relative Fitness
DOX
TOB
CPR
DOX+TOB
DOX+CPR
TOB+CPR
DOX+TOB+CPR
Antibiotics

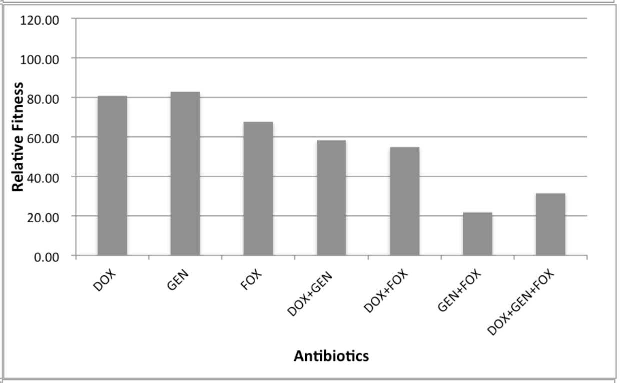
120.00
100.00
80.00
60.00
40.00
20.00
0.00
Relative Fitness
DOX
GEN
FOX
DOX+GEN
DOX+FOX
GEN+FOX
DOX+GEN+FOX
Antibiotics

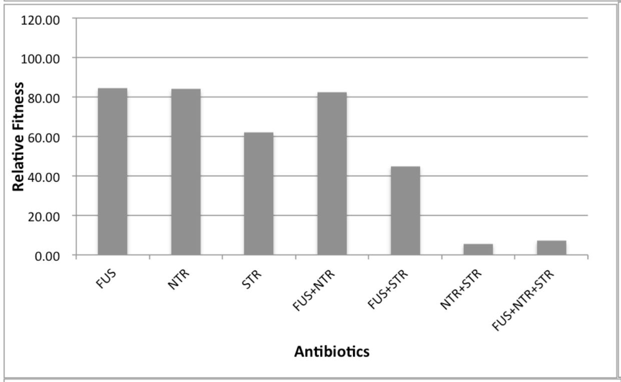
120.00
100.00
80.00
60.00
40.00
20.00
0.00
Relative Fitness
FUS
NTR
STR
FUS+NTR
FUS+STR
NTR+STR
FUS+NTR+STR
Antibiotics

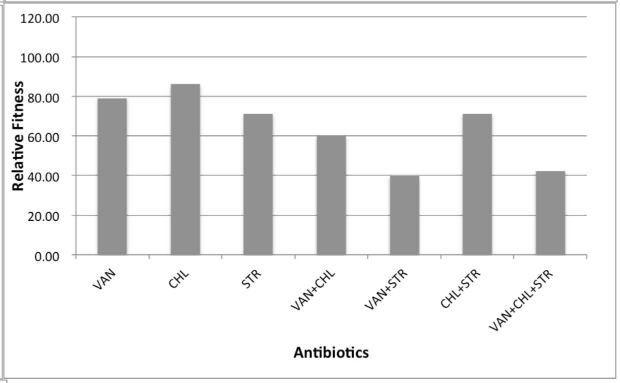
120.00
100.00
80.00
60.00
40.00
20.00
0.00
Relative Fitness
VAN
CHL
STR
VAN+CHL
VAN+STR
CHL+STR
VAN+CHL+STR
Antibiotics

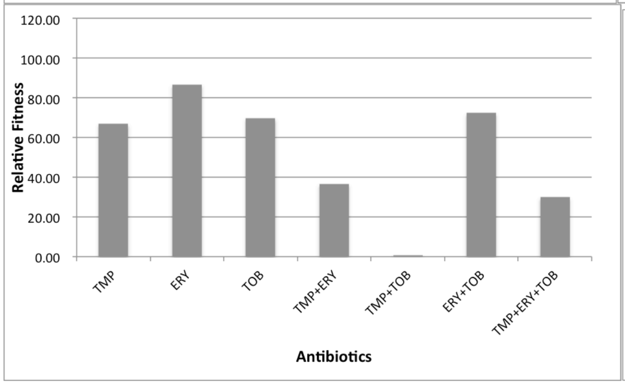
120.00
100.00
80.00
60.00
40.00
20.00
0.00
Relative Fitness
TMP
ERY
TOB
TMP+ERY
TMP+TOB
ERY+TOB
TMP+ERY+TOB
Antibiotics

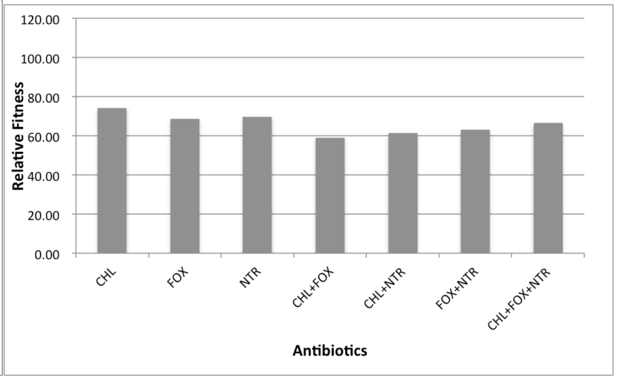
120.00
100.00
80.00
60.00
40.00
20.00
0.00
Relative Fitness
CHL
FOX
NTR
CHL+FOX
CHL+NTR
FOX+NTR
CHL+FOX+NTR
Antibiotics

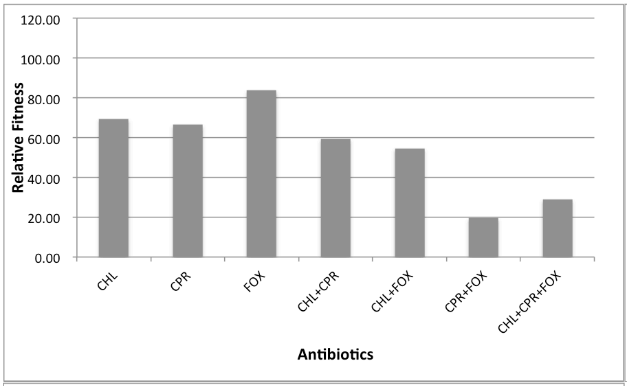
120.00
100.00
80.00
60.00
40.00
20.00
0.00
Relative Fitness
CHL
CPR
FOX
CHL+CPR
CHL+FOX
CPR+FOX
CHL+CPR+FOX
Antibiotics

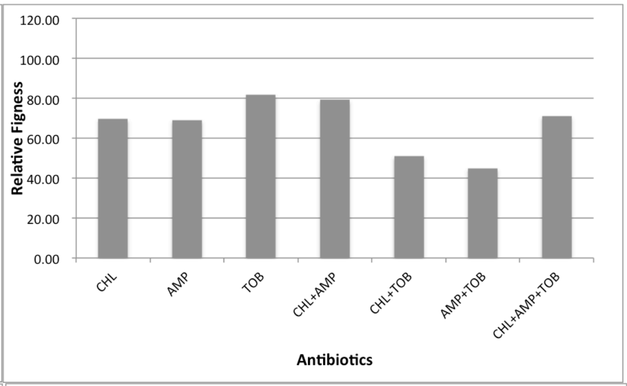
120.00
100.00
80.00
60.00
40.00
20.00
0.00
Relative Figness
CHL
AMP
TOB
CHL+AMP
CHL+TOB
AMP+TOB
CHL+AMP+TOB
Antibiotics

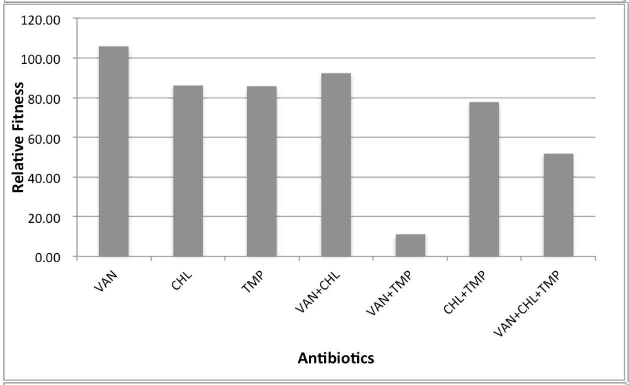
120.00
100.00
80.00
60.00
40.00
20.00
0.00
Relative Fitness
VAN
CHL
TMP
VAN+CHL
VAN+TMP
CHL+TMP
VAN+CHL+TMP
Antibiotics

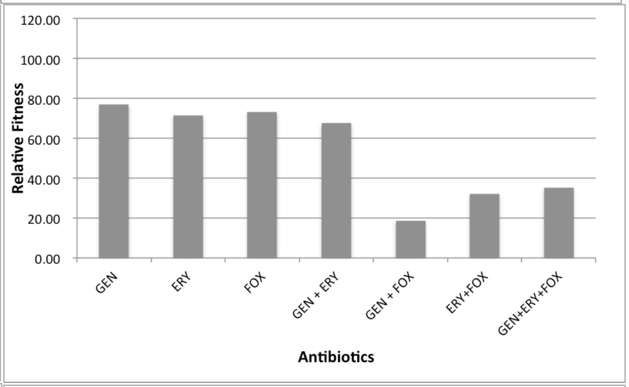
120.00
100.00
80.00
60.00
40.00
20.00
0.00
Relative Fitness
GEN
ERY
FOX
GEN + ERY
GEN + FOX
ERY+FOX
GEN+ERY+FOX
Antibiotics

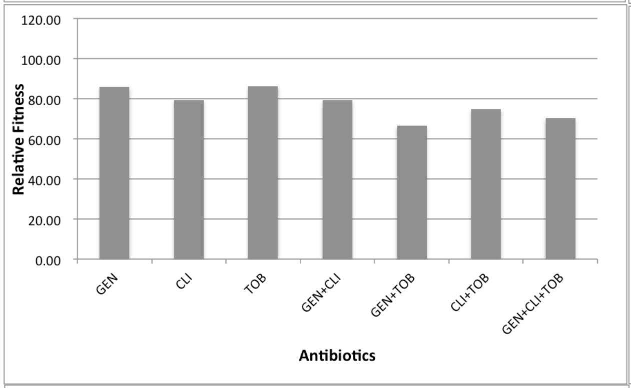
120.00
100.00
80.00
60.00
40.00
20.00
0.00
Relative Fitness
GEN
CLI
TOB
GEN+CLI
GEN+TOB
CLI+TOB
GEN+CLI+TOB
Antibiotics

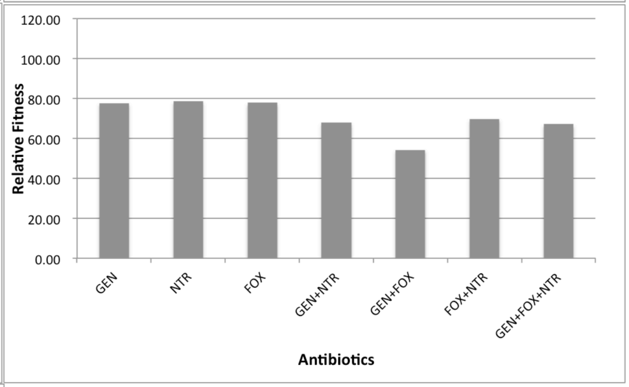
120.00
100.00
80.00
60.00
40.00
20.00
0.00
Relative Fitness
GEN
NTR
FOX
GEN+NTR
GEN+FOX
FOX+NTR
GEN+FOX+NTR
Antibiotics

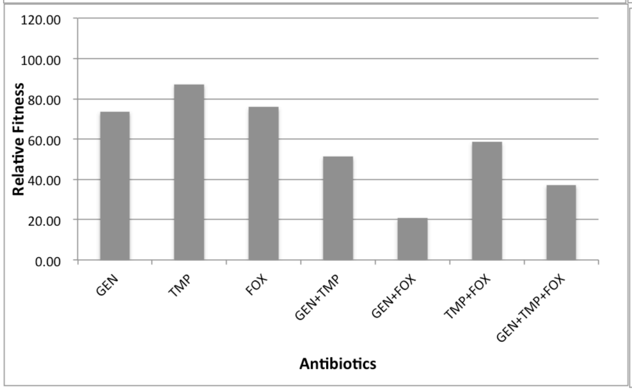
120.00
100.00
80.00
60.00
40.00
20.00
0.00
Relative Fitness
GEN
TMP
FOX
GEN+TMP
GEN+FOX
TMP+FOX
GEN+TMP+FOX
Antibiotics

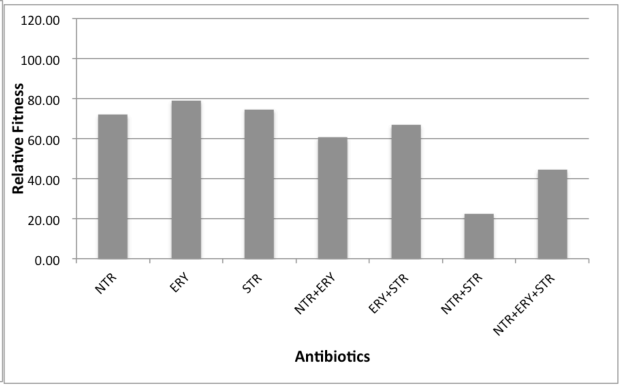
120.00
100.00
80.00
60.00
40.00
20.00
0.00
Relative Fitness
NTR
ERY
STR
NTR+ERY
ERY+STR
NTR+STR
NTR+ERY+STR
Antibiotics

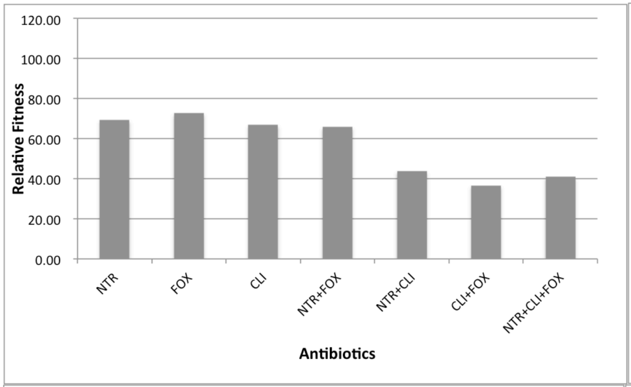
Relative Fitness
120.00
100.00
80.00
60.00
40.00
20.00
0.00
NTR
FOX
CLI
NTR+FOX
NTR+CLI
CLI+FOX
NTR+CLI+FOX
Antibiotics

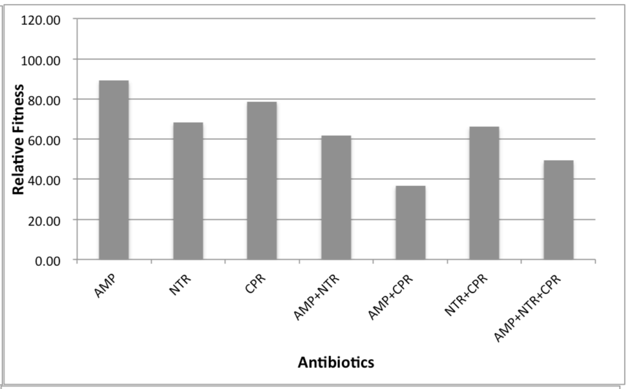
Relative Fitness
120.00
100.00
80.00
60.00
40.00
20.00
0.00
AMP
NTR
CPR
AMP+NTR
AMP+CPR
NTR+CPR
AMP+NTR+CPR
Antibiotics

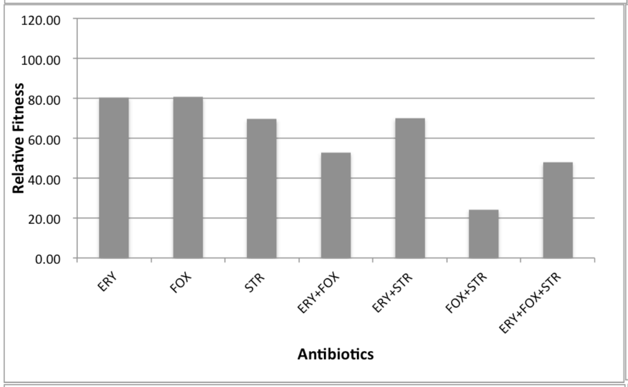
Relative Fitness
120.00
100.00
80.00
60.00
40.00
20.00
0.00
ERY
FOX
STR
ERY+FOX
ERY+STR
FOX+STR
ERY+FOX+STR
Antibiotics

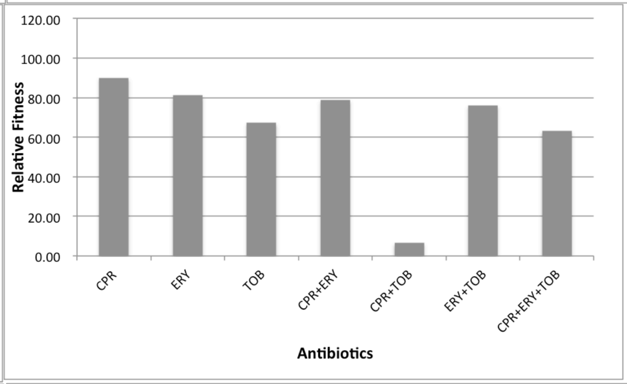
Relative Fitness
120.00
100.00
80.00
60.00
40.00
20.00
0.00
CPR
ERY
TOB
CPR+ERY
CPR+TOB
ERY+TOB
CPR+ERY+TOB
Antibiotics

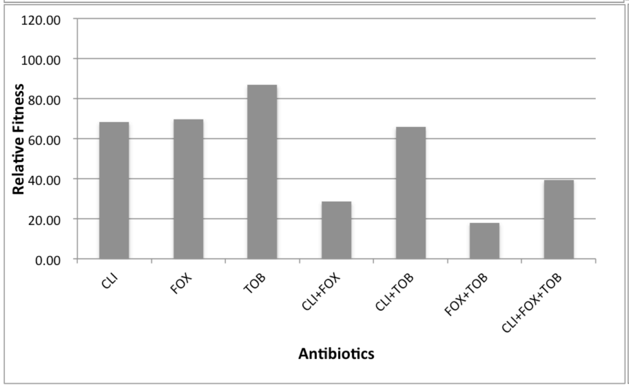
Relative Fitness
120.00
100.00
80.00
60.00
40.00
20.00
0.00
CLI
FOX
TOB
CLI+FOX
CLI+TOB
FOX+TOB
CLI+FOX+TOB
Antibiotics

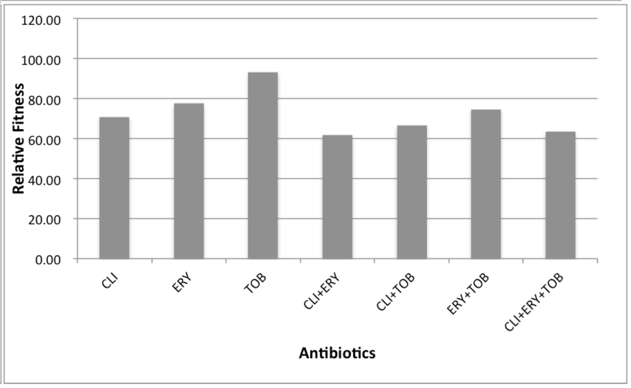
Relative Fitness
120.00
100.00
80.00
60.00
40.00
20.00
0.00
CLI
ERY
TOB
CLI+ERY
CLI+TOB
ERY+TOB
CLI+ERY+TOB
Antibiotics

Supplementary Figure 4. Suppressive three-drug interactions in *S. epidermidis* 14990 and *E. coli* CFT073. Growth measurements are shown for bacteria in single-drug, two-drug, or three-drug conditions relative to the no-drug control (100% growth, not shown). Emergent suppression was determined following Tekin et al. (Tekin et al., 2016) (see Methods). The upper left figure is a schematic with X, Y, and Z representing three different drugs. Only the experimental data for the three-drug combination (X+Y+Z), the suppressed pairwise drug combination with the lower growth (Y+Z), and the suppressor single drug (X) is shown for simplicity. In *S. epidermidis* 14990, 35 triple combinations of a total 313 interaction measures were determined to be suppressive. In *E. coli* CFT073, 88 triple combinations of a total 356 interaction measures were determined to be suppressive. Antibiotic abbreviations are as listed in Table 1.

S. epidermidis 14990

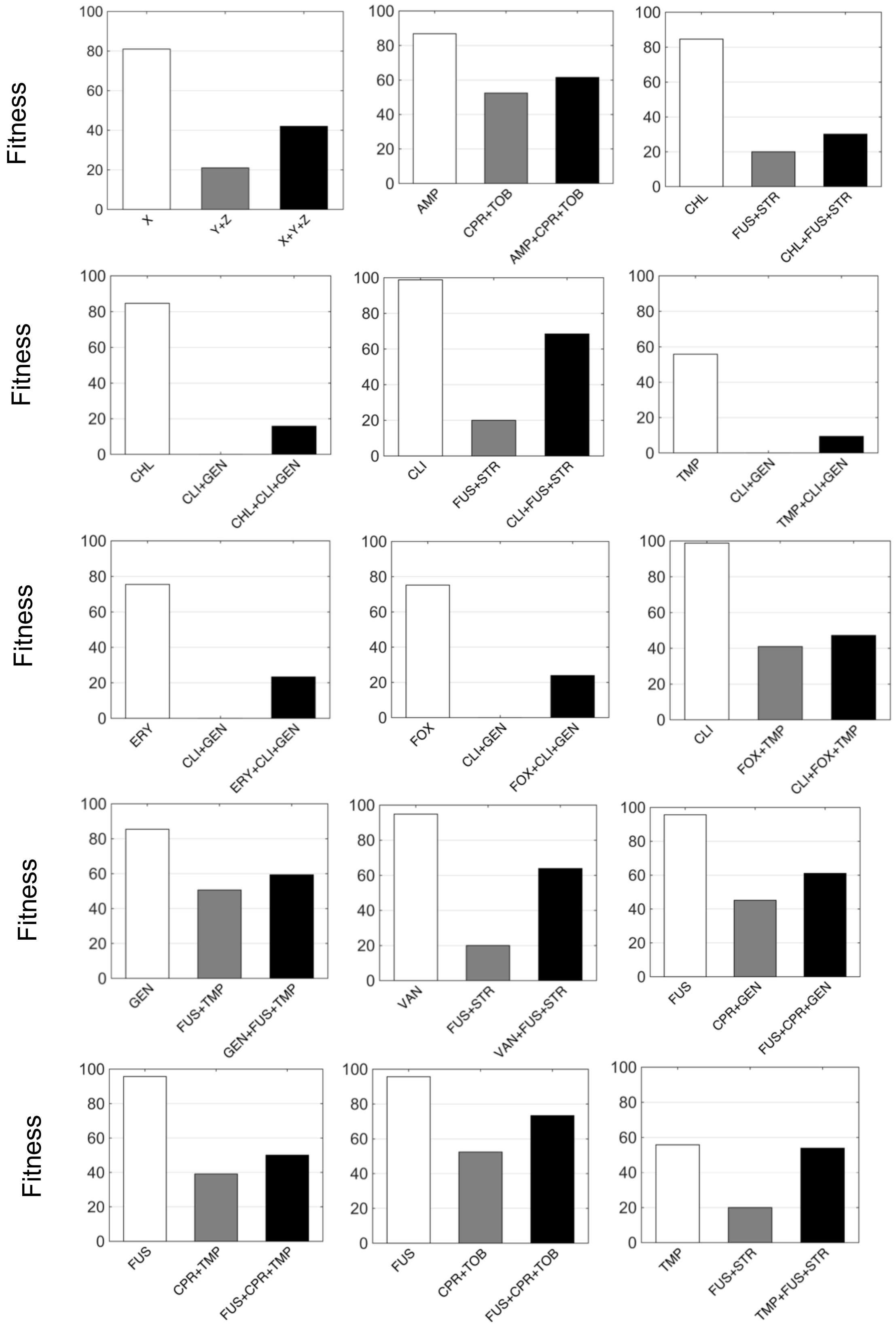

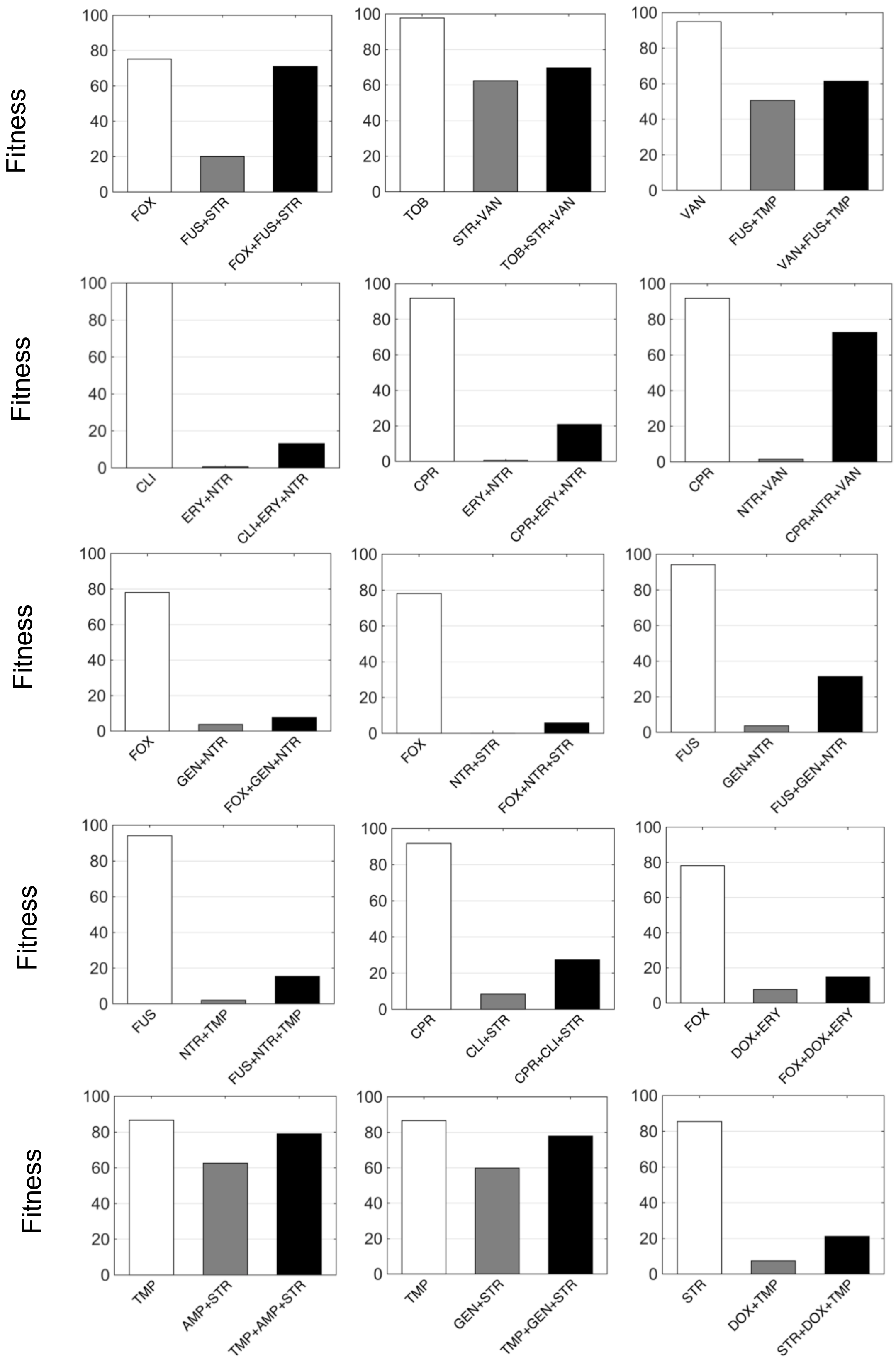

Fitness
Fitness
Fitness
Fitness
Fitness
0
20
40
60
80
100
FOX
FUS+STR
FOX+FUS+STR
TOB
STR+VAN
TOB+STR+VAN
VAN
FUS+TMP
VAN+FUS+TMP
CLI
ERY+NTR
CLI+ERY+NTR
CPR
ERY+NTR
CPR+ERY+NTR
CPR
NTR+VAN
CPR+NTR+VAN
FOX
GEN+NTR
FOX+GEN+NTR
FOX
NTR+STR
FOX+NTR+STR
FUS
GEN+NTR
FUS+GEN+NTR
FUS
NTR+TMP
FUS+NTR+TMP
CPR
CLI+STR
CPR+CLI+STR
FOX
DOX+ERY
FOX+DOX+ERY
TMP
AMP+STR
TMP+AMP+STR
TMP
GEN+STR
TMP+GEN+STR
STR
DOX+TMP
STR+DOX+TMP

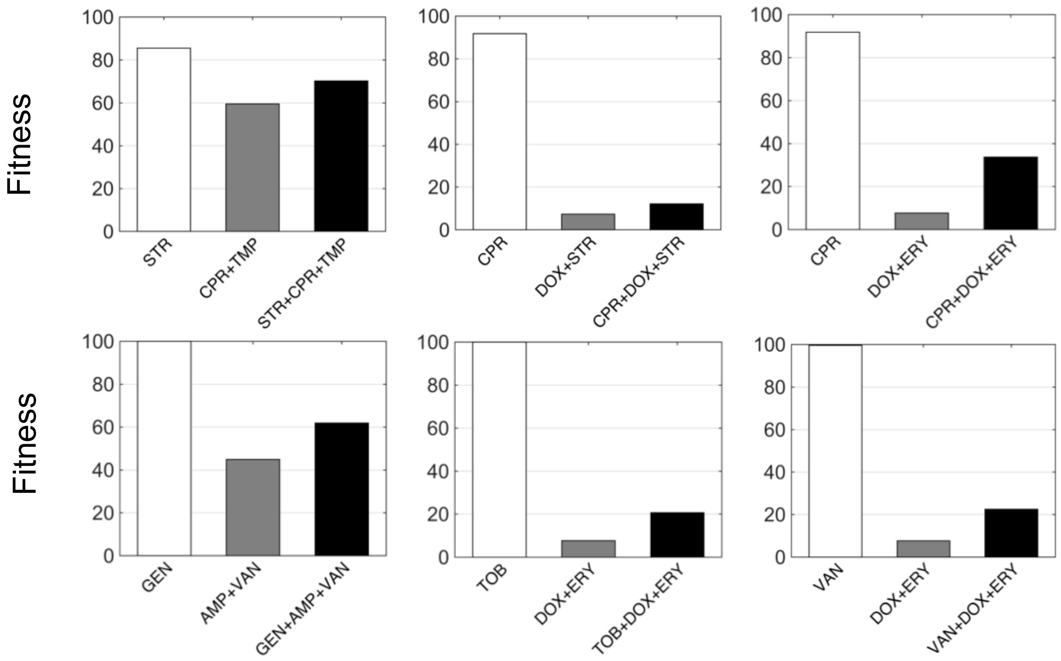

Fitness
Fitness
STR
CPR+TMP
STR+CPR+TMP
CPR
DOX+STR
CPR+DOX+STR
CPR
DOX+ERY
CPR+DOX+ERY
GEN
AMP+VAN
GEN+AMP+VAN
TOB
DOX+ERY
TOB+DOX+ERY
VAN
DOX+ERY
VAN+DOX+ERY

E. coli CTF073

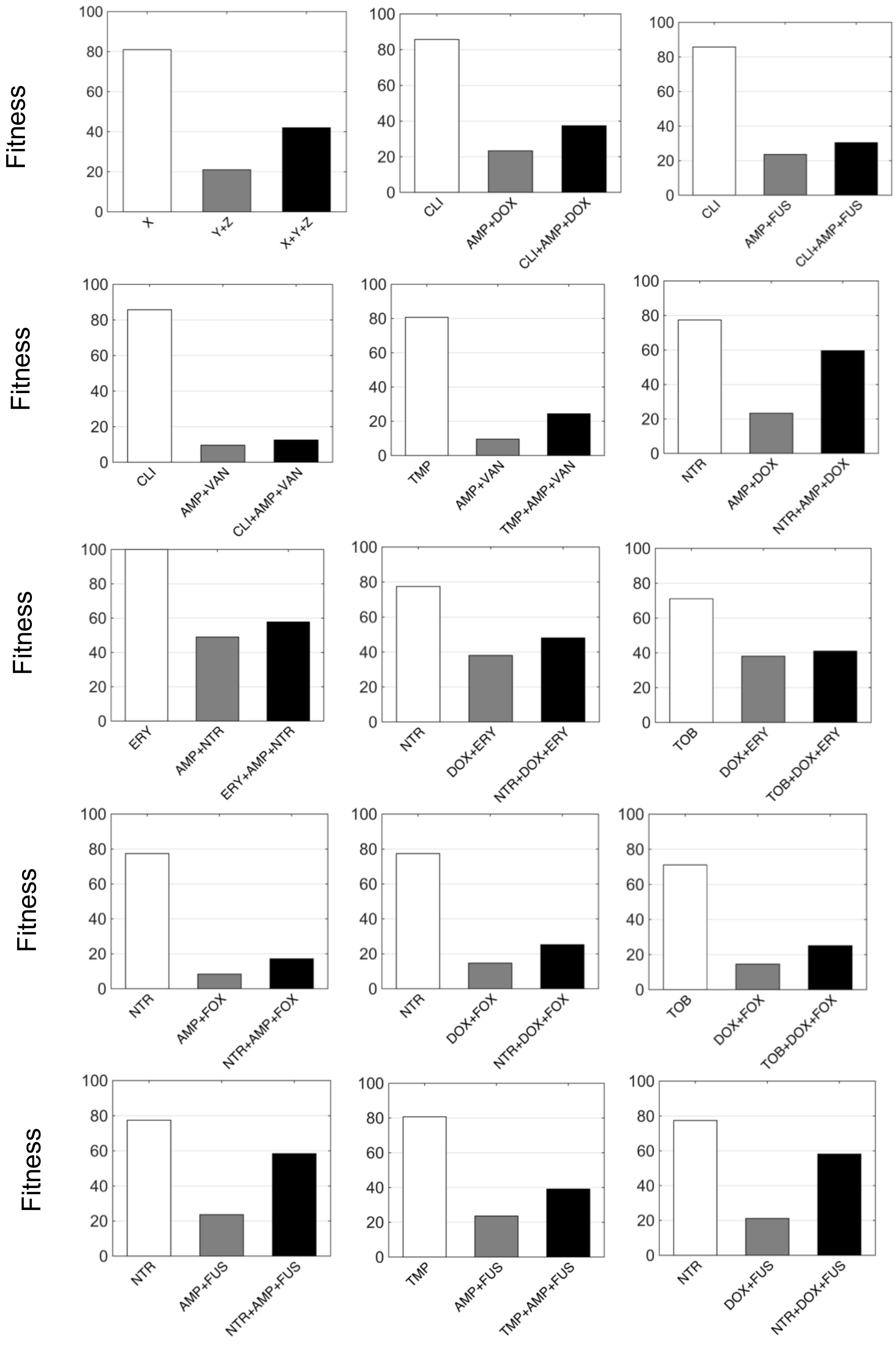

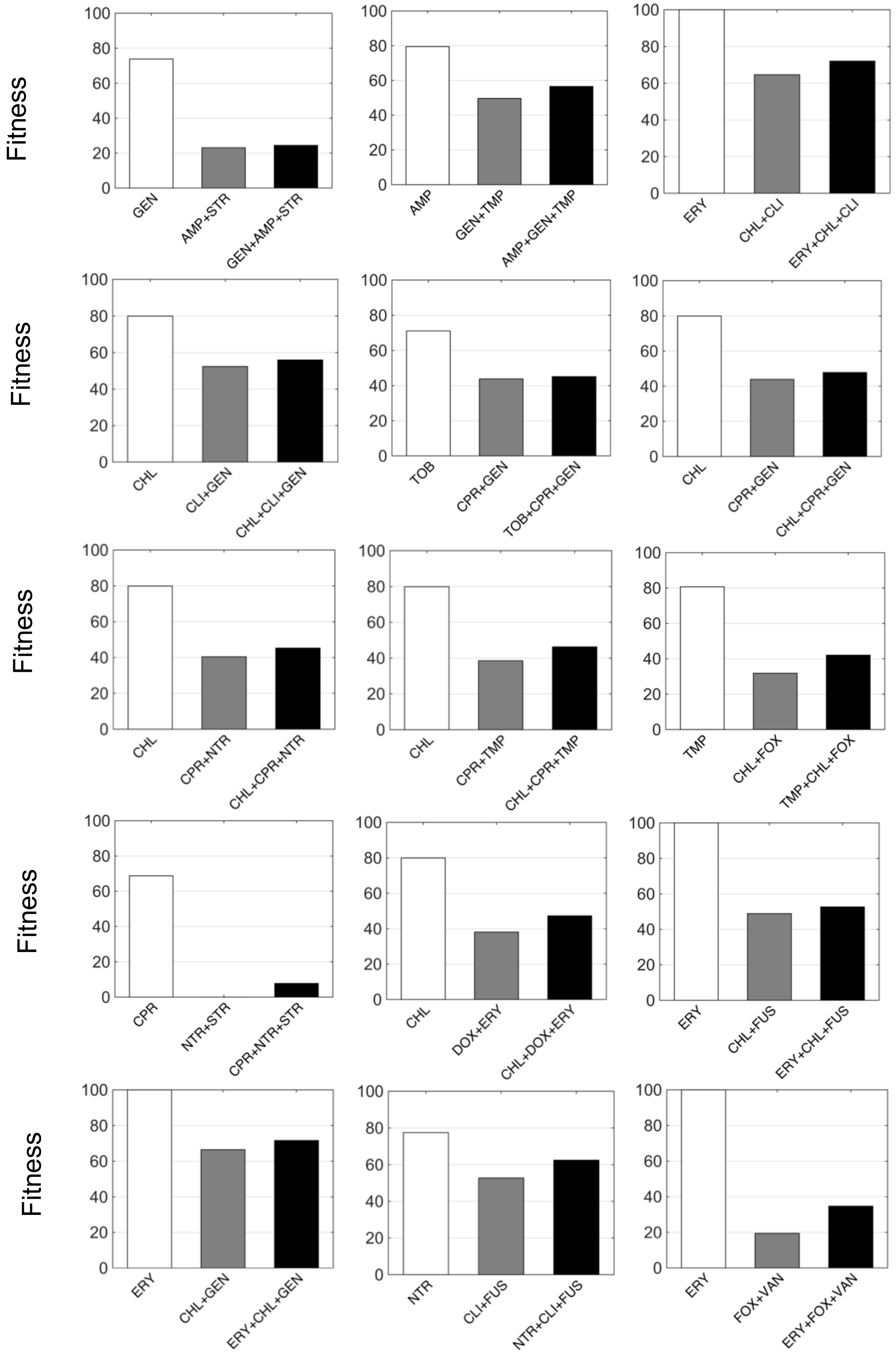

Fitness
GEN
AMP+STR
GEN+AMP+STR
AMP
GEN+TMP
AMP+GEN+TMP
ERY
CHL+CLI
ERY+CHL+CLI
Fitness
CHL
CLI+GEN
CHL+CLI+GEN
TOB
CPR+GEN
TOB+CPR+GEN
CHL
CPR+GEN
CHL+CPR+GEN
Fitness
CHL
CPR+NTR
CHL+CPR+NTR
CHL
CPR+TMP
CHL+CPR+TMP
TMP
CHL+FOX
TMP+CHL+FOX
Fitness
CPR
NTR+STR
CPR+NTR+STR
CHL
DOX+ERY
CHL+DOX+ERY
ERY
CHL+FUS
ERY+CHL+FUS
Fitness
ERY
CHL+GEN
ERY+CHL+GEN
NTR
CLI+FUS
NTR+CLI+FUS
ERY
FOX+VAN
ERY+FOX+VAN

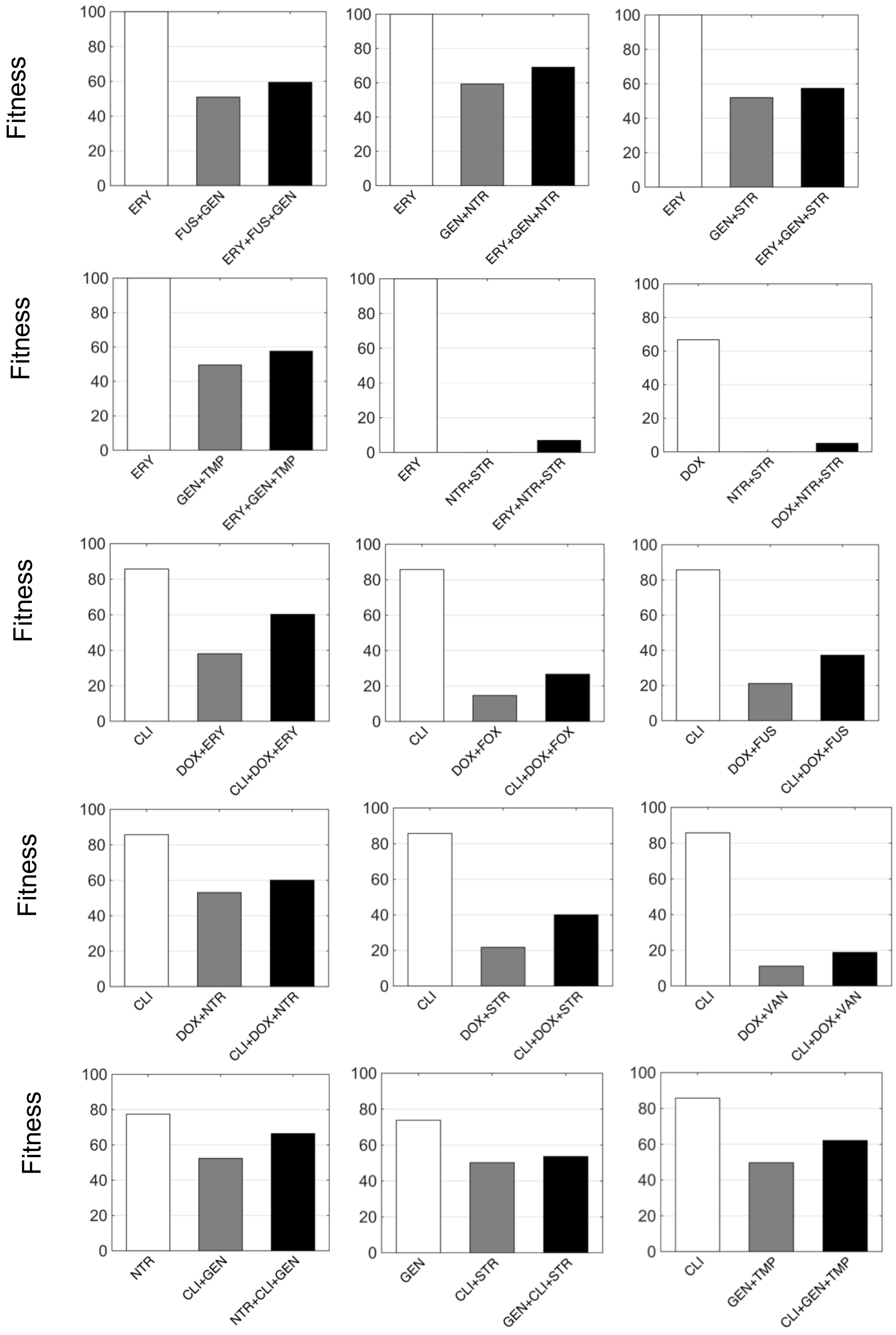
Fitness
Fitness
Fitness
Fitness
Fitness
0
20
40
60
80
100
ERY
FUS+GEN
ERY+FUS+GEN
ERY
GEN+NTR
ERY+GEN+NTR
ERY
GEN+STR
ERY+GEN+STR
ERY
GEN+TMP
ERY+GEN+TMP
ERY
NTR+STR
ERY+NTR+STR
DOX
NTR+STR
DOX+NTR+STR
CLI
DOX+ERY
CLI+DOX+ERY
CLI
DOX+FOX
CLI+DOX+FOX
CLI
DOX+FUS
CLI+DOX+FUS
CLI
DOX+NTR
CLI+DOX+NTR
CLI
DOX+STR
CLI+DOX+STR
CLI
DOX+VAN
CLI+DOX+VAN
NTR
CLI+GEN
NTR+CLI+GEN
GEN
CLI+STR
GEN+CLI+STR
CLI
GEN+TMP
CLI+GEN+TMP

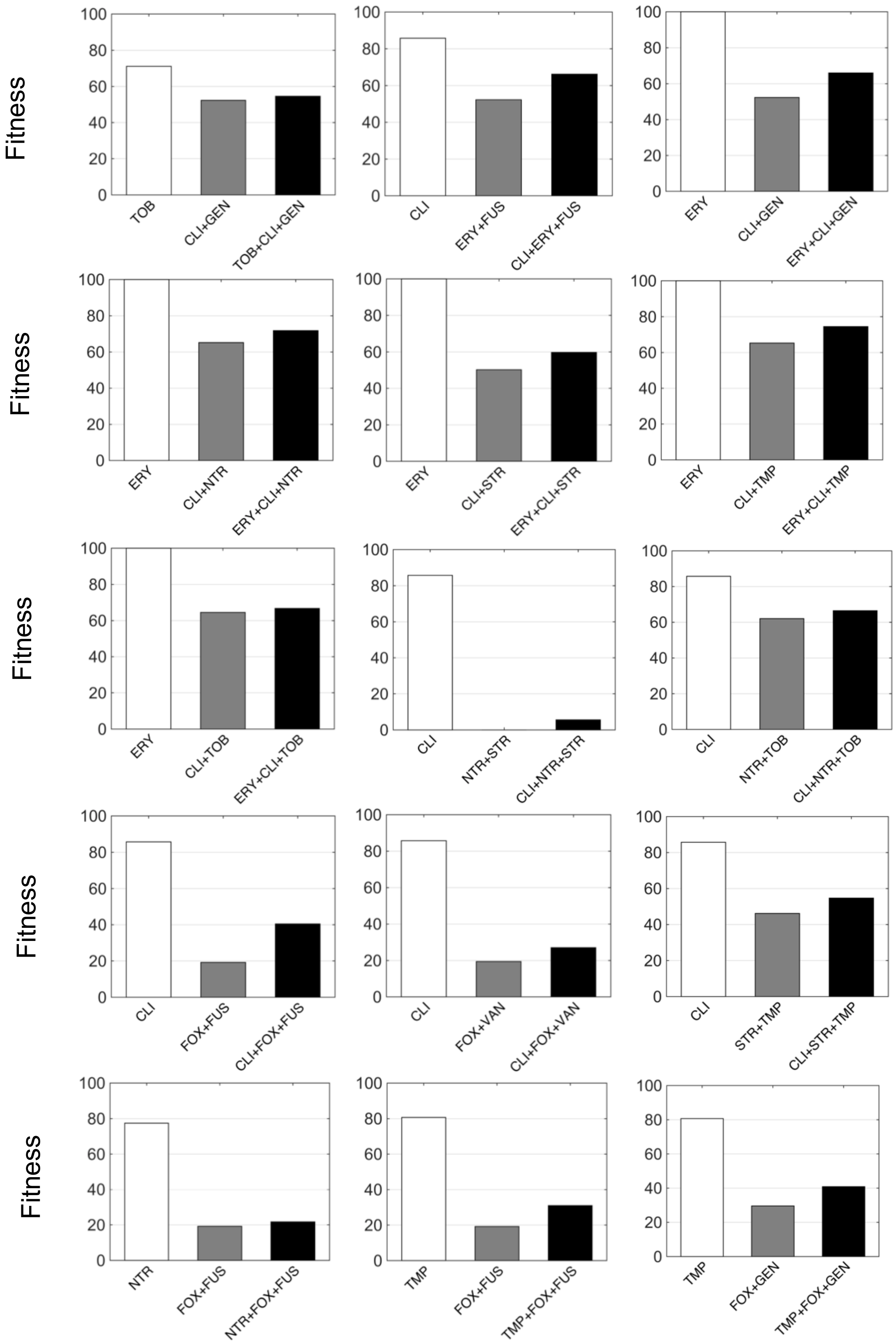
Fitness
Fitness
Fitness
Fitness
Fitness
0
20
40
60
80
100
TOB
CLI+GEN
TOB+CLI+GEN
CLI
ERY+FUS
CLI+ERY+FUS
ERY
CLI+GEN
ERY+CLI+GEN
ERY
CLI+NTR
ERY+CLI+NTR
ERY
CLI+STR
ERY+CLI+STR
ERY
CLI+TMP
ERY+CLI+TMP
ERY
CLI+TOB
ERY+CLI+TOB
CLI
NTR+STR
CLI+NTR+STR
CLI
NTR+TOB
CLI+NTR+TOB
CLI
FOX+FUS
CLI+FOX+FUS
CLI
FOX+VAN
CLI+FOX+VAN
CLI
STR+TMP
CLI+STR+TMP
NTR
FOX+FUS
NTR+FOX+FUS
TMP
FOX+FUS
TMP+FOX+FUS
TMP
FOX+GEN
TMP+FOX+GEN

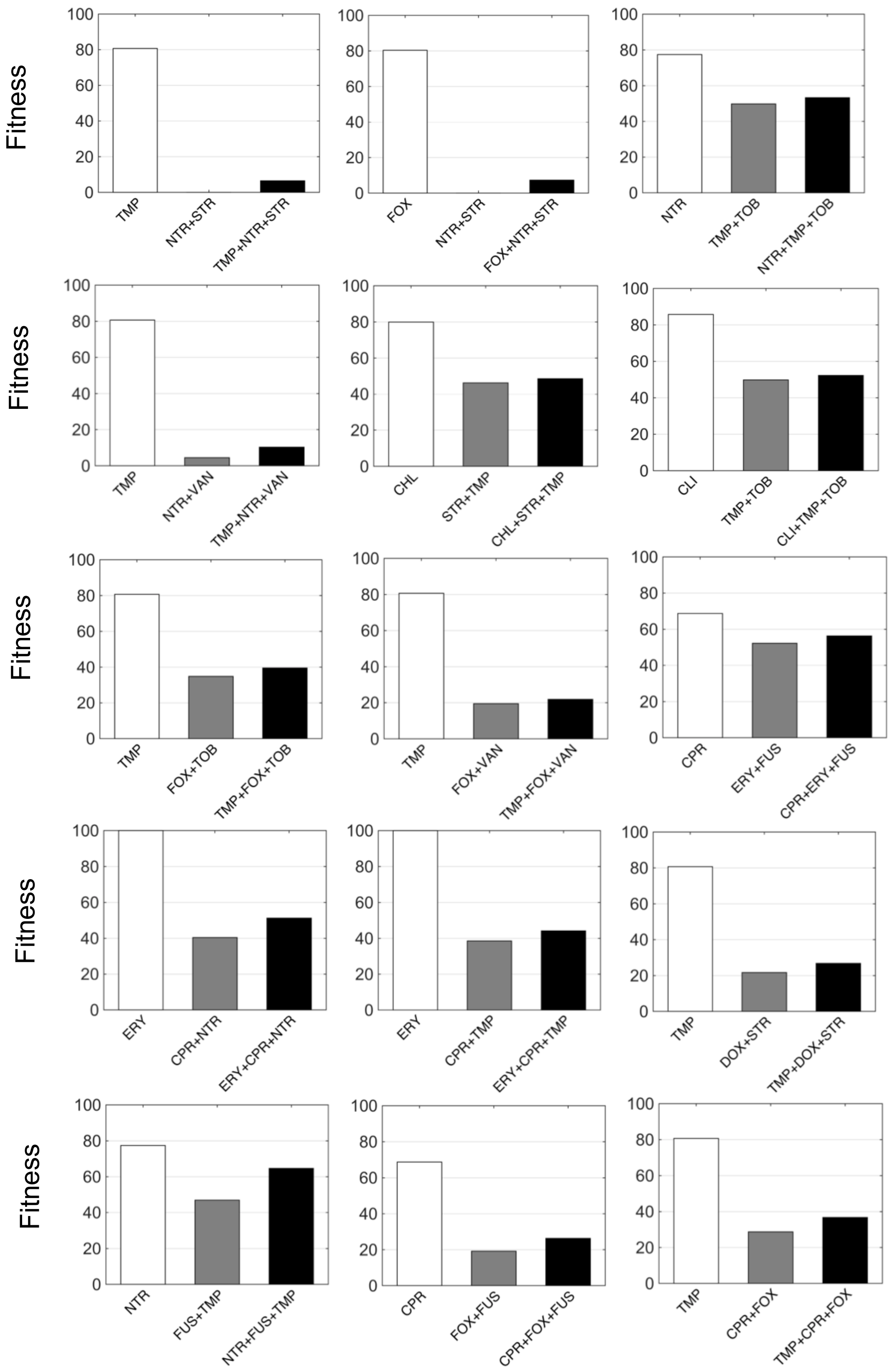
Fitness
100
80
60
40
20
0
TMP
NTR+STR
TMP+NTR+STR
FOX
NTR+STR
FOX+NTR+STR
NTR
TMP+TOB
NTR+TMP+TOB
TMP
NTR+VAN
TMP+NTR+VAN
CHL
STR+TMP
CHL+STR+TMP
CLI
TMP+TOB
CLI+TMP+TOB
TMP
FOX+TOB
TMP+FOX+TOB
TMP
FOX+VAN
TMP+FOX+VAN
CPR
ERY+FUS
CPR+ERY+FUS
ERY
CPR+NTR
ERY+CPR+NTR
ERY
CPR+TMP
ERY+CPR+TMP
TMP
DOX+STR
TMP+DOX+STR
NTR
FUS+TMP
NTR+FUS+TMP
CPR
FOX+FUS
CPR+FOX+FUS
TMP
CPR+FOX
TMP+CPR+FOX

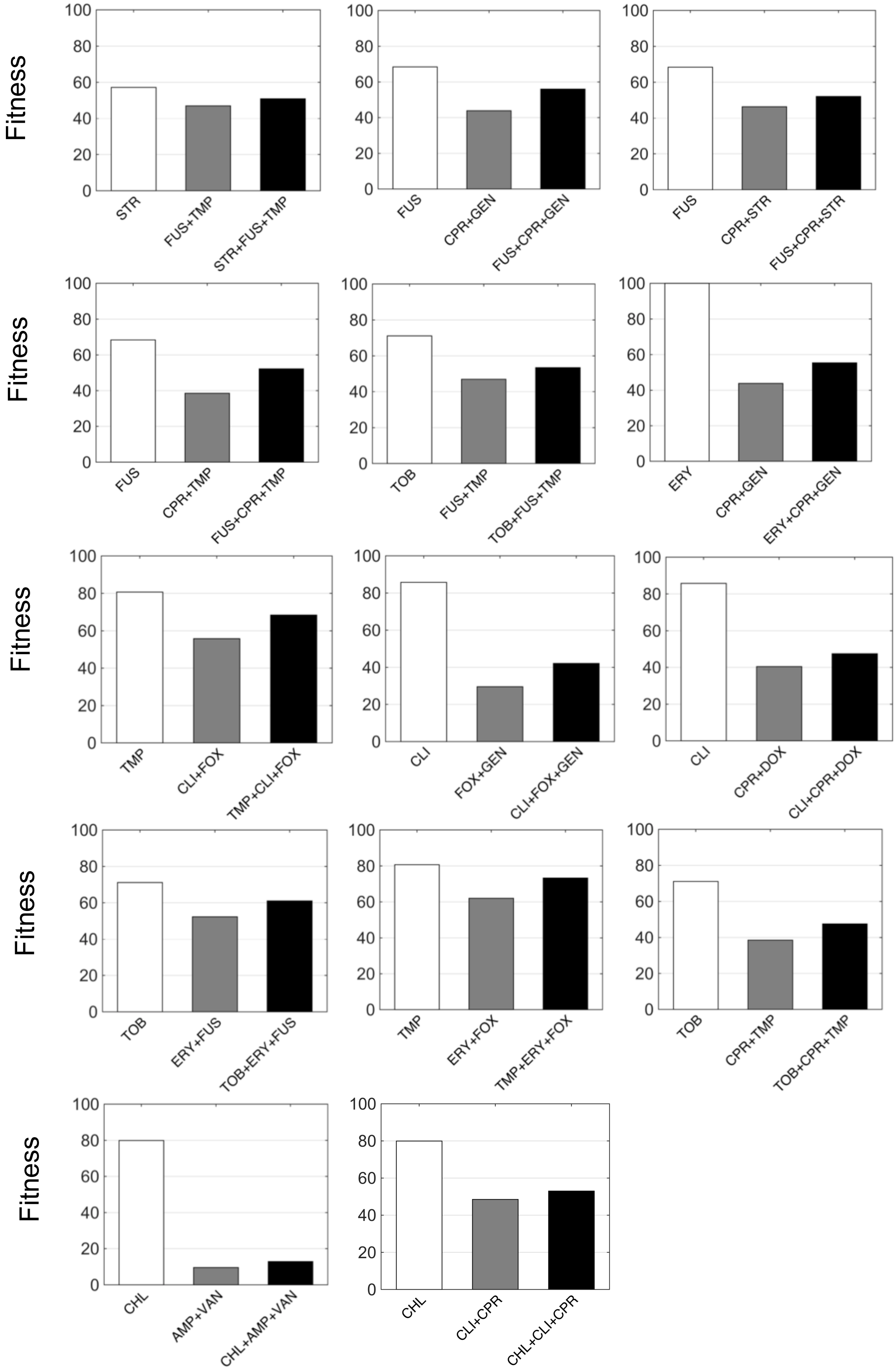
Fitness
0
20
40
60
80
100
STR
FUS+TMP
STR+FUS+TMP
FUS
CPR+GEN
FUS+CPR+GEN
FUS
CPR+STR
FUS+CPR+STR
FUS
CPR+TMP
FUS+CPR+TMP
TOB
FUS+TMP
TOB+FUS+TMP
ERY
CPR+GEN
ERY+CPR+GEN
TMP
CLI+FOX
TMP+CLI+FOX
CLI
FOX+GEN
CLI+FOX+GEN
CLI
CPR+DOX
CLI+CPR+DOX
TOB
ERY+FUS
TOB+ERY+FUS
TMP
ERY+FOX
TMP+ERY+FOX
TOB
CPR+TMP
TOB+CPR+TMP
CHL
AMP+VAN
CHL+AMP+VAN
CHL
CLI+CPR
CHL+CLI+CPR

Supplementary Figure 5. Suppressor and suppressee antibiotics for *S. epidermidis* 14990 and *E. coli* CFT07. For each antibiotic, the number of suppressive interactions in *S. epidermidis* 14990 (above, R=-0.115, N=14, one-tailed p-value=0.347) and *E. coli* CFT07 (below, R=-0.275, N=14, one-tailed p-value=0.171) in which it acts as the suppressor (x axis) and the suppressee (y axis) are plotted. Antibiotic abbreviations are as listed in Table 1.

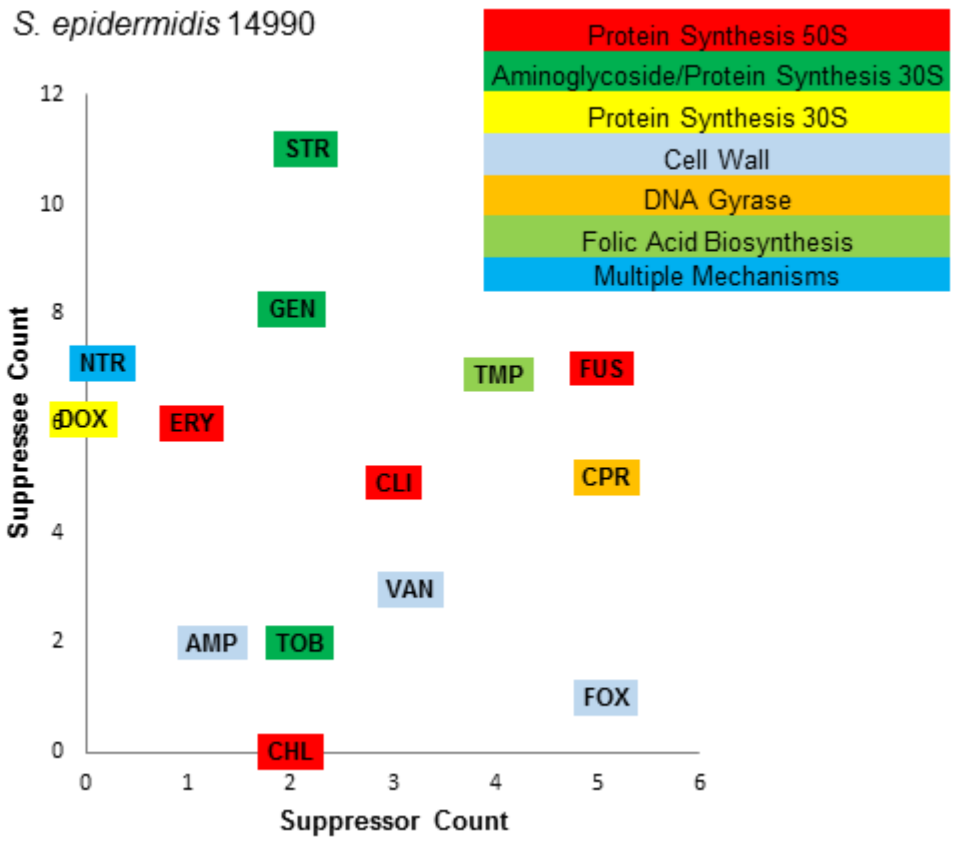

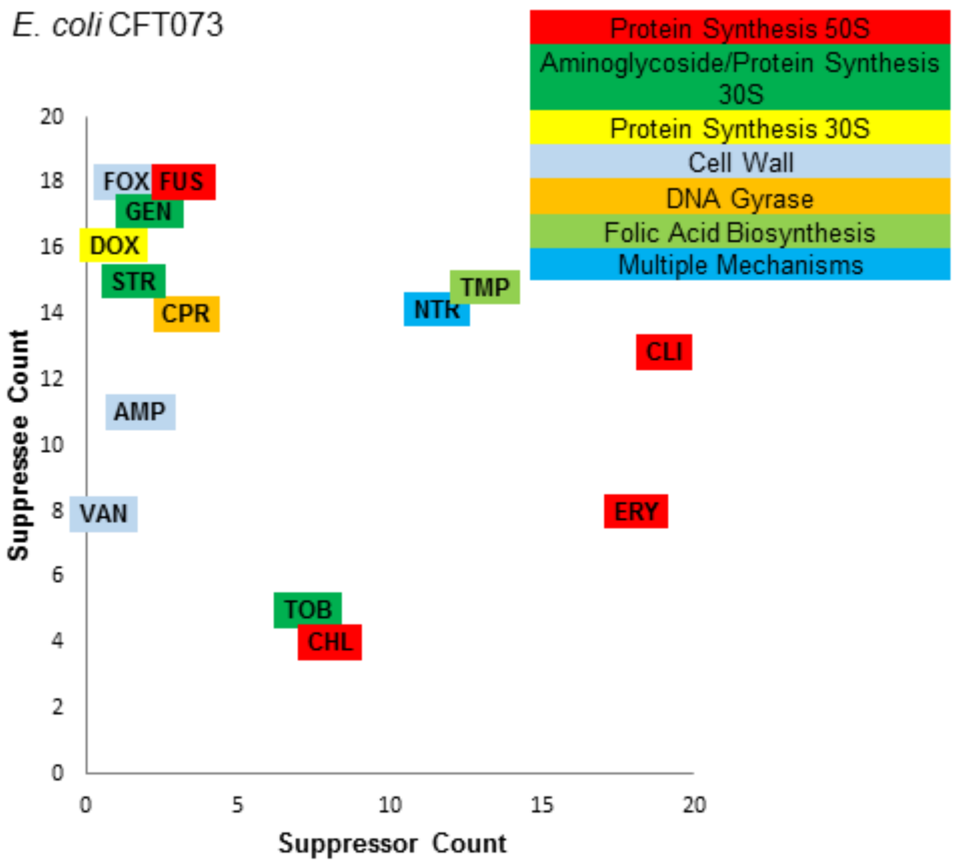

E. coli CFT073
Protein Synthesis 50S
Aminoglycoside/Protein Synthesis 30S
Protein Synthesis 30S
Cell Wall
DNA Gyrase
Folic Acid Biosynthesis
Multiple Mechanisms
FOX
FUS
GEN
DOX
STR
CPR
NTR
TMP
CLI
AMP
VAN
ERY
TOB
CHL
Suppressee Count
Suppressor Count
0
2
4
6
8
10
12
14
16
18
20
0
5
10
15
20

Supplementary Table 1. Full data set for 14 drugs in *E. coli* BW25113, *S. epidermidis* 14990, and *E. coli* CFT073. Growth percentages (median, maximum, and minimum) as compared to the no-drug control (100% growth) are given for the three single-drug, the three two-drug, and the triple combination. For *E. coli* BW25113 concentrations are listed in μg/mL and for *S. epidermidis* 14990 and *E. coli* CFT073 concentrations are listed in μM. For *E. coli* BW25113, n represents the number of independent experiments, each with 4-6 samples per experiment. For *E. coli* CFT073 and *S. epidermidis* 14990, all combinations were tested in exactly 2 independent experiments, and n represents the number of total samples over 2 experiments. Abbreviations are as listed in Table 1.

E. coli **BW25113**							
Combinations	X	Y	Z	XY	XZ	YZ	XYZ
CLI 31.5+CPR 13+ERY 14							
Median (n=2)	68.9	87.85	77.65	49.95	61.2	75.85	19.6
Max	72.5	88.4	77.8	76.3	62.8	76.3	27
Min	65.3	87.3	77.5	23.6	59.6	75.4	12.2
CLI 31.5+CPR 13+FOX 1.16							
Median (n=3)	65.3	88.4	57.4	29	18.2	37.6	14.6
Max	71.5	91.8	93.6	76.3	38.9	60.8	17.3
Min	64.3	63.8	56.3	26	14.1	16.3	9.7
CPR 0.01+CLI 31.5+STR 4.5							
Median (n=5)	89	74.4	73.3	47.5	16.1	64	0.1
Max	92.1	77.8	84.2	64.6	60.2	66.5	0.3
Min	55.1	70.6	58.8	13.5	0.3	55.5	0
CPR 0.01+CLI 31.5+TOB 1.3							
Median (n=5)	91.1	76.9	68	63.4	5.7	69.7	11.8
Max	92.4	78.9	73.5	64.6	22.9	76.6	48.4
Min	55.1	70.6	59.7	13.5	0.3	65.7	0.1

CLI 31.5+ERY 14+FOX 1.16							
Median (n=4)	64.5	79.65	83.15	64.35	30.95	54.95	24.15
Max	82.5	82	94.6	73.5	38.3	65.1	35.3
Min	59	73.3	70.9	60.7	18.9	37	20.4
CLI 31.5+ERY 14+STR 4.5							
Median (n=2)	68.9	77.65	55.15	61.2	58.45	66.45	54.45
Max	72.5	77.8	55.2	62.8	61.8	72.4	57.7
Min	65.3	77.5	55.1	59.6	55.1	60.5	51.2
CLI 31.5+ERY 14+TOB 1.3							
Median (n=4)	70.65	77.65	93.35	61.8	66.55	74.65	63.35
Max	74.5	83.1	94.5	70.5	73.8	81.7	70.1
Min	65.3	77.3	80.9	59.6	66.1	73.6	60
CLI 31.5+FOX 1.16+STR 4.5							
Median (n=6)	70.95	80.7	69.6	39.15	58.2	20.45	37.5
Max	76.9	93.6	92.8	44.5	66.5	29	41.3
Min	61.5	57.4	51.4	14.1	47.6	0.7	0.9
CLI 31.5+FOX 1.16+TOB 1.3							
Median (n=4)	68.4	69.85	86.9	28.55	65.95	18.05	39.5
Max	74.5	93.6	93.7	48.7	73.8	46.9	55.6

Min	64.3	56.3	67.3	14.1	46	14	18.7
CLI 31.5+STR 4.5+TOB 1.3							
Median (n=7)	73.1	75.2	68.9	61.3	72.1	0.7	0.8
Max	76.9	92.8	93.7	66.5	75	2.5	13
Min	61.5	39.9	65	48.9	61.5	0	0.1
CPR 0.01+ERY 14+FOX 1.16							
Median (n=2)	92.05	80.35	81.1	81.95	53.9	46.7	37.95
Max	92.4	82.2	82.6	84.3	55.7	52.2	42.2
Min	91.7	78.5	79.6	79.6	52.1	41.2	33.7
CPR 0.01+ERY 14+STR 4.5							
Median (n=4)	90.05	82.05	74.6	78.7	28.9	70.75	62.4
Max	92.1	85.5	84.2	79.9	60.2	76.9	77.8
Min	88	79.6	65.9	74.5	15.3	68.7	55.5
CPR 0.01+ERY 14+TOB 1.3							
Median (n=4)	90.05	81.1	67.2	78.95	6.6	76.1	63.3
Max	92.4	85.5	73.5	79.9	22.9	77.5	67.6
Min	88	78.5	65	74.5	0.3	71.8	60.2
CPR 0.01+FOX 1.16+STR 4.5							
Median (n=3)	91.1	79	73.3	62.3	41.7	25.3	28.6

Max	92.1	82.4	84.2	83.2	60.2	29	36.9
Min	88	78.9	65.9	61.6	16.1	23.2	10.8
CPR 0.01+FOX 1.16+TOB 1.3							
Median (n=3)	91.1	82.4	68.9	62.3	5.7	44.8	20.1
Max	92.4	82.6	73.5	83.2	22.9	46.8	39.7
Min	88	79	65	55.7	0.3	36.6	0.4
CPR 0.01+STR 4.5+TOB 1.3							
Median (n=3)	89	73.3	65.5	16.1	7.5	0	0
Max	91.1	75.9	68.9	41.7	22.9	2.5	0
Min	88	65.9	65	15.3	5.7	0	0
ERY 14+FOX 1.16+STR 4.5							
Median (n=4)	80.55	80.7	69.6	52.85	70.1	24.25	47.95
Max	85.5	93.6	84.2	54.4	76.9	29	51.4
Min	77.5	78.9	55.1	33.7	60.5	7.9	41.1
ERY 14+FOX 1.16+TOB 1.3							
Median (n=2)	79.65	90.3	96.3	59.95	78.3	17.15	56.8
Max	82	94.6	99.6	65.1	81.4	17.6	62.8
Min	77.3	86	93	54.8	75.2	16.7	50.8
ERY 14+STR 4.5+TOB 1.3							

Median (n=9)	82.3	75.2	68.9	71.5	75.6	0.8	1.5
Max	87.9	92.8	93.7	76.9	81.7	78.9	65.4
Min	75.6	39.9	59.7	33.2	69.9	0	0.3
FOX 1.16+STR 4.5+TOB 1.3							
Median (n=6)	80.25	74.25	85.05	12.8	39.45	0.65	0.3
Max	93.6	92.8	93.7	29	57.1	50.8	11.4
Min	57.4	51.4	65	0.4	14	0	0
AMP 1.0+CPR 10+CLI 17.5							
Median (n=7)	79.14	68.46	69.81	40.38	45.34	37.61	16.71
Max	88.16	78.77	80.74	53.99	59.79	49.41	28.5
Min	70.43	63.98	63.82	20.97	26.86	0.83	6.75
AMP 0.9+CPR 10+ERY 18							
Median (n=2)	77.45	77.595	82.485	43.405	80.23	69.24	55.545
Max	87.31	84.65	83.57	45.61	82.76	75.1	60.21
Min	67.59	70.54	81.4	41.2	77.7	63.38	50.88
AMP 0.9+CPR 10+FOX 1							
Median (n=4)	77.595	78.93	65.39	51.155	33.71	30.96	10.37
Max	84.39	79.76	84.73	67.75	45.65	44.74	24.68
Min	70.43	69.94	58.81	34.48	18.96	21.12	7.89

AMP 1.0+CPR 10+NTR 1							
Median (n=4)	89.11	68.195	78.715	61.67	36.79	66.12	49.27
Max	89.98	79.19	84.25	93.41	58.17	74.92	61.61
Min	84.43	57.61	50.51	47.47	35.22	56.34	37.35
AMP 0.9+CPR 10+STR 2.3							
Median (n=5)	87.52	78.7	77.73	43.25	42.2	45.36	1.44
Max	91.62	91.4	86.99	65.59	77.4	74.82	23.06
Min	65.04	72.26	61.37	34.9	23.49	19.52	1.04
AMP 0.9+CPR 10+TOB 0.8							
Median (n=3)	88.27	87.43	73.07	46.27	48.86	46.05	0.98
Max	88.85	88.53	76.03	50.88	52.29	68.52	11.37
Min	82.57	72.26	72.42	39.97	48.78	1.08	0.88
AMP 1.0+STR 2.3+CLI 17.5							
Median (n=3)	75.08	73.71	69.81	60.1	38.01	59.59	23.93
Max	88.16	79.72	72.36	61.77	45.34	65.07	29.51
Min	70.43	67.41	63.82	46.93	26.86	58.45	8.61
AMP 1.0+STR 2.3+ERY 18							
Median (n=3)	80.82	79.7	76.91	49.74	87.8	76.6	58.98
Max	88.2	80.54	87.33	60.1	89.6	90.22	62.2

Min	75.08	67.41	76.9	46.93	62.75	70.7	47.78
AMP 0.9+STR 2.3+FOX 1							
Median (n=4)	76.075	74.28	76.165	45.09	33.13	33.795	15.435
Max	87.31	88.78	84.73	70.15	45.65	69.87	26.23
Min	67.59	73.71	65.59	19.75	24.34	22.46	4.52
AMP 1.1+STR 2.3+NTR 1							
Median (n=4)	88.5	76.485	84.29	61.67	52.295	30.63	6.73
Max	92.54	86.16	86.58	71.52	66.11	61.39	7.09
Min	84.43	64.41	74.72	48.89	46.78	16.45	0.82
AMP 0.9+STR 2.3+TOB 0.8							
Median (n=3)	88.85	77.54	74.31	42.57	51.47	12.71	0.46
Max	90.4	77.73	76.03	43.01	52.29	18.8	0.97
Min	82.57	76.25	72.42	25.13	48.86	7.72	0.41
AMP 1.0+ERY 18+CLI 17.5							
Median (n=4)	79.98	75.34	69.165	63.835	52.975	75.09	48.815
Max	86.93	87.33	80.28	87.8	59.79	84.72	55.67
Min	75.08	68.55	63.82	52.46	45.34	68.28	33.42
AMP 1.0+ERY 18+FOX 1							
Median (n=2)	86.535	66.11	67.455	67.04	31.44	41.57	21.61

Max	86.93	73.77	71.25	69.16	35.8	42.43	25.25
Min	86.14	58.45	63.66	64.92	27.08	40.71	17.97
AMP 1.1+ERY 18+NTR 1							
Median (n=5)	89.78	79.19	76.04	66.88	69.64	57.99	53.01
Max	92.54	86.16	81.69	93.41	76.34	67.92	62.68
Min	84.43	64.41	61.76	48.89	48.01	56.99	34.96
AMP 1.0+ERY 18+TOB 0.8							
Median (n=2)	77.95	82.12	63.3	75.275	20.99	76.11	55.835
Max	80.82	87.33	63.76	87.8	40.98	86.63	66.88
Min	75.08	76.91	62.84	62.75	1	65.59	44.79
AMP 1.0+NTR 1+CLI 17.5							
Median (n=3)	86.46	75.51	74.29	42.85	60.94	55.58	40.49
Max	89.98	79.19	77.25	93.41	62.13	65.63	52.99
Min	86.26	64.23	62.15	36.02	58.5	55.5	34.96
AMP 1.1+NTR 1+FOX 0.9							
Median (n=3)	86.46	69.32	66.82	62.13	38.31	66.89	36.91
Max	88.77	74.36	89.07	81.59	40.48	68.01	39.77
Min	86.02	62.15	53.55	60.76	34.87	65.83	29.5
AMP 1.0+NTR 1+TOB 0.8							

Median (n=3)	89.98	79.19	59.61	66.88	19.95	1	1.15
Max	92.54	80.99	87.32	93.41	27.32	1.53	1.67
Min	84.43	64.41	55.39	48.89	1.28	0.98	1
AMP 1.1+CLI 17.5+FOX 0.9							
Median (n=2)	86.36	69.87	65.115	39.435	38.05	32.945	19.97
Max	86.46	75.51	76.68	42.85	38.31	34.87	20.58
Min	86.26	64.23	53.55	36.02	37.79	31.02	19.36
AMP 1.0+CLI 17.5+TOB 0.8							
Median (n=5)	80.82	69.04	63.76	45.34	13.28	68.82	11.9
Max	86.93	80.58	79.11	59.79	53.57	76.62	41.13
Min	75.08	63.82	55.19	32.02	0.95	49.36	1.54
AMP 1.0+FOX 1+TOB 0.8							
Median (n=3)	86.14	71.25	72.5	27.08	13.28	38.2	0.71
Max	86.93	80.11	73.4	35.8	64.2	52.23	0.91
Min	75.47	63.66	55.19	27.05	0.95	32.03	0.55
NTR 1+CPR 10+CLI 17.5							
Median (n=3)	71.22	75.23	74.35	65.24	66.03	38.25	46.62
Max	74.24	76.28	74.62	84.28	66.83	40.73	46.91
Min	65.43	71.66	72.6	53.14	63.54	36.67	39.32

NTR 1+CPR 10+ERY 18							
Median (n=4)	80.08	68.165	79.69	63.265	65.53	62.395	57.71
Max	91.5	80.06	89.1	87.57	77.9	81.44	72.43
Min	71.98	59.86	61.76	56.34	57.99	43.91	50.32
NTR 1+CPR 10+FOX 0.9							
Median (n=4)	67.465	69.02	75.28	54.395	65.995	14.81	59.95
Max	83.55	87.08	77.8	89.35	81.75	59.49	61.7
Min	54.68	61.23	63.46	50.83	35.14	8.9	56.08
NTR 1+CPR 10+STR 2.3							
Median (n=5)	74	62.27	79.45	70.41	22.46	1.24	0.76
Max	91.5	80.06	89.14	87.57	50.41	27.19	9.58
Min	71.98	59.86	73.19	56.34	5.24	0.02	0.15
NTR 1+CPR 9+TOB 0.6							
Median (n=4)	78.485	68.135	74.975	68.115	8.18	1.28	2.82
Max	83.52	75.3	81.25	92.19	25.69	12.42	8.6
Min	60.7	62.27	65.42	58.61	0.09	0.23	0.12
NTR 1+CLI 17.5+ERY 18							
Median (n=3)	71.22	71.66	88.79	53.14	67.31	71.27	53.22
Max	71.3	75.22	92.45	65.23	67.48	81.03	59.66

Min	65.43	66.01	69.03	52.88	49.92	62.62	40.82
NTR 1+CLI 17.5+FOX 0.9							
Median (n=3)	69.32	72.8	66.91	65.83	43.85	36.65	40.9
Max	83.55	77.8	68.67	81.75	57.5	41.34	42.54
Min	65.94	66.82	37.33	35.14	38.25	31.82	39.92
NTR 1+CLI 17.5+STR 2.3							
Median (n=4)	68.325	68.835	73.2	53.01	34.155	55.05	6
Max	71.3	75.22	84.75	65.23	39.09	70.33	10.67
Min	61.25	65.14	62.96	52.81	4.57	44.37	0.88
NTR 1+CLI 17.5+TOB 0.6							
Median (n=3)	82.97	75.79	79.15	58.11	25.69	60.9	36.09
Max	83.52	78.09	81.25	64.87	31.6	67.18	45.96
Min	60.7	71.06	65.92	45.41	16.18	41.51	30.73
NTR 1+ERY 18+FOX 0.9							
Median (n=6)	72.19	72.42	76.815	69.59	57.68	47.55	48.325
Max	92.9	95.65	90.13	87.56	88.84	67.23	59.37
Min	59.63	51.89	67.6	0.5	45.18	37.88	28.01
NTR 1+ERY 18+STR 2.3							
Median (n=3)	71.98	79.02	74.72	60.65	66.84	22.43	44.44

Max	74	81.69	79.45	63.27	69.54	38.8	45.64
Min	64.12	77.69	69.13	57.99	66.75	5.7	0.97
NTR 1+ERY 18+TOB 0.8							
Median (n=5)	74	80.42	71.34	67.94	3.78	73.54	52.84
Max	92.9	90.13	92	88.84	31.45	99.94	69.08
Min	71.99	73.21	57.77	60.55	0.09	67.51	33.17
NTR 1+FOX 0.9+STR 2.3							
Median (n=3)	69.32	72.8	67.78	65.83	13.34	42.95	4.62
Max	74.36	89.07	82.42	66.89	20.75	46.77	21.26
Min	65.94	66.82	64.39	35.14	8.99	41.66	1.3
NTR 1+FOX 0.9+TOB 0.6							
Median (n=3)	82.97	73.36	79.15	69.35	16.18	43.55	1.27
Max	83.52	78.23	81.25	77.64	25.69	54.67	17.93
Min	75.23	59.8	66.21	63.64	1.48	38.29	1.24
NTR 1+STR 2.3+TOB 0.8							
Median (n=3)	74	79.45	70.8	22.43	0.85	1.2	0.64
Max	91.5	89.14	92	50.41	3.78	1.79	0.72
Min	64.12	69.13	59.24	5.7	0.09	0.8	0.45
GEN 0.4+TMP 0.07+AMP 1.2							

Median (n=4)	74.195	57.82	74.585	24.635	29.315	0.53	0.675
Max	79.85	62.85	84	27.47	34.57	2.21	1.02
Min	69.55	53.99	66.66	22.38	22.38	0.43	0.38
GEN 0.4+TMP 0.1+CPR 0.01							
Median (n=3)	73.74	87.29	78.21	51.36	65.4	51.4	4.32
Max	74.17	88.09	85.25	56.01	65.72	54.09	15.75
Min	69.64	85.33	71.4	48.34	59.34	51.2	1.55
GEN 0.4+TMP 0.1+CLI 20							
Median (n=3)	73.74	87.29	74.96	51.36	69.53	38.25	38.46
Max	74.17	88.09	80.7	56.01	73.41	41.04	39.48
Min	69.64	85.33	70.01	48.34	68.22	37.9	36.33
GEN 0.4+TMP 0.1+FOX 1							
Median (n=3)	73.74	87.29	75.95	51.36	20.7	58.52	37.32
Max	74.17	88.09	78.5	56.01	20.71	63.32	40.63
Min	69.64	85.33	70.36	48.34	18.9	53.13	22.18
GEN 0.4+CHL 40+TMP 0.1							
Median (n=5)	76.97	91.51	57.08	80.89	7.78	51.61	34.52
Max	85.49	94.18	59.12	84.75	21.49	66.47	55.65
Min	71.76	83.49	49.15	68.93	1.25	45.74	18.39

GEN 0.4+TMP 0.07+NTR 1							
Median (n=4)	72.01	81.15	86.35	23.105	49.11	52.735	0.695
Max	73.29	84.54	90.25	32.54	51.69	63.26	1.3
Min	70.72	79.07	83.75	13.24	36.43	50.72	0.45
GEN 0.4+CLI 20+STR 20							
Median (n=2)	87.05	81.08	87.82	80.175	71.47	72.7	62.84
Max	88.1	82.85	87.99	80.93	75.01	79.67	68.3
Min	86	79.31	87.65	79.42	67.93	65.73	57.38
GEN 0.4+NTR 1+FOX 1							
Median (n=5)	77.83	78.73	77.84	68.06	54.34	69.87	67.14
Max	84.25	88.5	87.57	83.92	61.3	85.41	75.22
Min	70.13	64.22	68.66	59.43	43.13	60.98	47.42
GEN 0.4+TOB 0.5+FOX 1							
Median (n=3)	86	86.22	84.55	66.56	58.67	35.2	47.44
Max	88.1	88.96	91.72	76.44	75.66	50.41	69.49
Min	70.13	76.9	83.35	40.88	45.7	31.27	24.84
GEN 0.4+CLI 20+NTR 1							
Median (n=3)	77.16	75.96	68.35	72.48	68.06	57.14	62.43
Max	77.83	79.46	78.73	81.61	76.04	67.16	75.02

Min	70.13	75.11	64.22	63.52	59.43	43.05	61.24
GEN 0.4+CLI 20+TOB 0.5							
Median (n=3)	86	79.46	86.22	79.42	66.56	74.82	70.52
Max	88.1	82.85	88.96	80.93	76.44	75.24	75.96
Min	70.13	79.31	76.9	72.48	40.88	61.52	51.03
GEN 0.4+AMP 1.2+FOX 1							
Median (n=7)	80.88	82.49	75.44	45.83	44.98	23.27	16.98
Max	92.63	85.45	80.35	60.79	62.19	50.65	23.83
Min	72.42	74.28	67.16	29.52	19.43	16.86	8.23
GEN 0.4+AMP 1.2+STR 1							
Median (n=3)	85.14	65.36	77.86	13.7	51.35	15.05	11.41
Max	88.44	72.93	86.3	13.74	59.86	26	19.4
Min	83.36	56.99	70.98	13.09	48.32	9.48	7.48
GEN 0.4+AMP 1.2+TOB 0.5							
Median (n=5)	86.19	93.47	83.32	49.64	59.36	39.12	15.22
Max	91.35	94.94	90.89	57.53	73.98	49.56	20.55
Min	80.72	83.39	73.17	40.24	51.54	22.03	8.24
GEN 0.4+AMP 1.2+NTR 1							
Median (n=5)	72.73	82.49	87.47	51.8	64.05	80.49	42.82

Max	80.88	90.24	93.76	60.79	68.2	89.44	56.66
Min	67.86	79.59	80.73	29.52	61.33	70.46	41.89
GEN 0.4+CPR 0.01+ERY 30							
Median (n=3)	85.14	65.36	63.46	13.7	69.86	41.05	22.76
Max	88.44	72.93	65.15	13.74	71.33	42.22	50.52
Min	83.36	56.99	51.95	13.09	63.42	34.21	21.14
GEN 04+CPR 0.01+FOX 1							
Median (n=5)	73.75	91.21	73.83	44.49	64.85	73.97	41.47
Max	80.72	94.16	83.14	47.58	71.03	78	48.55
Min	72.13	82.47	67.16	40.29	63.55	71.04	40.22
GEN 0.4+CPR 0.01+TOB 0.5							
Median (n=3)	77.52	88.17	70.99	62.11	67.02	63.46	34.18
Max	77.54	91.91	75.36	71.75	79.37	70.74	64.8
Min	68.88	85.5	69.74	51.77	51.57	50.25	0.92
GEN 0.4+NTR 1.2+CPR 1							
Median (n=4)	77.53	90.455	86.835	59.34	62.645	66.65	10.48
Max	87.47	95	91.91	90.86	71.75	93.52	56.05
Min	68.88	78.43	83.8	31.22	51.77	52.14	1.33
GEN 0.4+CLI 20+ERY 30							

Median (n=3)	83.37	76.49	62.91	64.03	61.5	56.01	53.49
Max	85.85	78.59	65.78	78.75	69.08	58.16	55.87
Min	79.79	73.05	61.54	61.02	57.8	55.11	48.43
GEN 0.55+CLI 17.5+FOX 0.9							
Median (n=2)	62.445	77.225	74.935	36.83	37.875	37.84	38.2
Max	63.56	83.55	82.44	53.96	52.93	41.81	39.62
Min	61.33	70.9	67.43	19.7	22.82	33.87	36.78
GEN 0.4+ERY 30+NTR 1							
Median (n=3)	85.98	89.46	90.6	77.27	75.14	75.62	68.37
Max	86.29	94.29	91.18	79.52	77.48	79.04	70.76
Min	81.98	89.29	89.48	76.63	69.86	74.15	65.68
GEN 0.4+CPR 0.01+CLI 20							
Median (n=3)	85.98	88.83	75.61	70.99	72.39	62.66	54.42
Max	86.29	89.61	76.71	73.39	73.27	63.08	58.23
Min	81.98	86.97	72.25	64.34	69.72	55.6	53.35
GEN 0.4+STR 2+NTR 1							
Median (n=6)	85.13	72.56	86.86	59.315	66.32	55.145	20.66
Max	85.36	74.49	87.92	77.57	68.84	64.95	23.83
Min	79.93	66.27	84.39	50.32	0.81	53.11	14.43

GEN 0.4+CHL 50+FOX 1							
Median (n=6)	85.13	78.96	68.82	77.235	44.8	20.425	26.93
Max	85.36	84.94	71.64	89.52	50.55	36.65	51.86
Min	79.93	76.17	62.33	70.41	40.74	15.53	15.3
GEN 0.4+AMP 1.2+CLI 20							
Median (n=5)	73.74	76.44	72.88	21.5	68.22	42.77	9.77
Max	76.97	79.85	80.7	40.43	73.41	44.29	29.53
Min	69.64	63.83	62.13	15.73	66.8	7.02	2.84
GEN 0.4+CHL 40+CLI 20							
Median (n=4)	81.77	82.46	79.4	80.335	72.47	78.72	80.14
Max	84.48	88.92	88.48	84.87	86	79.7	89.06
Min	72.36	69.63	75.72	74.6	62.96	67.94	68.15
GEN 0.4+ERY 30+TOB 0.5							
Median (n=3)	77.02	71.58	76.48	67.48	64.53	72.14	67.98
Max	77.33	74.29	82.02	69.93	65.48	72.99	70.25
Min	71.1	67.61	74.44	65.54	63.39	68.19	65.33
GEN 0.4+ERY 30+FOX							
Median (n=3)	77.02	71.58	73.27	67.48	18.55	32.15	35.12
Max	77.33	74.29	82.56	69.93	19.75	36.25	37.37

Min	71.1	67.61	71.14	65.54	16.78	31.89	27.14
GEN 0.4+CHL 40+AMP 1.2							
Median (n=4)	77.515	89.02	66.385	77.965	29.47	15.345	5.685
Max	85.49	94.18	76.48	84.75	34.72	20.37	6.52
Min	71.76	83.49	59.56	68.93	21.5	14.02	4.4
GEN 0.4+CHL 40+NTR 1							
Median (n=4)	83.755	70.81	90.355	66.825	74.41	72.485	61.205
Max	96.43	78.37	94.45	76.31	78.68	74.41	67.52
Min	77.04	67.11	84.01	65.18	70.12	69.88	57.05
GEN 0.4+AMP 1.2+ERY 30							
Median (n=4)	61.255	84.92	77.755	24.655	63.8	19.45	9.88
Max	64.19	89.34	83.31	27.27	70.48	31.61	10.24
Min	55.11	76.92	72.52	19.85	62.29	16.65	1.25
GEN 0.4+AMP 1.2+CPR 0.01							
Median (n=3)	62.23	86.39	83.96	26.43	50.23	45.11	4.45
Max	64.19	89.34	85.54	27.27	59.08	50.01	6.16
Min	55.11	76.92	79.02	22.88	47.22	28.1	4.31
FUS 80+CHL 50+CPR 0.01							
Median (n=4)	64.875	79.73	89.35	43.56	43.92	77.185	27.595

Max	73.26	83.54	92.48	48.96	57.89	79.21	32.91
Min	55.82	73.93	86.58	38.07	38.9	71.71	25.73
FUS 80+CHL 50+STR 2							
Median (n=4)	64.875	79.73	69.875	43.56	38.53	69.075	23.505
Max	73.26	83.54	72.08	48.96	40.1	71.53	34.35
Min	55.82	73.93	68.61	38.07	29.35	67.1	19.78
FUS 80+CHL 50+NTR 1							
Median (n=4)	64.875	79.73	86.905	43.56	73.75	68.655	45.405
Max	73.26	83.54	88.79	48.96	75.67	68.89	48.39
Min	55.82	73.93	86.29	38.07	72.04	68.13	34.69
FUS 80+CHL 50+TOB 0.5							
Median (n=4)	64.875	79.73	73.49	43.56	30.625	74.24	32.47
Max	73.26	83.54	73.64	48.96	34.78	76.65	39.39
Min	55.82	73.93	72.27	38.07	20.77	72.43	26.01
FUS 80+CHL 50+CLI 20							
Median (n=4)	64.875	79.73	72.235	43.56	60.55	72.38	32.68
Max	73.26	83.54	77.5	48.96	61.99	76.41	37.4
Min	55.82	73.93	70.82	38.07	50.15	63.53	31.51
FUS 80+GEN 0.4+TMP 0.07							

Median (n=3)	77.68	72.51	81.59	42.87	46.3	62.03	0.99
Max	79.17	73.98	82.36	49.16	58.6	64.63	4.01
Min	74.9	67.44	73.56	40.45	43.22	48.93	0.69
FUS 80+GEN 0.4+CLI 20							
Median (n=5)	78.38	70.99	69.82	42.87	62.54	71	55.38
Max	79.17	73.98	74.93	56.65	70.14	76	57.19
Min	74.9	67.44	65.86	40.45	59.45	68.36	49.73
FUS 80+GEN 0.4+TOB 0.5							
Median (n=5)	78.38	70.99	62.08	42.87	37.56	36.49	1.82
Max	79.17	73.98	64.91	56.65	42.84	46.44	7.81
Min	74.9	67.44	54.91	40.45	31.03	30.22	0.9
FUS 80+CLI 20+TOB 0.5							
Median (n=5)	78.38	69.82	62.08	62.54	37.56	66.1	48.88
Max	79.17	74.93	64.91	70.14	42.84	67.85	62.29
Min	74.9	65.86	54.91	59.45	31.03	60.49	45.43
FUS 80+TMP 0.07+CLI 20							
Median (n=3)	77.68	81.59	72.76	46.3	68.31	39.29	1.33
Max	79.17	82.36	74.93	58.6	70.14	46.31	5.24
Min	74.9	73.56	68.21	43.22	62.54	31.81	1.32

FUS 80+TMP 0.07+TOB 0.5							
Median (n=3)	77.68	81.59	62.08	46.3	37.19	50.16	1.11
Max	79.17	82.36	64.91	58.6	41.68	50.5	1.38
Min	74.9	73.56	57.07	43.22	31.03	38.71	0.81
FUS 80+NTR 1+FOX							
Median (n=4)	65.255	85.585	60.125	69.2	19.785	61.145	28.745
Max	68.41	91.39	67.2	71.34	26.69	62.29	34.93
Min	61.78	78.17	55.93	60.57	18.01	54.57	24.86
FUS 80+NTR 1+CLI 20							
Median (n=4)	65.255	85.585	74.995	69.2	46.825	69.415	46.315
Max	68.41	91.39	77.24	71.34	50.41	76.12	53.22
Min	61.78	78.17	66.52	60.57	41.26	63.41	38.54
FUS 80+NTR 1+TOB 0.5							
Median (n=4)	65.255	85.585	70.065	69.2	23.505	59.255	32.91
Max	68.41	91.39	75.43	71.34	29.82	65.06	35.5
Min	61.78	78.17	67.84	60.57	15.33	54.12	26.81
FUS 80+FOX 1+CLI 20							
Median (n=4)	65.255	60.125	74.995	19.785	46.825	33.58	17.51
Max	68.41	67.2	77.24	26.69	50.41	43.19	24.44

Min	61.78	55.93	66.52	18.01	41.26	26.04	15.23
FUS 80+FOX 1+TOB 0.5							
Median (n=4)	65.255	60.125	70.065	19.785	23.505	43.39	3.03
Max	68.41	67.2	75.43	26.69	29.82	53.28	4.78
Min	61.78	55.93	67.84	18.01	15.33	40.96	1.95
FUS 80+CHL 50+FOX 1							
Median (n=4)	65.255	77.02	60.125	35.645	19.785	28.32	2.04
Max	68.41	83.01	67.2	38.76	26.69	37.25	4.85
Min	61.78	67.75	55.93	24.6	18.01	24.97	1.1
FUS 80+AMP 1.2+CPR 0.01							
Median (n=4)	73.075	73.935	89.29	0.85	51.025	45.05	0.635
Max	74.41	78.44	90.54	1.24	66.78	52.73	0.67
Min	64.71	68.84	87.25	0.42	46.39	25.71	0.57
FUS 80+AMP 1.2+STR 2							
Median (n=4)	73.075	73.935	69.8	0.85	45.08	27.46	0.6
Max	74.41	78.44	75.63	1.24	48.87	29.23	0.71
Min	64.71	68.84	66.88	0.42	32.08	25.96	0.54
FUS 80+AMP 1.2+NTR 1							
Median (n=4)	73.075	73.935	86.63	0.85	76.495	65.46	13.135

Max	74.41	78.44	96.78	1.24	78.07	77.24	19.52
Min	64.71	68.84	83.31	0.42	71.34	50.85	6.26
FUS 80+AMP 1.2+CLI 20							
Median (n=4)	73.075	73.935	73.415	0.85	62.505	15.45	0.565
Max	74.41	78.44	84.88	1.24	68.63	18.15	1.46
Min	64.71	68.84	72.16	0.42	58.07	10.94	0.49
FUS 80+AMP 1.2+TOB 0.5							
Median (n=4)	73.075	73.935	73.725	0.85	38.575	28.685	0.565
Max	74.41	78.44	77.75	1.24	48.96	30.55	0.64
Min	64.71	68.84	70.41	0.42	27.11	23.56	0.55
FUS 80+STR 2+CLI 20							
Median (n=4)	64.81	75.67	62.305	39.245	49.11	60.255	36.83
Max	68.57	82.23	69.94	46.15	54.93	63.15	61.46
Min	61.96	65.69	60.87	36.07	42.37	53.56	23.3
FUS 80+CPR 0.01+CLI 20							
Median (n=5)	65.61	82.69	62.04	45.58	51.61	47.36	25.51
Max	68.61	86.76	69.94	98.94	54.93	55.74	39.94
Min	61.96	75.02	60.8	42.46	42.37	37.02	22.64
FUS 80+ERY 30+CLI 20							

Median (n=5)	65.61	65.1	62.04	38.46	51.61	48.85	20.7
Max	68.61	69.7	69.94	44.84	54.93	58.68	29.61
Min	61.96	56.73	60.8	34.31	42.37	46.07	14.54
FUS 80+TMP 0.07+CPR 0.01							
Median (n=4)	64.81	56.315	83.99	43.045	48.8	43.265	13.87
Max	68.57	63.81	86.76	52.57	98.94	49.15	21.2
Min	61.96	54.49	75.02	38.77	42.46	37.47	10.51
FUS 80+TMP 0.07+STR 2							
Median (n=4)	64.81	56.315	75.67	43.045	39.245	82.835	11.095
Max	68.57	63.81	82.23	52.57	46.15	99.42	12.86
Min	61.96	54.49	65.69	38.77	36.07	78.88	10.47
FUS 80+TMP 0.07+CHL 50							
Median (n=4)	76.265	84.25	87.33	53.57	55.425	77.45	45.055
Max	84.26	85.68	93.4	64.26	58.11	92.88	52.33
Min	69.67	79.13	83.51	51.68	47.71	66.84	31.99
FUS 80+TMP 0.07+AMP 1.2							
Median (n=3)	77.92	85.67	64.01	53.77	0.61	1.79	0.78
Max	84.26	85.68	70.38	64.26	0.76	3.42	0.8
Min	74.61	82.83	60.13	53.37	0.54	0.85	0.72

FUS 80+TMP 0.07+FOX 1							
Median (n=3)	77.92	85.67	50.91	53.77	31.44	25.04	18.2
Max	84.26	85.68	55.83	64.26	33.44	31.74	22.07
Min	74.61	82.83	45.45	53.37	18.93	23.01	14.27
FUS 80+TMP 0.07+ERY 30							
Median (n=3)	74.61	85.67	84.2	53.77	9.71	50.92	0.91
Max	84.26	85.68	93.36	64.26	48.6	52.71	1.39
Min	69.67	79.13	70.89	51.68	9.61	45.81	0.88
FUS 80+TMP 0.07+NTR 1							
Median (n=4)	76.265	84.25	78.345	53.57	71.62	66.31	21.965
Max	84.26	85.68	80.55	64.26	72.55	72.34	36.73
Min	69.67	79.13	76.43	51.68	67.22	47.69	4.68
FUS 80+ERY 30+CPR 0.01							
Median (n=3)	55.04	83.41	85.9	37.06	61.06	77.19	15.89
Max	57.85	92.06	91.45	42.47	64.95	92.52	18.09
Min	47.91	76.32	79.86	10.53	37.86	76.96	15.52
FUS 80+ERY 30+STR 2							
Median (n=5)	57.85	84.46	73.33	37.06	40.15	71.76	18.94
Max	62.91	92.06	74.74	45.89	46.54	80.74	33.05

Min	47.91	76.32	60.78	10.53	33.74	64.86	3.75
FUS 80+ERY 30+NTR 1							
Median (n=5)	57.85	84.46	83.66	37.06	64.84	71.56	52.98
Max	62.91	92.06	94.33	45.89	75.22	76.82	60.41
Min	47.91	76.32	77.84	10.53	53.73	63.51	45.69
FUS 80+ERY 30+FOX 1							
Median (n=4)	56.445	87.58	70.61	24.08	28.99	46.41	6.78
Max	62.91	92.06	77.79	42.47	35.96	48.13	12.99
Min	47.91	76.32	64.5	10.53	14.49	27.62	2.11
FUS 80+GEN 0.4+FOX 1							
Median (n=4)	56.445	77.27	70.61	45.8	28.99	37.96	8.125
Max	62.91	83.28	77.79	49.81	35.96	45.67	13.29
Min	47.91	68.55	64.5	33.56	14.49	36.86	6.79
VAN 75+FUS 80+CPR 0.01							
Median (n=3)	69.06	61.18	74.43	26.61	5.19	43.68	0.49
Max	78.2	70.84	89.49	35.18	19.22	53.91	0.9
Min	61.2	57.69	72.9	23.89	2.51	35.42	0.47
VAN 75+FUS 80+ERY 30							
Median (n=4)	69.39	67.125	98.235	28.98	32.17	13.575	2.485

Max	73.53	70.84	112.98	35.18	74.52	34.33	4.66
Min	61.2	61.18	95.43	26.61	28.56	6.06	0.65
VAN 75+FUS 80+FOX 1							
Median (n=4)	71.625	63.675	72.885	27.21	60.45	36.28	10.34
Max	78.2	68.08	80.81	30.15	70.12	40.13	15.22
Min	69.06	57.69	63.16	23.89	53.61	34.11	5.22
VAN 75+FUS 80+CLI 20							
Median (n=4)	69.39	63.675	67.865	28.38	18.71	46.765	0.885
Max	78.2	70.84	79.78	35.18	39.67	52.38	1.82
Min	61.2	57.69	62.83	23.89	16.91	45.13	0.52
VAN 75+FUS 80+TOB 0.5							
Median (n=4)	86.5	63.23	89.26	16.755	69.615	47.135	1.09
Max	94.74	69.54	93.35	36.88	79	52.32	3.62
Min	78.15	60.88	85.51	10.11	67.56	36.92	0.76
VAN 75+FUS 80+GEN 0.4							
Median (n=5)	73.62	68.01	90.45	33.92	48.92	34.45	3.47
Max	84.89	69.15	97.33	47.57	56.31	35.69	6.32
Min	70.85	64.1	89.42	30.16	42.49	29.25	1.01
VAN 75+FUS 80+CHL 50							

Median (n=5)	73.62	68.01	88.79	33.92	38.51	34.83	1.5
Max	84.89	69.15	91.42	47.57	43.2	49.41	2.66
Min	70.85	64.1	86.52	30.16	33.32	32.91	1.28
VAN 75+FUS 80+TMP 0.07							
Median (n=5)	73.62	68.01	75.58	33.92	0.8	44.23	3.38
Max	84.89	69.15	82.56	47.57	0.93	56.63	13.7
Min	70.85	64.1	60.59	30.16	0.57	36.68	0.87
VAN 75+FUS 80+NTR 1							
Median (n=5)	73.62	68.01	89.95	33.92	63.37	77.18	30.44
Max	84.89	69.15	98.89	47.57	72.52	78.63	34.47
Min	70.85	64.1	81.82	30.16	56.02	64.64	21.57
VAN 75+FUS 80+AMP 1							
Median (n=5)	91.99	62.72	79.38	14.89	53.79	0.93	0.68
Max	95.32	69.54	87.66	36.88	58.69	2.63	0.81
Min	78.15	50.07	74.51	3.85	46.08	0.69	0.58
VAN 75+GEN 0.4+STR 2							
Median (n=4)	73.91	69.78	65.485	36.725	25.845	37.98	18.32
Max	85.9	75.08	71.79	40.07	36.98	50.37	20.17
Min	68.28	62.73	61.38	32.96	23.72	31.9	10.23

VAN 75+GEN 0.4+ERY 30							
Median (n=5)	76.78	67.9	94.15	35.08	34.97	62.85	39.6
Max	85.9	75.08	100.64	40.07	57.94	86.13	52.17
Min	68.28	62.73	86.15	32.52	28.54	57.84	26.13
VAN 75+GEN 0.4+FOX 1							
Median (n=4)	72.71	66.65	74.965	34.02	49.78	26.97	15.335
Max	79.18	71.66	80.42	40.07	65.6	28.52	23.06
Min	68.28	62.73	64.91	32.52	40.93	22.24	12.62
VAN 75+GEN 0.4+CLI 20							
Median (n=5)	76.78	67.9	72.81	35.08	36.45	61.68	27.15
Max	85.9	75.08	90.14	40.07	45.82	75.56	33.24
Min	68.28	62.73	68.39	32.52	24.67	51.72	22.03
VAN 75+FUS 80+STR 2							
Median (n=4)	73.91	67.145	65.485	28.355	25.845	38.42	0.52
Max	85.9	74.4	71.79	41.19	36.98	45.44	0.61
Min	68.28	67	61.38	8.24	23.72	34.52	0.43
VAN 75+GEN 0.4+CHL 50							
Median (n=5)	83.35	80.1	85.44	60.81	52.06	83.26	45.82
Max	88.31	81.18	88.69	65.74	62.04	86.02	47.81

Min	79.15	79.75	80.59	54.33	50.79	77.93	38.01
VAN 75+GEN 0.4+TMP 0.07							
Median (n=5)	83.35	80.1	84.32	60.81	0.37	65.69	0.65
Max	88.31	81.18	91.25	65.74	0.74	67.46	20.76
Min	79.15	79.75	78.56	54.33	0.21	51.77	0.14
VAN 75+GEN 0.4+NTR 1							
Median (n=5)	83.35	80.1	85.95	60.81	58.08	66.92	38.07
Max	88.31	81.18	86.65	65.74	61.08	68.6	43.04
Min	79.15	79.75	80.31	54.33	54.79	63.32	22.49
VAN 75+GEN 0.4+TOB 0.5							
Median (n=5)	83.35	80.1	85.13	60.81	69.74	66.18	40.99
Max	88.31	81.18	86.86	65.74	77.76	67.09	43.8
Min	79.15	79.75	84.4	54.33	62.34	60.76	23.98
VAN 75+AMP 1+NTR 1							
Median (n=5)	92.79	79.38	86.8	53.79	72.66	72.18	30.41
Max	95.79	85.36	93.55	58.69	75.66	73.25	49.44
Min	81.01	74.54	84.51	47.76	57.53	70.94	28.38
VAN 75+AMP 1+TOB 0.5							
Median (n=6)	93.765	80.7	88.385	54.78	76.615	41.645	39.2

Max	95.79	87.66	94.1	58.5	82.11	63.07	40.82
Min	91.99	69.18	83.29	47.76	67.28	31.54	30.91
VAN 75+AMP 1+FOX 1							
Median (n=6)	93.765	80.7	73.12	54.78	71.775	23.745	17.505
Max	95.79	87.66	80.86	58.5	80.22	27.21	20.62
Min	91.99	69.18	63.26	47.76	57.12	21.53	15.24
VAN 75+AMP 1+CLI 20							
Median (n=4)	89.575	81.76	70.4	54.54	40.245	20.035	6.295
Max	95.79	89.44	77.84	56.31	49.19	21.95	13.76
Min	81.06	74.54	69.92	47.9	32.56	17.21	0.85
VAN 75+NTR 1+TOB 0.5							
Median (n=4)	94.15	86.74	83.895	74.9	78.17	77.065	59.54
Max	95.79	89.34	84.65	75.66	82.32	79.27	60.89
Min	92.24	85.36	83.29	63.96	67.28	75.74	57.72
VAN 75+NTR 1+CPR 0.01							
Median (n=5)	86.11	88.1	87.69	54.39	48.99	79.61	3.88
Max	101.73	89.94	88.66	55.85	54.5	82.03	8.87
Min	77.28	80.05	84.89	53.77	24.09	69.24	0.72
VAN 75+NTR 1+STR 2							

Median (n=7)	86.11	89.94	74.92	55.85	54.91	63.89	30.88
Max	94.02	113.89	96.72	80.99	61.67	80.14	57.07
Min	77.28	80.05	70.9	53.77	39.69	55.68	2.84
VAN 75+NTR 1+FOX 1							
Median (n=4)	83.395	87.22	70.905	54.6	70.47	56.17	49.41
Max	94.02	89.94	72.86	55.85	72.67	58.44	55.16
Min	77.28	80.05	64.43	53.77	64.01	49.73	46.35
VAN 75+NTR 1+ERY 30							
Median (n=4)	83.395	87.22	85.305	54.6	65.715	74.965	37.43
Max	94.02	89.94	88.02	55.85	74.24	76.19	50.59
Min	77.28	80.05	78.52	53.77	61.58	70.03	36.96
VAN 75+NTR 1+CLI 20							
Median (n=4)	83.395	87.22	72.01	54.6	46.715	68.335	0.68
Max	94.02	89.94	75.07	55.85	47.21	73.54	0.74
Min	77.28	80.05	67.69	53.77	45.11	61.8	0.61
VAN 75+TOB 0.5+FOX 1							
Median (n=3)	96.7	79.83	64.52	49.96	59.76	32.33	19.82
Max	99.82	86.82	72.79	55.27	65.24	36.01	28.89
Min	90.73	78.82	57.41	45.86	58.25	31.08	17.05

VAN 75+TOB 0.5+ERY 30							
Median (n=3)	96.7	79.83	84.07	49.96	48.65	57.11	56.8
Max	99.82	86.82	91.4	55.27	51.8	62.17	60.82
Min	90.73	78.82	80.29	45.86	46.59	55.23	39.18
VAN 75+TOB 0.5+CLI 20							
Median (n=3)	96.7	79.83	69.62	49.96	40.09	66.54	27.63
Max	99.82	86.82	72.17	55.27	43.37	73.19	37.35
Min	90.73	78.82	66.7	45.86	36.14	56.65	27.06
VAN 75+FOX 1+ERY 30							
Median (n=3)	96.7	64.52	84.07	59.76	48.65	27.1	13.72
Max	99.82	72.79	91.4	65.24	51.8	28.39	13.93
Min	90.73	57.41	80.29	58.25	46.59	24.1	8.87
VAN 75+FOX 1+CLI 20							
Median (n=3)	96.7	64.52	69.62	59.76	40.09	24.22	14.64
Max	99.82	72.79	72.17	65.24	43.37	25.88	15.03
Min	90.73	57.41	66.7	58.25	36.14	20.89	9.61
VAN 75+ERY 30+CLI 20							
Median (n=3)	96.7	84.07	69.62	48.65	40.09	57.47	18.9
Max	99.82	91.4	72.17	51.8	43.37	59.56	20.74

Min	90.73	80.29	66.7	46.59	36.14	50.37	11.34
VAN 75+CHL 50+TOB 0.5							
Median (n=3)	74.25	83.86	94.35	62.86	67.72	83.63	47.05
Max	85	91.06	94.78	64.99	71.85	94.66	48.49
Min	65.39	83.2	91.75	60.87	67.43	82.75	44.58
VAN 75+CHL 50+FOX 1							
Median (n=5)	75.51	85	80	62.86	50.14	27.93	24.15
Max	85	92.47	82.5	67.63	58.29	29.34	31.5
Min	65.39	83.2	71.26	57.31	41.94	20.18	19.34
VAN 75+CHL 50+ERY 30							
Median (n=5)	75.51	85	90.41	62.86	58.66	66.45	60.34
Max	85	92.47	96.09	67.63	71.83	73.42	67.91
Min	65.39	83.2	87.1	57.31	52.55	59.05	52.52
VAN 35+TMP 0.07+CLI 20							
Median (n=5)	105.69	85.68	76.21	11.01	66.68	26.53	0.88
Max	113.23	90.99	79.53	33.21	73.7	34.87	1.07
Min	97.41	75.51	74.35	3.78	64.55	9.73	0.82
VAN 35+TMP 0.07+STR 2							
Median (n=5)	105.69	85.68	72.56	11.01	70.59	57.46	10.32

Max	113.23	90.99	73.65	33.21	83.81	60.92	30.03
Min	97.41	75.51	68.06	3.78	63.29	50.52	8.71
VAN 35+TMP 0.07+TOB 0.5							
Median (n=5)	105.69	85.68	81.78	11.01	92.07	78.5	30.99
Max	113.23	90.99	85.04	33.21	110.94	82.68	36.25
Min	97.41	75.51	76.93	3.78	82.04	58.28	29.26
VAN 35+CHL 50+TMP 0.07							
Median (n=5)	105.69	86.15	85.68	92.41	11.01	77.65	51.9
Max	113.23	90.39	90.99	97.52	33.21	86.54	53.04
Min	97.41	84.15	75.51	75.94	3.78	74.6	48.67
VAN 35+TMP 0.07+NTR 1							
Median (n=7)	91.49	60.26	91.36	13.27	80.99	38.55	9.52
Max	100.11	107.32	93.49	54.64	83.03	79.66	59.26
Min	86.99	51.47	85.94	5.27	72.89	18.53	0.71
VAN 35+TMP 0.07+CPR 0.01							
Median (n=5)	88.85	59.16	86	7.65	70.78	37.3	6.32
Max	91.49	60.97	93.63	26.58	74.27	41.84	16.88
Min	80.99	51.47	80.31	3.17	62.09	34.06	0.76
VAN 35+TMP 0.07+FOX 1							

Median (n=8)	87.92	60.615	67.31	8.425	56.35	31.415	1.075
Max	95.75	75.36	75.57	26.58	83.1	38.73	21.7
Min	74.9	51.47	54.62	1.78	38.84	19.76	0.81
VAN 35+TMP 0.07+ERY 30							
Median (n=6)	87.92	59.71	82.225	10.92	62.425	40.935	10.94
Max	91.49	62.21	89.47	13.56	88.63	55.07	15.51
Min	79.11	51.47	75.6	1.77	50.33	27.13	6.29
CHL 40+TMP 0.07+AMP 1.2							
Median (n=3)	76.21	72.76	75.12	72.67	73.84	0.69	0.97
Max	77.2	78.48	80.58	75.19	86.14	56.62	44.57
Min	75.13	71.6	71.15	57.72	52.89	0.58	0.92
CHL 40+TMP 0.07+CPR 9							
Median (n=2)	76.705	72.18	81.835	65.195	64.085	0.925	31.835
Max	77.2	72.76	82.88	72.67	67.37	0.93	62.96
Min	76.21	71.6	80.79	57.72	60.8	0.92	0.71
CHL 40+TMP 0.07+CLI 17.5							
Median (n=3)	76.21	71.6	76.74	57.72	69.09	12.96	19.82
Max	77.2	72.76	79.34	72.67	81.72	29.56	43.53
Min	68.07	59.61	75.55	49.44	69.06	11.68	2.87

CHL 40+TMP 0.06+ERY 18							
Median (n=3)	71.24	68.22	78.03	66.12	67.73	57.06	52.74
Max	77.13	78.06	79.92	82.51	82.94	79.46	61.12
Min	64.88	62.23	77.05	64.67	62.08	48.05	47.35
CHL 40+TMP 0.06+FOX 0.9							
Median (n=3)	71.24	68.22	66.98	66.12	49.92	25.52	24.9
Max	77.13	78.06	71.74	82.51	69.67	45.27	34.95
Min	64.88	62.23	63.72	64.67	48.51	14.42	24.68
CHL 40+TMP 0.06+STR 2.3							
Median (n=3)	71.24	68.22	66.96	66.12	54.27	46.72	37.12
Max	77.13	78.06	79.65	82.51	76.38	77.58	45.31
Min	64.88	62.23	65.81	64.67	53.87	10.77	4.71
CHL 40+TMP 0.08+TOB 0.5							
Median (n=2)	83.77	83.77	83.77	83.77	83.77	83.77	83.77
Max	85.36	85.36	85.36	85.36	85.36	85.36	85.36
Min	82.18	82.18	82.18	82.18	82.18	82.18	82.18
CHL 40+TMP 0.06+NTR 1							
Median (n=4)	72.38	80.495	64.11	72.92	61.855	61.625	66.12
Max	75.13	89.19	76.52	102.93	67.67	93.86	72.07

Min	71.14	76.62	60.43	70.65	48.09	59.3	61.01
CHL 40+AMP 1.3+CPR 9							
Median (n=3)	81.99	80.58	69.18	82.07	58.86	35.72	48.46
Max	89.11	81.87	84.26	86.14	70.15	40.22	67.32
Min	75.13	66.01	65.97	75.22	58.63	0.22	15.86
CHL 40+AMP 1.3+CLI 17.5							
Median (n=3)	76.21	75.12	75.55	73.84	74.79	30.24	61.96
Max	77.2	79.2	76.74	84.62	81.72	78.16	71.78
Min	72.01	71.15	65.44	52.89	69.09	16.15	8.83
CHL 40+AMP 1.3+ERY 18							
Median (n=3)	69.66	69.15	70.98	79.22	62.3	86.58	59.64
Max	81.62	86.97	89.23	94.52	71.45	88.29	72.99
Min	65.52	65.43	66.45	61.17	36.87	78.02	49.18
CHL 40+AMP 1.3+FOX 0.9							
Median (n=2)	75.475	96.71	86.965	71.29	54.28	53.38	26.765
Max	76.72	98.65	95.07	71.48	59.06	61.25	30.53
Min	74.23	94.77	78.86	71.1	49.5	45.51	23
CHL 40+AMP 1.3+STR 2.3							
Median (n=2)	75.64	76.2	71.455	77.845	56.625	52.295	46.105

Max	81.62	86.97	77.06	94.52	65.25	60.13	58.49
Min	69.66	65.43	65.85	61.17	48	44.46	33.72
CHL 40+AMP 1.3+TOB 0.6							
Median (n=3)	69.66	69.15	81.78	79.22	50.93	44.97	71
Max	81.62	86.97	85.53	94.52	66.46	62.56	74.52
Min	65.52	65.43	70.65	61.17	41.72	44.82	47.19
CHL 40+AMP 1.3+NTR 1							
Median (n=5)	75.13	69.15	66.99	79.22	66.78	46.83	51.37
Max	89.11	86.97	73.33	94.52	86.76	55.76	62.65
Min	65.52	65.43	65.66	61.17	43.48	43.49	39.08
CHL 40+CPR 9+CLI 17.5							
Median (n=3)	76.21	80.79	76.74	63.83	69.09	19.56	43.21
Max	77.2	82.88	79.34	67.37	81.72	28.69	66.28
Min	68.07	61.53	75.55	60.8	69.06	11.25	28.36
CHL 38+CPR 8+ERY 18							
Median (n=4)	77.205	74.83	78.64	66.86	63.785	68.51	63.135
Max	84.62	87.69	83.07	77.42	71.12	79.07	74.1
Min	71.14	67.55	75.43	20.44	34.29	36.72	27.86
CHL 40+CPR 12+FOX 0.9							

Median (n=3)	69.48	66.51	83.72	59.53	54.63	19.53	28.93
Max	71.47	68.72	86.3	79.09	60.68	20.48	35.4
Min	66.94	59.25	61.4	47.77	46.58	2.18	24.9
CHL 38+CPR 8+STR 2.3							
Median (n=3)	72.82	85.19	76.05	72.04	70.82	62.09	58.2
Max	76.9	89.95	93.93	76.2	82.66	69.27	72.31
Min	65.19	71.02	61.85	70.84	59.87	55.71	56.99
CHL 40+CPR 12+TOB 0.5							
Median (n=3)	69.7	66.38	85.22	74.69	65.86	57.22	59.47
Max	72.78	83.06	86.26	79.73	68.15	57.76	66.81
Min	66.94	59.25	68.15	47.77	65.44	6.49	46.07
CHL 40+CPR 12+NTR 1							
Median (n=2)	68.32	62.815	76.495	61.23	64.36	69.24	51.48
Max	69.7	66.38	80.15	74.69	66.28	75.45	58.86
Min	66.94	59.25	72.84	47.77	62.44	63.03	44.1
CHL 40+CLI 17.5+ERY 18							
Median (n=3)	82.18	83.12	79.82	68.68	66.29	61.22	55.8
Max	85.36	88.93	85.43	76	80.66	69.5	71.34
Min	74.23	76.7	75.26	52.96	64.6	36.37	37.1

CHL 40+CLI 17.5+FOX 0.9							
Median (n=2)	75.475	82.845	86.965	54.28	68.775	53.895	44.34
Max	76.72	83.12	95.07	59.06	68.87	56.58	52.39
Min	74.23	82.57	78.86	49.5	68.68	51.21	36.29
CHL 38+STR 2.3+CLI 17.5							
Median (n=3)	73.82	79.52	69.53	59.96	69.69	78.01	68.61
Max	78.89	93.93	90.84	83.47	71.59	78.64	72.3
Min	65.19	68.39	67.73	59.87	52.31	66.55	59.8
CHL 38+TOB 0.5+CLI 17.5							
Median (n=3)	78.89	81.17	69.53	82.15	67.84	77.74	78.88
Max	95.28	83.59	89.9	90.27	85.73	78.64	93.69
Min	73.82	67.47	67.73	79.56	60.42	66.55	72.93
CHL 40+CLI 17.5+NTR 1							
Median (n=2)	78.56	79.23	60.23	59.255	70.29	42.135	40.72
Max	81.99	84.33	65.66	70.68	75.3	46.14	45.58
Min	75.13	74.13	54.8	47.83	65.28	38.13	35.86
CHL 40+ERY 18+FOX 0.9							
Median (n=3)	71.24	78.03	66.98	67.73	49.92	46.42	22.12
Max	77.13	79.92	71.74	82.94	69.67	59.78	22.74

Min	64.88	77.05	63.72	62.08	48.51	32.09	19.59
CHL 40+ERY 18+STR 2.3							
Median (n=3)	71.24	78.03	66.96	67.73	54.27	67.24	58.55
Max	77.13	79.92	79.65	82.94	76.38	73.95	66.49
Min	64.88	77.05	65.81	62.08	53.87	60.23	34.92
CHL 40+ERY 18+TOB 0.6							
Median (n=3)	69.66	70.98	81.78	50.93	62.3	79.19	57.2
Max	81.62	89.23	85.53	71.45	66.46	83.78	61.79
Min	65.52	66.45	70.65	41.72	36.87	72.64	50.8
CHL 40+ERY 18+NTR 1							
Median (n=4)	70.905	74.5	67.75	66.875	69.82	65.07	66.14
Max	81.62	89.23	73.74	76.03	86.76	69.66	68.13
Min	65.52	66.45	65.7	36.87	66.78	55.13	57.19
CHL 40+FOX 0.9+STR 2.3							
Median (n=3)	71.24	66.98	66.96	49.92	54.27	19.75	33.36
Max	77.13	71.74	79.65	69.67	76.38	28.02	62.67
Min	64.88	63.72	65.81	48.51	53.87	18.24	24.34
CHL 40+FOX 0.9+TOB 0.5							
Median (n=2)	68.21	73.85	82.41	53.63	69.06	54.915	40.48

Max	69.48	86.3	85.22	60.68	69.97	81.63	42.53
Min	66.94	61.4	79.6	46.58	68.15	28.2	38.43
CHL 38+FOX 0.9+NTR 1							
Median (n=3)	74.29	68.53	69.85	59.16	61.28	63.05	66.5
Max	78.61	90.52	89.97	61.5	79.33	76.48	67.79
Min	72.82	64.17	60.18	50.67	58.93	55.35	52.09
CHL 38+STR 2.3+TOB 0.5							
Median (n=3)	74.74	73.18	71.46	71.69	45.91	69.43	45.54
Max	78.61	76.34	72.87	71.87	56.38	71.95	66.95
Min	74.29	63.1	67.24	67.78	42.5	43.61	34.89
CHL 38+STR 2.3+NTR 1							
Median (n=2)	76.45	74.76	75.075	71.78	69.13	54.09	56.055
Max	78.61	76.34	89.97	71.87	79.33	55.8	62.52
Min	74.29	73.18	60.18	71.69	58.93	52.38	49.59
CHL 40+NTR 1+TOB 0.5							
Median (n=3)	69.7	72.84	81.03	66.28	65.86	56.71	51.97
Max	87.49	80.15	85.22	71.75	68.15	69.51	60.97
Min	66.94	71.15	68.15	62.44	43.94	1.41	5.56
TMP 0.07+AMP 1.2+CPR 9							

Median (n=3)	65.96	76.79	74	17.68	18.83	41.03	1.09
Max	77.51	78	76.23	18.59	23.59	50	1.11
Min	64.8	72.24	71.25	16.76	8.21	38.71	1.04
TMP 0.07+AMP 1.2+CLI 17.5							
Median (n=4)	65.38	77.395	72.665	17.22	30.775	35.015	6.52
Max	77.51	81.94	75.19	18.59	35.72	42.93	18.05
Min	63.42	72.24	69.08	1.82	25.92	30.49	1.6
TMP 0.07+AMP 1.2+ERY 18							
Median (n=3)	65.96	76.79	68.79	17.68	62	82.43	23.68
Max	77.51	78	80.37	18.59	67.55	92.06	28.93
Min	64.8	72.24	65.82	16.76	60.32	78.21	22.78
TMP 0.08+AMP 1.1+FOX 0.9							
Median (n=2)	63.535	81.975	85.85	2.495	27.76	37.815	1.12
Max	66.4	90.64	92.74	3.61	31.46	42.44	1.17
Min	60.67	73.31	78.96	1.38	24.06	33.19	1.07
TMP 0.08+AMP 1.1+STR 2.3							
Median (n=2)	63.535	81.975	80.905	2.495	28.55	26.395	1.38
Max	66.4	90.64	89.81	3.61	31.29	26.48	1.79
Min	60.67	73.31	72	1.38	25.81	26.31	0.97

TMP 0.07+AMP 1.2+TOB 0.6							
Median (n=2)	68.565	70.995	68.08	13.35	31.56	75.49	1.185
Max	71.17	76.79	73.05	18.59	39.28	83.44	1.36
Min	65.96	65.2	63.11	8.11	23.84	67.54	1.01
TMP 0.08+AMP 1.1+NTR 1							
Median (n=2)	63.535	81.975	74.395	2.495	75.68	63.465	3.39
Max	66.4	90.64	78.25	3.61	81.46	67.63	3.48
Min	60.67	73.31	70.54	1.38	69.9	59.3	3.3
TMP 0.08+CPR 9+CLI 17.5							
Median (n=3)	66.5	68.41	74.37	8.21	24.86	29.89	1.41
Max	77.51	74	75.19	14.08	25.92	37.94	8.19
Min	62.51	65.42	73.39	1.32	23.24	19.18	0.86
TMP 0.08+CPR 9+ERY 18							
Median (n=2)	72.005	71.205	77.775	4.765	55.47	88.995	18.175
Max	77.51	74	86.76	8.21	67.55	100.14	23.06
Min	66.5	68.41	68.79	1.32	43.39	77.85	13.29
TMP 0.07+CPR 9+FOX 0.9							
Median (n=2)	61.1	73.805	82.09	14.41	25.47	13.635	2.73
Max	65.2	79.21	82.28	14.7	26.5	14.1	3.8

Min	57	68.4	81.9	14.12	24.44	13.17	1.66
TMP 0.08+CPR 9+STR 2.3							
Median (n=2)	72.32	69.64	71.695	7.935	3.455	2.225	0.885
Max	79.44	70.88	71.7	14.7	6.41	3.3	1.17
Min	65.2	68.4	71.69	1.17	0.5	1.15	0.6
TMP 0.08+CPR 9+TOB 0.6							
Median (n=2)	66.55	73.25	73	57.9	7.3	8.6	0.35
Max	68.7	78.7	77.8	57.9	14.3	16.9	0.5
Min	64.4	67.8	68.2	57.9	0.3	0.3	0.2
TMP 0.08+CPR 9+NTR 1							
Median (n=3)	64.4	67.8	69.2	57.9	73.9	68.2	58.8
Max	68.7	78.7	81.4	57.9	80.3	86.3	65.1
Min	59.66	63.79	66.7	15.13	73.54	66.79	49.42
TMP 0.08+CLI 17.5+ERY 18							
Median (n=3)	63.42	73.39	76.87	26.79	34.23	64.81	13.54
Max	66.5	78.91	86.76	43.39	46.8	69.96	16.21
Min	59.95	69.08	75.77	23.19	23.24	59.1	13.08
TMP 0.07+CLI 17.5+FOX 0.9							
Median (n=3)	65.2	69.93	71.15	27.7	26.5	34.2	27.2

Max	71.26	83.5	81.9	31.4	30.5	34.5	27.7
Min	51.5	67.9	70.2	24.42	14.27	25.82	20.66
TMP 0.07+CLI 17.5+STR 2.3							
Median (n=2)	72.1	71.8	66.7	53	0.7	59.95	13.85
Max	72.7	81.3	67.2	62.1	0.9	65.6	15.7
Min	71.5	62.3	66.2	43.9	0.5	54.3	12
TMP 0.08+CLI 17.5+TOB 0.6							
Median (n=2)	68.5	64.37	62.285	33.61	0.925	1.225	0.925
Max	69	65.44	64.4	66.1	1.25	1.45	1.25
Min	68	63.3	60.17	1.12	0.6	1	0.6
TMP 0.08+CLI 17.5+NTR 1							
Median (n=2)	63.71	69.125	71.355	31.835	64.275	61.95	45.48
Max	64.64	72.23	75.07	33.25	66.39	63.74	45.54
Min	62.78	66.02	67.64	30.42	62.16	60.16	45.42
TMP 0.07+ERY 18+FOX 0.9							
Median (n=2)	65.145	78.595	70.645	28.79	13.1	65.225	16.14
Max	65.65	83.75	79.96	48.62	20.7	67.75	31.48
Min	64.64	73.44	61.33	8.96	5.5	62.7	0.8
TMP 0.07+ERY 18+STR 2.3							

Median (n=3)	71.5	89.7	67.2	64.5	0.9	59.9	37.1
Max	72.7	89.73	69.58	69.1	0.94	66	39.8
Min	68.28	74.9	66.2	64.31	0.5	59.3	30.23
TMP 0.07+ERY 18+TOB 0.6							
Median (n=2)	66.965	86.74	69.815	36.635	0.75	72.33	29.945
Max	68.28	89.73	75.19	64.31	0.79	74.34	37
Min	65.65	83.75	64.44	8.96	0.71	70.32	22.89
TMP 0.08+ERY 18+NTR 1							
Median (n=2)	67.325	80.25	57.25	5.1	31.78	46.375	1.495
Max	69	83.75	62.61	8.96	46.91	54.51	1.71
Min	65.65	76.75	51.89	1.24	16.65	38.24	1.28
TMP 0.08+FOX 0.9+STR 2.3							
Median (n=2)	63.535	85.85	80.905	27.76	28.55	21.735	2.525
Max	66.4	92.74	89.81	31.46	31.29	30.55	3.91
Min	60.67	78.96	72	24.06	25.81	12.92	1.14
TMP 0.07+FOX 0.9+TOB 0.6							
Median (n=3)	69.02	75.22	63.11	0.79	23.84	37.43	0.92
Max	71.17	77.35	75.19	19.3	40.18	57.54	1.14
Min	68.28	72.62	60.97	0.67	17.33	35.39	0.89

TMP 0.08+NTR 1+FOX 0.9							
Median (n=3)	66.4	78.25	82.82	31.46	69.9	85.42	40.95
Max	77.27	88.21	92.74	89.07	81.46	92.77	75.27
Min	60.67	70.54	78.96	24.06	49.99	60.77	18.8
TMP 0.08+TOB 0.6+NTR 1							
Median (n=3)	68.7	68.38	67.64	16.65	1.52	10	1.48
Max	69	77.8	81.4	62.16	73.9	12.26	1.49
Min	64.64	60.17	62.61	0.3	1.25	1.22	0.5
TMP 0.07+STR 2.3+TOB 0.6							
Median (n=3)	68.28	67.2	64.44	0.71	0.73	0.86	0.91
Max	72.7	69.58	75.19	0.94	0.79	1.72	2.2
Min	65.65	60.76	63.7	0.5	0.5	0.6	0.83
TMP 0.08+STR 2.3+NTR 1							
Median (n=2)	63.535	80.905	74.395	75.68	28.55	31.97	8.85
Max	66.4	89.81	78.25	81.46	31.29	37.51	12.51
Min	60.67	72	70.54	69.9	25.81	26.43	5.19
VAN 75+CPR 0.01+ERY 30							
Median (n=3)	83.31	88.08	89.03	61.05	79.1	81.37	36.53
Max	93.13	92.27	95.02	62.8	82.63	88.38	40.69

Min	75.25	86.41	76.46	56.32	59.2	73.69	30.86
VAN 75+CPR 0.01+GEN 0.4							
Median (n=3)	83.31	88.08	79.17	61.05	70.24	63.05	8.31
Max	93.13	92.27	81.79	62.8	82.07	67.18	11.88
Min	75.25	86.41	71.96	56.32	39.34	61.08	7.35
VAN 75+CPR 0.01+FOX 1							
Median (n=3)	83.31	88.08	76.77	61.05	67.13	58.5	18.83
Max	93.13	92.27	78.12	62.8	75.85	58.97	28.93
Min	75.25	86.41	64.02	56.32	58.46	47.92	13.64
VAN 75+CPR 0.01+TOB 0.5							
Median (n=3)	83.31	88.08	82.74	61.05	78.46	76.33	22.64
Max	93.13	92.27	87.96	62.8	84.88	79.24	34.21
Min	75.25	86.41	76.12	56.32	60.24	76.23	11.98
VAN 75+CPR 0.01+STR 2							
Median (n=3)	83.31	88.08	72.97	61.05	63.44	74.71	12.11
Max	93.13	92.27	77.72	62.8	72.16	75.51	15.7
Min	75.25	86.41	68.29	56.32	55.94	68.41	8
VAN 75+CPR 0.01+CLI 20							
Median (n=7)	90.4	85.92	77.38	43.5	42.69	45.66	0.97

Max	93.83	95.68	84.43	67.74	52.86	55.32	2.22
Min	73.95	78.09	69.23	22.21	38.05	19.78	0.5
VAN 75+STR 2+TOB 0.5							
Median (n=3)	79.05	50	86.51	26.68	61.11	43.46	15.56
Max	90.4	50.91	86.65	34	61.18	45.18	22.27
Min	73.95	44.72	82.04	26.01	57.1	37.64	11.25
VAN 75+STR 2+FOX 1							
Median (n=3)	79.05	50	73.09	26.68	61.61	9.54	6.97
Max	90.4	50.91	74.16	34	71.1	9.77	14.5
Min	73.95	44.72	65.75	26.01	57.77	9.48	5.18
VAN 75+STR 2+ERY 30							
Median (n=5)	79.46	71.45	87.15	41.13	56.19	70.21	50.71
Max	93.83	78.13	93.06	54.38	73.75	90.16	68.49
Min	76.53	61.03	83.26	27.3	40.4	45.21	24.62
VAN 75+STR 2+CLI 20							
Median (n=3)	79.05	50	75.34	26.68	47.37	53.71	15.26
Max	90.4	50.91	81.55	34	47.78	60.68	15.79
Min	73.95	44.72	69.23	26.01	42.67	51.64	12.73
VAN 75+AMP 1+CPR 0.01							

Median (n=5)	91.58	82.11	93.16	54.97	41.14	49.22	11.91
Max	93.83	87.96	95.68	63.28	67.74	60.23	16.16
Min	78.65	77.07	84.45	34.75	11.32	34.8	2.23
VAN 75+AMP 1+STR 2							
Median (n=4)	79.055	78.485	72.19	55.4	45.39	23.965	19.725
Max	91.58	87.96	78.13	66.67	54.38	25.99	35.46
Min	76.53	67.09	61.03	34.75	29.1	13.23	14
VAN 75+AMP 1+ERY 30							
Median (n=4)	85.52	83.75	88.145	51.245	49.85	39.275	12.78
Max	93.83	87.96	93.06	63.28	57.95	46.61	22.14
Min	78.65	77.07	84.78	34.75	40.4	31.04	9.67
VAN 75+GEN 0.4+AMP 1							
Median (n=5)	87.2	83.5	85.7	49.73	43.96	26.33	21.94
Max	91.13	85.92	90.19	52.8	55.46	39.7	34.76
Min	78.65	71.8	77.07	29.08	34.75	15.52	19.17
VAN 75+CHL 50+AMP 1							
Median (n=4)	89.165	86.27	87.085	75.59	44.71	52.815	11.685
Max	95.78	93.65	90.19	78.69	64.82	66.91	22.05
Min	77.02	80.27	78.51	65.33	43.96	46.65	5.49

VAN 75+CHL 50+NTR 1							
Median (n=2)	79.015	86.09	86.73	60.045	57.535	73.08	43.055
Max	81.01	88.77	89.97	65.33	60.19	75.39	43.95
Min	77.02	83.41	83.49	54.76	54.88	70.77	42.16
VAN 75+CHL 50+CPR 0.01							
Median (n=4)	89.165	86.27	89.975	75.59	50.505	84.23	30.34
Max	95.78	93.65	91.85	78.69	52.75	90.5	38.09
Min	77.02	80.27	88.44	65.33	47.56	81.25	22.54
VAN 75+CHL 50+STR 2							
Median (n=2)	79.015	86.09	71.18	60.045	39.64	71.135	42.335
Max	81.01	88.77	73.39	65.33	41.99	74.24	43.42
Min	77.02	83.41	68.97	54.76	37.29	68.03	41.25
VAN 75+CHL 50+CLI 20							
Median (n=3)	91.13	83.77	84.4	77.88	45.38	77.97	64.22
Max	95.78	93.65	86.5	78.69	50.9	79.24	68.19
Min	87.2	80.27	78.73	73.3	43.17	77.32	58.27
FUS 80+CPR 0.01+FOX 1							
Median (n=8)	67.565	84.81	68.725	56.495	30.345	56.99	19.295
Max	71.17	93.27	86.45	66.65	47.21	72.42	36.46

Min	50.14	77.19	63.14	50.21	17.32	28.07	4.05
FUS 80+CPR 0.01+TOB 0.5							
Median (n=8)	67.565	84.81	72.78	56.495	28.795	65.66	1.365
Max	71.17	93.27	78.55	66.65	41.31	78.27	17.28
Min	50.14	77.19	59.75	50.21	8.63	43.17	0.44
FUS 80+STR 1+FOX 1							
Median (n=3)	67.74	72.67	76.17	30.18	51.36	52.67	14.22
Max	69.25	87.74	86.45	30.51	52.32	53.13	15.52
Min	62.22	72.24	75.32	25.08	47.52	48.59	9.09
FUS 80+STR 1+TOB 0.5							
Median (n=8)	67.565	67.63	72.78	28.795	44.795	42.275	8.1
Max	71.17	87.74	78.55	41.31	52.54	59.98	37.61
Min	50.14	59.17	59.75	8.63	31.95	30.28	0.46
FUS 80+AMP 1.2+FOX 1							
Median (n=7)	67.74	72.16	72.75	30.51	0.78	20.71	0.7
Max	71.17	87.62	86.45	47.21	12.67	29.43	9.56
Min	55.47	52.04	63.29	17.32	0.49	18.31	0.43
FUS 80+GEN 0.04+CHL 40							
Median (n=4)	90.215	81.415	84.265	50.175	62.03	71.31	43.215

Max	94.66	83.67	92.12	67.35	67.29	76.49	48.93
Min	86.78	80.57	75.65	28.45	54.69	65.53	35.9
FUS 80+GEN 0.04+ERY 30							
Median (n=4)	88.9	81.415	79.145	49.615	38.555	60.695	39.145
Max	94.66	83.67	89.67	67.35	51.79	79.34	41.65
Min	86.78	78.27	58.03	28.45	18.81	59.67	15.18
FUS 80+GEN 0.04+AMP 1.2							
Median (n=4)	90.215	81.415	70.81	50.175	36.495	23.62	1.48
Max	94.66	83.67	82.54	67.35	48.04	74.75	4.4
Min	86.78	80.57	52.42	28.45	30.13	7.27	0.07
FUS 80+CHL 40+ERY 30							
Median (n=4)	90.215	84.265	80.21	38.555	62.03	53.5	34.305
Max	94.66	92.12	89.67	53.31	67.29	59.55	53.72
Min	86.78	75.65	58.03	18.81	54.69	49.09	27.28
FUS 80+CHL 40+AMP 1.2							
Median (n=4)	90.215	81.595	70.81	62.03	36.495	41.2	6.39
Max	94.66	85.91	82.54	67.29	48.04	43.48	13.85
Min	86.78	75.65	52.42	54.69	30.13	33.99	4.1
FUS 80+ERY 30+AMP 1.2							

Median (n=4)	90.215	87.935	70.81	38.555	36.495	27.58	2.845
Max	94.66	92.12	82.54	53.31	48.04	36.36	9.75
Min	86.78	58.03	52.42	18.81	30.13	23.36	-0.01
FUS 80+ERY 30+TOB 0.5							
Median (n=5)	67.39	80.23	67.59	19.4	38.72	75.22	11.06
Max	71.17	90.04	77.97	31.75	57.88	78.84	48.39
Min	55.47	68.44	61.02	8.63	36.47	65.94	4.57
FUS 80+GEN 0.4+CPR 0.01							
Median (n=9)	78.98	77.99	83.1	49.71	60.3	55.98	1.13
Max	89.87	88.74	96.25	66.93	74.73	72.47	9.08
Min	63.45	63.84	76.9	0.8	19	40.68	0.5
FUS 80+CPR 0.01+STR 1							
Median (n=5)	76.38	85.22	68.1	62.68	49.4	52.11	6.5
Max	85.51	90.6	74.61	66.73	53.16	56.26	20.45
Min	69.56	79.61	62.59	52.2	44.16	18.94	1.12
GEN 0.4+ERY 20+STR 2							
Median (n=3)	78.87	81.38	75.33	78.1	2.19	56.77	2.97
Max	83.78	82.2	81.02	80.6	47.31	67.06	6.87
Min	71.73	68.43	66.62	77.64	0.51	49.36	1.61

GEN 0.4+ERY 20+CHL 40							
Median (n=3)	77.16	79.46	84.55	81.61	78.22	73.42	73.03
Max	83.04	92.75	87.57	85.66	84.58	85.41	75.22
Min	70.13	75.11	77.84	72.48	69.49	60.98	67.14
GEN 0.4+STR 2+FOX 1.2							
Median (n=4)	83.21	72.82	82.58	76.57	57.805	60.46	42.675
Max	87.47	76.33	92.5	81.17	71.75	62.32	50.44
Min	77.16	68.35	72.78	61.24	40.88	43.13	24.96
GEN 0.4+NTR 1.2+TOB 0.5							
Median (n=2)	73.2	90.455	72.55	47.36	65.47	32.8	0.96
Max	77.52	90.92	75.36	63.5	79.37	56.38	1.05
Min	68.88	89.99	69.74	31.22	51.57	9.22	0.87
GEN 0.4+STR 2+CHL 40							
Median (n=3)	77.16	71.26	84.55	74.21	78.22	81.89	74.34
Max	83.04	82.06	87.57	75.02	84.58	88.46	80.64
Min	70.13	68.35	77.84	61.24	69.49	72.27	62.5
GEN 0.4+CHL 40+TOB 0.5							
Median (n=3)	77.52	69.19	70.99	75.99	67.02	66.91	61.72
Max	77.54	82.84	75.36	78.95	79.37	76.2	62.41

Min	68.88	68.78	69.74	57.45	51.57	59.72	46.4
GEN 0.4+STR 1.8+TOB 0.5							
Median (n=5)	77.87	68.49	80.1	27.32	63.02	45.74	5.58
Max	82.5	74.48	85.31	42.03	70.82	54.2	12.53
Min	71.74	60.23	72.21	16.56	51.1	34.64	0.81
GEN 0.4+CPR 0.012+STR 1.8							
Median (n=3)	82.5	80.97	70.78	1.14	16.56	0.82	1.75
Max	82.5	80.97	70.78	1.14	16.56	0.82	1.75
Min	82.5	80.97	70.78	1.14	16.56	0.49	0.47
GEN 0.4+TMP 0.075+STR 1.8							
Median (n=3)	82.5	83.13	70.78	6.61	16.56	9.57	1.26
Max	82.5	83.13	70.78	6.61	16.56	9.57	1.26
Min	82.5	83.13	70.78	2.89	16.56	2.82	0.56
GEN 0.4+TMP 0.075+ERY 50							
Median (n=3)	82.5	83.13	59.58	6.61	42.18	2.57	0.71
Max	82.5	83.13	62.36	6.61	44.96	8.1	0.72
Min	82.5	83.13	59.58	2.89	42.18	2.57	0.71
GEN 0.4+TMP 0.075+TOB 0.4							
Median (n=3)	82.5	83.13	85.31	6.61	70.82	3.09	0.94

Max	82.5	83.13	85.31	6.61	70.82	20.69	0.94
Min	82.5	83.13	85.31	2.89	70.82	3.09	0.5
FUS 80+NTR 1.5+CPR 0.01							
Median (n=3)	78.98	77.3	86.71	77.68	62.97	64.71	59.02
Max	89.87	90.8	88.74	87.34	66.93	70.22	62.54
Min	63.45	75.38	63.84	75.68	55.87	56.66	48.3
FUS 80+NTR 1.5+STR 2							
Median (n=2)	84.425	84.05	62.075	82.51	44.89	5.675	7.095
Max	89.87	90.8	64.99	87.34	46.87	9.21	11.67
Min	78.98	77.3	59.16	77.68	42.91	2.14	2.52
FUS 80+NTR 1.5+GEN 0.4							
Median (n=3)	78.98	77.3	77.33	77.68	34.63	25.27	1.14
Max	89.87	90.8	83.12	87.34	40.68	33.5	1.82
Min	63.45	75.38	76.95	75.68	19	24.03	0.88
FUS 80+STR 2+GEN 0.4							
Median (n=3)	78.98	61.06	77.3	46	34.63	43.87	0.93
Max	89.87	64.99	83.1	46.87	40.68	49.97	2.13
Min	63.45	59.16	76.9	42.91	19	37.62	0.89
GEN 0.4+CHL 70+CPR 0.012							

Median (n=3)	79.9	88.8	86.5	71.1	36.9	68.7	55.2
Max	91.9	90.1	87.5	74.7	43.6	72.1	56.8
Min	78.9	86.5	83.5	69.7	33.4	64	48.5
DOX 0.65+GEN 0.4+FOX 1							
Median (n=4)	80.785	82.855	67.635	58.47	54.8	21.825	31.575
Max	92.57	89.19	76.96	70.28	62.97	30.73	44.68
Min	72.48	81.26	64.39	47.46	53.5	14.75	15.38
DOX 0.65+GEN 0.4+TOB 0.5							
Median (n=4)	85.235	81.84	89.095	66.92	71.36	70.5	52.8
Max	92.57	83.29	94	70.28	87.79	77.3	59.18
Min	72.48	78.56	85.9	47.46	62.57	62.42	2.43
DOX 0.65+GEN 0.4+STR 2							
Median (n=4)	79.81	81.84	79.815	56.54	65.435	56.46	43.115
Max	92.57	89.19	89.31	70.28	78.97	57.35	54.32
Min	72.48	78.56	71.59	47.46	54.91	48.87	10.94
DOX 0.65+GEN 0.4+CPR 0.012							
Median (n=4)	85.235	81.84	85.11	66.92	82.555	73.58	58.525
Max	92.57	83.29	89	70.28	91.36	76.54	67.54

Min	72.48	78.56	76.86	47.46	48.99	62.31	5.65
DOX 0.65+GEN 0.4+NTR 2							
Median (n=5)	87.4	83.27	86.4	59.46	83.78	49.78	38.46
Max	92.83	90.86	90.2	75.12	92.37	64.69	56.53
Min	82.31	73.28	81.43	58.4	76.44	40.54	26.81
DOX 0.65+FOX 1+TOB 0.5							
Median (n=3)	86.21	64.39	88.68	55.29	72.07	52.09	54.83
Max	92.57	65.1	89.51	62.97	87.79	54.09	56.79
Min	84.26	58.15	85.9	53.95	70.65	51.67	48.29
DOX 0.65+FOX 1+STR 2							
Median (n=4)	83.25	60.285	85.845	41.625	69.59	30.665	30.84
Max	92.78	63.7	86.69	52.45	74.35	38.39	34.21
Min	77.76	51.19	71.37	35.4	60.56	11.83	22.65
DOX 0.65+FOX 1+CPR 0.012							
Median (n=3)	82.31	59.29	90.78	44.66	44.55	19.26	28.93
Max	85.64	63.7	93.99	47.48	65.9	20.08	30.29
Min	77.76	57.48	79.82	35.4	32.75	12.02	24.13
DOX 0.65+STR 2+CPR 0.012							
Median (n=3)	87.4	85.58	91.92	60.56	68.14	68.81	51.18

Max	92.78	86.11	93.99	73.77	75.77	74.58	69.01
Min	82.31	71.21	89.53	50.01	65.9	27.56	30.12
DOX 0.65+TOB 0.5+CPR 0.012							
Median (n=4)	86.585	93.155	92.955	83.87	70.835	84.51	75.54
Max	92.78	94.12	95.28	87.22	76.81	87.75	82.47
Min	82.31	88.39	91.02	67.62	39.61	81.43	18.55
DOX 0.65+FOX 1+NTR 2							
Median (n=2)	90.94	94.015	93.57	111.795	99.59	95.245	67.745
Max	92.9	94.24	95.19	114.64	104.97	96.98	69.08
Min	88.98	93.79	91.95	108.95	94.21	93.51	66.41
DOX 0.6+NTR 2+ERY 40							
Median (n=4)	74.375	87.78	76.03	79.345	20.14	63.685	30.015
Max	81.66	91.1	85.75	89.9	43.47	70.07	49.13
Min	66.7	81.26	67.5	73.97	0.6	45.9	8.2
DOX 0.6+NTR 2+TMP 0.08							
Median (n=4)	63.4	85.8	63.7	76.1	50.2	0.5	0.35
Max	69.3	91.1	80.6	89.9	61.6	1.3	0.8
Min	57.6	82.6	56.8	69.1	41.1	0.2	0.2
DOX 0.65+NTR 2+CLI 20							

Median (n=2)	81.665	88.26	90.715	76.495	60.455	45.565	47.345
Max	83.88	95.26	94.36	79.02	66.51	48.17	51.68
Min	79.45	81.26	87.07	73.97	54.4	42.96	43.01
DOX 0.65+FUS 110+AMP 1.2							
Median (n=3)	87.31	81.66	72.39	47.58	49.27	25.48	4.98
Max	92.85	87.36	76.48	49.89	51.55	30.84	8.81
Min	81.77	75.96	68.29	45.27	46.99	20.12	1.16
DOX 0.65+TMP 0.07+ERY 60							
Median (n=2)	82.0279	52.9497	83.4541	57.9604	80.516	14.9225	0.91359
Max	96.2638	62.1698	85.487	61.3172	94.1689	16.9787	0.99962
Min	67.792	43.7296	81.4211	54.6037	66.8631	12.8663	0.82757
DOX 0.65+AMP 1.2+ERY 60							
Median (n=3)	87.31	72.39	63.97	49.27	7.8	5.27	3.56
Max	92.85	76.48	69.89	51.55	7.9	5.28	4.2
Min	81.77	68.29	58.05	46.99	7.7	5.27	2.92
DOX 0.65+TMP 0.07+AMP 1.2							
Median (n=2)	82.0279	66.1454	52.9497	45.8473	57.9604	1.2951	0.77656
Max	96.2638	71.0641	62.1698	52.5685	61.3172	1.8069	0.79723
Min	67.792	61.2267	43.7296	39.126	54.6037	0.78336	0.75589

DOX 0.5+CHL 50+CPR 0.01							
Median (n=5)	75.9	81.9	90.4	56.5	67.7	74.3	47.6
Max	83.2	85.6	94.9	59.3	69.8	79.6	50.6
Min	68.6	76	89.5	53.5	61.3	71.5	46.6
DOX 0.5+CHL 50+NTR 1							
Median (n=5)	75.9	81.9	90.9	56.5	76.8	70.8	55
Max	83.2	85.6	95.2	59.3	82.5	73	56.5
Min	68.6	76	87.4	53.5	74.1	62.4	50.4
DOX 0.5+CHL 50+AMP 1							
Median (n=5)	75.9	81.9	80.7	56.5	20.4	24.1	8.2
Max	83.2	85.6	85.7	59.3	26.5	40.9	12
Min	68.6	76	80.2	53.5	13.6	19.5	6.2
DOX 0.5+CHL 50+ERY 30							
Median (n=5)	75.9	81.9	82.7	56.5	69.2	67.5	25.4
Max	83.2	85.6	86.4	59.3	71.3	75.6	27.9
Min	68.6	76	75.7	53.5	61.2	61.4	24.7
DOX 0.5+CHL 50+CLI 20							
Median (n=5)	75.9	81.9	72.5	56.5	67.3	69.4	47
Max	83.2	85.6	78.3	59.3	73.6	75.9	52.6

Min	68.6	76	69.7	53.5	62.4	63.9	43.7
DOX 0.5+CHL 50+TMP 0.07							
Median (n=3)	67.6	63.3	78.5	46.6	54	67	52.1
Max	70.4	66.1	90.9	47.8	57.8	78.4	52.2
Min	63	63	77.6	45.9	52.6	65.6	49.4
DOX 0.5+CHL 50+FOX 1							
Median (n=4)	65.3	64.7	77.05	47.2	47.8	13.4	18.1
Max	70.4	66.9	79.5	48.2	52.8	21.7	21.2
Min	62.4	63	66.2	45.9	42.5	8.7	12.2
DOX 0.5+CHL 50+TOB 0.5							
Median (n=4)	65.3	64.7	81.75	47.2	59.05	65.9	50.45
Max	70.4	66.9	87.1	48.2	64.1	67.4	50.6
Min	62.4	63	79.2	45.9	50.5	61.6	49.1
DOX 0.5+CHL 50+STR 2							
Median (n=3)	63	63.3	83.6	47.8	54.5	65.6	47.5
Max	70.4	66.9	85.2	48.2	57	70.5	47.7
Min	62.4	63	78	46.6	53.7	63	47
DOX 0.5+TOB 0.5+STR 2							
Median (n=3)	63	83.6	83.6	63	54.5	77.8	51.9

Max	70.4	87.1	85.2	64.1	57	80.4	56.2
Min	62.4	79.2	78	55.1	53.7	70.5	47.5
DOX 0.5+CPR 0.01+NTR 1							
Median (n=3)	78.59	92.16	62.77	59.28	84.58	2.76	6.96
Max	79.18	96.98	68.71	72.02	86.31	3.92	17.96
Min	77.42	89.9	60.59	46.33	78.64	0.88	4.62
DOX 0.65+CPR 0.015+AMP 1.2							
Median (n=3)	75.5	64.7	85	43.8	12.5	15.7	0.1
Max	84.2	79.4	94	46	22.4	18.6	0.1
Min	74	60.4	67.6	38	0	15.4	0
DOX 0.5+CPR 0.01+ERY 30							
Median (n=3)	71.7	93.3	74.6	64.5	49	76.5	50.2
Max	73.8	93.6	80.9	65.6	49.3	84.9	50.8
Min	71.6	86.2	65.8	62.7	46.4	71.1	46.5
DOX 0.5+CPR 0.01+CLI 20							
Median (n=3)	71.7	93.3	77.3	64.5	58.9	51.7	44.9
Max	73.8	93.6	79.4	65.6	60.9	54.4	46.4
Min	71.6	86.2	71.6	62.7	56.3	51.5	42.6

DOX 0.6+NTR 2+AMP 1.2							
Median (n=5)	79	62.8	73.4	86.3	9.9	16.4	17.2
Max	90.3	82.2	92.7	89.7	17.3	24.4	20.3
Min	77.4	60.6	66.2	78.6	9.6	13.1	13.9
DOX 0.5+GEN 0.4+TMP 0.07							
Median (n=4)	62.8	76.5	66.4	42.9	40.65	36.5	3.45
Max	64	81.3	70	51.2	47	49	7.1
Min	58.6	73.5	58.7	34.3	39.1	12.7	0.9
DOX 0.5+GEN 0.4+CHL 50							
Median (n=4)	62.8	76.5	65.55	42.9	41.1	66.9	37.7
Max	64	81.3	70.2	51.2	42.9	68.4	42.6
Min	58.6	73.5	61.7	34.3	33.4	65	30
DOX 0.5+GEN 0.4+AMP 1.2							
Median (n=3)	62	79.4	82.3	38	14.3	23.9	1.2
Max	64	81.3	82.4	51.2	28.3	24.2	1.6
Min	58.6	73.5	76.7	34.3	12.8	21.6	0.7
DOX 0.5+GEN 0.4+ERY 30							
Median (n=4)	62.8	76.5	54.75	42.9	35.35	57.65	18
Max	64	81.3	59.4	51.2	41.5	61.6	32.4

Min	58.6	73.5	51.7	34.3	17.9	55.7	4.3
DOX 0.5+GEN 0.4+CLI 20							
Median (n=4)	62.8	76.5	67.65	42.9	55	65.85	49.65
Max	64	81.3	70.9	51.2	56.9	67.8	51.9
Min	58.6	73.5	63.1	34.3	52.8	62.9	45.5
DOX 0.4+STR 2+NTR 1.5							
Median (n=5)	83.7	72.6	79.3	66.1	74.6	37.9	44.1
Max	85.2	73.9	82.4	67.6	78.5	42.4	50
Min	76.7	68.4	77.1	61.2	72.6	4	40.9
DOX 0.4+STR 2+AMP 1.2							
Median (n=5)	83.7	72.6	67	66.1	21.4	26.5	1.9
Max	85.2	73.9	68.3	67.6	30	28.9	10.1
Min	76.7	68.4	59.6	61.2	12.8	16.1	1
DOX 0.4+STR 2+ERY 30							
Median (n=5)	83.7	72.6	89.1	66.1	75.4	82	67.8
Max	85.2	73.9	91.9	67.6	86.3	85.3	78.2
Min	76.7	68.4	86.4	61.2	68.5	79.2	63.6
DOX 0.4+STR 2+CLI 20							
Median (n=5)	83.7	72.6	70.9	66.1	72.9	68.4	64.8

Max	85.2	73.9	72.1	67.6	81.4	71.4	71.8
Min	76.7	68.4	68.1	61.2	65.6	66.8	63
DOX 0.4+ERY 30+CLI 20							
Median (n=5)	83.7	89.1	70.9	75.4	72.9	67	61.5
Max	85.2	91.9	72.1	86.3	81.4	67.5	69.8
Min	76.7	86.4	68.1	68.5	65.6	66.2	55.2
DOX 0.4+FOX 1+AMP 1.2							
Median (n=4)	84.25	79.9	77.25	74.95	54.95	23.65	1
Max	91.4	88.3	86.8	89.3	57	31.8	4.6
Min	75.3	70.7	74.9	64.8	44.4	15.8	0.8
DOX 0.4+FOX 1+ERY 30							
Median (n=3)	87.3	83.7	65.5	82.3	53.3	17	17.8
Max	91.4	88.3	73.9	89.3	56.2	23.7	20.5
Min	81.2	76.1	62.4	64.8	47.8	17	12.6
DOX 0.4+FOX 1+CLI 20							
Median (n=4)	84.25	79.9	75.05	74.95	68.95	19	14.85
Max	91.4	88.3	77.5	89.3	75.5	25.1	27.6
Min	75.3	70.7	73.4	64.8	65.4	10.3	10.2
DOX 0.65+TMP 0.08+FOX 1							

Median (n=3)	64.4	78.9	66.7	41.8	28.4	21.8	26.4
Max	68	79.3	67.2	53.9	40.6	23.9	30.5
Min	60.9	69	62.2	25.5	21.8	18.6	11.8
DOX 0.65+TMP 0.08+CLI 20							
Median (n=3)	64.4	78.9	74.4	41.8	58.7	4.3	0.2
Max	68	79.3	81.7	53.9	61	22.8	2.7
Min	60.9	69	74.2	25.5	38.1	0.1	0.1
DOX 0.4+FUS 80+CHL 50							
Median (n=3)	88.4	91.1	89.2	56.3	71.6	58.1	32.5
Max	89.4	94.4	92.5	65.5	77.5	69.6	35.6
Min	82.6	89.6	84.1	53.5	62.7	52.3	21.6
DOX 0.4+FUS 80+FOX 1							
Median (n=4)	88.9	92.65	63.4	60.25	45.95	45.2	16.5
Max	93.8	94.4	64.9	65.5	49.6	47.9	23.5
Min	81.2	91.1	61.7	56.3	39.6	38	9.8
DOX 0.4+FUS 80+TMP 0.07							
Median (n=6)	87.3	90.85	62.25	57.25	62.9	63.4	34.45
Max	93.8	94.4	86.8	65.5	72.6	81.4	51.3
Min	81.2	86.8	56.5	52.2	29.5	49.7	13.8

DOX 0.4+FUS 80+TOB 0.5							
Median (n=2)	86	90.35	88	54.9	69.8	67.45	30.75
Max	89.4	91.1	88.3	56.3	74.2	68.6	34.9
Min	82.6	89.6	87.7	53.5	65.4	66.3	26.6
DOX 0.65+TMP 0.08+TOB 0.5							
Median (n=5)	68	78.9	81	41.8	43.4	30	7.4
Max	93.8	80.6	87.4	78.4	66.6	42.1	16.2
Min	60.9	56.5	79.6	25.5	37.2	17.3	0.4
DOX 0.65+VAN 35+FUS 110							
Median (n=4)	65	96.15	83.4	9.95	11.3	52.9	0.75
Max	75.7	116.1	94.1	12.5	13.4	65.2	0.8
Min	55.9	91.7	79	0.9	9.5	39.5	0.6
DOX 0.5+VAN 35+GEN 0.4							
Median (n=5)	87.7	111	88.4	49.8	34.9	62.5	1.3
Max	96	115.7	92.6	59.6	40.5	80.9	9.1
Min	83.8	106.6	81	30.9	24.3	55.3	0.7
DOX 0.65+VAN 35+CHL 70							
Median (n=4)	69.35	108.3	89.9	11.2	31.8	46.3	9.6
Max	75.7	120.5	96.9	14	40.2	91.1	11.6

Min	62.1	91.8	82	0.9	22.5	38	0.7
DOX 0.5+VAN 35+FOX							
Median (n=5)	87.7	111	67.2	49.8	44.2	74.5	30.7
Max	96	115.7	71.5	59.6	50	80.3	46.7
Min	83.8	106.6	60.4	30.9	37.2	70.3	7.5
DOX 0.65+FUS 110+GEN 0.4							
Median (n=4)	65	83.4	81.95	11.3	14.7	12.85	1
Max	75.7	94.1	84.7	13.4	37.8	31.9	1.2
Min	55.9	79	78.8	9.5	11.3	2.9	0.8
DOX 0.7+TOB 0.5+NTR 3							
Median (n=5)	67.7	84.1	67	62.2	67.7	34	42.6
Max	72.6	87.3	82.1	64.8	68.7	39.3	44.5
Min	64.8	76.2	62	59.8	61.7	30	39.6
DOX 0.7+TOB 0.5+AMP 1.2							
Median (n=5)	67.7	84.1	68.9	62.2	28.8	16.7	15.9
Max	72.6	87.3	73.3	64.8	31.6	19.5	23.8
Min	64.8	76.2	67.4	59.8	23.4	13.1	6.7
DOX 0.7+TOB 0.5+ERY 60							
Median (n=5)	67.7	84.1	86.4	62.2	8.9	78.9	7.7

Max	72.6	87.3	89.5	64.8	16	81.5	12.2
Min	64.8	76.2	73.8	59.8	5.7	66.5	6.8
DOX 0.7+TOB 0.5+CLI 20							
Median (n=5)	67.7	84.1	72.1	62.2	59.1	75.5	65.3
Max	72.6	87.3	75.1	64.8	62.4	78.4	66.3
Min	64.8	76.2	67.6	59.8	55.2	70.9	60.9
DOX 0.7+AMP 1.2+CLI 20							
Median (n=5)	67.7	68.9	72.1	28.8	59.1	18.3	5.6
Max	72.6	73.3	75.1	31.6	62.4	23.9	13.6
Min	64.8	67.4	67.6	23.4	55.2	13.6	4.4
DOX 0.6+VAN 35+NTR 2							
Median (n=5)	79	109.2	62.8	12.6	86.3	3.4	7.6
Max	90.3	119.1	82.2	41.6	89.7	7.6	14.2
Min	77.4	103.3	60.6	0.6	78.6	1.2	1.4
DOX 0.6+VAN 35+AMP 1.2							
Median (n=3)	90.6	110.2	93.6	30.1	38	94.6	13
Max	94.5	112	95	37.5	39.5	98.8	17.3
Min	85.5	109.1	90.2	29.1	18.5	87	8.8
DOX 0.6+VAN 35+CLI 20							

Median (n=3)	90.6	109.1	90.5	30.1	72.6	76.6	52.9
Max	91	110.2	93.8	33.5	79.4	86.4	55.7
Min	85.5	107.9	86.7	29.1	67.7	75.7	52.4
DOX 0.65+TMP 0.1+CPR 0.015							
Median (n=2)	79.85	70.35	72.05	40.9	40.45	0.05	3.5
Max	84.2	76.7	79.4	43.8	61.9	0.1	5.8
Min	75.5	64	64.7	38	19	0	1.2
DOX 0.65+TMP 0.1+STR 1.5							
Median (n=4)	78.95	70.35	75.95	56.6	52.45	2.55	41.85
Max	84.2	93.8	83.4	74.5	54	13.4	48.9
Min	75.5	61.6	74.2	19	49.3	0.3	27.6
DOX 0.65+FUS 110+NTR 2.8							
Median (n=5)	79	68.4	62.8	5.1	86.3	33.5	29.8
Max	90.3	82.9	82.2	13.1	89.7	40.5	35.1
Min	77.4	63.6	60.6	3.4	78.6	20.1	17.2
DOX 0.65+VAN 35+ERY 30							
Median (n=5)	70.2	102.2	112.7	7.5	5.3	59.4	16.1
Max	74.2	107.4	121	34.6	21.9	62.3	19.1

Min	65	93.7	111	3.2	3.4	32.3	7.5
DOX 0.65+VAN 35+TMP 0.08							
Median (n=4)	69.6	102.15	64.15	20.75	23.55	0.15	0.2
Max	74.2	107.4	68.5	34.6	33.7	0.4	0.4
Min	65	93.7	62	3.2	13.1	0.1	0
DOX 0.65+VAN 35+CPR 0.012							
Median (n=3)	71.2	100.3	85.9	34	49.7	32.9	0.2
Max	74.2	107.4	94.3	34.6	59.9	34.1	0.2
Min	65	93.7	84.1	7.5	44.4	14	0
DOX 0.65+FUS 110+CPR 0.012							
Median (n=3)	71.2	90.2	85.9	11.9	49.7	18.7	0
Max	74.2	90.7	94.3	14.6	59.9	41.2	0.1
Min	65	78.4	84.1	5.3	44.4	7.9	0
DOX 0.6+FUS 110+CLI 20							
Median (n=5)	81	72.9	91	10	66.7	46.3	11.4
Max	99.6	77.6	96.9	11.2	74.6	65.3	13.2
Min	78.2	59.1	79.2	7.2	63.8	44.4	7.8
DOX 0.65+FUS 110+ERY 60							

Median (n=3)	87.3	81.7	64	47.6	7.8	5.3	3.6
Max	92.9	87.4	69.9	49.9	7.9	5.3	4.2
Min	81.8	76	58	45.3	7.7	5.3	2.9
DOX 0.4+VAN 35+TOB 0.5							
Median (n=4)	83.81	101.58	76.935	36.725	56.145	81.285	26.38
Max	93.56	106.55	83.55	79.8	63.66	89.46	49.3
Min	83.25	96.3	71.22	22.45	49.42	68.15	20.76
DOX 0.4+VAN 35+STR 2							
Median (n=3)	83.56	101.51	68.7	39.43	52.72	62.63	9.96
Max	84.06	106.55	74.31	79.8	60.44	66.75	12.77
Min	83.25	96.3	62.49	34.02	50.73	55.31	1.01
DOX 0.4+FUS 80+STR 2							
Median (n=4)	83.81	78.5	66.71	44.455	52.96	44.865	20.595
Max	86.48	89.83	74.31	68.22	60.44	56.87	27.26
Min	83.25	71.27	62.49	34.51	50.73	37.72	16.68
VAN 75+TMP 0.07+AMP 1.2							
Median (n=7)	109.3	70	72.1	1.2	53.4	3.9	1.2
Max	119.6	85.9	115.7	49.8	62	8.2	1.4
Min	104.1	60.7	61	0.9	49.6	1.2	1

***S. epidermidis* 14990**							
AMP 0.3+CHL 120+CLI 0.04							
Median (n=32)	86.85	84.63	99.43	28.79	95.77	63.16	-7.58
Max	152.63	91.82	128.75	72.20	120.33	73.49	1.73
Min	54.14	79.26	85.73	4.35	62.47	49.41	-10.46
AMP 0.3+CHL 120+CPR 0.2							
Median (n=32)	86.85	84.63	78.04	28.79	66.20	46.87	2.67
Max	152.63	91.82	89.38	72.20	73.42	54.46	8.32
Min	54.14	79.26	61.17	4.35	64.16	40.09	-4.62
AMP 0.3+CHL 120+DOX 1							
Median (n=32)	86.85	84.63	95.35	28.79	25.36	14.23	-9.25
Max	152.63	91.82	109.79	72.20	39.29	22.72	0.84
Min	54.14	79.26	83.39	4.35	12.82	8.10	-15.84
AMP 0.3+CHL 120+ERY 0.08							
Median (n=32)	86.85	84.63	75.48	28.79	51.70	63.86	-9.01
Max	152.63	91.82	81.90	72.20	89.47	86.13	51.48
Min	54.14	79.26	71.57	4.35	20.35	39.91	-16.64
AMP 0.3+CHL 120+FOX 0.7							

Median (n=32)	86.85	84.63	75.22	28.79	69.31	55.83	-11.37
Max	152.63	91.82	113.77	72.20	120.28	70.61	-2.31
Min	54.14	79.26	69.35	4.35	61.17	42.49	-16.54
AMP 0.3+CHL 120+FUS 0.02							
Median (n=32)	86.85	84.63	95.69	28.79	86.57	56.79	-6.83
Max	152.63	91.82	131.31	72.20	145.18	64.81	45.41
Min	54.14	79.26	68.66	4.35	79.14	30.44	-14.20
AMP 0.3+CHL 120+GEN 0.25							
Median (n=32)	86.85	84.63	85.43	28.79	86.68	47.99	-10.65
Max	152.63	91.82	104.78	72.20	95.54	58.56	-7.80
Min	54.14	79.26	66.01	4.35	83.72	33.53	-12.71
AMP 0.3+CHL 120+TMP 5							
Median (n=32)	86.85	84.63	55.81	28.79	57.40	50.04	0.89
Max	152.63	91.82	60.53	72.20	67.17	69.42	16.68
Min	54.14	79.26	51.17	4.35	46.24	41.30	-6.55
AMP 0.3+CHL 120+TOB 0.2							
Median (n=32)	86.85	84.63	97.75	28.79	106.89	68.21	1.54
Max	152.63	91.82	136.91	72.20	140.00	81.29	41.88
Min	54.14	79.26	69.31	4.35	83.79	48.32	-7.49

AMP 0.3+CLI 0.04+DOX 1							
Median (n=32)	86.85	99.43	95.36	95.77	25.36	18.98	-3.89
Max	152.63	128.75	109.79	120.33	39.29	22.69	11.92
Min	54.14	85.73	83.39	62.47	12.82	12.25	-14.89
AMP 0.3+CLI 0.04+ERY 0.08							
Median (n=32)	86.85	99.43	75.48	95.77	51.70	100.16	17.20
Max	152.63	128.75	81.90	120.33	89.47	110.96	98.94
Min	54.14	85.73	71.57	62.47	20.35	68.66	8.73
AMP 0.3+CLI 0.04+FOX 0.7							
Median (n=32)	86.85	99.43	75.22	95.77	69.31	67.51	33.47
Max	152.63	128.75	113.77	120.33	120.28	111.52	88.90
Min	54.14	85.73	69.35	62.47	61.17	60.84	17.53
AMP 0.3+CLI 0.04+FUS 0.02							
Median (n=32)	86.85	99.43	95.69	95.77	86.57	94.56	44.05
Max	152.63	128.75	131.31	120.33	145.18	112.05	121.66
Min	54.14	85.73	68.66	62.47	79.14	64.41	35.44
AMP 0.3+CLI 0.04+GEN 0.25							
Median (n=32)	86.85	99.43	85.43	95.77	86.68	-1.32	-10.91
Max	152.63	128.75	104.78	120.33	95.54	9.55	-3.73

Min	54.14	85.73	66.01	62.47	83.72	-8.46	-13.95
AMP 0.3+CLI 0.04+TMP 5							
Median (n=32)	86.85	99.43	55.81	95.77	57.40	46.99	11.55
Max	152.63	128.75	60.53	120.33	67.17	58.92	22.03
Min	54.14	85.73	51.17	62.47	46.24	33.51	6.67
AMP 0.3+CLI 0.04+TOB 0.2							
Median (n=32)	86.85	99.43	97.75	95.77	106.89	90.92	14.68
Max	152.63	128.75	136.91	120.33	140.00	138.17	100.94
Min	54.14	85.73	69.31	62.47	83.79	64.61	5.99
AMP 0.3+CLI 0.04+CPR 0.2							
Median (n=32)	86.85	99.43	78.04	95.77	66.20	49.17	48.96
Max	152.63	128.75	89.38	120.33	73.42	54.56	60.00
Min	54.14	85.73	61.17	62.47	64.16	36.92	38.49
AMP 0.3+CPR 0.2+DOX 1							
Median (n=32)	86.85	78.04	95.36	66.20	25.36	47.05	1.44
Max	152.63	89.38	109.79	73.42	39.29	54.20	12.28
Min	54.14	61.17	83.39	64.16	12.82	44.87	-6.53
AMP 0.3+CPR 0.2+ERY 0.08							
Median (n=32)	86.85	78.04	75.48	66.20	51.70	62.04	24.63

Max	152.63	89.38	81.90	73.42	89.47	66.43	34.71
Min	54.14	61.17	71.57	64.16	20.35	52.16	8.07
AMP 0.3+CPR 0.2+FOX 0.7							
Median (n=32)	86.85	78.04	75.22	66.20	69.31	53.73	38.62
Max	152.63	89.38	113.77	73.42	120.28	63.56	43.07
Min	54.14	61.17	69.35	64.16	61.17	46.84	30.26
AMP 0.3+CPR 0.2+FUS 0.02							
Median (n=32)	86.85	78.04	95.69	66.20	86.57	74.68	62.02
Max	152.63	89.38	131.31	73.42	145.18	87.86	77.31
Min	54.14	61.17	68.66	64.16	79.14	61.49	54.49
AMP 0.3+CPR 0.2+GEN 0.25							
Median (n=32)	86.85	78.04	85.43	66.20	86.68	45.17	21.13
Max	152.63	89.38	104.78	73.42	95.54	54.75	48.68
Min	54.14	61.17	66.01	64.16	83.72	32.60	12.67
AMP 0.3+CPR 0.2+TMP 5							
Median (n=32)	86.85	78.04	55.82	66.20	57.40	39.13	1.72
Max	152.63	89.38	60.53	73.42	67.17	47.03	16.13
Min	54.14	61.17	51.17	64.16	46.24	31.59	-5.91
AMP 0.3+CPR 0.2+TOB 0.2							

Median (n=32)	86.85	78.04	97.75	66.20	106.89	52.41	61.56
Max	152.63	89.38	136.91	73.42	140.00	59.02	67.24
Min	54.14	61.17	69.31	64.16	83.79	45.63	54.01
AMP 0.3+DOX 1+FOX 0.7							
Median (n=32)	86.85	95.36	75.22	25.36	69.31	46.36	-0.68
Max	152.63	109.79	113.77	39.29	120.28	52.62	5.48
Min	54.14	83.39	69.35	12.82	61.17	34.79	-9.12
AMP 0.3+DOX 1+FUS 0.02							
Median (n=32)	86.85	95.36	95.69	25.36	86.57	47.93	13.14
Max	152.63	109.79	131.31	39.29	145.18	68.27	20.17
Min	54.14	83.39	68.66	12.82	79.14	38.52	-13.52
AMP 0.3+DOX 1+GEN 0.25							
Median (n=32)	86.85	95.36	85.43	25.36	86.68	50.80	3.11
Max	152.63	109.79	104.78	39.29	95.54	73.19	11.46
Min	54.14	83.39	66.01	12.82	83.72	29.08	-11.82
AMP 0.3+DOX 1+TMP 5							
Median (n=32)	86.85	95.36	55.82	25.36	57.40	50.31	-2.75
Max	152.63	109.79	60.53	39.29	67.17	73.34	3.78
Min	54.14	83.39	51.17	12.82	46.24	40.37	-7.41

AMP 0.3+DOX 1+TOB 0.2							
Median (n=32)	86.85	95.36	97.75	25.36	106.89	75.77	2.97
Max	152.63	109.79	136.91	39.29	140.00	86.50	11.76
Min	54.14	83.39	69.31	12.82	83.79	72.31	-2.33
AMP 0.3+TMP 5+TOB 0.2							
Median (n=32)	86.85	55.82	97.75	57.40	106.89	59.58	52.99
Max	152.63	60.53	136.91	67.17	140.00	68.60	71.80
Min	54.14	51.17	69.31	46.24	83.79	57.47	3.61
AMP 0.3+ERY 0.08+FOX 0.7							
Median (n=32)	86.85	75.48	75.22	51.70	69.31	82.63	-4.91
Max	152.63	81.90	113.77	89.47	120.28	93.07	84.26
Min	54.14	71.57	69.35	20.35	61.17	53.44	-10.81
AMP 0.3+ERY 0.08+FUS 0.02							
Median (n=32)	86.85	75.48	95.69	51.70	86.57	77.41	-4.68
Max	152.63	81.90	131.31	89.47	145.18	96.39	81.68
Min	54.14	71.57	68.66	20.35	79.14	64.57	-7.79
AMP 0.3+ERY 0.08+GEN 0.25							
Median (n=32)	86.85	75.48	85.43	51.70	86.68	46.29	-7.38
Max	152.63	81.90	104.78	89.47	95.54	72.96	4.88

Min	54.14	71.57	66.01	20.35	83.72	11.61	-13.30
AMP 0.3+ERY 0.08+TMP 5							
Median (n=32)	86.85	75.48	55.82	51.70	57.40	21.30	-16.06
Max	152.63	81.90	60.53	89.47	67.17	41.77	-14.33
Min	54.14	71.57	51.17	20.35	46.24	10.06	-20.91
AMP 0.3+ERY 0.08+TOB 0.2							
Median (n=32)	86.85	75.48	97.75	51.70	106.89	94.59	24.44
Max	152.63	81.90	136.91	89.47	140.00	111.44	70.85
Min	54.14	71.57	69.31	20.35	83.79	68.61	9.94
AMP 0.3+FOX 0.7+FUS 0.02							
Median (n=32)	86.85	75.22	95.69	69.31	86.57	86.05	63.34
Max	152.63	113.77	131.31	120.28	145.18	95.12	128.33
Min	54.14	69.35	68.66	61.17	79.14	75.21	50.97
AMP 0.3+FOX 0.7+TMP 5							
Median (n=32)	86.85	75.22	55.82	69.31	57.40	40.97	-9.77
Max	152.63	113.77	60.53	120.28	67.17	51.39	5.21
Min	54.14	69.35	51.17	61.17	46.24	33.23	-20.25
AMP 0.3+FOX 0.7+TOB 0.2							
Median (n=32)	86.85	75.22	97.75	69.31	106.89	103.02	59.28

Max	152.63	113.77	136.91	120.28	140.00	124.11	92.37
Min	54.14	69.35	69.31	61.17	83.79	78.38	54.92
DOX 1+FOX 0.7+GEN 0.25							
Median (n=16)	95.35	75.22	85.44	46.36	50.80	60.15	24.19
Max	109.79	113.77	104.78	52.62	73.19	66.12	42.70
Min	83.39	69.35	66.01	34.79	29.08	53.36	5.18
DOX 1+FOX 0.7+TMP 5							
Median (n=8)	95.35	75.22	55.82	46.36	50.31	40.97	26.20
Max	109.79	113.77	60.53	52.62	73.34	51.39	34.03
Min	83.39	69.35	51.17	34.79	40.37	33.23	23.24
DOX 1+FOX 0.7+TOB 0.2							
Median (n=32)	95.35	75.22	97.75	46.36	75.77	103.02	43.51
Max	109.79	113.77	136.91	52.62	86.50	124.11	49.94
Min	83.39	69.35	69.31	34.79	72.31	78.38	39.59
DOX 1+FOX 0.7+VAN 0.5							
Median (n=32)	95.35	75.22	94.89	46.36	62.73	80.06	-19.15
Max	109.79	113.77	132.58	52.62	86.12	86.92	-11.07
Min	83.39	69.35	62.31	34.79	50.67	-4.66	-24.54
AMP 0.3+FUS 0.02+GEN 0.25							

Median (n=32)	86.85	95.69	85.44	86.57	86.68	81.45	64.21
Max	152.63	131.31	104.78	145.18	95.54	91.84	90.34
Min	54.14	68.66	66.01	79.14	83.72	66.37	34.74
AMP 0.3+FUS 0.02+TMP 5							
Median (n=32)	86.85	95.69	55.82	86.57	57.40	69.21	48.62
Max	152.63	131.31	60.53	145.18	67.17	78.25	67.47
Min	54.14	68.66	51.17	79.14	46.24	67.05	26.43
AMP 0.3+FUS 0.02+TOB 0.2							
Median (n=32)	86.85	95.69	97.75	86.57	106.89	88.95	79.27
Max	152.63	131.31	136.91	145.18	140.00	103.73	87.81
Min	54.14	68.66	69.31	79.14	83.79	70.58	73.13
DOX 1+FOX 0.7+FUS 0.02							
Median (n=16)	95.35	75.22	95.69	46.36	47.93	86.05	29.36
Max	109.79	113.77	131.31	52.62	68.27	95.12	75.14
Min	83.39	69.35	68.66	34.79	38.52	75.21	22.54
DOX 1+FUS 0.02+TMP 5							
Median (n=16)	95.35	95.69	55.82	47.93	48.50	50.63	39.92
Max	109.79	131.31	60.53	68.27	57.58	68.78	42.80
Min	83.39	68.66	51.17	38.52	31.99	38.72	35.67

DOX 1+FUS 0.02+TOB 0.2							
Median (n=32)	95.35	95.69	97.75	47.93	70.76	88.95	19.29
Max	109.79	131.31	136.91	68.27	89.44	103.73	26.71
Min	83.39	68.66	69.31	38.52	22.61	70.58	7.78
DOX 1+FUS 0.02+VAN 0.5							
Median (n=32)	95.35	95.69	94.89	47.93	62.73	109.37	33.93
Max	109.79	131.31	132.58	68.27	86.12	124.39	41.00
Min	83.39	68.66	62.31	38.52	50.67	92.71	22.17
AMP 0.3+FOX 0.7+GEN 0.25							
Median (n=32)	86.85	75.22	85.44	69.31	86.68	60.15	56.20
Max	152.63	113.77	104.78	120.28	95.54	66.12	69.41
Min	54.14	69.35	66.01	61.17	83.72	53.36	43.26
AMP 0.3+GEN 0.25+TMP 5							
Median (n=32)	86.85	85.44	55.82	86.69	57.40	51.37	35.41
Max	152.63	104.78	60.53	95.54	67.17	61.62	42.27
Min	54.14	66.01	51.17	83.72	46.24	38.92	31.08
AMP 0.3+GEN 0.25+TOB 0.2							
Median (n=32)	86.85	85.44	97.75	86.69	106.89	57.79	32.31
Max	152.63	104.78	136.91	95.54	140.00	71.29	45.19

Min	54.14	66.01	69.31	83.72	83.79	-6.01	26.73
DOX 1+FUS 0.02+GEN 0.25							
Median (n=16)	95.35	95.69	85.44	47.93	50.80	81.45	-2.96
Max	109.79	131.31	104.78	68.27	73.19	91.84	2.83
Min	83.39	68.66	66.01	38.52	29.08	66.37	-14.39
DOX 1+GEN 0.25+TMP 5							
Median (n=16)	95.35	85.44	55.82	50.80	48.50	51.37	30.79
Max	109.79	104.78	60.53	73.19	57.58	61.62	35.55
Min	83.39	66.01	51.17	29.08	31.99	38.92	18.92
DOX 1+GEN 0.25+TOB 0.2							
Median (n=32)	95.35	85.44	97.75	50.80	75.77	57.79	4.02
Max	109.79	104.78	136.91	73.19	86.50	71.29	16.08
Min	83.39	66.01	69.31	29.08	72.31	-6.01	-4.18
DOX 1+GEN 0.25+VAN 0.5							
Median (n=32)	95.35	85.44	94.89	50.80	62.73	60.44	13.63
Max	109.79	104.78	132.58	73.19	86.12	80.56	16.95
Min	83.39	66.01	62.31	29.08	50.67	-3.66	7.14
CHL 120+CLI 0.04+STR 2.8							
Median (n=16)	84.63	99.43	71.19	63.16	64.53	81.21	29.29

Max	91.82	128.75	105.54	73.49	87.71	87.93	57.97
Min	79.26	85.73	18.56	49.41	46.40	8.12	0.78
CHL 120+CLI 0.04+TMP 5							
Median (n=8)	84.63	99.43	55.82	63.16	50.04	46.99	31.36
Max	91.82	128.75	60.53	73.49	69.42	58.92	40.63
Min	79.26	85.73	51.17	49.41	41.30	33.51	14.53
CHL 120+CLI 0.04+TOB 0.2							
Median (n=32)	84.63	99.43	97.75	63.16	68.21	90.92	53.67
Max	91.82	128.75	136.91	73.49	81.29	138.17	72.37
Min	79.26	85.73	69.31	49.41	48.32	64.61	26.52
CHL 120+CLI 0.04+VAN 0.5							
Median (n=32)	84.63	99.43	94.89	63.16	64.60	105.83	44.85
Max	91.82	128.75	132.58	73.49	68.18	146.82	61.51
Min	79.26	85.73	62.31	49.41	56.68	68.49	9.07
CPR 0.2+GEN 0.25+TMP 5							
Median (n=16)	78.04	85.44	55.82	45.17	37.24	51.37	7.56
Max	89.38	104.78	60.53	54.75	48.85	61.62	18.06
Min	61.17	66.01	51.17	32.60	22.81	38.92	1.01
CPR 0.2+GEN 0.25+TOB 0.2							

Median (n=32)	78.04	85.44	97.75	45.17	52.41	57.79	1.36
Max	89.38	104.78	136.91	54.75	59.02	71.29	13.59
Min	61.17	66.01	69.31	32.60	45.63	-6.01	-13.79
CPR 0.2+GEN 0.25+VAN 0.5							
Median (n=32)	78.04	85.44	94.89	45.17	58.63	60.44	17.04
Max	89.38	104.78	132.58	54.75	61.45	80.56	34.29
Min	61.17	66.01	62.31	32.60	51.51	-3.66	7.76
CHL 120+CLI 0.04+CPR 0.2							
Median (n=16)	84.63	99.43	78.04	63.16	46.87	49.17	26.28
Max	91.82	128.75	89.38	73.49	54.46	54.56	38.54
Min	79.26	85.73	61.17	49.41	40.09	36.92	17.48
CHL 120+CPR 0.2+ERY 0.08							
Median (n=16)	84.63	78.04	75.48	46.87	63.86	62.04	15.42
Max	91.82	89.38	81.90	54.46	86.13	66.43	22.79
Min	79.26	61.17	71.57	40.09	39.91	52.16	5.47
CHL 120+CPR 0.2+FOX 0.7							
Median (n=16)	84.63	78.04	75.22	46.87	55.83	53.73	18.70
Max	91.82	89.38	113.77	54.46	70.61	63.56	35.93
Min	79.26	61.17	69.35	40.09	42.49	46.84	11.74

CHL 120+CPR 0.2+FUS 0.02							
Median (n=16)	84.63	78.04	95.69	46.87	56.79	74.68	14.56
Max	91.82	89.38	131.31	54.46	64.81	87.86	22.56
Min	79.26	61.17	68.66	40.09	30.44	61.49	5.02
CHL 120+CPR 0.2+TMP 5							
Median (n=8)	84.63	78.04	55.82	46.87	50.04	39.13	12.64
Max	91.82	89.38	60.53	54.46	69.42	47.03	21.46
Min	79.26	61.17	51.17	40.09	41.30	31.59	6.03
CHL 120+CPR 0.2+TOB 0.2							
Median (n=32)	84.63	78.04	97.75	46.87	68.21	52.41	19.80
Max	91.82	89.38	136.91	54.46	81.29	59.02	43.46
Min	79.26	61.17	69.31	40.09	48.32	45.63	16.46
CHL 120+CPR 0.2+VAN 0.5							
Median (n=32)	84.63	78.04	94.89	46.87	64.60	58.63	5.87
Max	91.82	89.38	132.58	54.46	68.18	61.45	19.69
Min	79.26	61.17	62.31	40.09	56.68	51.51	-6.29
CHL 120+CLI 0.04+DOX 1							
Median (n=16)	84.63	99.43	95.36	63.16	14.23	18.98	-4.10
Max	91.82	128.75	109.79	73.49	22.72	22.69	4.31

Min	79.26	85.73	83.39	49.41	8.10	12.25	-16.24
CHL 120+CPR 0.2+DOX 1							
Median (n=16)	84.63	78.04	95.36	46.87	14.23	47.05	8.30
Max	91.82	89.38	109.79	54.46	22.72	54.20	22.85
Min	79.26	61.17	83.39	40.09	8.10	44.87	2.25
CHL 120+DOX 1+FUS 0.02							
Median (n=16)	84.63	95.36	95.69	14.23	56.79	47.93	-3.71
Max	91.82	109.79	131.31	22.72	64.81	68.27	14.40
Min	79.26	83.39	68.66	8.10	30.44	38.52	-6.87
CHL 120+DOX 1+TMP 5							
Median (n=8)	84.63	95.36	55.82	14.23	50.04	50.31	0.59
Max	91.82	109.79	60.53	22.72	69.42	73.34	6.05
Min	79.26	83.39	51.17	8.10	41.30	40.37	-3.86
CHL 120+DOX 1+TOB 0.2							
Median (n=32)	84.63	95.36	97.75	14.23	68.21	75.77	0.21
Max	91.82	109.79	136.91	22.72	81.29	86.50	3.19
Min	79.26	83.39	69.31	8.10	48.32	72.31	-9.64
CHL 120+DOX 1+VAN 0.5							
Median (n=32)	84.63	95.36	94.89	14.23	64.60	62.73	-7.80

Max	91.82	109.79	132.58	22.72	68.18	86.12	5.48
Min	79.26	83.39	62.31	8.10	56.68	50.67	-13.79
CHL 120+CLI 0.04+ERY 0.08							
Median (n=16)	84.63	99.43	75.48	63.86	63.16	100.16	50.01
Max	91.82	128.75	81.90	86.13	73.49	110.96	77.74
Min	79.26	85.73	71.57	39.91	49.41	68.66	6.16
CHL 120+ERY 0.08+FUS 0.02							
Median (n=16)	84.63	75.48	95.69	63.86	56.79	77.41	50.83
Max	91.82	81.90	131.31	86.13	64.81	96.39	68.78
Min	79.26	71.57	68.66	39.91	30.44	64.57	-3.98
CHL 120+ERY 0.08+STR 2.8							
Median (n=16)	84.63	75.48	71.19	63.86	64.53	79.14	47.61
Max	91.82	81.90	105.54	86.13	87.71	96.35	68.65
Min	79.26	71.57	18.56	39.91	46.40	48.60	16.24
CHL 120+ERY 0.08+TMP 5							
Median (n=8)	84.63	75.48	55.82	63.86	50.04	21.30	16.91
Max	91.82	81.90	60.53	86.13	69.42	41.77	22.34
Min	79.26	71.57	51.17	39.91	41.30	10.06	13.80
CHL 120+ERY 0.08+TOB 0.2							

Median (n=32)	84.63	75.48	97.75	63.86	68.21	94.59	55.33
Max	91.82	81.90	136.91	86.13	81.29	111.44	69.75
Min	79.26	71.57	69.31	39.91	48.32	68.61	13.26
CHL 120+ERY 0.08+VAN 0.5							
Median (n=32)	84.63	75.48	94.89	63.86	64.60	103.91	50.71
Max	91.82	81.90	132.58	86.13	68.18	124.03	63.58
Min	79.26	71.57	62.31	39.91	56.68	92.44	-3.64
CPR 0.2+TMP 5+TOB 0.2							
Median (n=32)	78.04	55.82	97.75	39.13	52.41	59.58	34.20
Max	89.38	60.53	136.91	47.03	59.02	68.60	47.52
Min	61.17	51.17	69.31	31.59	45.63	57.47	31.07
CHL 120+CLI 0.04+FOX 0.7							
Median (n=16)	84.63	99.43	75.22	63.16	55.83	67.51	48.84
Max	91.82	128.75	113.77	73.49	70.61	111.52	84.24
Min	79.26	85.73	69.35	49.41	42.49	60.84	19.62
CHL 120+DOX 1+FOX 0.7							
Median (n=16)	84.63	95.36	75.22	14.23	55.83	46.36	-0.51
Max	91.82	109.79	113.77	22.72	70.61	52.62	15.83
Min	79.26	83.39	69.35	8.10	42.49	34.79	-9.97

CHL 120+ERY 0.08+FOX 0.7							
Median (n=16)	84.63	75.48	75.22	63.86	55.83	82.63	41.07
Max	91.82	81.90	113.77	86.13	70.61	93.07	72.75
Min	79.26	71.57	69.35	39.91	42.49	53.44	3.99
CHL 120+FOX 0.7+GEN 0.25							
Median (n=16)	84.63	75.22	85.44	55.83	47.99	60.15	24.42
Max	91.82	113.77	104.78	70.61	58.56	66.12	50.72
Min	79.26	69.35	66.01	42.49	33.53	53.36	9.64
CHL 120+FOX 0.7+STR 2.8							
Median (n=16)	84.63	75.22	71.19	55.83	64.53	65.35	42.33
Max	91.82	113.77	105.54	70.61	87.71	88.02	53.16
Min	79.26	69.35	18.56	42.49	46.40	53.01	12.35
CHL 120+FOX 0.7+TMP 5							
Median (n=8)	84.63	75.22	55.82	55.83	50.04	40.97	28.32
Max	91.82	113.77	60.53	70.61	69.42	51.39	36.03
Min	79.26	69.35	51.17	42.49	41.30	33.23	24.82
CHL 120+FOX 0.7+TOB 0.2							
Median (n=32)	84.63	75.22	97.75	55.83	68.21	103.02	51.33
Max	91.82	113.77	136.91	70.61	81.29	124.11	63.07

Min	79.26	69.35	69.31	42.49	48.32	78.38	41.81
CHL 120+FOX 0.7+VAN 0.5							
Median (n=32)	84.63	75.22	94.89	55.83	64.60	80.06	34.93
Max	91.82	113.77	132.58	70.61	68.18	86.92	50.54
Min	79.26	69.35	62.31	42.49	56.68	-4.66	-10.63
ERY 0.08+FOX 0.7+FUS 0.02							
Median (n=16)	75.48	75.22	95.69	82.63	77.41	86.05	74.63
Max	81.90	113.77	131.31	93.07	96.39	95.12	79.89
Min	71.57	69.35	68.66	53.44	64.57	75.21	68.58
ERY 0.08+FOX 0.7+GEN 0.25							
Median (n=16)	75.48	75.22	85.44	82.63	46.29	60.15	38.58
Max	81.90	113.77	104.78	93.07	72.96	66.12	65.24
Min	71.57	69.35	66.01	53.44	11.61	53.36	23.67
ERY 0.08+FOX 0.7+STR 2.8							
Median (n=16)	75.48	75.22	71.19	82.63	79.14	65.35	63.74
Max	81.90	113.77	105.54	93.07	96.35	88.02	74.14
Min	71.57	69.35	18.56	53.44	48.60	53.01	9.23
ERY 0.08+FOX 0.7+TMP 5							
Median (n=8)	75.48	75.22	55.82	82.63	21.30	40.97	25.01

Max	81.90	113.77	60.53	93.07	41.77	51.39	38.95
Min	71.57	69.35	51.17	53.44	10.06	33.23	4.50
ERY 0.08+FOX 0.7+TOB 0.2							
Median (n=32)	75.48	75.22	97.75	82.63	94.59	103.02	83.63
Max	81.90	113.77	136.91	93.07	111.44	124.11	90.67
Min	71.57	69.35	69.31	53.44	68.61	78.38	71.52
ERY 0.08+FOX 0.7+VAN 0.5							
Median (n=32)	75.48	75.22	94.89	82.63	103.91	80.06	70.67
Max	81.90	113.77	132.58	93.07	124.03	86.92	76.84
Min	71.57	69.35	62.31	53.44	92.44	-4.66	58.84
CHL 120+CLI 0.04+FUS 0.02							
Median (n=16)	84.63	99.43	95.69	63.16	56.79	94.56	49.76
Max	91.82	128.75	131.31	73.49	64.81	112.05	76.03
Min	79.26	85.73	68.66	49.41	30.44	64.41	2.00
CHL 120+FOX 0.7+FUS 0.02							
Median (n=16)	84.63	75.22	95.69	55.83	56.79	86.05	39.77
Max	91.82	113.77	131.31	70.61	64.81	95.12	52.14
Min	79.26	69.35	68.66	42.49	30.44	75.21	0.62
CHL 120+FUS 0.02+STR 2.8							

Median (n=16)	84.63	95.69	71.19	56.79	64.53	20.03	30.11
Max	91.82	131.31	105.54	64.81	87.71	84.73	56.94
Min	79.26	68.66	18.56	30.44	46.40	3.26	2.71
CHL 120+FUS 0.02+TMP 5							
Median (n=16)	84.63	95.69	55.82	56.79	50.04	50.63	15.24
Max	91.82	131.31	60.53	64.81	69.42	68.78	36.87
Min	79.26	68.66	51.17	30.44	41.30	38.72	3.84
CHL 120+FUS 0.02+TOB 0.2							
Median (n=32)	84.63	95.69	97.75	56.79	68.21	88.95	44.74
Max	91.82	131.31	136.91	64.81	81.29	103.73	58.53
Min	79.26	68.66	69.31	30.44	48.32	70.58	35.96
CHL 120+FUS 0.02+VAN 0.5							
Median (n=32)	84.63	95.69	94.89	56.79	64.60	109.37	43.77
Max	91.82	131.31	132.58	64.81	68.18	124.39	56.32
Min	79.26	68.66	62.31	30.44	56.68	92.71	33.11
ERY 0.08+FUS 0.02+STR 2.8							
Median (n=16)	75.48	95.69	71.19	77.42	79.14	20.03	68.29
Max	81.90	131.31	105.54	96.39	96.35	84.73	87.76
Min	71.57	68.66	18.56	64.57	48.60	3.26	9.90

ERY 0.08+FUS 0.02+TMP 5							
Median (n=16)	75.48	95.69	55.82	77.42	21.30	50.63	9.85
Max	81.90	131.31	60.53	96.39	41.77	68.78	31.07
Min	71.57	68.66	51.17	64.57	10.06	38.72	5.72
ERY 0.08+FUS 0.02+TOB 0.2							
Median (n=32)	75.48	95.69	97.75	77.42	94.59	88.95	80.54
Max	81.90	131.31	136.91	96.39	111.44	103.73	118.03
Min	71.57	68.66	69.31	64.57	68.61	70.58	32.25
ERY 0.08+FUS 0.02+VAN 0.5							
Median (n=32)	75.48	95.69	94.89	77.42	103.91	109.37	79.73
Max	81.90	131.31	132.58	96.39	124.03	124.39	96.17
Min	71.57	68.66	62.31	64.57	92.44	92.71	31.72
CHL 120+CLI 0.04+GEN 0.25							
Median (n=16)	84.63	99.43	85.44	63.16	47.99	-1.32	15.84
Max	91.82	128.75	104.78	73.49	58.56	9.55	74.18
Min	79.26	85.73	66.01	49.41	33.53	-8.46	-1.91
CHL 120+CPR 0.2+GEN 0.25							
Median (n=16)	84.63	78.04	85.44	46.87	47.99	45.17	12.23
Max	91.82	89.38	104.78	54.46	58.56	54.75	25.53

Min	79.26	61.17	66.01	40.09	33.53	32.60	5.04
CHL 120+DOX 1+GEN 0.25							
Median (n=16)	84.63	95.36	85.44	14.23	47.99	50.80	-6.04
Max	91.82	109.79	104.78	22.72	58.56	73.19	-0.45
Min	79.26	83.39	66.01	8.10	33.53	29.08	-10.66
CHL 120+ERY 0.08+GEN 0.25							
Median (n=16)	84.63	75.48	85.44	63.86	47.99	46.29	8.30
Max	91.82	81.90	104.78	86.13	58.56	72.96	27.19
Min	79.26	71.57	66.01	39.91	33.53	11.61	-1.48
CHL 120+FUS 0.02+GEN 0.25							
Median (n=16)	84.63	95.69	85.44	56.79	47.99	81.45	1.95
Max	91.82	131.31	104.78	64.81	58.56	91.84	45.48
Min	79.26	68.66	66.01	30.44	33.53	66.37	-5.20
CHL 120+GEN 0.25+TMP 5							
Median (n=16)	84.63	85.44	55.82	47.99	50.04	51.37	26.54
Max	91.82	104.78	60.53	58.56	69.42	61.62	34.78
Min	79.26	66.01	51.17	33.53	41.30	38.92	12.30
CHL 120+GEN 0.25+TOB 0.2							
Median (n=32)	84.63	85.44	97.75	47.99	68.21	57.79	1.77

Max	91.82	104.78	136.91	58.56	81.29	71.29	23.66
Min	79.26	66.01	69.31	33.53	48.32	-6.01	-5.48
CHL 120+GEN 0.25+VAN 0.5							
Median (n=32)	84.63	85.44	94.89	47.99	64.60	60.44	4.11
Max	91.82	104.78	132.58	58.56	68.18	80.56	22.35
Min	79.26	66.01	62.31	33.53	56.68	-3.66	-5.18
ERY 0.08+FUS 0.02+GEN 0.25							
Median (n=16)	75.48	95.69	85.44	77.41	46.29	81.45	1.47
Max	81.90	131.31	104.78	96.39	72.96	91.84	14.47
Min	71.57	68.66	66.01	64.57	11.61	66.37	-2.58
ERY 0.08+GEN 0.25+TMP 5							
Median (n=16)	75.48	85.44	55.82	46.29	21.30	51.37	-0.99
Max	81.90	104.78	60.53	72.96	41.77	61.62	12.58
Min	71.57	66.01	51.17	11.61	10.06	38.92	-8.50
ERY 0.08+GEN 0.25+TOB 0.2							
Median (n=32)	75.48	85.44	97.75	46.29	94.59	57.79	2.83
Max	81.90	104.78	136.91	72.96	111.44	71.29	63.43
Min	71.57	66.01	69.31	11.61	68.61	-6.01	-3.47
ERY 0.08+GEN 0.25+VAN 0.5							

Median (n=33)	75.48	85.44	94.89	46.29	103.91	60.44	27.11
Max	81.90	104.78	132.58	72.96	124.03	80.56	37.76
Min	71.57	66.01	62.31	11.61	92.44	-3.66	19.74
CLI 0.04+CPR 0.2+DOX 1							
Median (n=8)	99.43	78.04	95.36	49.17	18.98	47.05	12.82
Max	128.75	89.38	109.79	54.56	22.69	54.20	17.46
Min	85.73	61.17	83.39	36.92	12.25	44.87	3.68
CLI 0.04+CPR 0.2+ERY 0.08							
Median (n=8)	99.43	78.04	75.48	49.17	100.16	62.04	44.89
Max	128.75	89.38	81.90	54.56	110.96	66.43	46.67
Min	85.73	61.17	71.57	36.92	68.66	52.16	36.13
CLI 0.04+CPR 0.2+FOX 0.7							
Median (n=8)	99.43	78.04	75.22	49.17	67.51	53.73	40.26
Max	128.75	89.38	113.77	54.56	111.52	63.56	44.92
Min	85.73	61.17	69.35	36.92	60.84	46.84	33.37
CLI 0.04+CPR 0.2+FUS 0.02							
Median (n=16)	99.43	78.04	95.69	49.17	94.56	74.68	46.13
Max	128.75	89.38	131.31	54.56	112.05	87.86	50.69
Min	85.73	61.17	68.66	36.92	64.41	61.49	39.43

CLI 0.04+CPR 0.2+GEN 0.25							
Median (n=16)	99.43	78.04	85.44	49.17	-1.32	45.17	-7.98
Max	128.75	89.38	104.78	54.56	9.55	54.75	0.96
Min	85.73	61.17	66.01	36.92	-8.46	32.60	-13.67
CLI 0.04+CPR 0.2+TMP 5							
Median (n=8)	99.43	78.04	55.82	49.17	46.99	39.13	29.67
Max	128.75	89.38	60.53	54.56	58.92	47.03	36.77
Min	85.73	61.17	51.17	36.92	33.51	31.59	19.97
CLI 0.04+CPR 0.2+TOB 0.2							
Median (n=32)	99.43	78.04	97.75	49.17	90.92	52.41	31.79
Max	128.75	89.38	136.91	54.56	138.17	59.02	46.05
Min	85.73	61.17	69.31	36.92	64.61	45.63	14.38
CLI 0.04+CPR 0.2+VAN 0.5							
Median (n=32)	99.43	78.04	94.89	49.17	105.83	58.63	41.49
Max	128.75	89.38	132.58	54.56	146.82	61.45	46.41
Min	85.73	61.17	62.31	36.92	68.49	51.51	34.74
CLI 0.04+FUS 0.02+GEN 0.25							
Median (n=16)	99.43	95.69	85.44	94.56	-1.32	81.45	-5.91
Max	128.75	131.31	104.78	112.05	9.55	91.84	2.98

Min	85.73	68.66	66.01	64.41	-8.46	66.37	-10.68
CLI 0.04+FUS 0.02+STR 2.8							
Median (n=16)	99.43	95.69	71.19	94.56	81.21	20.03	68.49
Max	128.75	131.31	105.54	112.05	87.93	84.73	77.61
Min	85.73	68.66	18.56	64.41	8.12	3.26	-6.06
CLI 0.04+FUS 0.02+TMP 5							
Median (n=16)	99.43	95.69	55.82	94.56	46.99	50.63	43.56
Max	128.75	131.31	60.53	112.05	58.92	68.78	49.11
Min	85.73	68.66	51.17	64.41	33.51	38.72	33.83
CLI 0.04+FUS 0.02+TOB 0.2							
Median (n=32)	99.43	95.69	97.75	94.56	90.92	88.95	71.94
Max	128.75	131.31	136.91	112.05	138.17	103.73	79.96
Min	85.73	68.66	69.31	64.41	64.61	70.58	31.92
CLI 0.04+FUS 0.02+VAN 0.5							
Median (n=32)	99.43	95.69	94.89	94.56	105.83	109.37	78.52
Max	128.75	131.31	132.58	112.05	146.82	124.39	93.60
Min	85.73	68.66	62.31	64.41	68.49	92.71	48.66
CLI 0.04+DOX 1+FOX 0.7							
Median (n=16)	99.43	95.36	75.22	18.98	67.51	46.36	13.41

Max	128.75	109.79	113.77	22.69	111.52	52.62	58.35
Min	85.73	83.39	69.35	12.25	60.84	34.79	-0.68
CLI 0.04+DOX 1+FUS 0.02							
Median (n=16)	99.43	95.36	95.69	18.98	94.56	47.93	2.19
Max	128.75	109.79	131.31	22.69	112.05	68.27	13.79
Min	85.73	83.39	68.66	12.25	64.41	38.52	-7.97
CLI 0.04+DOX 1+GEN 0.25							
Median (n=16)	99.43	95.36	85.44	18.98	-1.32	50.80	-10.00
Max	128.75	109.79	104.78	22.69	9.55	73.19	-4.79
Min	85.73	83.39	66.01	12.25	-8.46	29.08	-16.25
CLI 0.04+DOX 1+TMP 5							
Median (n=8)	99.43	95.36	55.82	18.98	46.99	50.31	-12.45
Max	128.75	109.79	60.53	22.69	58.92	73.34	-0.55
Min	85.73	83.39	51.17	12.25	33.51	40.37	-15.22
CLI 0.04+DOX 1+TOB 0.2							
Median (n=32)	99.43	95.36	97.75	18.98	90.92	75.77	-7.56
Max	128.75	109.79	136.91	22.69	138.17	86.50	2.09
Min	85.73	83.39	69.31	12.25	64.61	72.31	-15.62
CLI 0.04+DOX 1+VAN 0.5							

Median (n=32)	99.43	95.36	94.89	18.98	105.83	62.73	-7.98
Max	128.75	109.79	132.58	22.69	146.82	86.12	7.01
Min	85.73	83.39	62.31	12.25	68.49	50.67	-18.12
CLI 0.04+GEN 0.25+TMP 5							
Median (n=16)	99.43	85.44	55.82	-1.32	46.99	51.37	9.43
Max	128.75	104.78	60.53	9.55	58.92	61.62	14.35
Min	85.73	66.01	51.17	-8.46	33.51	38.92	2.19
CLI 0.04+GEN 0.25+TOB 0.2							
Median (n=32)	99.43	85.44	97.75	-1.32	90.92	57.79	-8.39
Max	128.75	104.78	136.91	9.55	138.17	71.29	-4.71
Min	85.73	66.01	69.31	-8.46	64.61	-6.01	-15.52
CLI 0.04+GEN 0.25+VAN 0.5							
Median (n=32)	99.43	85.44	94.89	-1.32	105.83	60.44	-11.00
Max	128.75	104.78	132.58	9.55	146.82	80.56	0.16
Min	85.73	66.01	62.31	-8.46	68.49	-3.66	-17.04
CLI 0.04+ERY 0.08+FUS 0.02							
Median (n=16)	99.43	75.48	95.69	100.16	94.56	77.41	86.79
Max	128.75	81.90	131.31	110.96	112.05	96.39	99.55
Min	85.73	71.57	68.66	68.66	64.41	64.57	2.30

CLI 0.04+ERY 0.08+GEN 0.25							
Median (n=16)	99.43	75.48	85.44	100.16	-1.32	46.29	23.40
Max	128.75	81.90	104.78	110.96	9.55	72.96	41.45
Min	85.73	71.57	66.01	68.66	-8.46	11.61	17.09
CLI 0.04+ERY 0.08+STR 2.8							
Median (n=16)	99.43	75.48	71.19	100.16	81.21	79.14	18.24
Max	128.75	81.90	105.54	110.96	87.93	96.35	77.65
Min	85.73	71.57	18.56	68.66	8.12	48.60	11.40
CLI 0.04+ERY 0.08+TMP 5							
Median (n=8)	99.43	75.48	55.82	100.16	46.99	21.30	19.55
Max	128.75	81.90	60.53	110.96	58.92	41.77	54.39
Min	85.73	71.57	51.17	68.66	33.51	10.06	7.52
CLI 0.04+ERY 0.08+TOB 0.2							
Median (n=32)	99.43	75.48	97.75	100.16	90.92	94.59	85.06
Max	128.75	81.90	136.91	110.96	138.17	111.44	98.37
Min	85.73	71.57	69.31	68.66	64.61	68.61	41.79
CLI 0.04+ERY 0.08+VAN 0.5							
Median (n=32)	99.43	75.48	94.89	100.16	105.83	103.91	80.26
Max	128.75	81.90	132.58	110.96	146.82	124.03	88.87

Min	85.73	71.57	62.31	68.66	68.49	92.44	77.41
CLI 0.04+ERY 0.08+FOX 0.7							
Median (n=16)	99.43	75.48	75.22	100.16	67.51	82.63	86.97
Max	128.75	81.90	113.77	110.96	111.52	93.07	124.67
Min	85.73	71.57	69.35	68.66	60.84	53.44	47.26
CLI 0.04+FOX 0.7+FUS 0.02							
Median (n=16)	99.43	75.22	95.69	67.51	94.56	86.05	76.94
Max	128.75	113.77	131.31	111.52	112.05	95.12	113.19
Min	85.73	69.35	68.66	60.84	64.41	75.21	54.48
CLI 0.04+FOX 0.7+GEN 0.25							
Median (n=16)	99.43	75.22	85.44	67.51	-1.32	60.15	23.89
Max	128.75	113.77	104.78	111.52	9.55	66.12	39.10
Min	85.73	69.35	66.01	60.84	-8.46	53.36	6.52
CLI 0.04+FOX 0.7+STR 2.8							
Median (n=16)	99.43	75.22	71.19	67.51	81.21	65.35	65.00
Max	128.75	113.77	105.54	111.52	87.93	88.02	91.56
Min	85.73	69.35	18.56	60.84	8.12	53.01	-8.06
CLI 0.04+FOX 0.7+TMP 5							
Median (n=8)	99.43	75.22	55.82	67.51	46.99	40.97	47.18

Max	128.75	113.77	60.53	111.52	58.92	51.39	56.27
Min	85.73	69.35	51.17	60.84	33.51	33.23	44.81
CLI 0.04+FOX 0.7+TOB 0.2							
Median (n=32)	99.43	75.22	97.75	67.51	90.92	103.02	79.95
Max	128.75	113.77	136.91	111.52	138.17	124.11	105.01
Min	85.73	69.35	69.31	60.84	64.61	78.38	39.03
CLI 0.04+FOX 0.7+VAN 0.5							
Median (n=32)	99.43	75.22	94.89	67.51	105.83	80.06	64.68
Max	128.75	113.77	132.58	111.52	146.82	86.92	103.70
Min	85.73	69.35	62.31	60.84	68.49	-4.66	-13.72
CLI 0.04+STR 2.8+TMP 5							
Median (n=16)	99.43	71.19	55.82	46.99	46.99	45.28	9.43
Max	128.75	105.54	60.53	58.92	58.92	55.20	30.99
Min	85.73	18.56	51.17	33.51	33.51	37.23	-3.92
CLI 0.04+STR 2.8+VAN 0.5							
Median (n=32)	99.43	71.19	94.89	81.21	105.83	62.44	70.67
Max	128.75	105.54	132.58	87.93	146.82	72.65	88.61
Min	85.73	18.56	62.31	8.12	68.49	-11.87	-7.73
CPR 0.2+DOX 1+FOX 0.7							

Median (n=8)	78.04	95.36	75.22	47.05	53.73	46.36	28.83
Max	89.38	109.79	113.77	54.20	63.56	52.62	35.71
Min	61.17	83.39	69.35	44.87	46.84	34.79	19.04
CPR 0.2+DOX 1+FUS 0.02							
Median (n=16)	78.04	95.36	95.69	47.05	74.68	47.93	34.77
Max	89.38	109.79	131.31	54.20	87.86	68.27	45.90
Min	61.17	83.39	68.66	44.87	61.49	38.52	29.02
CPR 0.2+DOX 1+GEN 0.25							
Median (n=16)	78.04	95.36	85.44	47.05	45.17	50.80	15.24
Max	89.38	109.79	104.78	54.20	54.75	73.19	18.60
Min	61.17	83.39	66.01	44.87	32.60	29.08	4.57
CPR 0.2+DOX 1+TMP 5							
Median (n=8)	78.04	95.36	55.82	47.05	39.13	50.31	-6.73
Max	89.38	109.79	60.53	54.20	47.03	73.34	-0.45
Min	61.17	83.39	51.17	44.87	31.59	40.37	-14.21
CPR 0.2+DOX 1+TOB 0.2							
Median (n=32)	78.04	95.36	97.75	47.05	52.41	75.77	29.99
Max	89.38	109.79	136.91	54.20	59.02	86.50	37.02
Min	61.17	83.39	69.31	44.87	45.63	72.31	27.29

CPR 0.2+DOX 1+VAN 0.5							
Median (n=32)	78.04	95.36	94.89	47.05	58.63	62.73	18.45
Max	89.38	109.79	132.58	54.20	61.45	86.12	25.62
Min	61.17	83.39	62.31	44.87	51.51	50.67	4.74
FUS 0.02+GEN 0.25+TMP 5							
Median (n=16)	95.69	85.44	55.82	81.45	50.63	51.37	59.36
Max	131.31	104.78	60.53	91.84	68.78	61.62	70.05
Min	68.66	66.01	51.17	66.37	38.72	38.92	55.31
FUS 0.02+GEN 0.25+TOB 0.2							
Median (n=32)	95.69	85.44	97.75	81.45	88.95	57.79	19.93
Max	131.31	104.78	136.91	91.84	103.73	71.29	50.36
Min	68.66	66.01	69.31	66.37	70.58	-6.01	4.50
FUS 0.02+GEN 0.25+VAN 0.5							
Median (n=32)	95.69	85.44	94.89	81.45	109.37	60.44	48.59
Max	131.31	104.78	132.58	91.84	124.39	80.56	76.87
Min	68.66	66.01	62.31	66.37	92.71	-3.66	40.36
CPR 0.2+ERY 0.08+FUS 0.02							
Median (n=16)	78.04	75.48	95.69	62.04	74.68	77.41	55.63
Max	89.38	81.90	131.31	66.43	87.86	96.39	69.49

Min	61.17	71.57	68.66	52.16	61.49	64.57	36.91
CPR 0.2+ERY 0.08+GEN 0.25							
Median (n=16)	78.04	75.48	85.44	62.04	45.17	46.29	38.55
Max	89.38	81.90	104.78	66.43	54.75	72.96	62.22
Min	61.17	71.57	66.01	52.16	32.60	11.61	15.64
CPR 0.2+ERY 0.08+TMP 5							
Median (n=8)	78.04	75.48	55.82	62.04	39.13	21.30	16.37
Max	89.38	81.90	60.53	66.43	47.03	41.77	44.11
Min	61.17	71.57	51.17	52.16	31.59	10.06	4.39
CPR 0.2+ERY 0.08+TOB 0.2							
Median (n=32)	78.04	75.48	97.75	62.04	52.41	94.59	58.68
Max	89.38	81.90	136.91	66.43	59.02	111.44	69.59
Min	61.17	71.57	69.31	52.16	45.63	68.61	49.54
CPR 0.2+ERY 0.08+VAN 0.5							
Median (n=32)	78.04	75.48	94.89	62.04	58.63	103.91	50.30
Max	89.38	81.90	132.58	66.43	61.45	124.03	61.05
Min	61.17	71.57	62.31	52.16	51.51	92.44	-5.29
CPR 0.2+ERY 0.08+FOX 0.7							
Median (n=16)	78.04	75.48	75.22	62.04	53.73	82.63	45.32

Max	89.38	81.90	113.77	66.43	63.56	93.07	51.20
Min	61.17	71.57	69.35	52.16	46.84	53.44	36.11
CPR 0.2+FOX 0.7+GEN 0.25							
Median (n=16)	78.04	75.22	85.44	53.73	45.17	60.15	49.78
Max	89.38	113.77	104.78	63.56	54.75	66.12	63.77
Min	61.17	69.35	66.01	46.84	32.60	53.36	40.26
CPR 0.2+FOX 0.7+TMP 5							
Median (n=8)	78.04	75.22	55.82	53.73	39.13	40.97	8.45
Max	89.38	113.77	60.53	63.56	47.03	51.39	17.04
Min	61.17	69.35	51.17	46.84	31.59	33.23	3.88
CPR 0.2+FOX 0.7+TOB 0.2							
Median (n=32)	78.04	75.22	97.75	53.73	52.41	103.02	51.88
Max	89.38	113.77	136.91	63.56	59.02	124.11	58.16
Min	61.17	69.35	69.31	46.84	45.63	78.38	46.85
CPR 0.2+FOX 0.7+VAN 0.5							
Median (n=32)	78.04	75.22	94.89	53.73	58.63	80.06	-14.52
Max	89.38	113.77	132.58	63.56	61.45	86.92	-5.04
Min	61.17	69.35	62.31	46.84	51.51	-4.66	-20.29
DOX 1+TMP 5+VAN 0.5							

Median (n=32)	95.36	55.82	94.89	50.31	62.73	53.53	42.09
Max	109.79	60.53	132.58	73.34	86.12	59.72	50.79
Min	83.39	51.17	62.31	40.37	50.67	42.30	29.39
FUS 0.02+STR 2.8+TOB 0.2							
Median (n=32)	95.69	71.19	97.75	20.03	88.95	70.06	-2.20
Max	131.31	105.54	136.91	84.73	103.73	80.47	87.00
Min	68.66	18.56	69.31	3.26	70.58	67.84	-10.72
FUS 0.02+STR 2.8+VAN 0.5							
Median (n=32)	95.69	71.19	94.89	20.03	109.37	62.44	63.93
Max	131.31	105.54	132.58	84.73	124.39	72.65	89.56
Min	68.66	18.56	62.31	3.26	92.71	-11.87	-10.28
CPR 0.2+FOX 0.7+FUS 0.02							
Median (n=16)	78.04	75.22	95.69	53.73	74.68	86.05	58.27
Max	89.38	113.77	131.31	63.56	87.86	95.12	62.06
Min	61.17	69.35	68.66	46.84	61.49	75.21	48.47
CPR 0.2+FUS 0.02+GEN 0.25							
Median (n=16)	78.04	95.69	85.44	74.68	45.17	81.45	61.09
Max	89.38	131.31	104.78	87.86	54.75	91.84	72.49
Min	61.17	68.66	66.01	61.49	32.60	66.37	57.79

CPR 0.2+FUS 0.02+TMP 5							
Median (n=16)	78.04	95.69	55.82	74.68	39.13	50.63	50.13
Max	89.38	131.31	60.53	87.86	47.03	68.78	54.14
Min	61.17	68.66	51.17	61.49	31.59	38.72	36.04
CPR 0.2+FUS 0.02+TOB 0.2							
Median (n=32)	78.04	95.69	97.75	74.68	52.41	88.95	73.39
Max	89.38	131.31	136.91	87.86	59.02	103.73	81.59
Min	61.17	68.66	69.31	61.49	45.63	70.58	67.48
CPR 0.2+FUS 0.02+VAN 0.5							
Median (n=32)	78.04	95.69	94.89	74.68	58.63	109.37	56.68
Max	89.38	131.31	132.58	87.86	61.45	124.39	67.15
Min	61.17	68.66	62.31	61.49	51.51	92.71	50.66
DOX 1+TMP 5+TOB 0.2							
Median (n=32)	95.36	55.82	97.75	48.50	75.77	59.58	44.91
Max	109.79	60.53	136.91	57.58	86.50	68.60	57.55
Min	83.39	51.17	69.31	31.99	72.31	57.47	26.87
DOX 1+TOB 0.2+VAN 0.5							
Median (n=32)	95.36	97.75	94.89	75.77	62.73	102.49	38.29
Max	109.79	136.91	132.58	86.50	86.12	113.36	50.53

Min	83.39	69.31	62.31	72.31	50.67	93.41	17.07
FUS 0.02+STR 2.8+TMP 5							
Median (n=16)	95.69	71.19	55.82	20.03	69.21	45.28	53.90
Max	131.31	105.54	60.53	84.73	78.25	55.20	78.83
Min	68.66	18.56	51.17	3.26	67.05	37.23	41.20
FUS 0.02+TMP 5+TOB 0.2							
Median (n=32)	95.69	55.82	97.75	69.21	88.95	59.58	67.50
Max	131.31	60.53	136.91	78.25	103.73	68.60	73.49
Min	68.66	51.17	69.31	67.05	70.58	57.47	61.33
FOX 0.7+FUS 0.02+GEN 0.25							
Median (n=16)	95.69	95.69	85.44	86.05	60.15	81.45	58.23
Max	131.31	131.31	104.78	95.12	66.12	91.84	71.19
Min	68.66	68.66	66.01	75.21	53.36	66.37	51.36
FOX 0.7+FUS 0.02+STR 2.8							
Median (n=16)	75.22	95.69	71.19	86.05	65.35	20.03	71.03
Max	113.77	131.31	105.54	95.12	88.02	84.73	95.74
Min	69.35	68.66	18.56	75.21	53.01	3.26	13.87
FOX 0.7+FUS 0.02+TMP 5							
Median (n=16)	75.22	95.69	55.82	86.05	40.97	50.63	40.92

Max	113.77	131.31	60.53	95.12	51.39	68.78	43.95
Min	69.35	68.66	51.17	75.21	33.23	38.72	31.03
FOX 0.7+FUS 0.02+TOB 0.2							
Median (n=32)	75.22	95.69	97.75	86.05	103.02	88.95	85.41
Max	113.77	131.31	136.91	95.12	124.11	103.73	97.35
Min	69.35	68.66	69.31	75.21	78.38	70.58	68.50
FOX 0.7+FUS 0.02+VAN 0.5							
Median (n=32)	75.22	95.69	94.89	86.05	80.06	109.37	72.46
Max	113.77	131.31	132.58	95.12	86.92	124.39	110.53
Min	69.35	68.66	62.31	75.21	-4.66	92.71	59.62
FOX 0.7+STR 2.8+TMP 5							
Median (n=16)	75.22	71.19	55.82	84.67	40.97	45.28	39.82
Max	113.77	105.54	60.53	95.24	51.39	55.20	51.85
Min	69.35	18.56	51.17	72.00	33.23	37.23	25.93
FOX 0.7+STR 2.8+TOB 0.2							
Median (n=32)	75.22	71.19	97.75	84.67	103.02	60.88	70.37
Max	113.77	105.54	136.91	95.24	124.11	84.18	91.03
Min	69.35	18.56	69.31	72.00	78.38	3.91	-11.14
GEN 0.25+TOB 0.2+VAN 0.5							

Median (n=32)	85.44	97.75	94.89	60.44	68.35	102.49	8.94
Max	104.78	136.91	132.58	80.56	79.66	113.36	70.58
Min	66.01	69.31	62.31	-3.66	51.67	93.41	-1.99
STR 2.8+TOB 0.2+VAN 0.5							
Median (n=32)	71.19	97.75	94.89	67.94	62.44	102.49	69.68
Max	105.54	136.91	132.58	84.18	72.65	113.36	92.32
Min	18.56	69.31	62.31	3.91	-11.87	93.41	-2.00
TMP 5+TOB 0.2+VAN 0.5							
Median (n=32)	55.82	97.75	94.89	59.58	53.53	102.49	54.08
Max	60.53	136.91	132.58	68.60	59.72	113.36	68.84
Min	51.17	69.31	62.31	57.47	42.30	93.41	49.89
GEN 0.25+TMP 5+TOB 0.2							
Median (n=32)	85.44	55.82	97.75	54.24	57.79	59.59	54.36
Max	104.78	60.53	136.91	60.21	71.29	68.60	62.37
Min	66.01	51.17	69.31	50.80	-6.01	57.47	44.99
GEN 0.25+TMP 5+VAN 0.5							
Median (n=32)	85.44	55.82	94.89	54.24	60.44	53.53	53.10
Max	104.78	60.53	132.58	60.21	80.56	59.72	57.17
Min	66.01	51.17	62.31	50.80	-3.66	42.30	47.74

STR 2.8+TMP 5+TOB 0.2							
Median (n=32)	71.19	55.82	97.75	45.28	70.06	59.59	15.69
Max	105.54	60.53	136.91	55.20	80.47	68.60	31.11
Min	18.56	51.17	69.31	37.23	67.84	57.47	3.91
STR 2.8+TMP 5+VAN 0.5							
Median (n=32)	71.19	55.82	94.89	45.29	53.53	53.53	22.75
Max	105.54	60.53	132.58	55.20	59.72	59.72	47.81
Min	18.56	51.17	62.31	37.23	42.30	42.30	6.56
FOX 0.7+GEN 0.25+TMP 5							
Median (n=16)	75.22	85.44	55.82	60.15	40.97	51.37	42.27
Max	113.77	104.78	60.53	66.12	51.39	61.62	48.10
Min	69.35	66.01	51.17	53.36	33.23	38.92	20.37
FOX 0.7+GEN 0.25+TOB 0.2							
Median (n=32)	75.22	85.44	97.75	60.15	103.02	57.79	52.79
Max	113.77	104.78	136.91	66.12	124.11	71.29	56.34
Min	69.35	66.01	69.31	53.36	78.38	-6.01	43.20
FOX 0.7+GEN 0.25+VAN 0.5							
Median (n=32)	75.22	85.44	94.89	60.15	80.06	60.44	-9.90
Max	113.77	104.78	132.58	66.12	86.92	80.56	-6.16

Min	69.35	66.01	62.31	53.36	-4.66	-3.66	-20.18
CHL 120+TMP 5+VAN 0.5							
Median (n=32)	84.63	55.82	94.89	50.04	64.60	53.53	31.00
Max	91.82	60.53	132.58	69.42	68.18	59.72	44.39
Min	79.26	51.17	62.31	41.30	56.68	42.30	13.09
CLI 0.04+TMP 5+TOB 0.2							
Median (n=32)	99.43	55.82	97.75	46.99	90.92	59.59	34.74
Max	128.75	60.53	136.91	58.92	138.17	68.60	52.57
Min	85.73	51.17	69.31	33.51	64.61	57.47	25.95
CLI 0.04+TMP 5+VAN 0.5							
Median (n=32)	99.43	55.82	94.89	46.99	105.83	53.53	40.23
Max	128.75	60.53	132.58	58.92	146.82	59.72	45.34
Min	85.73	51.17	62.31	33.51	68.49	42.30	29.37
CPR 0.2+TMP 5+VAN 0.5							
Median (n=32)	78.04	55.82	94.89	37.24	58.63	53.53	19.04
Max	89.38	60.53	132.58	48.85	61.45	59.72	39.42
Min	61.17	51.17	62.31	22.81	51.51	42.30	-5.58
ERY 0.08+TMP 5+VAN 0.5							
Median (n=32)	75.48	55.82	94.89	21.30	103.91	53.53	12.22

Max	81.90	60.53	132.58	41.77	124.03	59.72	47.99
Min	71.57	51.17	62.31	10.06	92.44	42.30	-0.52
FOX 0.7+TMP 5+TOB 0.2							
Median (n=32)	75.22	55.82	97.75	40.97	103.02	59.59	43.70
Max	113.77	60.53	136.91	51.39	124.11	68.60	51.55
Min	69.35	51.17	69.31	33.23	78.38	57.47	19.46
FOX 0.7+TMP 5+VAN 0.5							
Median (n=32)	75.22	55.82	94.89	40.97	80.06	53.53	3.85
Max	113.77	60.53	132.58	51.39	86.92	59.72	33.43
Min	69.35	51.17	62.31	33.23	-4.66	42.30	-1.61
FUS 0.02+TMP 5+VAN 0.5							
Median (n=32)	95.69	55.82	94.89	50.63	109.37	53.53	61.54
Max	131.31	60.53	132.58	68.78	124.39	59.72	73.93
Min	68.66	51.17	62.31	38.72	92.71	42.30	29.40
CHL 120+STR 2.8+TMP 5							
Median (n=16)	84.63	71.19	55.82	64.53	50.04	45.28	20.29
Max	91.82	105.54	60.53	87.71	69.42	55.20	30.54
Min	79.26	18.56	51.17	46.40	41.30	37.23	13.43
CHL 120+STR 2.8+TOB 0.2							

Median (n=32)	84.63	71.19	97.75	64.53	68.21	67.94	39.46
Max	91.82	105.54	136.91	87.71	81.29	84.18	45.99
Min	79.26	18.56	69.31	46.40	48.32	3.91	7.21
CHL 120+STR 2.8+VAN 0.5							
Median (n=32)	84.63	71.19	94.89	64.53	64.60	62.44	37.08
Max	91.82	105.54	132.58	87.71	68.18	72.65	46.78
Min	79.26	18.56	62.31	46.40	56.68	-11.87	29.16
CLI 0.04+STR 2.8+TOB 0.2							
Median (n=32)	99.43	71.19	97.75	81.21	90.92	67.94	66.11
Max	128.75	105.54	136.91	87.93	138.17	84.18	77.57
Min	85.73	18.56	69.31	8.12	64.61	3.91	-3.23
ERY 0.08+STR 2.8+TMP 5							
Median (n=16)	75.48	71.19	55.82	79.14	21.30	45.28	7.04
Max	81.90	105.54	60.53	96.35	41.77	55.20	23.48
Min	71.57	18.56	51.17	48.60	10.06	37.23	-2.55
ERY 0.08+STR 2.8+TOB 0.2							
Median (n=32)	75.48	71.19	97.75	79.14	94.59	67.94	65.37
Max	81.90	105.54	136.91	96.35	111.44	84.18	78.17
Min	71.57	18.56	69.31	48.60	68.61	3.91	59.11

ERY 0.08+STR 2.8+VAN 0.5							
Median (n=32)	75.48	71.19	94.89	79.14	103.91	62.44	63.38
Max	81.90	105.54	132.58	96.35	124.03	72.65	78.04
Min	71.57	18.56	62.31	48.60	92.44	-11.87	-1.61
CHL 120+TMP 5+TOB 0.2							
Median (n=32)	84.63	55.82	97.75	50.04	68.21	59.59	41.72
Max	91.82	60.53	136.91	69.42	81.29	68.60	56.95
Min	79.26	51.17	69.31	41.30	48.32	57.47	29.70
CPR 0.2+TOB 0.2+VAN 0.5							
Median (n=32)	78.04	97.75	94.89	52.41	58.63	102.49	49.00
Max	89.38	136.91	132.58	59.02	61.45	113.36	56.11
Min	61.17	69.31	62.31	45.63	51.51	93.41	22.84
ERY 0.08+TMP 5+TOB 0.2							
Median (n=32)	75.48	55.82	97.75	21.30	94.59	59.59	15.56
Max	81.90	60.53	136.91	41.77	111.44	68.60	36.01
Min	71.57	51.17	69.31	10.06	68.61	57.47	-2.58
CHL 120+TOB 0.2+VAN 0.5							
Median (n=32)	84.63	97.75	94.89	68.21	64.60	102.49	57.75
Max	91.82	136.91	132.58	81.29	68.18	113.36	83.21

Min	79.26	69.31	62.31	48.32	56.68	93.41	37.43
CLI 0.04+TOB 0.2+VAN 0.5							
Median (n=32)	84.63	97.75	94.89	90.92	64.60	102.49	91.88
Max	91.82	136.91	132.58	138.17	68.18	113.36	121.65
Min	79.26	69.31	62.31	64.61	56.68	93.41	27.77
ERY 0.08+TOB 0.2+VAN 0.5							
Median (n=32)	75.48	97.75	94.89	94.59	103.91	102.49	90.03
Max	81.90	136.91	132.58	111.44	124.03	113.36	129.25
Min	71.57	69.31	62.31	68.61	92.44	93.41	66.60
FOX 0.7+STR 2.8+VAN 0.5							
Median (n=32)	75.22	71.19	94.89	65.35	80.06	62.44	66.81
Max	113.77	105.54	132.58	88.02	86.92	72.65	75.66
Min	69.35	18.56	62.31	53.01	-4.66	-11.87	-3.18
FOX 0.7+TOB 0.2+VAN 0.5							
Median (n=32)	75.22	97.75	94.89	103.02	80.06	102.49	85.31
Max	113.77	136.91	132.58	124.11	86.92	113.36	116.81
Min	69.35	69.31	62.31	78.38	-4.66	93.41	70.53
FUS 0.02+TOB 0.2+VAN 0.5							
Median (n=32)	95.69	97.75	94.89	88.95	109.37	102.49	86.61

Max	131.31	136.91	132.58	103.73	124.39	113.36	115.05
Min	68.66	69.31	62.31	70.58	92.71	93.41	52.76
AMP 0.25+CHL 120+NTR 20							
Median (n=32)	96.19	99.22	83.19	53.91	42.49	-2.18	-6.46
Max	103.71	102.38	113.27	106.21	126.61	8.13	1.21
Min	91.41	92.59	65.04	-8.84	16.61	-8.65	-16.44
AMP 0.25+CLI 0.04+NTR 20							
Median (n=32)	96.19	106.46	83.19	74.96	42.49	52.85	-7.05
Max	103.71	111.15	113.27	86.29	126.61	84.83	10.39
Min	91.41	96.85	65.04	62.59	16.61	17.86	-15.74
AMP 0.25+CPR 0.15+NTR 20							
Median (n=32)	96.19	91.80	83.19	70.27	42.49	91.17	33.06
Max	103.71	126.70	113.27	108.11	126.61	109.30	100.43
Min	91.41	80.38	65.04	59.24	16.61	70.09	-0.34
AMP 0.25+DOX 0.9+NTR 20							
Median (n=32)	96.19	84.25	83.19	52.95	42.49	3.41	-10.71
Max	103.71	94.76	113.27	109.43	126.61	20.42	24.91
Min	91.41	72.51	65.04	40.26	16.61	-17.15	-18.42
AMP 0.25+ERY 0.07+NTR 20							

Median (n=32)	96.19	94.33	83.19	87.85	42.49	-4.67	-10.91
Max	103.71	117.27	113.27	159.19	126.61	7.43	25.97
Min	91.41	79.10	65.04	82.92	16.61	-13.46	-14.37
AMP 0.25+FOX 0.7+NTR 20							
Median (n=32)	96.19	78.10	83.19	70.30	42.49	56.19	10.68
Max	103.71	125.01	113.27	77.13	126.61	81.60	88.88
Min	91.41	68.65	65.04	64.36	16.61	28.08	-15.99
AMP 0.25+FUS 0.02+NTR 20							
Median (n=32)	96.19	94.12	83.19	83.58	42.49	58.25	6.67
Max	103.71	107.80	113.27	101.66	126.61	89.03	98.37
Min	91.41	82.11	65.04	74.06	16.61	37.74	-22.38
AMP 0.25+GEN 0.02+NTR 20							
Median (n=32)	96.19	102.73	83.19	100.76	42.49	0.70	-5.78
Max	103.71	128.07	113.27	110.97	126.61	71.31	25.45
Min	91.41	95.16	65.04	95.72	16.61	-18.54	-19.88
AMP 0.25+NTR 20+STR 2.5							
Median (n=32)	96.19	83.19	85.53	42.49	62.55	-9.26	-5.55
Max	103.71	113.27	88.88	126.61	94.99	-6.05	118.32
Min	91.41	65.04	75.78	16.61	47.67	-15.84	-19.25

AMP 0.25+NTR 20+TMP 5							
Median (n=32)	96.19	83.19	86.60	42.49	83.21	1.96	1.21
Max	103.71	113.27	99.83	126.61	88.36	17.63	112.64
Min	91.41	65.04	73.41	16.61	78.31	-10.20	-17.42
AMP 0.3+NTR 20+TOB 0.2							
Median (n=32)	96.19	83.19	105.58	42.49	100.04	6.01	-4.27
Max	103.71	113.27	120.42	126.61	107.78	42.16	37.38
Min	91.41	65.04	100.20	16.61	98.39	-1.93	-20.85
AMP 0.25+NTR 20+VAN 0.4							
Median (n=32)	96.19	83.19	99.60	42.49	44.95	1.58	-8.98
Max	103.71	113.27	123.10	126.61	124.37	59.41	27.93
Min	91.41	65.04	94.45	16.61	2.86	-17.36	-16.23
GEN 0.2+NTR 20+STR 2.5							
Median (n=32)	102.73	83.19	85.53	0.70	59.83	-9.26	-15.77
Max	128.07	113.27	88.88	71.31	105.36	-6.05	5.76
Min	95.16	65.04	75.78	-18.54	47.68	-15.84	-27.92
GEN 0.25+NTR 20+TMP 5							
Median (n=32)	102.73	83.19	86.60	0.70	75.09	1.96	-12.99
Max	128.07	113.27	99.83	71.31	86.05	17.63	101.58

Min	95.16	65.04	73.41	-18.54	67.25	-10.20	-23.67
GEN 0.25+NTR 20+TOB 0.2							
Median (n=32)	102.73	83.19	105.58	0.70	90.97	6.01	-11.34
Max	128.07	113.27	120.42	71.31	100.32	42.16	104.58
Min	95.16	65.04	100.20	-18.54	79.00	-1.93	-22.71
GEN 0.2+NTR 20+VAN 0.4							
Median (n=32)	102.73	83.19	99.60	0.70	92.85	1.58	-6.05
Max	128.07	113.27	123.10	71.31	114.28	59.41	33.22
Min	95.16	65.04	94.45	-18.54	81.87	-17.36	-12.68
CHL 120+CLI 0.04+NTR 20							
Median (n=32)	99.22	106.46	83.19	72.96	-2.18	52.85	-3.39
Max	102.38	111.15	113.27	91.20	8.13	84.83	13.42
Min	92.59	96.85	65.04	51.62	-8.65	17.86	-6.93
CHL 120+DOX 0.9+NTR 20							
Median (n=32)	99.22	84.25	83.19	34.24	-2.18	3.41	-8.75
Max	102.38	94.76	113.27	58.47	8.13	20.42	0.79
Min	92.59	72.51	65.04	17.25	-8.65	-17.15	-12.94
CHL 120+ERY 0.07+NTR 20							
Median (n=32)	99.22	94.33	83.19	32.15	-2.18	-4.67	-10.37

Max	102.38	117.27	113.27	47.70	8.13	7.43	-1.73
Min	92.59	79.10	65.04	5.22	-8.65	-13.46	-15.05
CHL 120+FOX 0.7+NTR 20							
Median (n=32)	99.22	78.10	83.19	48.53	-2.18	56.19	-7.53
Max	102.38	125.01	113.27	61.71	8.13	81.60	10.10
Min	92.59	68.65	65.04	29.24	-8.65	28.08	-15.04
CHL 120+FUS 0.02+NTR 20							
Median (n=32)	99.22	94.12	83.19	13.68	-2.18	58.25	-8.46
Max	102.38	107.80	113.27	22.30	8.13	89.03	2.03
Min	92.59	82.11	65.04	10.34	-8.65	37.74	-20.13
CHL 120+GEN 0.2+NTR 20							
Median (n=32)	99.22	102.73	83.19	57.56	-2.18	0.70	-10.49
Max	102.38	128.07	113.27	89.63	8.13	71.31	-0.57
Min	92.59	95.16	65.04	50.61	-8.65	-18.54	-18.38
CHL 120+NTR 20+STR 2.5							
Median (n=32)	99.22	83.19	85.53	-2.18	56.26	-9.26	-11.73
Max	102.38	113.27	88.88	8.13	70.69	-6.05	-8.48
Min	92.59	65.04	75.78	-8.65	42.03	-15.84	-19.95
CHL 120+NTR 20+TMP 5							

Median (n=32)	99.22	83.19	86.60	-2.18	68.27	1.96	-13.90
Max	102.38	113.27	99.83	8.13	86.92	17.63	-4.43
Min	92.59	65.04	73.41	-8.65	33.36	-10.20	-16.87
CHL 120+NTR 20+TOB 0.2							
Median (n=32)	99.22	83.19	105.58	-2.18	89.44	6.01	-11.26
Max	102.38	113.27	120.42	8.13	102.56	42.16	5.76
Min	92.59	65.04	100.20	-8.65	68.11	-1.93	-19.85
CHL 120+NTR 20+VAN 0.4							
Median (n=32)	99.22	83.19	99.60	-2.18	68.45	1.58	-12.88
Max	102.38	113.27	123.10	8.13	103.13	59.41	-8.48
Min	92.59	65.04	94.45	-8.65	39.77	-17.36	-14.74
NTR 20+STR 2.5+TMP 5							
Median (n=32)	83.19	85.53	86.60	-9.26	1.96	72.49	-4.13
Max	113.27	88.88	99.83	-6.05	17.63	134.21	13.35
Min	65.04	75.78	73.41	-15.84	-10.20	65.03	-13.66
NTR 20+STR 2.5+TOB 0.2							
Median (n=32)	83.19	85.53	105.58	-9.26	6.01	47.05	0.58
Max	113.27	88.88	120.42	-6.05	42.16	71.87	25.32
Min	65.04	75.78	100.20	-15.84	-1.93	39.96	-11.60

NTR 20+STR 2.5+VAN 0.4							
Median (n=32)	83.19	85.53	99.60	-9.26	1.58	63.50	-3.26
Max	113.27	88.88	123.10	-6.05	59.41	100.61	0.81
Min	65.04	75.78	94.45	-15.84	-17.36	21.07	-8.30
CLI 0.04+DOX 0.9+NTR 20							
Median (n=32)	106.46	84.25	83.19	34.12	52.85	3.41	8.99
Max	111.15	94.76	113.27	78.06	84.83	20.42	29.45
Min	96.85	72.51	65.04	21.22	17.86	-17.15	-5.00
CLI 0.04+ERY 0.07+NTR 20							
Median (n=32)	106.46	94.33	83.19	81.95	52.85	-4.67	13.11
Max	111.15	117.27	113.27	113.78	84.83	7.43	34.87
Min	96.85	79.10	65.04	66.47	17.86	-13.46	-0.68
CLI 0.04+FOX 0.7+NTR 20							
Median (n=32)	106.46	78.10	83.19	64.31	52.85	56.19	6.09
Max	111.15	125.01	113.27	71.88	84.83	81.60	57.11
Min	96.85	68.65	65.04	58.55	17.86	28.08	1.66
CLI 0.04+FUS 0.02+NTR 20							
Median (n=32)	106.46	94.12	83.19	42.03	52.85	58.25	-4.48
Max	111.15	107.80	113.27	49.76	84.83	89.03	24.18

Min	96.85	82.11	65.04	34.07	17.86	37.74	-55.30
CLI 0.04+GEN 0.2+NTR 20							
Median (n=32)	106.46	102.73	83.19	73.76	52.85	0.70	-4.71
Max	111.15	128.07	113.27	101.00	84.83	71.31	8.61
Min	96.85	95.16	65.04	-0.16	17.86	-18.54	-13.61
CLI 0.04+NTR 20+STR 2.5							
Median (n=32)	106.46	83.19	85.53	52.85	8.36	-9.26	2.53
Max	111.15	113.27	88.88	84.83	94.49	-6.05	12.79
Min	96.85	65.04	75.78	17.86	-6.91	-15.84	-8.67
CLI 0.04+NTR 20+TMP 5							
Median (n=32)	106.46	83.19	86.60	52.85	71.56	1.96	0.47
Max	111.15	113.27	99.83	84.83	79.24	17.63	14.33
Min	96.85	65.04	73.41	17.86	65.01	-10.20	-9.18
CLI 0.04+NTR 20+TOB 0.2							
Median (n=32)	106.46	83.19	105.58	52.85	85.76	6.01	2.13
Max	111.15	113.27	120.42	84.83	94.13	42.16	19.78
Min	96.85	65.04	100.20	17.86	72.29	-1.93	-9.18
CLI 0.04+NTR 20+VAN 0.4							
Median (n=32)	106.46	83.19	99.60	52.85	87.67	1.58	10.99

Max	111.15	113.27	123.10	84.83	98.28	59.41	32.03
Min	96.85	65.04	94.45	17.86	77.72	-17.36	-13.64
NTR 20+TMP 5+TOB 0.2							
Median (n=32)	83.19	86.60	105.58	1.96	6.01	76.78	4.38
Max	113.27	99.83	120.42	17.63	42.16	86.75	18.65
Min	65.04	73.41	100.20	-10.20	-1.93	72.19	-7.48
NTR 20+TMP 5+VAN 0.4							
Median (n=32)	83.19	86.60	99.60	1.96	1.58	84.53	1.75
Max	113.27	99.83	123.10	17.63	59.41	97.10	20.32
Min	65.04	73.41	94.45	-10.20	-17.36	79.82	-4.32
CHL 120+CPR 0.15+NTR 20							
Median (n=32)	99.22	91.80	83.19	56.18	-2.18	91.17	3.53
Max	102.38	126.70	113.27	78.19	8.13	109.30	50.20
Min	92.59	80.38	65.04	43.89	-8.65	70.09	-13.39
CLI 0.04+CPR 0.2+NTR 20							
Median (n=32)	106.46	91.80	83.19	78.20	52.85	91.17	52.43
Max	111.15	126.70	113.27	85.92	84.83	109.30	71.58
Min	96.85	80.38	65.04	70.44	17.86	70.09	14.13
CPR 0.15+DOX 0.9+NTR 20							

Median (n=32)	91.80	84.25	83.19	76.87	91.17	3.41	23.78
Max	126.70	94.76	113.27	91.17	109.30	20.42	38.51
Min	80.38	72.51	65.04	67.23	70.09	-17.15	-2.77
CPR 0.15+ERY 0.07+NTR 20							
Median (n=32)	91.80	94.33	83.19	73.29	91.17	-4.67	20.89
Max	126.70	117.27	113.27	120.70	109.30	7.43	43.04
Min	80.38	79.10	65.04	63.13	70.09	-13.46	-7.13
CPR 0.15+FOX 0.7+NTR 20							
Median (n=32)	91.80	78.10	83.19	56.15	91.17	56.19	46.30
Max	126.70	125.01	113.27	61.36	109.30	81.60	81.17
Min	80.38	68.65	65.04	52.37	70.09	28.08	26.01
CPR 0.15+FUS 0.02+NTR 20							
Median (n=32)	91.80	94.12	83.19	78.98	91.17	58.25	40.94
Max	126.70	107.80	113.27	86.48	109.30	89.03	118.83
Min	80.38	82.11	65.04	74.43	70.09	37.74	-0.66
CPR 0.15+GEN 0.2+NTR 20							
Median (n=32)	91.80	102.73	83.19	88.22	91.17	0.70	14.26
Max	126.70	128.07	113.27	127.26	109.30	71.31	34.35
Min	80.38	95.16	65.04	66.70	70.09	-18.54	-2.26

CPR 0.15+NTR 20+STR 2.5							
Median (n=32)	91.80	83.19	85.53	91.17	74.67	-9.26	10.89
Max	126.70	113.27	88.88	109.30	80.99	-6.05	19.08
Min	80.38	65.04	75.78	70.09	57.38	-15.84	-0.95
CPR 0.15+NTR 20+TMP 5							
Median (n=32)	91.80	83.19	86.60	91.17	59.55	1.96	3.26
Max	126.70	113.27	99.83	109.30	61.72	17.63	10.66
Min	80.38	65.04	73.41	70.09	57.91	-10.20	0.91
CPR 0.15+NTR 20+TOB 0.2							
Median (n=32)	91.80	83.19	105.58	91.17	79.67	6.01	10.04
Max	126.70	113.27	120.42	109.30	83.27	42.16	98.74
Min	80.38	65.04	100.20	70.09	74.63	-1.93	0.43
CPR 0.15+NTR 20+VAN 0.4							
Median (n=32)	91.80	83.19	99.60	91.17	72.41	1.58	72.62
Max	126.70	113.27	123.10	109.30	121.13	59.41	92.73
Min	80.38	65.04	94.45	70.09	67.69	-17.36	9.43
NTR 20+TOB 0.2+VAN 0.4							
Median (n=32)	83.19	105.58	99.60	6.01	1.58	99.17	11.93
Max	113.27	120.42	123.10	42.16	59.41	125.65	93.95

Min	65.04	100.20	94.45	-1.93	-17.36	92.28	0.09
DOX 0.9+ERY 0.07+NTR 20							
Median (n=32)	84.25	94.33	83.19	7.67	3.41	-4.67	-7.56
Max	94.76	117.27	113.27	87.95	20.42	7.43	-2.51
Min	72.51	79.10	65.04	-7.69	-17.15	-13.46	-14.74
DOX 0.9+FOX 0.7+NTR 20							
Median (n=32)	84.25	78.10	83.19	60.25	3.41	56.19	-6.97
Max	94.76	125.01	113.27	67.10	20.42	81.60	20.74
Min	72.51	68.65	65.04	55.61	-17.15	28.08	-14.47
DOX 0.9+FUS 0.02+NTR 20							
Median (n=32)	84.25	94.12	83.19	34.19	3.41	58.25	-2.19
Max	94.76	107.80	113.27	49.15	20.42	89.03	48.26
Min	72.51	82.11	65.04	25.03	-17.15	37.74	-13.09
DOX 0.9+GEN 0.2+NTR 20							
Median (n=32)	84.25	102.73	83.19	86.06	3.41	0.70	-4.48
Max	94.76	128.07	113.27	113.82	20.42	71.31	102.44
Min	72.51	95.16	65.04	52.18	-17.15	-18.54	-14.65
DOX 0.9+NTR 20+STR 2.5							
Median (n=32)	84.25	83.19	85.53	3.41	7.36	-9.26	-8.71

Max	94.76	113.27	88.88	20.42	78.81	-6.05	13.72
Min	72.51	65.04	75.78	-17.15	-11.02	-15.84	-19.67
DOX 0.9+NTR 20+TMP 5							
Median (n=32)	84.25	83.19	86.60	3.41	61.33	1.96	-13.35
Max	94.76	113.27	99.83	20.42	72.45	17.63	-5.89
Min	72.51	65.04	73.41	-17.15	56.34	-10.20	-17.36
DOX 0.9+NTR 20+TOB 0.2							
Median (n=32)	84.25	83.19	105.58	3.41	80.20	6.01	-8.73
Max	94.76	113.27	120.42	20.42	86.86	42.16	25.49
Min	72.51	65.04	100.20	-17.15	64.44	-1.93	-17.51
DOX 0.9+NTR 20+VAN 0.4							
Median (n=32)	84.25	83.19	99.60	3.41	76.38	1.58	-14.94
Max	94.76	113.27	123.10	20.42	106.28	59.41	38.44
Min	72.51	65.04	94.45	-17.15	63.45	-17.36	-26.56
ERY 0.07+FOX 0.7+NTR 20							
Median (n=32)	94.33	78.10	83.19	66.56	-4.67	56.19	-2.31
Max	117.27	125.01	113.27	71.64	7.43	81.60	9.93
Min	79.10	68.65	65.04	57.85	-13.46	28.08	-24.01
ERY 0.07+FUS 0.02+NTR 20							

Median (n=32)	94.33	94.12	83.19	48.14	-4.67	58.25	1.16
Max	117.27	107.80	113.27	63.49	7.43	89.03	30.82
Min	79.10	82.11	65.04	31.10	-13.46	37.74	-15.28
ERY 0.07+GEN 0.2+NTR 20							
Median (n=32)	94.33	102.73	83.19	103.35	-4.67	0.70	-6.85
Max	117.27	128.07	113.27	109.17	7.43	71.31	7.73
Min	79.10	95.16	65.04	92.86	-13.46	-18.54	-12.38
ERY 0.07+NTR 20+STR 2.5							
Median (n=32)	94.33	83.19	85.53	-4.67	61.93	-9.26	-11.74
Max	117.27	113.27	88.88	7.43	90.83	-6.05	8.32
Min	79.10	65.04	75.78	-13.46	44.37	-15.84	-20.15
ERY 0.07+NTR 20+TMP 5							
Median (n=32)	94.33	83.19	86.60	-4.67	74.67	1.96	-13.37
Max	117.27	113.27	99.83	7.43	81.42	17.63	10.62
Min	79.10	65.04	73.41	-13.46	71.53	-10.20	-17.04
ERY 0.07+NTR 20+TOB 0.2							
Median (n=32)	94.33	83.19	105.58	-4.67	96.39	6.01	-10.67
Max	117.27	113.27	120.42	7.43	100.63	42.16	-3.95
Min	79.10	65.04	100.20	-13.46	91.48	-1.93	-16.94

ERY 0.07+NTR 20+VAN 0.4							
Median (n=32)	94.33	83.19	99.60	-4.67	101.47	1.58	-5.91
Max	117.27	113.27	123.10	7.43	117.56	59.41	45.07
Min	79.10	65.04	94.45	-13.46	89.18	-17.36	-17.21
FOX 0.7+FUS 0.02+NTR 20							
Median (n=32)	78.10	94.12	83.19	67.29	56.19	58.25	11.42
Max	125.01	107.80	113.27	72.88	81.60	89.03	51.32
Min	68.65	82.11	65.04	57.89	28.08	37.74	-1.83
FOX 0.7+GEN 0.2+NTR 20							
Median (n=32)	78.10	102.73	83.19	84.75	56.19	0.70	7.75
Max	125.01	128.07	113.27	100.95	81.60	71.31	45.52
Min	68.65	95.16	65.04	78.45	28.08	-18.54	-7.82
FOX 0.7+NTR 20+STR 2.5							
Median (n=32)	78.10	83.19	85.53	56.19	61.11	-9.26	5.73
Max	125.01	113.27	88.88	81.60	78.69	-6.05	12.92
Min	68.65	65.04	75.78	28.08	44.45	-15.84	-16.62
FOX 0.7+NTR 20+TMP 5							
Median (n=32)	78.10	83.19	86.60	56.19	60.80	1.96	-1.19
Max	125.01	113.27	99.83	81.60	64.60	17.63	24.07

Min	68.65	65.04	73.41	28.08	57.02	-10.20	-11.71
FOX 0.7+NTR 20+TOB 0.2							
Median (n=32)	78.10	83.19	105.58	56.19	82.34	6.01	-5.02
Max	125.01	113.27	120.42	81.60	90.56	42.16	9.80
Min	68.65	65.04	100.20	28.08	76.53	-1.93	-14.35
FOX 0.7+NTR 20+VAN 0.4							
Median (n=32)	78.10	83.19	99.60	56.19	13.57	1.58	-4.85
Max	125.01	113.27	123.10	81.60	91.37	59.41	9.07
Min	68.65	65.04	94.45	28.08	-0.69	-17.36	-15.45
FUS 0.02+GEN 0.2+NTR 20							
Median (n=32)	94.12	102.73	83.19	85.56	58.25	0.70	31.32
Max	107.80	128.07	113.27	97.66	89.03	71.31	135.15
Min	82.11	95.16	65.04	58.83	37.74	-18.54	-8.08
FUS 0.02+NTR 20+STR 2.5							
Median (n=32)	94.12	83.19	85.53	58.25	55.38	-9.26	3.38
Max	107.80	113.27	88.88	89.03	103.03	-6.05	55.21
Min	82.11	65.04	75.78	37.74	37.01	-15.84	-12.22
FUS 0.02+NTR 20+TMP 5							
Median (n=32)	94.12	83.19	86.60	58.25	70.64	1.96	15.37

Max	107.80	113.27	99.83	89.03	83.59	17.63	41.50
Min	82.11	65.04	73.41	37.74	60.41	-10.20	-4.16
FUS 0.02+NTR 20+TOB 0.2							
Median (n=32)	94.12	83.19	105.58	58.25	83.88	6.01	5.30
Max	107.80	113.27	120.42	89.03	96.01	42.16	39.74
Min	82.11	65.04	100.20	37.74	62.74	-1.93	-1.41
FUS 0.02+NTR 20+VAN 0.4							
Median (n=32)	94.12	83.19	99.60	58.25	86.16	1.58	7.62
Max	107.80	113.27	123.10	89.03	104.11	59.41	41.67
Min	82.11	65.04	94.45	37.74	78.85	-17.36	-0.30
AMP 0.25+DOX 0.9+STR 2.5							
Median (n=16)	96.19	84.25	85.53	52.95	62.55	7.36	7.44
Max	103.71	94.76	88.88	109.43	94.99	78.81	94.35
Min	91.41	72.51	75.78	40.26	47.67	-11.02	-7.40
AMP 0.25+DOX 0.9+ERY 0.07							
Median (n=16)	96.19	84.25	94.33	52.95	87.85	7.67	14.66
Max	103.71	94.76	117.27	109.43	159.19	87.95	46.33
Min	91.41	72.51	79.10	40.26	82.92	-7.69	-0.45
AMP 0.25+GEN 0.2+STR 2.5							

Median (n=16)	96.19	102.73	85.53	100.76	62.55	59.83	24.05
Max	103.71	128.07	88.88	110.97	94.99	105.36	47.96
Min	91.41	95.16	75.78	95.72	47.67	47.68	-6.31
AMP 0.25+CHL 120+VAN 0.4							
Median (n=8)	96.19	99.22	99.60	53.91	44.95	68.45	4.62
Max	103.71	102.38	123.10	106.21	124.37	103.13	82.86
Min	91.41	92.59	94.45	-8.84	2.86	39.77	-8.67
AMP 0.25+CHL 120+STR 2.5							
Median (n=8)	96.19	99.22	85.53	53.91	62.55	56.26	5.67
Max	103.71	102.38	88.88	106.21	94.99	70.69	20.97
Min	91.41	92.59	75.78	-8.84	47.67	42.03	-13.31
AMP 0.25+ERY 0.07+VAN 0.4							
Median (n=16)	96.19	94.33	99.60	87.85	44.95	101.47	0.41
Max	103.71	117.27	123.10	159.19	124.37	117.56	110.64
Min	91.41	79.10	94.45	82.92	2.86	89.18	-14.00
AMP 0.25+ERY 0.07+STR 2.5							
Median (n=16)	96.19	94.33	85.53	87.85	62.55	61.93	11.17
Max	103.71	117.27	88.88	159.19	94.99	90.83	38.15
Min	91.41	79.10	75.78	82.92	47.67	44.37	1.58

CPR 0.15+GEN 0.2+STR 2.5							
Median (n=16)	91.80	102.73	85.53	88.22	74.67	59.83	26.45
Max	126.70	128.07	88.88	127.26	80.99	105.36	90.71
Min	80.38	95.16	75.78	66.70	57.38	47.68	8.70
CPR 0.15+STR 2.5+VAN 0.4							
Median (n=8)	91.80	85.53	99.60	74.67	72.41	63.50	21.80
Max	126.70	88.88	123.10	80.99	121.13	100.61	104.72
Min	80.38	75.78	94.45	57.38	67.69	21.07	-0.24
CHL 120+DOX 0.9+STR 2.5							
Median (n=8)	99.22	84.25	85.53	34.24	56.26	7.36	8.39
Max	102.38	94.76	88.88	58.47	70.69	78.81	14.18
Min	92.59	72.51	75.78	17.25	42.03	-11.02	3.60
CHL 120+DOX 0.9+ERY 0.07							
Median (n=16)	99.22	84.25	94.33	34.24	32.15	7.67	0.32
Max	102.38	94.76	117.27	58.47	47.70	87.95	13.23
Min	92.59	72.51	79.10	17.25	5.22	-7.69	-3.22
CHL 120+GEN 0.2+STR 2.5							
Median (n=8)	99.22	102.73	85.53	57.56	56.26	59.83	2.82
Max	102.38	128.07	88.88	89.63	70.69	105.36	6.49

Min	92.59	95.16	75.78	50.61	42.03	47.68	-0.77
CHL 120+CPR 0.15+STR 2.5							
Median (n=8)	99.22	91.80	85.53	56.18	56.26	74.67	30.45
Max	102.38	126.70	88.88	78.19	70.69	80.99	40.50
Min	92.59	80.38	75.78	43.89	42.03	57.38	22.60
AMP 0.25+CLI 0.04+VAN 0.4							
Median (n=8)	96.19	106.46	99.60	74.96	44.95	87.67	-5.15
Max	103.71	111.15	123.10	86.29	124.37	98.28	13.39
Min	91.41	96.85	94.45	62.59	2.86	77.72	-11.96
AMP 0.25+CLI 0.04+STR 2.5							
Median (n=8)	96.19	106.46	85.53	74.96	62.55	8.36	-9.88
Max	103.71	111.15	88.88	86.29	94.99	94.49	-6.62
Min	91.41	96.85	75.78	62.59	47.67	-6.91	-34.88
AMP 0.25+ERY 0.07+VAN 0.4							
Median (n=16)	96.19	78.10	99.60	70.30	44.95	13.57	-8.97
Max	103.71	125.01	123.10	77.13	124.37	91.37	19.74
Min	91.41	68.65	94.45	64.36	2.86	-0.69	-11.57
AMP 0.25+ERY 0.07+STR 2.5							
Median (n=16)	96.19	78.10	85.53	70.30	62.55	61.11	27.06

Max	103.71	125.01	88.88	77.13	94.99	78.69	43.48
Min	91.41	68.65	75.78	64.36	47.67	44.45	5.14
AMP 0.25+STR 2.5+VAN 0.4							
Median (n=8)	96.19	85.53	99.60	62.55	44.95	63.50	-5.62
Max	103.71	88.88	123.10	94.99	124.37	100.61	7.22
Min	91.41	75.78	94.45	47.67	2.86	21.07	-9.93
DOX 0.9+ERY 0.07+STR 2.5							
Median (n=16)	84.25	94.33	85.53	7.67	7.36	61.93	0.91
Max	94.76	117.27	88.88	87.95	78.81	90.83	10.74
Min	72.51	79.10	75.78	-7.69	-11.02	44.37	-5.15
ERY 0.07+GEN 0.2+STR 2.5							
Median (n=16)	94.33	102.73	85.53	103.35	61.93	59.83	13.33
Max	117.27	128.07	88.88	109.17	90.83	105.36	29.86
Min	79.10	95.16	75.78	92.86	44.37	47.68	3.28
CPR 0.15+ERY 0.07+STR 2.5							
Median (n=16)	91.80	94.33	85.53	73.29	74.67	61.93	61.70
Max	126.70	117.27	88.88	120.70	80.99	90.83	69.14
Min	80.38	79.10	75.78	63.13	57.38	44.37	57.81
DOX 0.9+GEN 0.2+STR 2.5							

Median (n=16)	84.25	102.73	85.53	86.06	7.36	59.83	-2.23
Max	94.76	128.07	88.88	113.82	78.81	105.36	12.76
Min	72.51	95.16	75.78	52.18	-11.02	47.68	-8.27
DOX 0.9+STR 2.5+VAN 0.4							
Median (n=8)	84.25	85.53	99.60	7.36	76.38	63.50	-3.56
Max	94.76	88.88	123.10	78.81	106.28	100.61	8.17
Min	72.51	75.78	94.45	-11.02	63.45	21.07	-6.65
CLI 0.04+DOX 0.9+STR 2.5							
Median (n=8)	106.46	84.25	85.53	34.12	8.36	7.36	5.92
Max	111.15	94.76	88.88	78.06	94.49	78.81	25.07
Min	96.85	72.51	75.78	21.22	-6.91	-11.02	1.33
CLI 0.04+DOX 0.9+ERY 0.07							
Median (n=16)	106.46	84.26	94.33	34.12	81.95	7.67	17.56
Max	111.15	94.76	117.27	78.06	113.78	87.95	29.02
Min	96.85	72.51	79.10	21.22	66.47	-7.69	12.11
CLI 0.04+GEN 0.2+STR 2.5							
Median (n=8)	106.46	102.73	85.53	73.76	8.36	59.83	4.87
Max	111.15	128.07	88.88	101.00	94.49	105.36	17.52
Min	96.85	95.16	75.78	-0.16	-6.91	47.68	-1.40

CLI 0.04+CPR 0.15+STR 2.5							
Median (n=8)	106.46	91.81	85.53	78.20	8.36	74.67	27.39
Max	111.15	126.70	88.88	85.92	94.49	80.99	40.08
Min	96.85	80.38	75.78	70.44	-6.91	57.38	7.40
DOX 0.9+FOX 0.7+STR 2.5							
Median (n=16)	84.25	78.10	85.53	60.25	7.36	61.11	4.35
Max	94.76	125.01	88.88	67.10	78.81	78.69	15.30
Min	72.51	68.65	75.78	55.61	-11.02	44.45	-2.55
DOX 0.9+ERY 0.07+FOX 0.7							
Median (n=16)	84.25	94.33	78.10	7.67	60.25	66.56	14.78
Max	94.76	117.27	125.01	87.95	67.10	71.64	25.44
Min	72.51	79.10	68.65	-7.69	55.61	57.85	8.76
AMP 0.25+CPR 0.15+VAN 0.4							
Median (n=8)	96.19	91.81	99.60	70.27	44.95	72.41	10.76
Max	103.71	126.70	123.10	108.11	124.37	121.13	17.10
Min	91.41	80.38	94.45	59.24	2.86	67.69	1.33
AMP 0.25+CPR 0.15+STR 2.5							
Median (n=16)	96.19	91.81	85.53	70.27	62.55	74.67	16.42
Max	103.71	126.70	88.88	108.11	94.99	80.99	112.12

Min	91.41	80.38	75.78	59.24	47.67	57.38	0.25
AMP 0.25+FUS 0.02+VAN 0.4							
Median (n=16)	96.19	94.12	99.60	83.58	44.95	86.16	9.41
Max	103.71	107.80	123.10	101.66	124.37	104.11	35.97
Min	91.41	82.11	94.45	74.06	2.86	78.85	-5.51
AMP 0.25+FUS 0.02+STR 2.5							
Median (n=16)	96.19	94.12	85.53	83.58	62.55	55.38	23.13
Max	103.71	107.80	88.88	101.66	94.99	103.03	44.69
Min	91.41	82.11	75.78	74.06	47.67	37.01	14.39
AMP 0.25+TMP 5+VAN 0.4							
Median (n=8)	96.19	86.60	99.60	83.21	44.95	84.53	11.50
Max	103.71	99.83	123.10	88.36	124.37	97.10	53.64
Min	91.41	73.41	94.45	78.31	2.86	79.82	-6.23
AMP 0.25+STR 2.5+TMP 5							
Median (n=8)	96.19	85.53	86.60	62.55	83.21	72.49	79.02
Max	103.71	88.88	99.83	94.99	88.36	134.21	103.76
Min	91.41	75.78	73.41	47.67	78.31	65.03	50.25
GEN 0.2+STR 2.5+TMP 5							
Median (n=8)	102.73	85.53	86.60	59.83	75.09	72.49	77.90

Max	128.07	88.88	99.83	105.36	86.05	134.21	85.80
Min	95.16	75.78	73.41	47.68	67.25	65.03	65.98
DOX 0.9+STR 2.5+TMP 5							
Median (n=8)	84.25	85.53	86.60	61.33	7.36	72.49	21.14
Max	94.76	88.88	99.83	72.45	78.81	134.21	25.04
Min	72.51	75.78	73.41	56.34	-11.02	65.03	11.68
DOX 0.9+ERY 0.07+TMP 5							
Median (n=16)	84.25	94.33	86.60	7.67	61.33	74.67	12.45
Max	94.76	117.27	99.83	87.95	72.45	81.42	19.68
Min	72.51	79.10	73.41	-7.69	56.34	71.53	2.18
CPR 0.15+STR 2.5+TMP 5							
Median (n=8)	91.81	85.53	86.60	74.67	59.55	79.69	70.24
Max	126.70	88.88	99.83	80.99	61.72	85.44	85.66
Min	80.38	75.78	73.41	57.38	57.91	66.76	63.64
FOX 0.7+GEN 0.2+STR 2.5							
Median (n=16)	78.10	102.73	85.53	84.75	61.11	59.83	44.57
Max	125.01	128.07	88.88	100.95	78.69	105.36	63.83
Min	68.65	95.16	75.78	78.45	44.45	47.68	-2.12
CPR 0.15+FOX 0.7+STR 2.5							

Median (n=16)	91.81	78.10	85.53	56.15	74.67	61.11	52.87
Max	126.70	125.01	88.88	61.36	80.99	78.69	76.51
Min	80.38	68.65	75.78	52.37	57.38	44.45	33.93
DOX 0.9+ERY 0.07+GEN 0.2							
Median (n=16)	84.25	94.33	102.73	7.67	86.06	103.35	17.58
Max	94.76	117.27	128.07	87.95	113.82	109.17	28.70
Min	72.51	79.10	95.16	-7.69	52.18	92.86	12.64
CPR 0.15+DOX 0.9+STR 2.5							
Median (n=16)	91.81	84.25	85.53	76.87	74.67	7.36	12.17
Max	126.70	94.76	88.88	91.17	80.99	78.81	60.89
Min	80.38	72.51	75.78	67.23	57.38	-11.02	-7.50
CPR 0.15+DOX 0.9+ERY 0.07							
Median (n=16)	91.81	84.25	94.33	76.87	73.29	7.67	33.67
Max	126.70	94.76	117.27	91.17	120.70	87.95	94.31
Min	80.38	72.51	79.10	67.23	63.13	-7.69	28.37
DOX 0.9+FUS 0.02+STR 2.5							
Median (n=16)	84.25	94.12	85.53	34.19	7.36	34.19	14.85
Max	94.76	107.80	88.88	49.15	78.81	49.15	19.46
Min	72.51	82.11	75.78	25.03	-11.02	25.03	6.09

DOX 0.9+ERY 0.07+FUS 0.02							
Median (n=16)	84.25	94.33	94.12	7.67	34.19	48.14	13.50
Max	94.76	117.27	107.80	87.95	49.15	63.49	27.47
Min	72.51	79.10	82.11	-7.69	25.03	31.10	8.56
AMP 0.25+DOX 0.9+VAN 0.4							
Median (n=8)	96.19	84.25	99.60	52.95	44.95	76.38	8.80
Max	103.71	94.76	123.10	109.43	124.37	106.28	100.10
Min	91.41	72.51	94.45	40.26	2.86	63.45	-3.60
AMP 0.25+GEN 0.2+VAN 0.4							
Median (n=8)	96.19	102.73	99.60	100.76	44.95	92.85	61.90
Max	103.71	128.07	123.10	110.97	124.37	114.28	86.62
Min	91.41	95.16	94.45	95.72	2.86	81.87	13.85
AMP 0.3+TOB 0.2+VAN 0.5							
Median (n=8)	96.19	105.58	99.60	100.04	44.95	99.17	50.55
Max	103.71	120.42	123.10	107.78	124.37	125.65	111.16
Min	91.41	100.20	94.45	98.39	2.86	92.28	26.71
AMP 0.3+STR 2.8+TOB 0.2							
Median (n=8)	96.19	85.53	105.58	62.55	100.04	47.05	25.74
Max	103.71	88.88	120.42	94.99	107.78	71.87	132.86

Min	91.41	75.78	100.20	47.67	98.39	39.96	9.86
GEN 0.25+STR 2.8+TOB 0.2							
Median (n=8)	102.73	85.53	105.58	59.83	90.97	47.05	30.16
Max	128.07	88.88	120.42	105.36	100.32	71.87	41.68
Min	95.16	75.78	100.20	47.68	79.00	39.96	24.66
DOX 1+STR 2.8+TOB 0.2							
Median (n=8)	84.25	85.53	105.58	7.36	80.20	47.05	11.81
Max	94.76	88.88	120.42	78.81	86.86	71.87	126.01
Min	72.51	75.78	100.20	-11.02	64.44	39.96	3.62
DOX 1+ERY 0.08+TOB 0.2							
Median (n=16)	84.25	94.33	105.58	7.67	80.20	96.39	20.66
Max	94.76	117.27	120.42	87.95	86.86	100.63	88.05
Min	72.51	79.10	100.20	-7.69	64.44	91.48	12.30
CPR 0.2+STR 2.8+TOB 0.2							
Median (n=8)	91.81	105.58	85.53	74.67	79.67	47.05	31.74
Max	126.70	120.42	88.88	80.99	83.27	71.87	50.34
Min	80.38	100.20	75.78	57.38	74.63	39.96	-24.06
FUS 0.02+GEN 0.2+STR 2.5							
Median (n=16)	94.12	102.73	85.53	85.56	55.38	59.83	6.40

Max	107.80	128.07	88.88	97.66	103.03	105.36	20.07
Min	82.11	95.16	75.78	58.83	37.01	47.68	-34.07
CPR 0.15+FUS 0.02 +STR 2.5							
Median (n=16)	91.80	94.12	85.53	78.98	74.67	55.38	49.55
Max	126.70	107.80	88.88	86.48	80.99	103.03	131.18
Min	80.38	82.11	75.78	74.43	57.38	37.01	34.82
GEN 0.2+STR 2.5+VAN 0.4							
Median (n=8)	102.73	85.53	99.60	59.83	92.85	63.50	29.35
Max	128.07	88.88	123.10	105.36	114.28	100.61	57.13
Min	95.16	75.78	94.45	47.68	81.87	21.07	7.87
DOX 0.9+ERY 0.07+VAN 0.4							
Median (n=17)	84.25	94.33	99.60	7.67	76.38	101.47	22.48
Max	94.76	117.27	123.10	87.95	106.28	117.56	91.90
Min	72.51	79.10	94.45	-7.69	63.45	89.18	8.09
***E. coli* CFT073**							
AMP 3.56+CHL 88.4+CLI 13.3							
Median (n=32)	79.51	79.93	85.73	41.62	56.99	64.60	36.88
Max	98.05	89.78	93.78	49.28	63.88	70.79	46.18

Min	64.70	76.34	82.45	31.78	28.94	62.23	31.65
AMP 3.56+CHL 88.4+CPR 0.04							
Median (n=32)	79.51	79.93	68.75	41.62	42.37	59.26	32.90
Max	98.05	89.78	81.60	49.28	51.22	66.26	34.96
Min	64.70	76.34	61.44	31.78	34.59	50.17	26.25
AMP 3.56+CHL 88.4+DOX 0.66							
Median (n=32)	79.51	79.93	66.74	41.62	23.32	53.28	7.29
Max	98.05	89.78	74.60	49.28	33.73	58.97	11.22
Min	64.70	76.34	59.94	31.78	19.49	46.10	3.61
AMP 3.56+CHL 88.4+ERY 3.62							
Median (n=32)	79.51	79.93	102.45	41.62	74.31	88.74	43.26
Max	98.05	89.78	112.12	49.28	80.19	99.59	53.49
Min	64.70	76.34	92.26	31.78	61.29	83.72	27.67
AMP 3.56+CHL 88.4+FOX 2.25							
Median (n=32)	79.51	79.93	80.36	41.62	8.33	31.79	3.77
Max	98.05	89.78	94.71	49.28	25.57	45.60	20.28
Min	64.70	76.34	35.04	31.78	3.56	18.40	0.13

AMP 3.56+CHL 88.4+FUS 38							
Median (n=32)	79.51	79.93	68.42	41.62	23.62	48.88	3.47
Max	98.05	89.78	95.37	49.28	47.35	52.19	22.09
Min	64.70	76.34	32.92	31.78	-7.95	44.28	-0.19
AMP 3.56+CHL 88.4+GEN 4.4							
Median (n=32)	79.51	79.93	73.83	41.62	50.59	66.50	34.46
Max	98.05	89.78	86.03	49.28	60.20	69.73	36.58
Min	64.70	76.34	60.41	31.78	15.88	38.56	29.39
AMP 3.56+CHL 88.4+NTR 16.5							
Median (n=32)	79.51	79.93	77.44	41.62	48.96	63.48	42.91
Max	98.05	89.78	103.36	49.28	55.06	69.02	51.87
Min	64.70	76.34	64.31	31.78	44.84	61.83	37.83
AMP 3.56+CHL 88.4+STR 8.47							
Median (n=32)	79.51	79.93	57.18	41.62	23.08	57.68	18.44
Max	98.05	89.78	77.02	49.28	34.32	65.61	22.50
Min	64.70	76.34	42.55	31.78	18.49	44.75	15.21
AMP 3.56+CHL 88.4+TMP 0.35							

Median (n=32)	79.51	79.93	80.68	41.62	89.39	64.13	63.95
Max	98.05	89.78	95.28	49.28	107.54	70.64	74.61
Min	64.70	76.34	69.35	31.78	87.48	55.75	61.34
AMP 3.56+CHL 88.4+TOB 4.1							
Median (n=32)	79.51	79.93	71.10	41.62	47.33	73.13	32.60
Max	98.05	89.78	84.19	49.28	50.75	90.95	36.77
Min	64.70	76.34	24.81	31.78	38.11	41.54	29.92
AMP 3.56+CHL 88.4+VAN 80							
Median (n=32)	79.51	79.93	55.55	41.62	9.58	56.42	12.87
Max	98.05	89.78	69.28	49.28	32.33	64.37	18.26
Min	64.70	76.34	22.28	31.78	-5.26	38.52	5.48
AMP 3.56+NTR 16.5+STR 8.47							
Median (n=32)	79.51	77.44	57.18	48.96	23.08	-1.04	-1.08
Max	98.05	103.36	77.02	55.06	34.32	6.07	12.92
Min	64.70	64.31	42.55	44.84	18.49	-5.76	-6.65
AMP 3.56+NTR 16.5+TMP 0.35							
Median (n=32)	79.51	77.44	80.68	48.96	89.39	59.58	45.98
Max	98.05	103.36	95.28	55.06	107.54	76.21	51.32

Min	64.70	64.31	69.35	44.84	87.48	39.33	20.27
AMP 3.56+NTR 16.5+TOB 4.1							
Median (n=32)	79.51	77.44	71.10	48.96	47.33	62.07	22.69
Max	98.05	103.36	84.19	55.06	50.75	71.72	25.39
Min	64.70	64.31	24.81	44.84	38.11	52.22	16.88
AMP 3.56+NTR 16.5+VAN 80							
Median (n=32)	79.51	77.44	55.55	48.96	9.58	4.46	0.04
Max	98.05	103.36	69.28	55.06	32.33	7.53	37.12
Min	64.70	64.31	22.28	44.84	-5.26	1.83	-1.92
AMP 3.56+CLI 13.3+CPR 0.04							
Median (n=32)	79.51	85.73	68.75	56.99	42.37	48.51	26.73
Max	98.05	93.78	81.60	63.88	51.22	55.00	37.17
Min	64.70	82.45	61.44	28.94	34.59	45.35	-17.55
AMP 3.56+CLI 13.3+DOX 0.66							
Median (n=32)	79.51	85.73	66.74	56.99	23.32	55.62	37.45
Max	98.05	93.78	74.60	63.88	33.73	57.88	42.93
Min	64.70	82.45	59.94	28.94	19.49	33.60	27.55
AMP 3.56+CLI 13.3+ERY 3.62							
Median (n=32)	79.51	85.73	102.45	56.99	74.31	80.27	53.97

Max	98.05	93.78	112.12	63.88	80.19	84.78	80.58
Min	64.70	82.45	92.26	28.94	61.29	74.41	42.29
AMP 3.56+CLI 13.3+FOX 2.25							
Median (n=32)	79.51	85.73	80.36	56.99	8.33	55.83	8.21
Max	98.05	93.78	94.71	63.88	25.57	65.15	10.34
Min	64.70	82.45	35.04	28.94	3.56	34.19	-1.23
AMP 3.56+CLI 13.3+FUS 38							
Median (n=32)	79.51	85.73	68.42	56.99	23.62	52.71	30.43
Max	98.05	93.78	95.37	63.88	47.35	58.40	44.20
Min	64.70	82.45	32.92	28.94	-7.95	49.34	25.59
AMP 3.56+CLI 13.3+GEN 4.4							
Median (n=32)	79.51	85.73	73.83	56.99	50.59	52.38	38.23
Max	98.05	93.78	86.03	63.88	60.20	62.10	43.86
Min	64.70	82.45	60.41	28.94	15.88	38.88	29.55
AMP 3.56+CLI 13.3+NTR 16.5							
Median (n=32)	79.51	85.73	77.44	56.99	48.96	65.25	47.77
Max	98.05	93.78	103.36	63.88	55.06	82.74	54.45
Min	64.70	82.45	64.31	28.94	44.84	34.35	37.01
AMP 3.56+CLI 13.3+STR 8.47							

Median (n=32)	79.51	85.73	57.18	56.99	23.08	50.17	17.22
Max	98.05	93.78	77.02	63.88	34.32	66.01	25.66
Min	64.70	82.45	42.55	28.94	18.49	28.12	10.39
AMP 3.56+CLI 13.3+TMP 0.35							
Median (n=32)	79.51	85.73	80.68	56.99	89.39	65.29	53.09
Max	98.05	93.78	95.28	63.88	107.54	76.70	58.22
Min	64.70	82.45	69.35	28.94	87.48	51.25	29.64
AMP 3.56+CLI 13.3+TOB 4.1							
Median (n=32)	79.51	85.73	71.10	56.99	47.33	64.55	31.03
Max	98.05	93.78	84.19	63.88	50.75	70.55	39.02
Min	64.70	82.45	24.81	28.94	38.11	42.49	25.54
AMP 3.56+CLI 13.3+VAN 80							
Median (n=32)	79.51	85.73	55.55	56.99	9.58	54.43	12.52
Max	98.05	93.78	69.28	63.88	32.33	62.33	19.18
Min	64.70	82.45	22.28	28.94	-5.26	10.37	4.66
AMP 3.56+STR 8.47+TMP 0.35							
Median (n=32)	79.51	57.18	80.68	23.08	89.39	46.20	43.52
Max	98.05	77.02	95.28	34.32	107.54	59.14	53.39
Min	64.70	42.55	69.35	18.49	87.48	39.27	18.79

AMP 3.56+STR 8.47+TOB 4.1							
Median (n=32)	79.51	57.18	71.10	23.08	47.33	56.45	13.50
Max	98.05	77.02	84.19	34.32	50.75	65.02	33.13
Min	64.70	42.55	24.81	18.49	38.11	50.40	0.50
AMP 3.56+STR 8.47+VAN 80							
Median (n=32)	79.51	57.18	55.55	23.08	9.58	35.57	3.25
Max	98.05	77.02	69.28	34.32	32.33	39.85	12.62
Min	64.70	42.55	22.28	18.49	-5.26	10.50	-0.14
AMP 3.56+CPR 0.04+DOX 0.66							
Median (n=32)	79.51	68.75	66.74	42.37	23.32	40.45	28.82
Max	98.05	81.60	74.60	51.22	33.73	47.06	39.31
Min	64.70	61.44	59.94	34.59	19.49	25.69	19.60
AMP 3.56+CPR 0.04+ERY 3.62							
Median (n=32)	79.51	68.75	102.45	42.37	74.31	63.47	36.38
Max	98.05	81.60	112.12	51.22	80.19	70.41	55.62
Min	64.70	61.44	92.26	34.59	61.29	59.44	7.77
AMP 3.56+CPR 0.04+FOX 2.25							
Median (n=32)	79.51	68.75	80.36	42.37	8.33	28.68	15.28

Max	98.05	81.60	94.71	51.22	25.57	35.14	20.42
Min	64.70	61.44	35.04	34.59	3.56	23.57	6.53
AMP 3.56+CPR 0.04+FUS 38							
Median (n=32)	79.51	68.75	68.42	42.37	23.62	61.41	20.84
Max	98.05	81.60	95.37	51.22	47.35	63.42	24.40
Min	64.70	61.44	32.92	34.59	-7.95	55.16	15.12
AMP 3.56+CPR 0.04+GEN 4.4							
Median (n=32)	79.51	68.75	73.83	42.37	50.59	43.85	20.60
Max	98.05	81.60	86.03	51.22	60.20	55.82	23.83
Min	64.70	61.44	60.41	34.59	15.88	40.21	10.32
AMP 3.56+CPR 0.04+NTR 16.5							
Median (n=32)	79.51	68.75	77.44	42.37	48.96	40.46	20.60
Max	98.05	81.60	103.36	51.22	55.06	47.50	35.81
Min	64.70	61.44	64.31	34.59	44.84	34.96	17.15
AMP 3.56+CPR 0.04+STR 8.47							
Median (n=32)	79.51	68.75	57.18	42.37	23.08	46.40	19.17
Max	98.05	81.60	77.02	51.22	34.32	50.59	27.10
Min	64.70	61.44	42.55	34.59	18.49	32.43	15.36
AMP 3.56+CPR 0.04+TMP							

0.35							
Median (n=32)	79.51	68.75	80.68	42.37	89.39	38.56	37.04
Max	98.05	81.60	95.28	51.22	107.54	50.13	48.04
Min	64.70	61.44	69.35	34.59	87.48	7.17	30.23
AMP 3.56+CPR 0.04+TOB 4.1							
Median (n=32)	79.51	68.75	71.10	42.37	47.33	56.65	24.74
Max	98.05	81.60	84.19	51.22	50.75	59.64	34.63
Min	64.70	61.44	24.81	34.59	38.11	7.91	11.65
AMP 3.56+CPR 0.04+VAN 80							
Median (n=32)	79.51	68.75	55.55	42.37	9.58	27.31	11.80
Max	98.05	81.60	69.28	51.22	32.33	35.95	22.37
Min	64.70	61.44	22.28	34.59	-5.26	7.10	-0.38
AMP 3.56+TMP 0.35+TOB 4.1							
Median (n=32)	79.51	80.68	71.10	89.39	47.33	49.81	51.01
Max	98.05	95.28	84.19	107.54	50.75	65.61	60.42
Min	64.70	69.35	24.81	87.48	38.11	10.01	42.23
AMP 3.56+TMP 0.35+VAN 80							
Median (n=32)	79.51	80.68	55.55	89.39	9.58	32.45	24.40
Max	98.05	95.28	69.28	107.54	32.33	69.49	31.04

Min	64.70	69.35	22.28	87.48	-5.26	23.81	18.51
AMP 3.56+DOX 0.66+ERY 3.62							
Median (n=32)	79.51	66.74	102.45	23.32	74.31	38.07	22.70
Max	98.05	74.60	112.12	33.73	80.19	53.96	37.06
Min	64.70	59.94	92.26	19.49	61.29	32.72	12.79
AMP 3.56+DOX 0.66+FOX 2.25							
Median (n=32)	79.51	66.74	80.36	23.32	8.33	14.61	-0.63
Max	98.05	74.60	94.71	33.73	25.57	22.40	16.23
Min	64.70	59.94	35.04	19.49	3.56	3.46	-4.53
AMP 3.56+DOX 0.66+FUS 38							
Median (n=32)	79.51	66.74	68.42	23.32	23.62	21.11	5.78
Max	98.05	74.60	95.37	33.73	47.35	27.77	14.04
Min	64.70	59.94	32.92	19.49	-7.95	4.66	2.80
AMP 3.56+DOX 0.66+GEN 4.4							
Median (n=32)	79.51	66.74	73.83	23.32	50.59	47.29	9.29
Max	98.05	74.60	86.03	33.73	60.20	52.81	18.43
Min	64.70	59.94	60.41	19.49	15.88	42.22	4.84

AMP 3.56+DOX 0.66+NTR 16.5							
Median (n=32)	79.51	66.74	77.44	23.32	48.96	53.09	59.61
Max	98.05	74.60	103.36	33.73	55.06	57.29	68.80
Min	64.70	59.94	64.31	19.49	44.84	40.44	49.38
AMP 3.56+DOX 0.66+STR 8.47							
Median (n=32)	79.51	66.74	57.18	23.32	23.08	21.72	12.26
Max	98.05	74.60	77.02	33.73	34.32	44.29	15.45
Min	64.70	59.94	42.55	19.49	18.49	6.56	2.62
AMP 3.56+DOX 0.66+TMP 0.35							
Median (n=32)	79.51	66.74	80.68	23.32	89.39	54.35	32.48
Max	98.05	74.60	95.28	33.73	107.54	70.73	38.78
Min	64.70	59.94	69.35	19.49	87.48	30.60	22.60
AMP 3.56+DOX 0.66+TOB 4.1							
Median (n=32)	79.51	66.74	71.10	23.32	47.33	54.27	15.55
Max	98.05	74.60	84.19	33.73	50.75	58.95	30.03
Min	64.70	59.94	24.81	19.49	38.11	36.63	9.30
AMP 3.56+DOX 0.66+VAN 80							

Median (n=32)	79.51	66.74	55.55	23.32	9.58	11.12	11.06
Max	98.05	74.60	69.28	33.73	32.33	18.14	15.84
Min	64.70	59.94	22.28	19.49	-5.26	-12.51	7.58
AMP 3.56+ERY 3.62+FOX 2.25							
Median (n=32)	79.51	102.45	80.36	74.31	8.33	62.01	8.50
Max	98.05	112.12	94.71	80.19	25.57	71.19	59.32
Min	64.70	92.26	35.04	61.29	3.56	51.25	-5.11
AMP 3.56+ERY 3.62+FUS 38							
Median (n=32)	79.51	102.45	68.42	74.31	23.62	52.31	21.28
Max	98.05	112.12	95.37	80.19	47.35	65.73	35.40
Min	64.70	92.26	32.92	61.29	-7.95	36.58	1.39
AMP 3.56+ERY 3.62+GEN 4.4							
Median (n=32)	79.51	102.45	73.83	74.31	50.59	67.28	44.63
Max	98.05	112.12	86.03	80.19	60.20	78.35	46.09
Min	64.70	92.26	60.41	61.29	15.88	21.17	38.20
AMP 3.56+ERY 3.62+NTR 16.5							
Median (n=32)	79.51	102.45	77.44	74.31	48.96	84.06	57.77

Max	98.05	112.12	103.36	80.19	55.06	87.25	68.03
Min	64.70	92.26	64.31	61.29	44.84	79.31	47.83
AMP 3.56+ERY 3.62+STR 8.47							
Median (n=32)	79.51	102.45	57.18	74.31	23.08	55.73	24.03
Max	98.05	112.12	77.02	80.19	34.32	64.57	49.30
Min	64.70	92.26	42.55	61.29	18.49	34.13	20.43
AMP 3.56+ERY 3.62+TMP 0.35							
Median (n=32)	79.51	102.45	80.68	74.31	89.39	80.64	85.20
Max	98.05	112.12	95.28	80.19	107.54	90.64	92.23
Min	64.70	92.26	69.35	61.29	87.48	28.20	76.24
AMP 3.56+ERY 3.62+TOB 4.1							
Median (n=32)	79.51	102.45	71.10	74.31	47.33	76.22	37.67
Max	98.05	112.12	84.19	80.19	50.75	84.26	40.19
Min	64.70	92.26	24.81	61.29	38.11	69.30	22.14
AMP 3.56+ERY 3.62+VAN 80							
Median (n=32)	79.51	102.45	55.55	74.31	9.58	71.42	25.63
Max	98.05	112.12	69.28	80.19	32.33	75.27	31.55
Min	64.70	92.26	22.28	61.29	-5.26	29.07	15.60

DOX 0.66+ERY 3.62+FOX 2.25							
Median (n=8)	66.74	102.45	80.36	38.07	14.61	62.01	15.76
Max	74.60	112.12	94.71	53.96	22.40	71.19	23.40
Min	59.94	92.26	35.04	32.72	3.46	51.25	11.04
DOX 0.66+ERY 3.62+FUS 38							
Median (n=16)	66.74	102.45	68.42	38.07	21.11	52.31	12.40
Max	74.60	112.12	95.37	53.96	27.77	65.73	16.48
Min	59.94	92.26	32.92	32.72	4.66	36.58	3.46
DOX 0.66+ERY 3.62+GEN 4.4							
Median (n=16)	66.74	102.45	73.83	38.07	47.29	67.28	33.31
Max	74.60	112.12	86.03	53.96	52.81	78.35	35.72
Min	59.94	92.26	60.41	32.72	42.22	21.17	26.92
DOX 0.66+ERY 3.62+NTR 16.5							
Median (n=16)	66.74	102.45	77.44	38.07	53.09	84.06	48.06
Max	74.60	112.12	103.36	53.96	57.29	87.25	52.63
Min	59.94	92.26	64.31	32.72	40.44	79.31	38.07
DOX 0.66+ERY 3.62+STR 8.47							

Median (n=16)	66.74	102.45	57.18	38.07	21.72	55.73	9.93
Max	74.60	112.12	77.02	53.96	44.29	64.57	19.79
Min	59.94	92.26	42.55	32.72	6.56	34.13	3.04
DOX 0.66+ERY 3.62+TMP 0.35							
Median (n=16)	66.74	102.45	80.68	38.07	54.35	80.64	37.82
Max	74.60	112.12	95.28	53.96	70.73	90.64	51.36
Min	59.94	92.26	69.35	32.72	30.60	28.20	31.54
DOX 0.66+ERY 3.62+TOB 4.1							
Median (n=32)	66.74	102.45	71.10	38.07	54.27	76.22	41.00
Max	74.60	112.12	84.19	53.96	58.95	84.26	46.53
Min	59.94	92.26	24.81	32.72	36.63	69.30	36.39
DOX 0.66+ERY 3.62+VAN 80							
Median (n=32)	66.74	102.45	55.55	38.07	11.12	71.42	-1.75
Max	74.60	112.12	69.28	53.96	18.14	75.27	2.35
Min	59.94	92.26	22.28	32.72	-12.51	29.07	-6.56
AMP 3.56+FOX 2.25+FUS 38							
Median (n=32)	79.51	80.36	68.42	8.33	23.62	19.20	0.53
Max	98.05	94.71	95.37	25.57	47.35	40.44	6.67

Min	64.70	35.04	32.92	3.56	-7.95	12.82	-10.87
AMP 3.56+FOX 2.25+GEN 4.4							
Median (n=32)	79.51	80.36	73.83	8.33	50.59	29.60	7.86
Max	98.05	94.71	86.03	25.57	60.20	33.33	13.32
Min	64.70	35.04	60.41	3.56	15.88	25.33	-5.02
AMP 3.56+FOX 2.25+NTR 16.5							
Median (n=32)	79.51	80.36	77.44	8.33	48.96	46.66	17.07
Max	98.05	94.71	103.36	25.57	55.06	57.85	19.15
Min	64.70	35.04	64.31	3.56	44.84	34.72	10.90
AMP 3.56+FOX 2.25+STR 8.47							
Median (n=32)	79.51	80.36	57.18	8.33	23.08	30.49	0.59
Max	98.05	94.71	77.02	25.57	34.32	48.88	6.41
Min	64.70	35.04	42.55	3.56	18.49	-5.18	-7.12
AMP 3.56+FOX 2.25+TMP 0.35							
Median (n=32)	79.51	80.36	80.68	8.33	89.39	77.11	42.89
Max	98.05	94.71	95.28	25.57	107.54	85.42	50.96
Min	64.70	35.04	69.35	3.56	87.48	38.63	35.31

AMP 3.56+FOX 2.25+TOB 4.1							
Median (n=32)	79.51	80.36	71.10	8.33	47.33	34.80	4.74
Max	98.05	94.71	84.19	25.57	50.75	37.59	7.72
Min	64.70	35.04	24.81	3.56	38.11	31.22	0.92
AMP 3.56+FOX 2.25+VAN 80							
Median (n=32)	79.51	80.36	55.55	8.33	9.58	19.46	1.07
Max	98.05	94.71	69.28	25.57	32.33	63.31	5.64
Min	64.70	35.04	22.28	3.56	-5.26	-6.12	-3.16
DOX 0.66+FOX 2.25+FUS 38							
Median (n=16)	66.74	80.36	68.42	14.61	21.11	19.20	1.13
Max	74.60	94.71	95.37	22.40	27.77	40.44	9.35
Min	59.94	35.04	32.92	3.46	4.66	12.82	-16.88
DOX 0.66+FOX 2.25+GEN 4.4							
Median (n=16)	66.74	80.36	73.83	14.61	47.29	29.60	21.77
Max	74.60	94.71	86.03	22.40	52.81	33.33	29.16
Min	59.94	35.04	60.41	3.46	42.22	25.33	5.78
DOX 0.66+FOX 2.25+NTR 16.5							
Median (n=16)	66.74	80.36	77.44	14.61	53.09	46.66	25.25

Max	74.60	94.71	103.36	22.40	57.29	57.85	28.94
Min	59.94	35.04	64.31	3.46	40.44	34.72	15.66
DOX 0.66+FOX 2.25+STR 8.47							
Median (n=16)	66.74	80.36	57.18	14.61	21.72	30.49	0.28
Max	74.60	94.71	77.02	22.40	44.29	48.88	4.22
Min	59.94	35.04	42.55	3.46	6.56	-5.18	-4.31
DOX 0.66+FOX 2.25+TMP 0.35							
Median (n=16)	66.74	80.36	80.68	14.61	54.35	77.11	26.29
Max	74.60	94.71	95.28	22.40	70.73	85.42	31.70
Min	59.94	35.04	69.35	3.46	30.60	38.63	18.46
DOX 0.66+FOX 2.25+TOB 4.1							
Median (n=32)	66.74	80.36	71.10	14.61	54.27	34.80	25.10
Max	74.60	94.71	84.19	22.40	58.95	37.59	28.36
Min	59.94	35.04	24.81	3.46	36.63	31.22	19.20
DOX 0.66+FOX 2.25+VAN 80							
Median (n=32)	66.74	80.36	55.55	14.61	11.12	19.46	2.36
Max	74.60	94.71	69.28	22.40	18.14	63.31	4.28
Min	59.94	35.04	22.28	3.46	-12.51	-6.12	-0.09

AMP 3.56+FUS 38+GEN 4.4							
Median (n=32)	79.51	68.42	73.83	23.62	50.59	50.98	13.77
Max	98.05	95.37	86.03	47.35	60.20	61.69	29.42
Min	64.70	32.92	60.41	-7.95	15.88	41.91	0.47
AMP 3.56+FUS 38+NTR 16.5							
Median (n=32)	79.51	68.42	77.44	23.62	48.96	72.18	58.41
Max	98.05	95.37	103.36	47.35	55.06	80.76	75.53
Min	64.70	32.92	64.31	-7.95	44.84	49.42	51.78
AMP 3.56+FUS 38+STR 8.47							
Median (n=32)	79.51	68.42	57.18	23.62	23.08	50.71	3.97
Max	98.05	95.37	77.02	47.35	34.32	66.73	9.16
Min	64.70	32.92	42.55	-7.95	18.49	39.23	-5.47
AMP 3.56+FUS 38+TMP 0.35							
Median (n=32)	79.51	68.42	80.68	23.62	89.39	46.93	39.12
Max	98.05	95.37	95.28	47.35	107.54	65.37	43.20
Min	64.70	32.92	69.35	-7.95	87.48	7.87	19.95
AMP 3.56+FUS 38+TOB 4.1							
Median (n=32)	79.51	68.42	71.10	23.62	47.33	61.10	6.56
Max	98.05	95.37	84.19	47.35	50.75	65.07	16.07

Min	64.70	32.92	24.81	-7.95	38.11	44.23	-2.55
AMP 3.56+FUS 38+VAN 80							
Median (n=32)	79.51	68.42	55.55	23.62	9.58	33.16	0.87
Max	98.05	95.37	69.28	47.35	32.33	37.55	8.50
Min	64.70	32.92	22.28	-7.95	-5.26	-11.89	-12.04
DOX 0.66+FUS 38+GEN 4.4							
Median (n=16)	66.74	68.42	73.83	21.11	47.29	50.98	14.95
Max	74.60	95.37	86.03	27.77	52.81	61.69	44.39
Min	59.94	32.92	60.41	4.66	42.22	41.91	-1.76
DOX 0.66+FUS 38+NTR 16.5							
Median (n=16)	66.74	68.42	77.44	21.11	53.09	72.18	58.18
Max	74.60	95.37	103.36	27.77	57.29	80.76	64.87
Min	59.94	32.92	64.31	4.66	40.44	49.42	54.66
DOX 0.66+FUS 38+STR 8.47							
Median (n=16)	66.74	68.42	57.18	21.11	21.72	50.71	9.32
Max	74.60	95.37	77.02	27.77	44.29	66.73	21.83
Min	59.94	32.92	42.55	4.66	6.56	39.23	4.32
DOX 0.66+FUS 38+TMP 0.35							
Median (n=16)	66.74	68.42	80.68	21.11	54.35	46.93	21.61

Max	74.60	95.37	95.28	27.77	70.73	65.37	32.41
Min	59.94	32.92	69.35	4.66	30.60	7.87	14.57
DOX 0.66+FUS 38+TOB 4.1							
Median (n=32)	66.74	68.42	71.10	21.11	54.27	61.10	14.08
Max	74.60	95.37	84.19	27.77	58.95	65.07	15.16
Min	59.94	32.92	24.81	4.66	36.63	44.23	2.58
DOX 0.66+FUS 38+VAN 80							
Median (n=32)	66.74	68.42	55.55	21.11	11.12	33.16	5.42
Max	74.60	95.37	69.28	27.77	18.14	37.55	11.44
Min	59.94	32.92	22.28	4.66	-12.51	-11.89	1.74
AMP 3.56+GEN 4.4+NTR 16.5							
Median (n=32)	79.51	73.83	77.44	50.59	48.96	59.31	46.08
Max	98.05	86.03	103.36	60.20	55.06	64.50	48.53
Min	64.70	60.41	64.31	15.88	44.84	51.01	43.27
AMP 3.56+GEN 4.4+STR 8.47							
Median (n=32)	79.51	73.83	57.18	50.59	23.08	51.96	24.45
Max	98.05	86.03	77.02	60.20	34.32	66.18	31.58
Min	64.70	60.41	42.55	15.88	18.49	33.29	19.16
AMP 3.56+GEN 4.4+TMP 0.35							

Median (n=32)	79.51	73.83	80.68	50.59	89.39	49.65	56.56
Max	98.05	86.03	95.28	60.20	107.54	72.97	62.26
Min	64.70	60.41	69.35	15.88	87.48	34.41	36.04
AMP 3.56+GEN 4.4+TOB 4.1							
Median (n=32)	79.51	73.83	71.10	50.59	47.33	59.75	24.73
Max	98.05	86.03	84.19	60.20	50.75	76.66	33.43
Min	64.70	60.41	24.81	15.88	38.11	36.72	-4.89
AMP 3.56+GEN 4.4+VAN 80							
Median (n=32)	79.51	73.83	55.55	50.59	9.58	54.44	14.25
Max	98.05	86.03	69.28	60.20	32.33	68.70	23.93
Min	64.70	60.41	22.28	15.88	-5.26	13.32	2.72
DOX 0.66+GEN 4.4+NTR 16.5							
Median (n=16)	66.74	73.83	77.44	47.29	53.09	59.31	43.46
Max	74.60	86.03	103.36	52.81	57.29	64.50	65.47
Min	59.94	60.41	64.31	42.22	40.44	51.01	39.15
DOX 0.66+GEN 4.4+STR 8.47							
Median (n=16)	66.74	73.83	57.18	47.29	21.72	51.96	8.31
Max	74.60	86.03	77.02	52.81	44.29	66.18	11.95
Min	59.94	60.41	42.55	42.22	6.56	33.29	4.10

DOX 0.66+GEN 4.4+TMP 0.35							
Median (n=16)	66.74	73.83	80.68	47.29	54.35	49.65	35.81
Max	74.60	86.03	95.28	52.81	70.73	72.97	49.45
Min	59.94	60.41	69.35	42.22	30.60	34.41	11.91
DOX 0.66+GEN 4.4+TOB 4.1							
Median (n=32)	66.74	73.83	71.10	47.29	54.27	59.75	37.40
Max	74.60	86.03	84.19	52.81	58.95	76.66	47.69
Min	59.94	60.41	24.81	42.22	36.63	36.72	27.05
DOX 0.66+GEN 4.4+VAN 80							
Median (n=32)	66.74	73.83	55.55	47.29	11.12	54.44	8.12
Max	74.60	86.03	69.28	52.81	18.14	68.70	15.17
Min	59.94	60.41	22.28	42.22	-12.51	13.32	-17.53
CHL 88.4+CLI 13.3+CPR 0.04							
Median (n=16)	79.93	85.73	68.75	64.60	59.26	48.51	53.01
Max	89.78	93.78	81.60	70.79	66.26	55.00	65.81
Min	76.34	82.45	61.44	62.23	50.17	45.35	42.26
CHL 88.4+CLI 13.3+DOX 0.66							
Median (n=16)	79.93	85.73	66.74	64.60	53.28	55.62	51.03
Max	89.78	93.78	74.60	70.79	58.97	57.88	61.01

Min	76.34	82.45	59.94	62.23	46.10	33.60	36.21
CHL 88.4+CLI 13.3+ERY 3.62							
Median (n=16)	79.93	85.73	102.45	64.60	88.74	80.27	72.08
Max	89.78	93.78	112.12	70.79	99.59	84.78	87.96
Min	76.34	82.45	92.26	62.23	83.72	74.41	51.39
CHL 88.4+CLI 13.3+FUS 38							
Median (n=16)	79.93	85.73	68.42	64.60	48.88	52.71	43.84
Max	89.78	93.78	95.37	70.79	52.19	58.40	54.28
Min	76.34	82.45	32.92	62.23	44.28	49.34	32.90
CHL 88.4+CLI 13.3+GEN 4.4							
Median (n=16)	79.93	85.73	73.83	64.60	66.50	52.38	55.98
Max	89.78	93.78	86.03	70.79	69.73	62.10	69.80
Min	76.34	82.45	60.41	62.23	38.56	38.88	43.52
CHL 88.4+CLI 13.3+NTR 16.5							
Median (n=16)	79.93	85.73	77.44	64.60	63.48	65.25	55.44
Max	89.78	93.78	103.36	70.79	69.02	82.74	69.38
Min	76.34	82.45	64.31	62.23	61.83	34.35	29.21
CHL 88.4+CLI 13.3+STR 8.47							
Median (n=16)	79.93	85.73	57.18	64.60	57.68	50.17	46.28

Max	89.78	93.78	77.02	70.79	65.61	66.01	53.15
Min	76.34	82.45	42.55	62.23	44.75	28.12	42.60
CHL 88.4+CLI 13.3+TMP 0.35							
Median (n=8)	79.93	85.73	80.68	64.60	64.13	65.29	55.34
Max	89.78	93.78	95.28	70.79	70.64	76.70	61.46
Min	76.34	82.45	69.35	62.23	55.75	51.25	51.62
CHL 88.4+CLI 13.3+TOB 4.1							
Median (n=32)	79.93	85.73	71.10	64.60	73.13	64.55	55.04
Max	89.78	93.78	84.19	70.79	90.95	70.55	65.89
Min	76.34	82.45	24.81	62.23	41.54	42.49	51.18
CHL 88.4+CLI 13.3+VAN 80							
Median (n=32)	79.93	85.73	55.55	64.60	56.42	54.43	31.23
Max	89.78	93.78	69.28	70.79	64.37	62.33	36.45
Min	76.34	82.45	22.28	62.23	38.52	10.37	25.34
CPR 0.04+GEN 4.4+NTR 16.5							
Median (n=16)	68.75	73.83	77.44	43.85	40.46	59.31	34.95
Max	81.60	86.03	103.36	55.82	47.50	64.50	62.63
Min	61.44	60.41	64.31	40.21	34.96	51.01	28.53
CPR 0.04+GEN 4.4+STR 8.47							

Median (n=16)	68.75	73.83	57.18	43.85	46.40	51.96	39.04
Max	81.60	86.03	77.02	55.82	50.59	66.18	45.54
Min	61.44	60.41	42.55	40.21	32.43	33.29	6.18
CPR 0.04+GEN 4.4+TMP 0.35							
Median (n=16)	68.75	73.83	80.68	43.85	38.56	49.65	36.49
Max	81.60	86.03	95.28	55.82	50.13	72.97	39.80
Min	61.44	60.41	69.35	40.21	7.17	34.41	30.71
CPR 0.04+GEN 4.4+TOB 4.1							
Median (n=32)	68.75	73.83	71.10	43.85	56.65	59.75	45.17
Max	81.60	86.03	84.19	55.82	59.64	76.66	51.61
Min	61.44	60.41	24.81	40.21	7.91	36.72	35.40
CPR 0.04+GEN 4.4+VAN 80							
Median (n=32)	68.75	73.83	55.55	43.85	27.31	54.44	19.36
Max	81.60	86.03	69.28	55.82	35.95	68.70	26.76
Min	61.44	60.41	22.28	40.21	7.10	13.32	11.47
CHL 88.4+CPR 0.04+DOX 0.66							
Median (n=16)	79.93	68.75	66.74	59.26	53.28	40.45	33.03
Max	89.78	81.60	74.60	66.26	58.97	47.06	41.65
Min	76.34	61.44	59.94	50.17	46.10	25.69	23.41

CHL 88.4+CPR 0.04+ERY 3.62							
Median (n=16)	79.93	68.75	102.45	59.26	88.74	63.47	55.40
Max	89.78	81.60	112.12	66.26	99.59	70.41	66.80
Min	76.34	61.44	92.26	50.17	83.72	59.44	25.95
CHL 88.4+CPR 0.04+FOX 2.25							
Median (n=16)	79.93	68.75	80.36	59.26	31.79	28.68	19.33
Max	89.78	81.60	94.71	66.26	45.60	35.14	26.21
Min	76.34	61.44	35.04	50.17	18.40	23.57	-4.03
CHL 88.4+CPR 0.04+FUS 38							
Median (n=16)	79.93	68.75	68.42	59.26	48.88	61.41	37.34
Max	89.78	81.60	95.37	66.26	52.19	63.42	42.02
Min	76.34	61.44	32.92	50.17	44.28	55.16	26.06
CHL 88.4+CPR 0.04+GEN 4.4							
Median (n=16)	79.93	68.75	73.83	59.26	66.50	43.85	47.81
Max	89.78	81.60	86.03	66.26	69.73	55.82	56.45
Min	76.34	61.44	60.41	50.17	38.56	40.21	2.09
CHL 88.4+CPR 0.04+NTR 16.5							
Median (n=16)	79.93	68.75	77.44	59.26	63.48	40.46	45.32
Max	89.78	81.60	103.36	66.26	69.02	47.50	56.09

Min	76.34	61.44	64.31	50.17	61.83	34.96	29.63
CHL 88.4+CPR 0.04+STR 8.47							
Median (n=16)	79.93	68.75	57.18	59.26	57.68	46.40	42.94
Max	89.78	81.60	77.02	66.26	65.61	50.59	51.10
Min	76.34	61.44	42.55	50.17	44.75	32.43	30.00
CHL 88.4+CPR 0.04+TMP 0.35							
Median (n=16)	79.93	68.75	80.68	59.26	64.13	38.56	46.30
Max	89.78	81.60	95.28	66.26	70.64	50.13	47.09
Min	76.34	61.44	69.35	50.17	55.75	7.17	40.50
CHL 88.4+CPR 0.04+TOB 4.1							
Median (n=32)	79.93	68.75	71.10	59.26	73.13	56.65	51.47
Max	89.78	81.60	84.19	66.26	90.95	59.64	56.42
Min	76.34	61.44	24.81	50.17	41.54	7.91	47.66
CHL 88.4+CPR 0.04+VAN 80							
Median (n=32)	79.93	68.75	55.55	59.26	56.42	27.31	19.22
Max	89.78	81.60	69.28	66.26	64.37	35.95	28.52
Min	76.34	61.44	22.28	50.17	38.52	7.10	3.85
CHL 88.4+FOX 2.25+FUS 38							
Median (n=16)	79.93	80.36	68.43	31.79	48.88	19.20	10.14

Max	89.78	94.71	95.37	45.60	52.19	40.44	24.94
Min	76.34	35.04	32.92	18.40	44.28	12.82	-1.04
CHL 88.4+FOX 2.25+GEN 4.4							
Median (n=16)	79.93	80.36	73.83	31.79	66.50	29.60	18.58
Max	89.78	94.71	86.03	45.60	69.73	33.33	26.71
Min	76.34	35.04	60.41	18.40	38.56	25.33	9.59
CHL 88.4+FOX 2.25+NTR 16.5							
Median (n=16)	79.93	80.36	77.44	31.79	63.48	46.66	26.49
Max	89.78	94.71	103.36	45.60	69.02	57.85	41.04
Min	76.34	35.04	64.31	18.40	61.83	34.72	11.98
CHL 88.4+FOX 2.25+STR 8.47							
Median (n=16)	79.93	80.36	57.18	31.79	57.68	30.49	15.73
Max	89.78	94.71	77.02	45.60	65.61	48.88	21.74
Min	76.34	35.04	42.55	18.40	44.75	-5.18	2.85
CHL 88.4+FOX 2.25+TMP 0.35							
Median (n=8)	79.93	80.36	80.68	31.79	64.13	77.11	42.05
Max	89.78	94.71	95.28	45.60	70.64	85.42	58.10
Min	76.34	35.04	69.35	18.40	55.75	38.63	33.54
CHL 88.4+FOX 2.25+TOB 4.1							

Median (n=32)	79.93	80.36	71.10	31.79	73.13	34.80	19.27
Max	89.78	94.71	84.19	45.60	90.95	37.59	29.62
Min	76.34	35.04	24.81	18.40	41.54	31.22	1.03
CHL 88.4+FOX 2.25+VAN 80							
Median (n=32)	79.93	80.36	55.55	31.79	56.42	19.46	10.72
Max	89.78	94.71	69.28	45.60	64.37	63.31	18.69
Min	76.34	35.04	22.28	18.40	38.52	-6.12	5.01
ERY 3.62+FOX 2.25+FUS 38							
Median (n=16)	102.45	80.36	68.43	62.01	52.31	19.20	20.21
Max	112.12	94.71	95.37	71.19	65.73	40.44	29.13
Min	92.26	35.04	32.92	51.25	36.58	12.82	15.71
ERY 3.62+FOX 2.25+GEN 4.4							
Median (n=16)	102.45	80.36	73.83	62.01	67.28	29.60	26.73
Max	112.12	94.71	86.03	71.19	78.35	33.33	62.28
Min	92.26	35.04	60.41	51.25	21.17	25.33	19.34
ERY 3.62+FOX 2.25+NTR 16.5							
Median (n=16)	102.45	80.36	77.44	62.01	84.06	46.66	33.74
Max	112.12	94.71	103.36	71.19	87.25	57.85	50.75
Min	92.26	35.04	64.31	51.25	79.31	34.72	22.64

CPR 0.04+NTR 16.5+STR 8.47							
Median (n=16)	68.75	77.44	57.18	40.46	46.40	-1.04	7.75
Max	81.60	103.36	77.02	47.50	50.59	6.07	13.98
Min	61.44	64.31	42.55	34.96	32.43	-5.76	-10.66
CPR 0.04+NTR 16.5+TMP 0.35							
Median (n=16)	68.75	77.44	80.68	40.46	38.56	59.58	30.43
Max	81.60	103.36	95.28	47.50	50.13	76.21	34.80
Min	61.44	64.31	69.35	34.96	7.17	39.33	16.73
CPR 0.04+NTR 16.5+TOB 4.1							
Median (n=32)	68.75	77.44	71.10	40.46	56.65	62.07	40.50
Max	81.60	103.36	84.19	47.50	59.64	71.72	50.56
Min	61.44	64.31	24.81	34.96	7.91	52.22	11.06
CPR 0.04+NTR 16.5+VAN 80							
Median (n=32)	68.75	77.44	55.55	40.46	27.31	4.46	5.89
Max	81.60	103.36	69.28	47.50	35.95	7.53	17.17
Min	61.44	64.31	22.28	34.96	7.10	1.83	-0.77
CHL 88.4+DOX 0.66+ERY 3.62							
Median (n=16)	79.93	66.74	102.45	53.28	88.74	38.07	47.31

Max	89.78	74.60	112.12	58.97	99.59	53.96	62.24
Min	76.34	59.94	92.26	46.10	83.72	32.72	36.77
CHL 88.4+DOX 0.66+FOX 2.25							
Median (n=16)	79.93	66.74	80.36	53.28	31.79	14.61	12.53
Max	89.78	74.60	94.71	58.97	45.60	22.40	23.36
Min	76.34	59.94	35.04	46.10	18.40	3.46	5.35
CHL 88.4+DOX 0.66+FUS 38							
Median (n=16)	79.93	66.74	68.43	53.28	48.88	21.11	13.10
Max	89.78	74.60	95.37	58.97	52.19	27.77	16.24
Min	76.34	59.94	32.92	46.10	44.28	4.66	5.69
CHL 88.4+DOX 0.66+GEN 4.4							
Median (n=16)	79.93	66.74	73.83	53.28	66.50	47.29	40.14
Max	89.78	74.60	86.03	58.97	69.73	52.81	46.80
Min	76.34	59.94	60.41	46.10	38.56	42.22	27.24
CHL 88.4+DOX 0.66+NTR 16.5							
Median (n=16)	79.93	66.74	77.44	53.28	63.48	53.09	41.45
Max	89.78	74.60	103.36	58.97	69.02	57.29	48.55

Min	76.34	59.94	64.31	46.10	61.83	40.44	19.24
CHL 88.4+DOX 0.66+STR 8.47							
Median (n=16)	79.93	66.74	57.18	53.28	57.68	21.72	20.18
Max	89.78	74.60	77.02	58.97	65.61	44.29	28.39
Min	76.34	59.94	42.55	46.10	44.75	6.56	-0.95
CHL 88.4+DOX 0.66+TMP 0.35							
Median (n=16)	79.93	66.74	80.68	53.28	64.13	54.35	40.30
Max	89.78	74.60	95.28	58.97	70.64	70.73	50.49
Min	76.34	59.94	69.35	46.10	55.75	30.60	38.65
CHL 88.4+DOX 0.66+TOB 4.1							
Median (n=32)	79.93	66.74	71.10	53.28	73.13	54.27	48.26
Max	89.78	74.60	84.19	58.97	90.95	58.95	55.05
Min	76.34	59.94	24.81	46.10	41.54	36.63	39.03
CHL 88.4+DOX 0.66+VAN 80							
Median (n=32)	79.93	66.74	55.55	53.28	56.42	11.12	13.29
Max	89.78	74.60	69.28	58.97	64.37	18.14	27.15
Min	76.34	59.94	22.28	46.10	38.52	-12.51	6.61
CHL 88.4+ERY 3.62+FOX 2.25							

Median (n=16)	79.93	102.45	80.36	88.74	31.79	62.01	24.64
Max	89.78	112.12	94.71	99.59	45.60	71.19	54.00
Min	76.34	92.26	35.04	83.72	18.40	51.25	6.91
CHL 88.4+ERY 3.62+FUS 38							
Median (n=16)	79.93	102.45	68.43	88.74	48.88	52.31	52.68
Max	89.78	112.12	95.37	99.59	52.19	65.73	62.12
Min	76.34	92.26	32.92	83.72	44.28	36.58	42.49
CHL 88.4+ERY 3.62+GEN 4.4							
Median (n=16)	79.93	102.45	73.83	88.74	66.50	67.28	71.63
Max	89.78	112.12	86.03	99.59	69.73	78.35	76.43
Min	76.34	92.26	60.41	83.72	38.56	21.17	32.61
CHL 88.4+ERY 3.62+NTR 16.5							
Median (n=16)	79.93	102.45	77.44	88.74	63.48	84.06	63.84
Max	89.78	112.12	103.36	99.59	69.02	87.25	70.35
Min	76.34	92.26	64.31	83.72	61.83	79.31	53.36
CHL 88.4+ERY 3.62+STR 8.47							
Median (n=16)	79.93	102.45	57.18	88.74	57.68	55.73	64.41
Max	89.78	112.12	77.02	99.59	65.61	64.57	66.93
Min	76.34	92.26	42.55	83.72	44.75	34.13	59.24

CHL 88.4+ERY 3.62+TMP 0.35							
Median (n=8)	79.93	102.45	80.68	88.74	64.13	80.64	56.20
Max	89.78	112.12	95.28	99.59	70.64	90.64	67.84
Min	76.34	92.26	69.35	83.72	55.75	28.20	33.17
CHL 88.4+ERY 3.62+TOB 4.1							
Median (n=32)	79.93	102.45	71.10	88.74	73.13	76.22	76.16
Max	89.78	112.12	84.19	99.59	90.95	84.26	90.95
Min	76.34	92.26	24.81	83.72	41.54	69.30	65.97
CHL 88.4+ERY 3.62+VAN 80							
Median (n=32)	79.93	102.45	55.55	88.74	56.42	71.42	55.07
Max	89.78	112.12	69.28	99.59	64.37	75.27	57.13
Min	76.34	92.26	22.28	83.72	38.52	29.07	47.03
CPR 0.04+STR 8.47+TMP 0.35							
Median (n=16)	68.75	57.18	80.68	46.40	38.56	46.20	38.23
Max	81.60	77.02	95.28	50.59	50.13	59.14	49.09
Min	61.44	42.55	69.35	32.43	7.17	39.27	-0.90
CPR 0.04+TMP 0.35+TOB 4.1							
Median (n=32)	68.75	80.68	71.10	38.56	56.65	49.81	47.55
Max	81.60	95.28	84.19	50.13	59.64	65.61	52.87

Min	61.44	69.35	24.81	7.17	7.91	10.01	6.95
CPR 0.04+TMP 0.35+VAN 80							
Median (n=32)	68.75	80.68	55.55	38.56	27.31	32.45	6.02
Max	81.60	95.28	69.28	50.13	35.95	69.49	14.26
Min	61.44	69.35	22.28	7.17	7.10	23.81	-15.03
CPR 0.04+STR 8.47+TOB 4.1							
Median (n=32)	68.75	57.18	71.10	46.40	56.65	56.45	47.73
Max	81.60	77.02	84.19	50.59	59.64	65.02	54.03
Min	61.44	42.55	24.81	32.43	7.91	50.40	43.67
CPR 0.04+STR 8.47+VAN 80							
Median (n=32)	68.75	57.18	55.55	46.40	27.31	35.57	13.79
Max	81.60	77.02	69.28	50.59	35.95	39.85	20.76
Min	61.44	42.55	22.28	32.43	7.10	10.50	-2.38
CHL 88.4+CLI 13.3+FOX 2.25							
Median (n=16)	79.93	85.73	80.36	64.60	31.79	55.83	19.80
Max	89.78	93.78	94.71	70.79	45.60	65.15	26.95
Min	76.34	82.45	35.04	62.23	18.40	34.19	10.53
CLI 13.3+CPR 0.04+GEN 4.4							
Median (n=16)	85.73	68.75	73.83	48.51	52.38	43.85	48.79

Max	93.78	81.60	86.03	55.00	62.10	55.82	51.81
Min	82.45	61.44	60.41	45.35	38.88	40.21	40.49
CLI 13.3+CPR 0.04+NTR 16.5							
Median (n=16)	85.73	68.75	77.44	48.51	65.25	40.46	43.95
Max	93.78	81.60	103.36	55.00	82.74	47.50	57.18
Min	82.45	61.44	64.31	45.35	34.35	34.96	35.41
CLI 13.3+CPR 0.04+STR 8.47							
Median (n=16)	85.73	68.75	57.18	48.51	50.17	46.40	38.41
Max	93.78	81.60	77.02	55.00	66.01	50.59	41.37
Min	82.45	61.44	42.55	45.35	28.12	32.43	33.26
CLI 13.3+CPR 0.04+TMP 0.35							
Median (n=16)	85.73	68.75	80.68	48.51	65.29	38.56	31.30
Max	93.78	81.60	95.28	55.00	76.70	50.13	36.37
Min	82.45	61.44	69.35	45.35	51.25	7.17	26.21
CLI 13.3+CPR 0.04+TOB 4.1							
Median (n=32)	85.73	68.75	71.10	48.51	64.55	56.65	44.58
Max	93.78	81.60	84.19	55.00	70.55	59.64	52.28
Min	82.45	61.44	24.81	45.35	42.49	7.91	24.97
CLI 13.3+CPR 0.04+VAN 80							

Median (n=32)	85.73	68.75	55.55	48.51	54.43	27.31	19.99
Max	93.78	81.60	69.28	55.00	62.33	35.95	28.44
Min	82.45	61.44	22.28	45.35	10.37	7.10	9.47
CLI 13.3+FUS 38+GEN 4.4							
Median (n=16)	85.73	68.43	73.83	52.71	52.38	50.98	49.48
Max	93.78	95.37	86.03	58.40	62.10	61.69	59.11
Min	82.45	32.92	60.41	49.34	38.88	41.91	47.78
CLI 13.3+FUS 38+NTR 16.5							
Median (n=16)	85.73	68.43	77.44	52.71	65.25	72.18	62.49
Max	93.78	95.37	103.36	58.40	82.74	80.76	69.30
Min	82.45	32.92	64.31	49.34	34.35	49.42	59.35
CLI 13.3+FUS 38+STR 8.47							
Median (n=16)	85.73	68.43	57.18	52.71	50.17	50.71	47.29
Max	93.78	95.37	77.02	58.40	66.01	66.73	50.64
Min	82.45	32.92	42.55	49.34	28.12	39.23	30.29
CLI 13.3+FUS 38+TMP 0.35							
Median (n=16)	85.73	68.43	80.68	52.71	65.29	46.93	48.94
Max	93.78	95.37	95.28	58.40	76.70	65.37	58.71
Min	82.45	32.92	69.35	49.34	51.25	7.87	44.07

ERY 3.62+FOX 2.25+STR 8.47							
Median (n=16)	102.45	80.36	57.18	62.01	55.73	30.49	18.63
Max	112.12	94.71	77.02	71.19	64.57	48.88	29.28
Min	92.26	35.04	42.55	51.25	34.13	-5.18	11.04
ERY 3.62+FOX 2.25+TMP 0.35							
Median (n=8)	102.45	80.36	80.68	62.01	80.64	77.11	73.31
Max	112.12	94.71	95.28	71.19	90.64	85.42	81.23
Min	92.26	35.04	69.35	51.25	28.20	38.63	64.26
ERY 3.62+FOX 2.25+TOB 4.1							
Median (n=32)	102.45	80.36	71.10	62.01	76.22	34.80	29.69
Max	112.12	94.71	84.19	71.19	84.26	37.59	38.07
Min	92.26	35.04	24.81	51.25	69.30	31.22	26.31
ERY 3.62+FOX 2.25+VAN 80							
Median (n=32)	102.45	80.36	55.55	62.01	71.42	19.46	34.69
Max	112.12	94.71	69.28	71.19	75.27	63.31	38.01
Min	92.26	35.04	22.28	51.25	29.07	-6.12	29.25
CHL 88.4+FUS 38+GEN 4.4							
Median (n=16)	79.93	68.42	73.83	48.88	66.50	50.98	40.40
Max	89.78	95.37	86.03	52.19	69.73	61.69	50.19

Min	76.34	32.92	60.41	44.28	38.56	41.91	34.10
CHL 88.4+FUS 38+NTR 16.5							
Median (n=16)	79.93	68.42	77.44	48.88	63.48	72.18	46.71
Max	89.78	95.37	103.36	52.19	69.02	80.76	58.33
Min	76.34	32.92	64.31	44.28	61.83	49.42	33.92
CHL 88.4+FUS 38+STR 8.47							
Median (n=16)	79.93	68.42	57.18	48.88	57.68	50.71	24.68
Max	89.78	95.37	77.02	52.19	65.61	66.73	28.94
Min	76.34	32.92	42.55	44.28	44.75	39.23	19.71
CHL 88.4+FUS 38+TMP 0.35							
Median (n=16)	79.93	68.42	80.68	48.88	64.13	46.93	40.10
Max	89.78	95.37	95.28	52.19	70.64	65.37	46.13
Min	76.34	32.92	69.35	44.28	55.75	7.87	35.13
CHL 88.4+FUS 38+TOB 4.1							
Median (n=32)	79.93	68.42	71.10	48.88	73.13	61.10	38.48
Max	89.78	95.37	84.19	52.19	90.95	65.07	47.92
Min	76.34	32.92	24.81	44.28	41.54	44.23	36.48
CHL 88.4+FUS 38+VAN 80							
Median (n=32)	79.93	68.42	55.55	48.88	56.42	33.16	2.80

Max	89.78	95.37	69.28	52.19	64.37	37.55	6.44
Min	76.34	32.92	22.28	44.28	38.52	-11.89	-2.89
ERY 3.62+FUS 38+GEN 4.4							
Median (n=16)	102.45	68.42	73.83	52.31	67.28	50.98	59.48
Max	112.12	95.37	86.03	65.73	78.35	61.69	64.88
Min	92.26	32.92	60.41	36.58	21.17	41.91	48.20
ERY 3.62+FUS 38+NTR 16.5							
Median (n=16)	102.45	68.42	77.44	52.31	84.06	72.18	64.53
Max	112.12	95.37	103.36	65.73	87.25	80.76	82.53
Min	92.26	32.92	64.31	36.58	79.31	49.42	50.40
ERY 3.62+FUS 38+STR 8.47							
Median (n=16)	102.45	68.42	57.18	52.31	55.73	50.71	51.15
Max	112.12	95.37	77.02	65.73	64.57	66.73	58.06
Min	92.26	32.92	42.55	36.58	34.13	39.23	42.18
ERY 3.62+FUS 38+TMP 0.35							
Median (n=16)	102.45	68.42	80.68	52.31	80.64	46.93	46.24
Max	112.12	95.37	95.28	65.73	90.64	65.37	65.14
Min	92.26	32.92	69.35	36.58	28.20	7.87	17.25
ERY 3.62+FUS 38+TOB 4.1							

Median (n=32)	102.45	68.42	71.10	52.31	76.22	61.10	61.02
Max	112.12	95.37	84.19	65.73	84.26	65.07	68.05
Min	92.26	32.92	24.81	36.58	69.30	44.23	49.29
ERY 3.62+FUS 38+VAN 80							
Median (n=32)	102.45	68.42	55.55	52.31	71.42	33.16	28.44
Max	112.12	95.37	69.28	65.73	75.27	37.55	38.76
Min	92.26	32.92	22.28	36.58	29.07	-11.89	20.26
CHL 88.4+GEN 4.4+NTR 16.5							
Median (n=16)	79.93	73.83	77.44	66.50	63.48	59.31	53.72
Max	89.78	86.03	103.36	69.73	69.02	64.50	60.26
Min	76.34	60.41	64.31	38.56	61.83	51.01	43.72
CHL 88.4+GEN 4.4+STR 8.47							
Median (n=16)	79.93	73.83	57.18	66.50	57.68	51.96	50.84
Max	89.78	86.03	77.02	69.73	65.61	66.18	62.21
Min	76.34	60.41	42.55	38.56	44.75	33.29	42.63
CHL 88.4+GEN 4.4+TMP 0.35							
Median (n=16)	79.93	73.83	80.68	66.50	64.13	49.65	48.73
Max	89.78	86.03	95.28	69.73	70.64	72.97	62.78
Min	76.34	60.41	69.35	38.56	55.75	34.41	31.88

CHL 88.4+GEN 4.4+TOB 4.1							
Median (n=32)	79.93	73.83	71.10	66.50	73.13	59.75	52.41
Max	89.78	86.03	84.19	69.73	90.95	76.66	59.22
Min	76.34	60.41	24.81	38.56	41.54	36.72	41.13
CHL 88.4+GEN 4.4+VAN 80							
Median (n=32)	79.93	73.83	55.55	66.50	56.42	54.44	41.21
Max	89.78	86.03	69.28	69.73	64.37	68.70	51.65
Min	76.34	60.41	22.28	38.56	38.52	13.32	35.84
ERY 3.62+GEN 4.4+NTR 16.5							
Median (n=16)	102.45	73.83	77.44	67.28	84.06	59.31	69.07
Max	112.12	86.03	103.36	78.35	87.25	64.50	76.87
Min	92.26	60.41	64.31	21.17	79.31	51.01	60.96
ERY 3.62+GEN 4.4+STR 8.47							
Median (n=16)	102.45	73.83	57.18	67.28	55.73	51.96	57.37
Max	112.12	86.03	77.02	78.35	64.57	66.18	61.76
Min	92.26	60.41	42.55	21.17	34.13	33.29	52.99
ERY 3.62+GEN 4.4+TMP 0.35							
Median (n=16)	102.45	73.83	80.68	67.28	80.64	49.65	57.61
Max	112.12	86.03	95.28	78.35	90.64	72.97	72.30

Min	92.26	60.41	69.35	21.17	28.20	34.41	35.26
ERY 3.62+GEN 4.4+TOB 4.1							
Median (n=32)	102.45	73.83	71.10	67.28	76.22	59.75	52.56
Max	112.12	86.03	84.19	78.35	84.26	76.66	71.37
Min	92.26	60.41	24.81	21.17	69.30	36.72	18.86
ERY 3.62+GEN 4.4+VAN 80							
Median (n=32)	102.45	73.83	55.55	67.28	71.42	54.44	45.85
Max	112.12	86.03	69.28	78.35	75.27	68.70	55.89
Min	92.26	60.41	22.28	21.17	29.07	13.32	31.49
CHL 88.4+NTR 16.5+STR 8.47							
Median (n=16)	79.93	77.44	57.18	63.48	57.68	-1.04	7.52
Max	89.78	103.36	77.02	69.02	65.61	6.07	11.70
Min	76.34	64.31	42.55	61.83	44.75	-5.76	0.53
CHL 88.4+NTR 16.5+TMP 0.35							
Median (n=16)	79.93	77.44	80.68	63.48	64.13	59.58	54.17
Max	89.78	103.36	95.28	69.02	70.64	76.21	61.40
Min	76.34	64.31	69.35	61.83	55.75	39.33	47.40
CHL 88.4+NTR 16.5+TOB 4.1							
Median (n=32)	79.93	77.44	71.10	63.48	73.13	62.07	43.82

Max	89.78	103.36	84.19	69.02	90.95	71.72	58.19
Min	76.34	64.31	24.81	61.83	41.54	52.22	24.02
CHL 88.4+NTR 16.5+VAN 80							
Median (n=32)	79.93	77.44	55.55	63.48	56.42	4.46	6.46
Max	89.78	103.36	69.28	69.02	64.37	7.53	17.22
Min	76.34	64.31	22.28	61.83	38.52	1.83	-10.51
ERY 3.62+NTR 16.5+STR 8.47							
Median (n=16)	102.45	77.44	57.18	84.06	55.73	-1.04	6.93
Max	112.12	103.36	77.02	87.25	64.57	6.07	11.29
Min	92.26	64.31	42.55	79.31	34.13	-5.76	-1.05
ERY 3.62+NTR 16.5+TMP 0.35							
Median (n=16)	102.45	77.44	80.68	84.06	80.64	59.58	64.89
Max	112.12	103.36	95.28	87.25	90.64	76.21	74.20
Min	92.26	64.31	69.35	79.31	28.20	39.33	34.79
ERY 3.62+NTR 16.5+TOB 4.1							
Median (n=32)	102.45	77.44	71.10	84.06	76.22	62.07	67.63
Max	112.12	103.36	84.19	87.25	84.26	71.72	74.34
Min	92.26	64.31	24.81	79.31	69.30	52.22	51.57
ERY 3.62+NTR 16.5+VAN 80							

Median (n=32)	102.45	77.44	55.55	84.06	71.42	4.46	0.99
Max	112.12	103.36	69.28	87.25	75.27	7.53	11.99
Min	92.26	64.31	22.28	79.31	29.07	1.83	-16.03
CLI 13.3+CPR 0.04+DOX 0.66							
Median (n=8)	85.73	68.75	66.74	48.51	55.62	40.45	47.44
Max	93.78	81.60	74.60	55.00	57.88	47.06	48.60
Min	82.45	61.44	59.94	45.35	33.60	25.69	33.64
CLI 13.3+CPR 0.04+ERY 3.62							
Median (n=8)	85.73	68.75	102.45	48.51	80.27	63.47	48.26
Max	93.78	81.60	112.12	55.00	84.78	70.41	54.18
Min	82.45	61.44	92.26	45.35	74.41	59.44	42.76
CLI 13.3+CPR 0.04+FOX 2.25							
Median (n=8)	85.73	68.75	80.36	48.51	28.68	28.68	19.24
Max	93.78	81.60	94.71	55.00	35.14	35.14	24.01
Min	82.45	61.44	35.04	45.35	23.57	23.57	12.48
CLI 13.3+CPR 0.04+FUS 38							
Median (n=16)	85.73	68.75	68.42	48.51	52.71	61.41	43.19
Max	93.78	81.60	95.37	55.00	58.40	63.42	52.39
Min	82.45	61.44	32.92	45.35	49.34	55.16	24.12

CPR 0.04+DOX 0.66+NTR 16.5							
Median (n=16)	68.75	66.74	77.44	40.45	40.46	53.09	41.60
Max	81.60	74.60	103.36	47.06	47.50	57.29	47.36
Min	61.44	59.94	64.31	25.69	34.96	40.44	29.14
CPR 0.04+DOX 0.66+STR 8.47							
Median (n=16)	68.75	66.74	57.18	40.45	46.40	21.72	15.51
Max	81.60	74.60	77.02	47.06	50.59	44.29	25.43
Min	61.44	59.94	42.55	25.69	32.43	6.56	8.37
CPR 0.04+DOX 0.66+TMP 0.35							
Median (n=16)	68.75	66.74	80.68	40.45	38.56	54.35	37.19
Max	81.60	74.60	95.28	47.06	50.13	70.73	41.69
Min	61.44	59.94	69.35	25.69	7.17	30.60	24.87
CPR 0.04+DOX 0.66+TOB 4.1							
Median (n=32)	68.75	66.74	71.10	40.45	56.65	54.27	39.14
Max	81.60	74.60	84.19	47.06	59.64	58.95	44.27
Min	61.44	59.94	24.81	25.69	7.91	36.63	25.10
CPR 0.04+DOX 0.66+VAN 80							
Median (n=32)	68.75	66.74	55.55	40.45	27.31	11.12	2.15

Max	81.60	74.60	69.28	47.06	35.95	18.14	10.06
Min	61.44	59.94	22.28	25.69	7.10	-12.51	-7.79
DOX 0.66+NTR 16.5+STR 8.47							
Median (n=16)	66.74	77.44	57.18	53.09	21.72	-1.04	5.08
Max	74.60	103.36	77.02	57.29	44.29	6.07	20.03
Min	59.94	64.31	42.55	40.44	6.56	-5.76	0.71
DOX 0.66+NTR 16.5+TMP 0.35							
Median (n=16)	66.74	77.44	80.68	53.09	54.35	59.58	47.41
Max	74.60	103.36	95.28	57.29	70.73	76.21	53.05
Min	59.94	64.31	69.35	40.44	30.60	39.33	37.43
DOX 0.66+NTR 16.5+TOB 4.1							
Median (n=32)	66.74	77.44	71.10	53.09	54.27	62.07	39.89
Max	74.60	103.36	84.19	57.29	58.95	71.72	50.39
Min	59.94	64.31	24.81	40.44	36.63	52.22	14.33
DOX 0.66+NTR 16.5+VAN 80							
Median (n=32)	66.74	77.44	55.55	53.09	11.12	4.46	4.80
Max	74.60	103.36	69.28	57.29	18.14	7.53	13.05
Min	59.94	64.31	22.28	40.44	-12.51	1.83	-5.36

FUS 38+GEN 4.4+NTR 16.5							
Median (n=16)	68.42	73.83	77.44	50.98	72.18	59.31	58.87
Max	95.37	86.03	103.36	61.69	80.76	64.50	70.44
Min	32.92	60.41	64.31	41.91	49.42	51.01	47.74
CLI 13.3+FUS 38+TOB 4.1							
Median (n=32)	85.73	68.42	71.10	52.71	64.55	61.10	47.22
Max	93.78	95.37	84.19	58.40	70.55	65.07	55.95
Min	82.45	32.92	24.81	49.34	42.49	44.23	32.35
CLI 13.3+FUS 38+VAN 80							
Median (n=32)	85.73	68.42	55.55	52.71	54.43	33.16	24.14
Max	93.78	95.37	69.28	58.40	62.33	37.55	34.78
Min	82.45	32.92	22.28	49.34	10.37	-11.89	18.68
CLI 13.3+DOX 0.66+ERY 3.62							
Median (n=16)	85.73	66.74	102.45	55.62	80.27	38.07	60.25
Max	93.78	74.60	112.12	57.88	84.78	53.96	71.77
Min	82.45	59.94	92.26	33.60	74.41	32.72	43.81
CLI 13.3+DOX 0.66+FOX 2.25							
Median (n=16)	85.73	66.74	80.36	55.62	55.83	14.61	26.70
Max	93.78	74.60	94.71	57.88	65.15	22.40	48.38

Min	82.45	59.94	35.04	33.60	34.19	3.46	11.95
CLI 13.3+DOX 0.66+FUS 38							
Median (n=16)	85.73	66.74	68.42	55.62	52.71	21.11	37.21
Max	93.78	74.60	95.37	57.88	58.40	27.77	42.32
Min	82.45	59.94	32.92	33.60	49.34	4.66	27.58
CLI 13.3+DOX 0.66+GEN 4.4							
Median (n=16)	85.73	66.74	73.83	55.62	52.38	47.29	50.31
Max	93.78	74.60	86.03	57.88	62.10	52.81	57.44
Min	82.45	59.94	60.41	33.60	38.88	42.22	26.08
CLI 13.3+DOX 0.66+NTR 16.5							
Median (n=16)	85.73	66.74	77.44	55.62	65.25	53.09	60.09
Max	93.78	74.60	103.36	57.88	82.74	57.29	65.49
Min	82.45	59.94	64.31	33.60	34.35	40.44	44.43
CLI 13.3+DOX 0.66+STR 8.47							
Median (n=16)	85.73	66.74	57.18	55.62	50.17	21.72	40.04
Max	93.78	74.60	77.02	57.88	66.01	44.29	46.34
Min	82.45	59.94	42.55	33.60	28.12	6.56	36.47
CLI 13.3+DOX 0.66+TMP 0.35							
Median (n=16)	85.73	66.74	80.68	55.62	65.29	54.35	52.05

Max	93.78	74.60	95.28	57.88	76.70	70.73	60.66
Min	82.45	59.94	69.35	33.60	51.25	30.60	33.02
CLI 13.3+DOX 0.66+TOB 4.1							
Median (n=32)	85.73	66.74	71.10	55.62	64.55	54.27	49.02
Max	93.78	74.60	84.19	57.88	70.55	58.95	53.29
Min	82.45	59.94	24.81	33.60	42.49	36.63	24.09
CLI 13.3+DOX 0.66+VAN 80							
Median (n=32)	85.73	66.74	55.55	55.62	54.43	11.12	18.86
Max	93.78	74.60	69.28	57.88	62.33	18.14	31.49
Min	82.45	59.94	22.28	33.60	10.37	-12.51	3.75
CLI 13.3+GEN 4.4+NTR 16.5							
Median (n=16)	85.73	73.83	77.44	52.38	65.25	59.31	66.44
Max	93.78	86.03	103.36	62.10	82.74	64.50	72.90
Min	82.45	60.41	64.31	38.88	34.35	51.01	31.84
CLI 13.3+GEN 4.4+STR 8.47							
Median (n=16)	85.73	73.83	57.18	52.38	50.17	51.96	53.68
Max	93.78	86.03	77.02	62.10	66.01	66.18	66.78
Min	82.45	60.41	42.55	38.88	28.12	33.29	36.63
CLI 13.3+GEN 4.4+TMP 0.35							

Median (n=16)	85.73	73.83	80.68	52.38	65.29	49.65	62.12
Max	93.78	86.03	95.28	62.10	76.70	72.97	66.20
Min	82.45	60.41	69.35	38.88	51.25	34.41	51.10
CLI 13.3+GEN 4.4+TOB 4.1							
Median (n=32)	85.73	73.83	71.10	52.38	64.55	59.75	54.63
Max	93.78	86.03	84.19	62.10	70.55	76.66	59.21
Min	82.45	60.41	24.81	38.88	42.49	36.72	29.39
CLI 13.3+GEN 4.4+VAN 80							
Median (n=32)	85.73	73.83	55.55	52.38	54.43	54.44	35.94
Max	93.78	86.03	69.28	62.10	62.33	68.70	46.67
Min	82.45	60.41	22.28	38.88	10.37	13.32	10.17
CLI 13.3+ERY 3.62+FOX 2.25							
Median (n=16)	85.73	102.45	80.36	80.27	55.83	62.01	51.48
Max	93.78	112.12	94.71	84.78	65.15	71.19	65.07
Min	82.45	92.26	35.04	74.41	34.19	51.25	36.59
CLI 13.3+ERY 3.62+FUS 38							
Median (n=16)	85.73	102.45	68.42	80.27	52.71	52.31	66.21
Max	93.78	112.12	95.37	84.78	58.40	65.73	72.97
Min	82.45	92.26	32.92	74.41	49.34	36.58	36.12

CLI 13.3+ERY 3.62+GEN 4.4							
Median (n=16)	85.73	102.45	73.83	80.27	52.38	67.28	66.01
Max	93.78	112.12	86.03	84.78	62.10	78.35	74.12
Min	82.45	92.26	60.41	74.41	38.88	21.17	48.42
CLI 13.3+ERY 3.62+NTR 16.5							
Median (n=16)	85.73	102.45	77.44	80.27	65.25	84.06	71.83
Max	93.78	112.12	103.36	84.78	82.74	87.25	86.32
Min	82.45	92.26	64.31	74.41	34.35	79.31	39.96
CLI 13.3+ERY 3.62+STR 8.47							
Median (n=16)	85.73	102.45	57.18	80.27	50.17	55.73	59.75
Max	93.78	112.12	77.02	84.78	66.01	64.57	65.41
Min	82.45	92.26	42.55	74.41	28.12	34.13	33.51
CLI 13.3+ERY 3.62+TMP 0.35							
Median (n=8)	85.73	102.45	80.68	80.27	65.29	80.64	74.52
Max	93.78	112.12	95.28	84.78	76.70	90.64	89.47
Min	82.45	92.26	69.35	74.41	51.25	28.20	66.01
CLI 13.3+ERY 3.62+TOB 4.1							
Median (n=32)	85.73	102.45	71.10	80.27	64.55	76.22	66.75
Max	93.78	112.12	84.19	84.78	70.55	84.26	84.75

Min	82.45	92.26	24.81	74.41	42.49	69.30	59.22
CLI 13.3+ERY 3.62+VAN 80							
Median (n=32)	85.73	102.45	55.55	80.27	54.43	71.42	51.55
Max	93.78	112.12	69.28	84.78	62.33	75.27	56.01
Min	82.45	92.26	22.28	74.41	10.37	29.07	32.75
CLI 13.3+NTR 16.5+STR 8.47							
Median (n=16)	85.73	77.44	57.18	65.25	50.17	-1.04	5.61
Max	93.78	103.36	77.02	82.74	66.01	6.07	12.88
Min	82.45	64.31	42.55	34.35	28.12	-5.76	-8.12
CLI 13.3+NTR 16.5+TMP 0.35							
Median (n=16)	85.73	77.44	80.68	65.25	65.29	59.58	56.86
Max	93.78	103.36	95.28	82.74	76.70	76.21	64.42
Min	82.45	64.31	69.35	34.35	51.25	39.33	22.72
CLI 13.3+NTR 16.5+TOB 4.1							
Median (n=32)	85.73	77.44	71.10	65.25	64.55	62.07	66.49
Max	93.78	103.36	84.19	82.74	70.55	71.72	73.58
Min	82.45	64.31	24.81	34.35	42.49	52.22	47.13
CLI 13.3+NTR 16.5+VAN 80							
Median (n=32)	85.73	77.44	55.55	65.25	54.43	4.46	6.11

Max	93.78	103.36	69.28	82.74	62.33	7.53	13.23
Min	82.45	64.31	22.28	34.35	10.37	1.83	-12.29
CLI 13.3+FOX 2.25+FUS 38							
Median (n=16)	85.73	80.36	68.42	55.83	52.71	19.20	40.46
Max	93.78	94.71	95.37	65.15	58.40	40.44	47.98
Min	82.45	35.04	32.92	34.19	49.34	12.82	33.62
CLI 13.3+FOX 2.25+GEN 4.4							
Median (n=16)	85.73	80.36	73.83	55.83	52.38	29.60	42.14
Max	93.78	94.71	86.03	65.15	62.10	33.33	51.26
Min	82.45	35.04	60.41	34.19	38.88	25.33	26.20
CLI 13.3+FOX 2.25+NTR 16.5							
Median (n=16)	85.73	80.36	77.44	55.83	65.25	46.66	42.24
Max	93.78	94.71	103.36	65.15	82.74	57.85	49.86
Min	82.45	35.04	64.31	34.19	34.35	34.72	38.97
CLI 13.3+FOX 2.25+STR 8.47							
Median (n=16)	85.73	80.36	57.18	55.83	50.17	30.49	30.78
Max	93.78	94.71	77.02	65.15	66.01	48.88	41.78
Min	82.45	35.04	42.55	34.19	28.12	-5.18	-7.02
CLI 13.3+FOX 2.25+TMP 0.35							

Median (n=8)	85.73	80.36	80.68	55.83	65.29	77.11	68.41
Max	93.78	94.71	95.28	65.15	76.70	85.42	72.35
Min	82.45	35.04	69.35	34.19	51.25	38.63	56.09
CLI 13.3+FOX 2.25+TOB 4.1							
Median (n=32)	85.73	80.36	71.10	55.83	64.55	34.80	33.90
Max	93.78	94.71	84.19	65.15	70.55	37.59	39.92
Min	82.45	35.04	24.81	34.19	42.49	31.22	22.03
CLI 13.3+FOX 2.25+VAN 80							
Median (n=32)	85.73	80.36	55.55	55.83	54.43	19.46	27.06
Max	93.78	94.71	69.28	65.15	62.33	63.31	36.40
Min	82.45	35.04	22.28	34.19	10.37	-6.12	9.59
CLI 13.3+STR 8.47+TMP 0.35							
Median (n=16)	85.73	57.18	80.68	50.17	65.29	46.20	54.70
Max	93.78	77.02	95.28	66.01	76.70	59.14	56.62
Min	82.45	42.55	69.35	28.12	51.25	39.27	42.28
CLI 13.3+STR 8.47+TOB 4.1							
Median (n=32)	85.73	57.18	71.10	50.17	64.55	56.45	50.32
Max	93.78	77.02	84.19	66.01	70.55	65.02	60.97
Min	82.45	42.55	24.81	28.12	42.49	50.40	12.76

CLI 13.3+STR 8.47+VAN 80							
Median (n=32)	85.73	57.18	55.55	50.17	54.43	35.57	32.52
Max	93.78	77.02	69.28	66.01	62.33	39.85	49.11
Min	82.45	42.55	22.28	28.12	10.37	10.50	9.73
FOX 2.25+FUS 38+GEN 4.4							
Median (n=16)	80.36	68.42	73.83	19.20	29.60	50.98	15.31
Max	94.71	95.37	86.03	40.44	33.33	61.69	34.25
Min	35.04	32.92	60.41	12.82	25.33	41.91	5.15
FOX 2.25+FUS 38+NTR 16.5							
Median (n=16)	80.36	68.42	77.44	19.20	46.66	72.18	21.80
Max	94.71	95.37	103.36	40.44	57.85	80.76	28.02
Min	35.04	32.92	64.31	12.82	34.72	49.42	19.39
FOX 2.25+FUS 38+STR 8.47							
Median (n=16)	80.36	68.42	57.18	19.20	30.49	50.71	8.60
Max	94.71	95.37	77.02	40.44	48.88	66.73	21.35
Min	35.04	32.92	42.55	12.82	-5.18	39.23	-6.94
FOX 2.25+FUS 38+TMP 0.35							
Median (n=16)	80.36	68.42	80.68	19.20	77.11	46.93	30.96
Max	94.71	95.37	95.28	40.44	85.42	65.37	34.27

Min	35.04	32.92	69.35	12.82	38.63	7.87	7.54
ERY 3.62+STR 8.47+TMP 0.35							
Median (n=16)	102.45	57.18	80.68	55.73	80.64	46.20	41.14
Max	112.12	77.02	95.28	64.57	90.64	59.14	51.77
Min	92.26	42.55	69.35	34.13	28.20	39.27	25.67
ERY 3.62+STR 8.47+TOB 4.1							
Median (n=32)	102.45	57.18	71.10	55.73	76.22	56.45	59.25
Max	112.12	77.02	84.19	64.57	84.26	65.02	65.20
Min	92.26	42.55	24.81	34.13	69.30	50.40	36.32
ERY 3.62+STR 8.47+VAN 80							
Median (n=32)	102.45	57.18	55.55	55.73	71.42	35.57	39.68
Max	112.12	77.02	69.28	64.57	75.27	39.85	50.25
Min	92.26	42.55	22.28	34.13	29.07	10.50	19.88
FOX 2.25+STR 8.47+TOB 4.1							
Median (n=32)	80.36	57.18	71.10	30.49	34.80	56.45	16.78
Max	94.71	77.02	84.19	48.88	37.59	65.02	36.62
Min	35.04	42.55	24.81	-5.18	31.22	50.40	3.72
FOX 2.25+STR 8.47+VAN 80							
Median (n=32)	80.36	57.18	55.55	30.49	19.46	35.57	16.81

Max	94.71	77.02	69.28	48.88	63.31	39.85	30.55
Min	35.04	42.55	22.28	-5.18	-6.12	10.50	0.18
FOX 2.25+GEN 4.4+NTR 16.5							
Median (n=16)	80.36	73.83	77.44	29.60	46.66	59.31	28.00
Max	94.71	86.03	103.36	33.33	57.85	64.50	37.18
Min	35.04	60.41	64.31	25.33	34.72	51.01	-7.54
FOX 2.25+GEN 4.4+STR 8.47							
Median (n=16)	80.36	73.83	57.18	29.60	30.49	51.96	16.95
Max	94.71	86.03	77.02	33.33	48.88	66.18	36.35
Min	35.04	60.41	42.55	25.33	-5.18	33.29	-10.75
FOX 2.25+GEN 4.4+TMP 0.35							
Median (n=16)	80.36	73.83	80.68	29.60	77.11	49.65	40.89
Max	94.71	86.03	95.28	33.33	85.42	72.97	46.45
Min	35.04	60.41	69.35	25.33	38.63	34.41	33.49
FOX 2.25+GEN 4.4+TOB 4.1							
Median (n=32)	80.36	73.83	71.10	29.60	34.80	59.75	19.33
Max	94.71	86.03	84.19	33.33	37.59	76.66	23.85
Min	35.04	60.41	24.81	25.33	31.22	36.72	12.26
FOX 2.25+GEN 4.4+VAN 80							

Median (n=32)	80.36	73.83	55.55	29.60	19.46	54.44	19.77
Max	94.71	86.03	69.28	33.33	63.31	68.70	26.21
Min	35.04	60.41	22.28	25.33	-6.12	13.32	16.74
GEN 4.4+STR 8.47+TMP 0.35							
Median (n=16)	73.83	57.18	80.68	51.96	49.65	46.20	43.60
Max	86.03	77.02	95.28	66.18	72.97	59.14	51.24
Min	60.41	42.55	69.35	33.29	34.41	39.27	36.16
GEN 4.4+STR 8.47+TOB 4.1							
Median (n=32)	73.83	57.18	71.10	51.96	59.75	56.45	48.54
Max	86.03	77.02	84.19	66.18	76.66	65.02	58.85
Min	60.41	42.55	24.81	33.29	36.72	50.40	17.24
GEN 4.4+STR 8.47+VAN 80							
Median (n=32)	73.83	57.18	55.55	51.96	54.44	35.57	31.90
Max	86.03	77.02	69.28	66.18	68.70	39.85	37.48
Min	60.41	42.55	22.28	33.29	13.32	10.50	26.25
NTR 16.5+STR 8.47+TMP 0.35							
Median (n=16)	77.44	57.18	80.68	-1.04	59.58	46.20	6.50
Max	103.36	77.02	95.28	6.07	76.21	59.14	13.03
Min	64.31	42.55	69.35	-5.76	39.33	39.27	0.11

NTR 16.5+STR 8.47+TOB 4.1							
Median (n=32)	77.44	57.18	71.10	-1.04	62.07	56.45	5.71
Max	103.36	77.02	84.19	6.07	71.72	65.02	12.90
Min	64.31	42.55	24.81	-5.76	52.22	50.40	-3.05
NTR 16.5+STR 8.47+VAN 80							
Median (n=32)	77.44	57.18	55.55	-1.04	4.46	35.57	3.97
Max	103.36	77.02	69.28	6.07	7.53	39.85	8.04
Min	64.31	42.55	22.28	-5.76	1.83	10.50	-6.20
FOX 2.25+NTR 16.5+STR 8.47							
Median (n=16)	80.36	77.44	57.18	46.66	30.49	-1.04	7.30
Max	94.71	103.36	77.02	57.85	48.88	6.07	13.73
Min	35.04	64.31	42.55	34.72	-5.18	-5.76	-4.93
FOX 2.25+NTR 16.5+TMP 0.35							
Median (n=16)	80.36	77.44	80.68	46.66	77.11	59.58	31.93
Max	94.71	103.36	95.28	57.85	85.42	76.21	53.94
Min	35.04	64.31	69.35	34.72	38.63	39.33	15.32
FOX 2.25+NTR 16.5+TOB 4.1							
Median (n=32)	80.36	77.44	71.10	46.66	34.80	62.07	25.57
Max	94.71	103.36	84.19	57.85	37.59	71.72	35.03

Min	35.04	64.31	24.81	34.72	31.22	52.22	15.28
FOX 2.25+NTR 16.5+VAN 80							
Median (n=32)	80.36	77.44	55.55	46.66	19.46	4.46	-2.34
Max	94.71	103.36	69.28	57.85	63.31	7.53	11.77
Min	35.04	64.31	22.28	34.72	-6.12	1.83	-19.74
GEN 4.4+TMP 0.35+TOB 4.1							
Median (n=32)	73.83	80.68	71.10	49.65	59.75	49.81	51.05
Max	86.03	95.28	84.19	72.97	76.66	65.61	64.19
Min	60.41	69.35	24.81	34.41	36.72	10.01	34.38
GEN 4.4+TMP 0.35+VAN 80							
Median (n=32)	73.83	80.68	55.55	49.65	54.44	32.45	23.12
Max	86.03	95.28	69.28	72.97	68.70	69.49	29.63
Min	60.41	69.35	22.28	34.41	13.32	23.81	-8.76
NTR 16.5+TMP 0.35+TOB 4.1							
Median (n=32)	77.44	80.68	71.10	59.58	62.07	49.81	53.33
Max	103.36	95.28	84.19	76.21	71.72	65.61	62.46
Min	64.31	69.35	24.81	39.33	52.22	10.01	38.52
NTR 16.5+TMP 0.35+VAN 80							
Median (n=32)	77.44	80.68	55.55	59.58	4.46	32.45	10.28

Max	103.36	95.28	69.28	76.21	7.53	69.49	12.70
Min	64.31	69.35	22.28	39.33	1.83	23.81	4.91
STR 8.47+TMP 0.35+TOB 4.1							
Median (n=32)	57.18	80.68	71.10	46.20	56.45	49.81	39.31
Max	77.02	95.28	84.19	59.14	65.02	65.61	50.34
Min	42.55	69.35	24.81	39.27	50.40	10.01	17.81
STR 8.47+TMP 0.35+VAN 80							
Median (n=32)	57.18	80.68	55.55	46.20	35.57	32.45	21.87
Max	77.02	95.28	69.28	59.14	39.85	69.49	38.93
Min	42.55	69.35	22.28	39.27	10.50	23.81	3.54
FOX 2.25+STR 8.47+TMP 0.35							
Median (n=16)	80.36	57.18	80.68	30.49	77.11	46.20	28.69
Max	94.71	77.02	95.28	48.88	85.42	59.14	51.21
Min	35.04	42.55	69.35	-5.18	38.63	39.27	16.72
GEN 4.4+TOB 4.1+VAN 80							
Median (n=32)	73.83	71.10	55.55	59.75	54.44	64.45	50.80
Max	86.03	84.19	69.28	76.66	68.70	69.76	57.28
Min	60.41	24.81	22.28	36.72	13.32	29.72	43.28
NTR 16.5+TOB 4.1+VAN 80							

Median (n=32)	77.44	71.10	55.55	62.07	4.46	64.45	9.83
Max	103.36	84.19	69.28	71.72	7.53	69.76	15.05
Min	64.31	24.81	22.28	52.22	1.83	29.72	1.46
STR 8.47+TOB 4.1+VAN 80							
Median (n=32)	57.18	71.10	55.55	56.45	35.57	64.45	43.40
Max	77.02	84.19	69.28	65.02	39.85	69.76	53.76
Min	42.55	24.81	22.28	50.40	10.50	29.72	37.03
TMP 0.35+TOB 4.1+VAN 80							
Median (n=32)	80.68	71.10	55.55	49.81	32.45	64.45	39.31
Max	95.28	84.19	69.28	65.61	69.49	69.76	49.43
Min	69.35	24.81	22.28	10.01	23.81	29.72	10.32
CHL 88.4+STR 8.47+TMP 0.35							
Median (n=16)	79.93	57.18	80.68	57.68	64.13	46.20	48.62
Max	89.78	77.02	95.28	65.61	70.64	59.14	57.98
Min	76.34	42.55	69.35	44.75	55.75	39.27	43.56
CHL 88.4+STR 8.47+TOB 4.1							
Median (n=32)	79.93	57.18	71.10	57.68	73.13	56.45	48.17
Max	89.78	77.02	84.19	65.61	90.95	65.02	53.05
Min	76.34	42.55	24.81	44.75	41.54	50.40	42.61

CHL 88.4+STR 8.47+VAN 80							
Median (n=32)	79.93	57.18	55.55	57.68	56.42	35.57	26.24
Max	89.78	77.02	69.28	65.61	64.37	39.85	31.31
Min	76.34	42.55	22.28	44.75	38.52	10.50	12.52
FUS 38+STR 8.47+VAN 80							
Median (n=32)	68.42	57.18	55.55	50.71	33.16	35.57	5.99
Max	95.37	77.02	69.28	66.73	37.55	39.85	25.33
Min	32.92	42.55	22.28	39.23	-11.89	10.50	-14.49
CHL 88.4+TMP 0.35+TOB 4.1							
Median (n=32)	79.93	80.68	71.10	64.13	73.13	49.81	55.10
Max	89.78	95.28	84.19	70.64	90.95	65.61	72.40
Min	76.34	69.35	24.81	55.75	41.54	10.01	35.91
CHL 88.4+TMP 0.35+VAN 80							
Median (n=32)	79.93	80.68	55.55	64.13	56.42	32.45	32.76
Max	89.78	95.28	69.28	70.64	64.37	69.49	40.49
Min	76.34	69.35	22.28	55.75	38.52	23.81	26.01
CLI 13.3+TMP 0.35+TOB 4.1							
Median (n=32)	85.73	80.68	71.10	65.29	64.55	49.81	52.29
Max	93.78	95.28	84.19	76.70	70.55	65.61	59.53

Min	82.45	69.35	24.81	51.25	42.49	10.01	28.68
CLI 13.3+TMP 0.35+VAN 80							
Median (n=32)	85.73	80.68	55.55	65.29	54.43	32.45	4.45
Max	93.78	95.28	69.28	76.70	62.33	69.49	13.28
Min	82.45	69.35	22.28	51.25	10.37	23.81	-5.76
ERY 3.62+TMP 0.35+TOB 4.1							
Median (n=32)	102.45	80.68	71.10	80.64	76.22	49.81	55.93
Max	112.12	95.28	84.19	90.64	84.26	65.61	74.32
Min	92.26	69.35	24.81	28.20	69.30	10.01	16.58
ERY 3.62+TMP 0.35+VAN 80							
Median (n=32)	102.45	80.68	55.55	80.64	71.42	32.45	36.28
Max	112.12	95.28	69.28	90.64	75.27	69.49	48.41
Min	92.26	69.35	22.28	28.20	29.07	23.81	-3.16
FOX 2.25+TMP 0.35+TOB 4.1							
Median (n=32)	80.36	80.68	71.10	77.11	34.80	49.81	39.56
Max	94.71	95.28	84.19	85.42	37.59	65.61	46.92
Min	35.04	69.35	24.81	38.63	31.22	10.01	21.13
FOX 2.25+TMP 0.35+VAN 80							
Median (n=32)	80.36	80.68	55.55	77.11	19.46	32.45	21.86

Max	94.71	95.28	69.28	85.42	63.31	69.49	28.93
Min	35.04	69.35	22.28	38.63	-6.12	23.81	-1.79
FUS 38+TMP 0.35+VAN 80							
Median (n=32)	68.42	80.68	55.55	46.93	33.16	32.45	6.59
Max	95.37	95.28	69.28	65.37	37.55	69.49	22.89
Min	32.92	69.35	22.28	7.87	-11.89	23.81	-11.29
CHL 88.4+TOB 4.1+VAN 80							
Median (n=32)	79.93	71.10	55.55	73.13	56.42	64.45	50.35
Max	89.78	84.19	69.28	90.95	64.37	69.76	61.76
Min	76.34	24.81	22.28	41.54	38.52	29.72	11.29
CLI 13.3+TOB 4.1+VAN 80							
Median (n=32)	85.73	71.10	55.55	64.55	54.43	64.45	48.20
Max	93.78	84.19	69.28	70.55	62.33	69.76	61.77
Min	82.45	24.81	22.28	42.49	10.37	29.72	11.14
CPR 0.04+TOB 4.1+VAN 80							
Median (n=32)	68.75	71.10	55.55	56.65	27.31	64.45	21.98
Max	81.60	84.19	69.28	59.64	35.95	69.76	35.02
Min	61.44	24.81	22.28	7.91	7.10	29.72	-14.55
ERY 3.62+TOB 4.1+VAN 80							

Median (n=32)	102.45	71.10	55.55	76.22	71.42	64.45	59.31
Max	112.12	84.19	69.28	84.26	75.27	69.76	79.20
Min	92.26	24.81	22.28	69.30	29.07	29.72	18.07
FOX 2.25+TOB 4.1+VAN 80							
Median (n=32)	80.36	71.10	55.55	34.80	19.46	64.45	23.09
Max	94.71	84.19	69.28	37.59	63.31	69.76	35.57
Min	35.04	24.81	22.28	31.22	-6.12	29.72	-7.43
FUS 38+TOB 4.1+VAN 80							
Median (n=32)	68.42	71.10	55.55	61.10	33.16	64.45	3.82
Max	95.37	84.19	69.28	65.07	37.55	69.76	35.92
Min	32.92	24.81	22.28	44.23	-11.89	29.72	-15.38
CPR 0.04+DOX 0.66+ERY 3.62							
Median (n=8)	68.75	66.74	102.45	40.45	63.47	38.07	37.80
Max	81.60	74.60	112.12	47.06	70.41	53.96	42.59
Min	61.44	59.94	92.26	25.69	59.44	32.72	30.86
CPR 0.04+DOX 0.66+FOX 2.25							
Median (n=8)	68.75	66.74	80.36	40.45	28.68	14.61	18.14
Max	81.60	74.60	94.71	47.06	35.14	22.40	30.86
Min	61.44	59.94	35.04	25.69	23.57	3.46	2.73

CPR 0.04+DOX 0.66+FUS 38							
Median (n=16)	68.75	66.74	68.42	40.45	61.41	21.11	18.18
Max	81.60	74.60	95.37	47.06	63.42	27.77	22.92
Min	61.44	59.94	32.92	25.69	55.16	4.66	11.14
CPR 0.04+DOX 0.66+GEN 4.4							
Median (n=16)	68.75	66.74	73.83	40.45	43.85	47.29	34.74
Max	81.60	74.60	86.03	47.06	55.82	52.81	38.96
Min	61.44	59.94	60.41	25.69	40.21	42.22	26.90
FOX 2.25+FUS 38+TOB 4.1							
Median (n=32)	80.36	68.42	71.10	19.20	34.80	61.10	12.20
Max	94.71	95.37	84.19	40.44	37.59	65.07	17.35
Min	35.04	32.92	24.81	12.82	31.22	44.23	7.72
FOX 2.25+FUS 38+VAN 80							
Median (n=32)	80.36	68.42	55.55	19.20	19.46	33.16	2.18
Max	94.71	95.37	69.28	40.44	63.31	37.55	4.60
Min	35.04	32.92	22.28	12.82	-6.12	-11.89	-0.90
GEN 4.4+NTR 16.5+STR 8.47							
Median (n=16)	73.83	77.44	57.18	59.31	51.96	-1.04	3.92
Max	86.03	103.36	77.02	64.50	66.18	6.07	11.33

Min	60.41	64.31	42.55	51.01	33.29	-5.76	-3.90
GEN 4.4+NTR 16.5+TMP 0.35							
Median (n=16)	73.83	77.44	80.68	59.31	49.65	59.58	48.38
Max	86.03	103.36	95.28	64.50	72.97	76.21	49.45
Min	60.41	64.31	69.35	51.01	34.41	39.33	37.77
GEN 4.4+NTR 16.5+TOB 4.1							
Median (n=32)	73.83	77.44	71.10	59.31	59.75	62.07	3.44
Max	86.03	103.36	84.19	64.50	76.66	71.72	8.93
Min	60.41	64.31	24.81	51.01	36.72	52.22	-1.44
GEN 4.4+NTR 16.5+VAN 80							
Median (n=32)	73.83	77.44	55.55	59.31	54.44	4.46	2.17
Max	86.03	103.36	69.28	64.50	68.70	7.53	7.12
Min	60.41	64.31	22.28	51.01	13.32	1.83	-6.71
FUS 38+GEN 4.4+STR 8.47							
Median (n=16)	68.42	73.83	57.18	50.98	50.71	51.96	43.18
Max	95.37	86.03	77.02	61.69	66.73	66.18	48.14
Min	32.92	60.41	42.55	41.91	39.23	33.29	24.95
FUS 38+GEN 4.4+TMP 0.35							
Median (n=16)	68.42	73.83	80.68	50.98	46.93	49.65	49.76

Max	95.37	86.03	95.28	61.69	65.37	72.97	55.09
Min	32.92	60.41	69.35	41.91	7.87	34.41	32.98
FUS 38+GEN 4.4+TOB 4.1							
Median (n=32)	68.42	73.83	71.10	50.98	61.10	59.75	44.46
Max	95.37	86.03	84.19	61.69	65.07	76.66	55.24
Min	32.92	60.41	24.81	41.91	44.23	36.72	21.77
FUS 38+GEN 4.4+VAN 80							
Median (n=32)	68.42	73.83	55.55	50.98	33.16	54.44	12.66
Max	95.37	86.03	69.28	61.69	37.55	68.70	43.41
Min	32.92	60.41	22.28	41.91	-11.89	13.32	4.47
CPR 0.04+ERY 3.62+FOX 2.25							
Median (n=16)	68.75	102.45	80.36	63.47	28.68	62.01	26.10
Max	81.60	112.12	94.71	70.41	35.14	71.19	35.69
Min	61.44	92.26	35.04	59.44	23.57	51.25	15.84
CPR 0.04+ERY 3.62+FUS 38							
Median (n=16)	68.75	102.45	68.42	63.47	61.41	52.31	56.37
Max	81.60	112.12	95.37	70.41	63.42	65.73	62.23
Min	61.44	92.26	32.92	59.44	55.16	36.58	46.62
CPR 0.04+ERY 3.62+GEN 4.4							

Median (n=16)	68.75	102.45	73.83	63.47	43.85	67.28	55.39
Max	81.60	112.12	86.03	70.41	55.82	78.35	66.28
Min	61.44	92.26	60.41	59.44	40.21	21.17	50.00
CPR 0.04+ERY 3.62+NTR 16.5							
Median (n=16)	68.75	102.45	77.44	63.47	40.46	84.06	51.25
Max	81.60	112.12	103.36	70.41	47.50	87.25	59.39
Min	61.44	92.26	64.31	59.44	34.96	79.31	42.98
CPR 0.04+ERY 3.62+STR 8.47							
Median (n=16)	68.75	102.45	57.18	63.47	46.40	55.73	45.13
Max	81.60	112.12	77.02	70.41	50.59	64.57	55.90
Min	61.44	92.26	42.55	59.44	32.43	34.13	23.90
CPR 0.04+ERY 3.62+TMP 0.35							
Median (n=16)	68.75	102.45	80.68	63.47	38.56	80.64	44.18
Max	81.60	112.12	95.28	70.41	50.13	90.64	52.61
Min	61.44	92.26	69.35	59.44	7.17	28.20	36.53
CPR 0.04+ERY 3.62+TOB 4.1							
Median (n=32)	68.75	102.45	71.10	63.47	56.65	76.22	53.71
Max	81.60	112.12	84.19	70.41	59.64	84.26	62.45
Min	61.44	92.26	24.81	59.44	7.91	69.30	35.98

CPR 0.04+ERY 3.62+VAN 80							
Median (n=32)	68.75	102.45	55.55	63.47	27.31	71.42	28.39
Max	81.60	112.12	69.28	70.41	35.95	75.27	38.47
Min	61.44	92.26	22.28	59.44	7.10	29.07	21.99
DOX 0.66+STR 8.47+TMP 0.35							
Median (n=16)	66.74	57.18	80.68	21.72	54.35	46.20	26.81
Max	74.60	77.02	95.28	44.29	70.73	59.14	46.29
Min	59.94	42.55	69.35	6.56	30.60	39.27	20.09
DOX 0.66+STR 8.47+TOB 4.1							
Median (n=32)	66.74	57.18	71.10	21.72	54.27	56.45	6.40
Max	74.60	77.02	84.19	44.29	58.95	65.02	22.07
Min	59.94	42.55	24.81	6.56	36.63	50.40	1.45
DOX 0.66+STR 8.47+VAN 80							
Median (n=32)	66.74	57.18	55.55	21.72	11.12	35.57	7.87
Max	74.60	77.02	69.28	44.29	18.14	39.85	13.77
Min	59.94	42.55	22.28	6.56	-12.51	10.50	-5.52
FUS 38+NTR 16.5+TMP 0.35							
Median (n=16)	68.42	77.44	80.68	72.18	46.93	59.58	64.67
Max	95.37	103.36	95.28	80.76	65.37	76.21	73.30

Min	32.92	64.31	69.35	49.42	7.87	39.33	21.22
FUS 38+NTR 16.5+TOB 4.1							
Median (n=32)	68.42	77.44	71.10	72.18	61.10	62.07	48.97
Max	95.37	103.36	84.19	80.76	65.07	71.72	62.46
Min	32.92	64.31	24.81	49.42	44.23	52.22	30.09
FUS 38+NTR 16.5+VAN 80							
Median (n=32)	68.42	77.44	55.55	72.18	33.16	4.46	3.67
Max	95.37	103.36	69.28	80.76	37.55	7.53	13.68
Min	32.92	64.31	22.28	49.42	-11.89	1.83	-11.43
CPR 0.04+FOX 2.25+FUS 38							
Median (n=16)	68.75	80.36	68.42	28.68	61.41	19.20	26.29
Max	81.60	94.71	95.37	35.14	63.42	40.44	32.90
Min	61.44	35.04	32.92	23.57	55.16	12.82	15.65
CPR 0.04+FOX 2.25+GEN 4.4							
Median (n=16)	68.75	80.36	73.83	28.68	43.85	29.60	19.16
Max	81.60	94.71	86.03	35.14	55.82	33.33	29.00
Min	61.44	35.04	60.41	23.57	40.21	25.33	7.06
CPR 0.04+FOX 2.25+NTR 16.5							
Median (n=16)	68.75	80.36	77.44	28.68	40.46	46.66	25.52

Max	81.60	94.71	103.36	35.14	47.50	57.85	36.00
Min	61.44	35.04	64.31	23.57	34.96	34.72	16.38
CPR 0.04+FOX 2.25+STR 8.47							
Median (n=16)	68.75	80.36	57.18	28.68	46.40	30.49	21.81
Max	81.60	94.71	77.02	35.14	50.59	48.88	36.38
Min	61.44	35.04	42.55	23.57	32.43	-5.18	19.44
CPR 0.04+FOX 2.25+TMP 0.35							
Median (n=16)	68.75	80.36	80.68	28.68	38.56	77.11	36.69
Max	81.60	94.71	95.28	35.14	50.13	85.42	40.16
Min	61.44	35.04	69.35	23.57	7.17	38.63	26.99
CPR 0.04+FOX 2.25+TOB 4.1							
Median (n=32)	68.75	80.36	71.10	28.68	56.65	34.80	24.82
Max	81.60	94.71	84.19	35.14	59.64	37.59	27.09
Min	61.44	35.04	24.81	23.57	7.91	31.22	2.60
CPR 0.04+FOX 2.25+VAN 80							
Median (n=32)	68.75	80.36	55.55	28.68	27.31	19.46	21.40
Max	81.60	94.71	69.28	35.14	35.95	63.31	26.88
Min	61.44	35.04	22.28	23.57	7.10	-6.12	16.89
DOX 0.66+TMP 0.35+TOB 4.1							

Median (n=32)	66.74	80.68	71.10	54.35	54.27	49.81	44.83
Max	74.60	95.28	84.19	70.73	58.95	65.61	64.95
Min	59.94	69.35	24.81	30.60	36.63	10.01	28.27
DOX 0.66+TMP 0.35+VAN 80							
Median (n=32)	66.74	80.68	55.55	54.35	11.12	32.45	13.26
Max	74.60	95.28	69.28	70.73	18.14	69.49	26.92
Min	59.94	69.35	22.28	30.60	-12.51	23.81	-2.55
FUS 38+NTR 16.5+STR 8.47							
Median (n=16)	68.42	77.44	57.18	72.18	50.71	-1.04	4.82
Max	95.37	103.36	77.02	80.76	66.73	6.07	68.67
Min	32.92	64.31	42.55	49.42	39.23	-5.76	-14.92
FUS 38+STR 8.47+TMP 0.35							
Median (n=16)	68.42	57.18	80.68	50.71	46.93	59.58	50.94
Max	95.37	77.02	95.28	66.73	65.37	76.21	62.41
Min	32.92	42.55	69.35	39.23	7.87	39.33	12.08
FUS 38+STR 8.47+TOB 4.1							
Median (n=32)	68.42	57.18	71.10	50.71	61.10	62.07	45.73
Max	95.37	77.02	84.19	66.73	65.07	71.72	66.65
Min	32.92	42.55	24.81	39.23	44.23	52.22	8.11

CPR 0.04+FUS 38+GEN 4.4							
Median (n=16)	68.75	68.42	73.83	61.41	43.85	50.98	56.05
Max	81.60	95.37	86.03	63.42	55.82	61.69	64.47
Min	61.44	32.92	60.41	55.16	40.21	41.91	23.35
CPR 0.04+FUS 38+NTR 16.5							
Median (n=16)	68.75	68.42	77.44	61.41	40.46	72.18	62.83
Max	81.60	95.37	103.36	63.42	47.50	80.76	67.93
Min	61.44	32.92	64.31	55.16	34.96	49.42	56.00
CPR 0.04+FUS 38+STR 8.47							
Median (n=16)	68.75	68.42	57.18	61.41	46.40	50.71	52.11
Max	81.60	95.37	77.02	63.42	50.59	66.73	57.84
Min	61.44	32.92	42.55	55.16	32.43	39.23	40.92
CPR 0.04+FUS 38+TMP 0.35							
Median (n=16)	68.75	68.42	80.68	61.41	38.56	46.93	52.27
Max	81.60	95.37	95.28	63.42	50.13	65.37	58.21
Min	61.44	32.92	69.35	55.16	7.17	7.87	46.93
CPR 0.04+FUS 38+TOB 4.1							
Median (n=32)	68.75	68.42	71.10	61.41	56.65	61.10	57.46
Max	81.60	95.37	84.19	63.42	59.64	65.07	66.07

Min	61.44	32.92	24.81	55.16	7.91	44.23	51.62
CPR 0.04+FUS 38+VAN 80							
Median (n=32)	68.75	68.42	55.55	61.41	27.31	33.16	12.18
Max	81.60	95.37	69.28	63.42	35.95	37.55	16.88
Min	61.44	32.92	22.28	55.16	7.10	-11.89	-9.14
DOX 0.66+TOB 4.1+VAN 80							
Median (n=32)	66.74	71.10	55.55	54.27	11.12	64.45	12.26
Max	74.60	84.19	69.28	58.95	18.14	69.76	17.38
Min	59.94	24.81	22.28	36.63	-12.51	29.72	-10.51
FUS 38+TMP 0.35+TOB 4.1							
Median (n=32)	68.42	80.68	71.10	46.93	61.10	49.81	53.48
Max	95.37	95.28	84.19	65.37	65.07	65.61	65.54
Min	32.92	69.35	24.81	7.87	44.23	10.01	-0.05
AMP 3.56+TOB 4.1+VAN 80							
Median (n=32)	79.51	71.10	55.55	47.33	9.58	64.45	22.81
Max	98.05	84.19	69.28	50.75	32.33	69.76	26.88
Min	64.70	24.81	22.28	38.11	-5.26	29.72	11.50

Supplementary Table 2. Emergent suppressive three-drug combinations from 14 antibiotics (see Materials and Methods, Fig 1) for *E. coli* CFT073, *E. coli* BW25113, *S. epidermidis* 14990.

E. coli CFT073		*E. coli* BW25113		*S. epidermidis* 14990	
**Combinations**	**E3**	**Combinations**	**E3**	**Combinations**	**E3**
AMP+CHL+VAN	1.7231	CLI+ERY+TOB	1.7323	AMP+CPR+TOB	4.4405
AMP+CLI+DOX	2.1715	CLI+FOX+STR	2.4922	CHL+FUS+STR	1.6223
AMP+CLI+FUS	1.4327	CLI+FOX+TOB	2.2007	CHL+CLI+GEN	1.3818
AMP+CLI+VAN	2.3609	CPR+ERY+STR	3.7891	CLI+FUS+STR	3.7604
AMP+TMP+VAN	5.3773	CPR+ERY+TOB	1.5561	CLI+GEN+TMP	3.8297
AMP+DOX+NTR	3.8918	CPR+FOX+TOB	1.6954	CLI+ERY+GEN	7.4911
AMP+ERY+NTR	6.1195	ERY+FOX+STR	13.8269	CLI+FOX+GEN	3.4186
DOX+ERY+NTR	4.6264	ERY+FOX+TOB	3.92	CLI+FOX+TMP	1.5959
DOX+ERY+TOB	4.8466	AMP+CPR+NTR	131.6786	FUS+GEN+TMP	3.1702
AMP+FOX+NTR	1.3583	NTR+CPR+FOX	2.9006	FUS+STR+VAN	68.1655
DOX+FOX+NTR	1.8473	NTR+CLI+FOX	2.5142	CPR+FUS+GEN	8.3327
DOX+FOX+TOB	1.7685	NTR+CLI+TOB	2.1255	CPR+FUS+TMP	3.8593
AMP+FUS+NTR	25.6027	NTR+ERY+STR	2.213	CPR+FUS+TOB	4.6025
AMP+FUS+TMP	1.5329	NTR+ERY+TOB	1.8461	FUS+STR+TMP	3.3908
DOX+FUS+NTR	3.1113	GEN+TMP+FOX	1.9109	FOX+FUS+STR	2.1196
AMP+GEN+STR	1.4666	GEN+CHL+TMP	4.5399	STR+TOB+VAN	2.3048
AMP+GEN+TMP	2.6184	GEN+NTR+FOX	30.5413	FUS+TMP+VAN	2.5293
CHL+CLI+CPR	1.5146	GEN+TOB+FOX	1.5219	CLI+ERY+NTR	1.3222
CHL+CLI+ERY	1.3513	GEN+CLI+TOB	2.0188	CPR+ERY+NTR	6.1404
CHL+CLI+GEN	1.5158	GEN+CHL+FOX	1.9254	CPR+NTR+VAN	59.6593
CPR+GEN+TOB	1.6777	GEN+ERY+FOX	2.3976	FOX+GEN+NTR	1.999
CHL+CPR+GEN	9.4774	FUS+CHL+TOB	1.8968	FOX+NTR+STR	1.468

CHL+CPR+NTR	2.3141	VAN+CHL+TMP	4.3274	FUS+GEN+NTR	2.0084
CHL+CPR+TMP	2.7167	CHL+TMP+CPR	2.5219	FUS+NTR+TMP	1.5059
CHL+FOX+TMP	2.0377	CHL+AMP+TOB	2.4369	CLI+CPR+STR	2.1775
CPR+NTR+STR	5.2644	CHL+CPR+CLI	3.1859	DOX+ERY+FOX	1.5507
CHL+DOX+ERY	6.0661	CHL+CPR+FOX	2.314	AMP+STR+TMP	2.6504
CHL+ERY+FUS	1.5534	CHL+FOX+STR	69.0205	GEN+STR+TMP	1.9295
CHL+ERY+GEN	3.0295	CHL+FOX+NTR	60.0554	DOX+STR+TMP	2.1796
CPR+TMP+TOB	1.8925	TMP+ERY+STR	306.9707	CPR+STR+TMP	2.5767
CLI+FUS+NTR	1.5853	TMP+ERY+TOB	5.0596	CPR+DOX+STR	2.1326
ERY+FOX+TMP	43.9075	VAN+STR+ERY	2.1534	CPR+DOX+ERY	3.115
ERY+FOX+VAN	4.9611	VAN+CHL+STR	1.7914	AMP+GEN+VAN	7.2899
ERY+FUS+GEN	1.5191	VAN+CHL+CLI	32.7416	DOX+ERY+TOB	7.2241
ERY+FUS+TOB	9.3363	FUS+NTR+STR	45.9139	DOX+ERY+VAN	8.2728
ERY+GEN+NTR	55.9415	GEN+CHL+CPR	2.7453		
ERY+GEN+STR	2.1248	DOX+GEN+FOX	1.9964		
ERY+GEN+TMP	2.4965	DOX+FOX+CPR	1.3859		
ERY+NTR+STR	1.5997	DOX+TOB+CPR	2.2834		
CLI+CPR+DOX	1.5056	DOX+CHL+TMP	1.4192		
DOX+NTR+STR	1.4262	DOX+CHL+FOX	2.9094		
CLI+DOX+ERY	3.077	DOX+CPR+ERY	10.64		
CLI+DOX+FOX	1.9993	DOX+NTR+AMP	35.2697		
CLI+DOX+FUS	2.993	DOX+VAN+NTR	1.7336		
CLI+DOX+NTR	1.731	DOX+VAN+CLI	1.9177		
CLI+DOX+STR	6.6398	DOX+TMP+STR	8.8322		
CLI+DOX+VAN	2.7423				
CLI+GEN+NTR	2.2981				

CLI+GEN+STR	1.3187
CLI+GEN+TMP	1.6645
CLI+GEN+TOB	1.36
CLI+ERY+FUS	1.8109
CLI+ERY+GEN	2.4136
CLI+ERY+NTR	3.5567
CLI+ERY+STR	3.1921
CLI+ERY+TMP	3.0736
CLI+ERY+TOB	3.3542
CLI+NTR+STR	22.9036
CLI+NTR+TOB	1.6425
CLI+FOX+FUS	2.2916
CLI+FOX+GEN	1.5537
CLI+FOX+TMP	4.7128
CLI+FOX+VAN	2.6637
CLI+STR+TMP	2.0782
FOX+FUS+NTR	4.9262
FOX+FUS+TMP	6.9777
FOX+GEN+TMP	3.4398
NTR+STR+TMP	5.0348
FOX+NTR+STR	1.3498
NTR+TMP+TOB	1.46
NTR+TMP+VAN	1.4826
CHL+STR+TMP	12.4006
CLI+TMP+TOB	1.3554
FOX+TMP+TOB	2.17

FOX+TMP+VAN	1.3481
CPR+ERY+FUS	1.7253
CPR+ERY+GEN	2.3743
CPR+ERY+NTR	22.3366
CPR+ERY+TMP	2.3071
DOX+STR+TMP	2.3158
FUS+NTR+TMP	5.0862
CPR+FOX+FUS	1.5605
CPR+FOX+TMP	1.7484
FUS+STR+TMP	3.589
CPR+FUS+GEN	5.1754
CPR+FUS+STR	2.7441
CPR+FUS+TMP	3.1839
FUS+TMP+TOB	1.7543

Supplementary References

Tekin, E., Beppler, C., White, C., Mao, Z., Savage, V. M. and Yeh, P. J. (2016) 'Enhanced identification of synergistic and antagonistic emergent interactions among three or more drugs', *J R Soc Interface,* 13(119).
